# Global gene expression in two potato cultivars in response to ‘*Candidatus* Liberibacter solanacearum’ infection

**DOI:** 10.1186/s12864-017-4313-2

**Published:** 2017-12-11

**Authors:** Julien G. Levy, Azucena Mendoza, J. Creighton Miller, Cecilia Tamborindeguy, Elizabeth A. Pierson

**Affiliations:** 1 Department of Horticultural Sciences, Texas A&M University, College Station, TX 77843 USA; 2 Department of Entomology, Texas A&M University, College Station, TX 77843 USA

**Keywords:** Transcriptome, Potato, Zebra Chip, *Candidatus* Liberibacter solanacearum, Psyllids

## Abstract

**Background:**

Transcriptomic analyses were performed to compare the molecular responses of two potato varieties previously shown to differ in the severity of disease symptoms due to infection by “*Candidatus* Liberibacter solanacearum” (Lso), the causative agent of Zebra Chip in potato. A factorial design utilizing the two varieties and psyllids either harboring Lso or without bacteria was used to discriminate varietal responses to pathogen infection versus psyllid feeding. Plant response was determined from leaf samples 3 weeks after infection.

**Results:**

In response to Lso infection, 397 genes were differentially expressed in the variety Atlantic (most susceptible) as compared to 1027 genes in Waneta. Over 80% of the transcriptionally-changed genes were down-regulated in both varieties, including genes involved in photosynthesis or primary and secondary metabolism. Many of the Lso-responsive genes involved in stress responses or hormonal pathways were regulated differently in the two potato varieties.

**Conclusions:**

This study focused on the time point just prior to the onset of symptom development and provides valuable insight into the mechanisms of *Liberibacter* pathogenicity, especially the widespread suppression of plant gene expression, including genes involved in plant defenses.

**Electronic supplementary material:**

The online version of this article (10.1186/s12864-017-4313-2) contains supplementary material, which is available to authorized users.

## Background

‘*Candidatus* Liberibacter solanacearum’ (Lso) are Gram-negative, phloem-limited, nonculturable bacteria. Lso is the causative agent of the Zebra Chip in potato [28, 48]. This pathogen is vectored to potato and other solanaceous hosts by the potato psyllid, *Bactericera cockerelli* (Hemiptera: Triozidae) [19, 34]. Lso has impacted potato production in Mexico since 1994, in Texas since 2000, and more recently in the Pacific Northwest of the US [9, 10]. Zebra Chip (ZC) is also a threat in Central America and New Zealand [1, 28, 47]. Lso affects other crops such as carrot, celery and parsnip in several countries in Europe and Africa [18], where it is vectored by several species of carrot psyllids. Lso can be seed transmitted in carrot, celery [6, 51] and chili pepper [7, 43]. Because of the emergence of Lso as a worldwide threat to different crops, studies such as this one aimed at elucidating plant defense mechanisms targeting Ca. Liberibacter species have global importance.

Potato (*Solanum tuberosum* L.) is the most important vegetable crop in the US and the third most important worldwide based on human consumption. Potato yields, on average per acre, more food and protein than cereals [13, 37]. Zebra Chip affects all cultivated potatoes, resulting in increased production costs and revenue losses in the US, Mexico, Central America and New Zealand [33]. Zebra Chip symptoms in potato include curling, purple coloration, and chlorosis on the youngest leaves. As the disease progresses, plants develop shortened and swollen internodes, aerial tubers and wilting; ultimately, plants may die prematurely [28, 34]. Fresh tubers from infected plants develop a characteristic brown discoloration when cut [10]; however, fresh tuber symptoms do not appear in all potato varieties. The trademark of the disease is the development of dark stripes when tubers are fried, resulting in tubers that are unsuitable for the production of potato chips or French fries [47]. Previous studies showed that all potato market classes tested, including fresh market, chip, fry and processing varieties, were susceptible to ZC [27, 32]. Although all commercial potato varieties are susceptible to ZC, understanding the host molecular responses associated with Lso infection could facilitate the identification of genes and pathways involved in pathogen virulence and host recognition and/or disease development. Knowledge of these molecular interactions is important for the development of disease management strategies such as directed breeding or gene editing for resistance.

Transcriptomic analysis is a useful tool for investigating the effects of biotic and abiotic stresses on genome-wide gene expression patterns. However, like many other crops, cultivated potato varieties are polyploids; they are typically autotetraploids (4n = 48), so there are four interchangeable genes at each locus. Despite this complexity, transcriptomic analyses have been used successfully in other potato studies [2, 31, 58]. In the current study, we used a transcriptomic approach to analyze plant responses to Lso. A two-by-two factorial design was used that included two potato varieties: Atlantic, a susceptible variety, and Waneta (formerly known as NY138), a variety that develops milder and delayed ZC symptoms [26], and two infection treatments: infestation of potato by psyllids infected with Lso (Lso+) or not (Lso-). The plants were sampled 3 weeks after infestation, a time point that corresponds with the detection of Lso by PCR and qPCR in both varieties, but the onset of the first aerial symptoms only in Atlantic [26]. We focused on this time point because it was likely to show the greatest difference between varieties in their response to Lso infection. The aims of this study were to identify plant responses to Lso infection that were either conserved or different among two potato varieties with different degrees of susceptibility to Zebra Chip, in terms of the rate of symptom progression. The results of the present study provide novel insights into the genome-wide responses of the two varieties to Lso infection, including the expression of genes involved in photosynthesis, cell wall synthesis and metabolism and abiotic/biotic stress signaling.

## Methods

### Plant material and plant growth

Two potato varieties were used in the study: Atlantic and Waneta. The Atlantic seed pieces were produced in Dalhart, TX and the Waneta seed pieces were obtained from Childstock farms, NY. For each variety, eight tuber seeds were planted individually in one-gallon pots containing autoclaved (1 h 121 °C) potting mix (MetroMix 300). The plants were maintained in a growth chamber at 24 °C with 50% humidity and a 16:8 h day:night light cycle. Plants were watered three times a week to field capacity.

### Insect source

Potato psyllids (*Bactericera cockerelli*) were obtained from a colony harboring both LsoA and LsoB haplotypes (Lso+) or from a colony that does not harbor Lso (Lso-) [56]. The colonies were tested every 6 weeks prior their use for the presence (Lso+) or absence (Lso-) of Lso by PCR [25]. All insects were from the Northwestern haplotype.

### Experimental procedure and tissue sampling

**Fig. 1:**
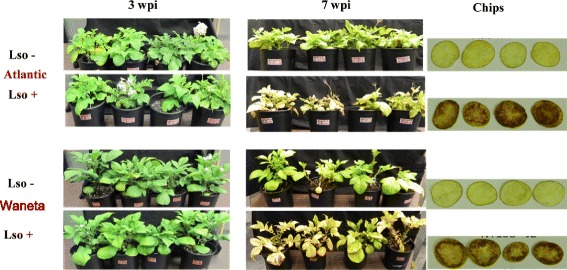
Leaf and chip symptoms 3 and 7 weeks post infestation. The two potato varieties Atlantic and Waneta developed ZC symptoms in aerial tissues and potato tubers. As reported previously, Waneta plants developed modest symptoms between weeks 3–4 compared to Atlantic plants, which typically develop obvious symptoms during that time, whereas both varieties generally have prominent symptoms by week 7 [26, 27]. Atlantic typically experiences a rapid decline immediately after week 6, leading to death between weeks7–8, whereas the decline is slower in Waneta . Thus, the two photo dates show minimal differences between the Lso + plants of the two varieties at 3 wpi, concurrent with the onset of symptom development, and at 7 wpi, where both have prominent symptoms

Four weeks after sprouting, plants from each cultivar were randomly assigned to each treatment (Lso + or Lso-). In total, there were eight replicate plants for each cultivar (Waneta and Atlantic); four plants of each cultivar received one or the other of the two insect treatments (Lso + or Lso-) (Fig. 1). Each plant was infested with three adult insects: either Lso + or Lso-. Insects were maintained in an organza bag placed on a single leaf located in the middle tier of plant leaves. After a 7-day inoculation access period, the leaf with the organza bag containing the insects was removed from the plant so that there would be no opportunity for insect escape and cross contamination.

Samples for RNA extraction were collected from the upper-most leaf of each plant at 3 weeks post infestation (wpi). All leaf samples were immediately flash frozen in liquid nitrogen and stored at −80 °C until RNA extraction. After sampling, the plants were maintained in the same conditions until the end of the experiment, which was 7 weeks post infestation. At the end of the experiment, leaf samples were collected and tested for Lso infection via PCR analysis of DNA samples extracted from the leaf mid-vein. Tuber samples were chipped and fried to detect ZC symptoms [27]. Only three replicates were used in the transcriptomic analysis.

### DNA extraction and Lso detection using PCR

DNA extraction and Lso detection by PCR were performed following methods regularly used in our laboratory. For DNA extraction from plant tissue, the protocol previously published [40] was followed, and for insects, the fast protocol [25] was used. The PCR primers used were LsoTX [36, 40].

### RNA extraction and cDNA library preparation

Total RNA from 100 mg of leaf tissue (including the central vein) from twelve plants was isolated: e.g., three biological replicates from each cultivar (Atlantic and Waneta) under two Lso treatments (Lso + and Lso-). Total RNA was isolated using the RNeasy Plant kit (Qiagen) according to the manufacturer’s protocol. Samples were treated with Turbo DNase (Ambion) following manufacturer recommendations to remove genomic DNA contamination. Absence of DNA contamination in the RNA samples was verified by PCR. RNA quantification and quality assurance were performed using a Bioanalyzer (Agilent Technologies). The construction of the cDNA libraries (TruSeq RNA, Illumina) and sequencing were performed by the Texas A&M AgriLife Genomics & Bioinformatics Services. One lane of the Illumina HiSeq 2000 was used for the 12 samples with the sequencing format of 130 bp single read.

### Transcriptomic analyses

Raw reads were trimmed to remove left over adapters using the program Cutadapt v1.1 [http://dx.doi.org/10.14806/ej.17.1.200], and then remaining bases with a minimum quality score of 15 were trimmed and filtered to a minimum length of 30 bp using the FASTX-Toolkit (http://hannonlab.cshl.edu/fastx_toolkit/). Low quality reads were discarded. The Texas A&M Brazos Cluster was used for file manipulation. All the bioinformatics analyses were performed using the cloud computing in CyVerse within the Discovery Environment [17]. The RNA-seq reads that passed the quality filters (FASTQC tools, [4]) were mapped to the potato reference genome (PGSC_DM_v3.4 gene models ensemblv19-preinstalled in CyVerse) using the Tuxedo RNA-seq pipeline [53]. The Tuxedo pipeline was comprised of TopHat2 (mapping), Cufflinks2 (transcript assembly), Cuffmerge2 (transcript merging), and Cuffdiff2 (differential gene and transcript expression). For TopHat2 the standard (per default) parameters for an Illumina 1.9 (PHRED33) were used. Determination of differential gene expression in response to Lso infection was made with Cuffdiff2 using the defaults parameters with multiple hit correction and upperquartile normalization (Minimum per-locus counts for significance testing: 10). Analyses to identify Lso-responsive genes were performed separately for each potato variety by comparing the transcriptomic responses of plants treated with Lso- insects to those treated with Lso + insects. We chose the significantly DEGs based on q value below 0.05.

For Gene Ontology (GO) enrichment analysis, the web based tool gprofiler (http://biit.cs.ut.ee/gprofiler/) was used with the default settings and Benjamini–Hochberg False Discovery Rate for significance threshold with a value of 0.05 [42]. The Open Source MapMan software was used to generate diagrams of the biologic and metabolic pathways that were differentially expressed in Atlantic and Waneta in response to Lso infection [52]. The data discussed in this publication have been deposited in the NCBI Gene Expression Omnibus [12] and are accessible through GEO Series accession number GSE92312 (https://www.ncbi.nlm.nih.gov/geo/query/acc.cgi?acc= GSE92312).

### Real-time PCR (RT-qPCR) quantitative analysis

Real-time quantitative PCR analyses were conducted to confirm the results obtained by RNA-seq analysis. Synthesis of cDNA was performed on eight different samples obtained from two separate plants from each treatment, e.g., plants infested with Lso + or Lso- psyllids from each potato variety (Atlantic, Waneta). Reverse transcription of RNA samples was performed using the Verso cDNA Synthesis kit (Thermo, Waltham, MA) according to the manufacturer’s instructions. Elongation factor 1a (Ef1a) was chosen as the reference gene [38].

A total of 19 genes were chosen for qPCR analysis: ten differentially expressed genes (DEGs) identified in both varieties as well as five genes that were transcriptionally-changed only in Atlantic and four genes transcriptionally changed only in Waneta. Primers were obtained from idtDNA (www.idtDNA.com) using the idtDNA primer design software, and all primer sequences are listed in Additional file 1: Table S1.

For qPCR amplification, each reaction contained 5 ng of cDNA, 250 nM of each primer and 1X of SensiFAST SYBR Hi-ROX Master Mix (Bioline, Taunton, MA); the volume was adjusted with nuclease-free water to 10 μL. The real-time PCR program was 95 °C for 2 min followed by 40 cycles at 95 °C for 5 s and 60 °C for 30 s. Real-time PCR assays were performed using an Applied Biosystems ABI 7300 real-time PCR Thermocycler (Applied Biosystems) according to the manufacturer’s recommendations. For RT-qPCR, two technical replicates for each of the eight synthetized cDNAs were performed, with negative controls in each run. The qPCR results were analyzed using the Pfaffl equation [39] of the comparative Ct method (2^-ΔΔCt^). A Pearson product-moment correlation test was performed to evaluate the correlation between the RNA-seq and the RT-qPCR results.

## Results

All infected plants showed aerial symptoms such as chlorosis and wilting by the end of the experiment (7 wpi), whereas these symptoms did not develop in non-infected plants. All plants were tested at 7 wpi for the presence of Lso in leaves by PCR and for ZC symptoms in tubers by frying. DNA was extracted from the mid-vein of a leaf from each plant and all tubers from each plant were chipped and fried [27]. All plants infested with Lso + insects tested positive for Lso by PCR and showed ZC symptoms in the frying test, whereas plants infested with Lso- insects tested negative for Lso in leaves and tubers had no ZC defects when fried (Fig. 1).

### Transcriptome data

To understand how Lso infection affects plant gene expression for each potato variety, we compared the transcriptomes of plants 3 weeks after infestation with potato psyllids harboring or not harboring ‘*Candidatus* Liberibacter solanacearum’, (Lso + and Lso-, respectively). This time point corresponds to the onset of symptoms in Atlantic, e.g., when the Lso + plants showed slight chlorosis at the base of the upper leaf. These symptoms were not yet visible on most Waneta plants. Plant samples were taken from leaf tissues.

We obtained approximately 20 million reads per sample (Table 1). After quality filtering, reads were mapped to the double haploid *Solanum tuberosum* reference genome DMI3.4 ensembl 19; using Tophat2 in CyVerse. Three biological replicates were sequenced for each of the four treatments: e.g., cultivar (Atlantic and Waneta) by bacterial infection status (Lso + and Lso-). However, one of the Atlantic samples in the Lso + treatment was removed after discovering that it was also infected with *Potato virus S* (PVS). In this study, between 59 to 71% of the reads in each library mapped to the potato genome; at least 9 million reads per sample mapped to the potato genome (Table 1).

**Table 1:** Summary of potato transcriptomic data

Variety	Treatment	Reads	Mapped reads	Mapped reads (%)
Atlantic	Lso+	28,874,601	18,865,812	65.3
Atlantic	Lso+	16,489,980	9,972,190	60.5
Atlantic	Lso-	14,819,808	10,060,020	67.9
Atlantic	Lso-	16,106,431	9,567,021	59.4
Atlantic	Lso-	18,219,259	10,890,012	59.8
Waneta	Lso-	26,727,692	18,435,375	69
Waneta	Lso-	35,950,390	24,050,379	66.9
Waneta	Lso-	29,583,541	21,154,857	71.5
Waneta	Lso+	15,547,755	10,354,955	66.6
Waneta	Lso+	15,214,633	10,649,743	70
Waneta	Lso+	25,997,451	17,814,346	68.5

### Differentially expressed genes

Using Cuffdiff2, the following comparisons were performed: Waneta Lso- versus Waneta Lso+, and Atlantic Lso- versus Atlantic Lso+. The objective was to identify genes that were differentially expressed in plants in response to Lso infection, by variety. A total of 397 differentially expressed genes (DEGs) were identified in the Atlantic comparison (Additional file 1: Table S2 and Figure S1), whereas in the Waneta comparison there were 1027 DEGs (Additional file 1: Table S3 and Figure S1). In order to characterize the plant processes potentially affected by Lso infection, DEGs were classified into MapMan functional plant categories [52] and Gene Onotology (GO) enrichment analyses (http://www.geneontology.org/page/go-enrichment-analysis) were performed on sets of DEGs to identify which GO terms (biological process, molecular function, or cellular component) were over- or under-represented, based on gene set annotations. In both Atlantic and Waneta the DEGs were associated predominantly with photosynthesis, primary and secondary metabolism and biotic and abiotic signal recognition and stress responses. Results are discussed under the headers transcriptomic overview, plant metabolism (including photosynthesis), and plant stress response.

### Transcriptomic overview

**Fig. 2:**
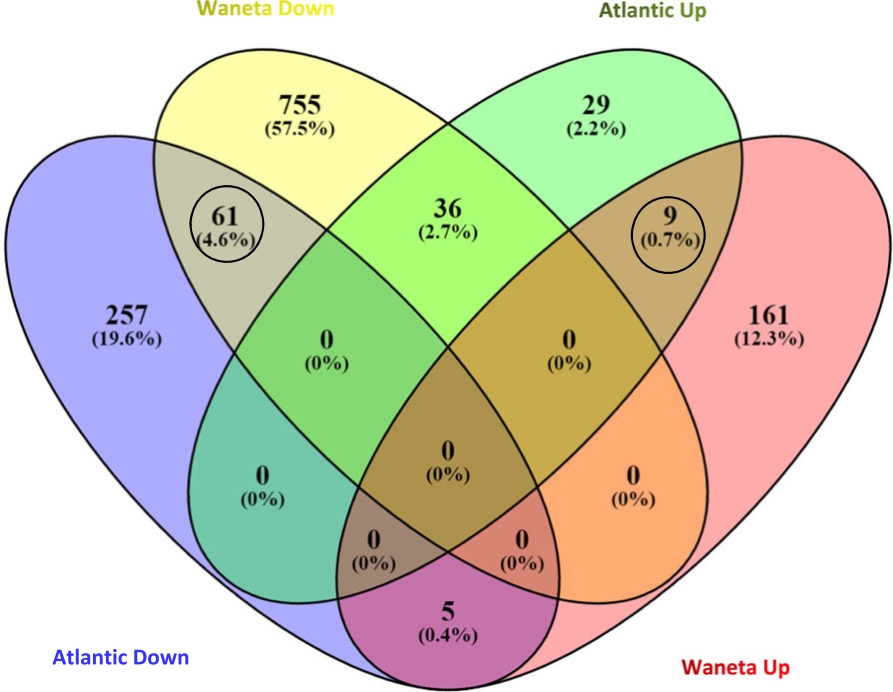
Venn diagram summarizing DEGs in Atlantic (AT) and Waneta(W) in response to Lso infection. Up and Down refer to genes with significantly higher or lower expression in Lso + versus Lso- plants (e.g., up-regulated vs. down-regulated DEGs in response to Lso infection). The number of DEGs and the percentage of the total DEGs are given. DEGs similarly up- or down-regulated in both potato varieties in response to Lso infection are circled

In both varieties, most of the DEGs were down-regulated in the Lso + samples (Atlantic =323 genes, Waneta =852 genes, which corresponds to approximately 82% of the DEGs in each comparison), indicating that the transcriptomic response to Lso infection by both varieties was primarily down-regulation in gene expression. Of the total DEGs, 111 were identified in both the Waneta and Atlantic comparisons: 61 of these conserved DEGs were down-regulated in both comparisons, nine were up-regulated in both comparisons, and 41 were oppositely regulated in the two comparisons (Fig. 2, Additional file 1: Tables S4A-D).

Of the 70 similarly down- or up-regulated DEGs identified in both the Waneta and Atlantic comparisons, GO enrichment analysis showed that GO terms with Molecular Function types associated with photosynthesis, such as GO 0046906 and GO 0016168, were significantly enriched (Additional file 1: Table S5A). Among the 41 Lso-responsive DEGs common to both comparisons but oppositely regulated in Atlantic and Waneta, two GO terms with Molecular Function types were significantly enriched, both associated with transcription factor activity: GO:0001071 and GO:0003700 (Additional file 1: Table S5B).

Of the total DEGs in Atlantic and Waneta, 13% and 21%, respectively, were annotated as conserved genes of unknown function. Since these genes have not yet been characterized, it is possible that they could be associated with specialized functions and may be worthy of future consideration.

### Plant metabolism

In response to Lso infection, a similar number of DEGs involved in plant metabolism were identified in Atlantic and Waneta, although these accounted for a greater percentage of the total number of DEGs identified in Atlantic (110 DEGs = 29% of the total DEGs vs 103 DEGs = 10% of the total DEGs). Furthermore, in Atlantic the DEGs involved in plant metabolism had some of the highest fold changes: 90 of the 110 DEGs were down-regulated and had over a 10-fold decrease in expression in response to Lso infection. GO enrichment analysis indicated that these DEGs were significantly enriched for molecular function terms (e.g., GO:0016798 hydrolase activity, acting on glycosyl bonds; GO:0004553 hydrolase activity, hydrolyzing O-glycosyl compounds; and GO:0003824 catalytic activity). For example, the two DEGs with the highest expression in Atlantic were annotated as the steroidal glycoalkaloid (SGA) pathway enzyme UDP-glucosyl transferase and a peroxidase. The highly expressed DEGS in Atlantic also were significantly enriched for biological process terms associated with photosynthesis and cell wall degradation. In Waneta, the GO enrichment analysis of down-regulated DEGs in response to Lso infection revealed a much wider array of functions (Additional file 1: Table S10).

**Fig. 3:**
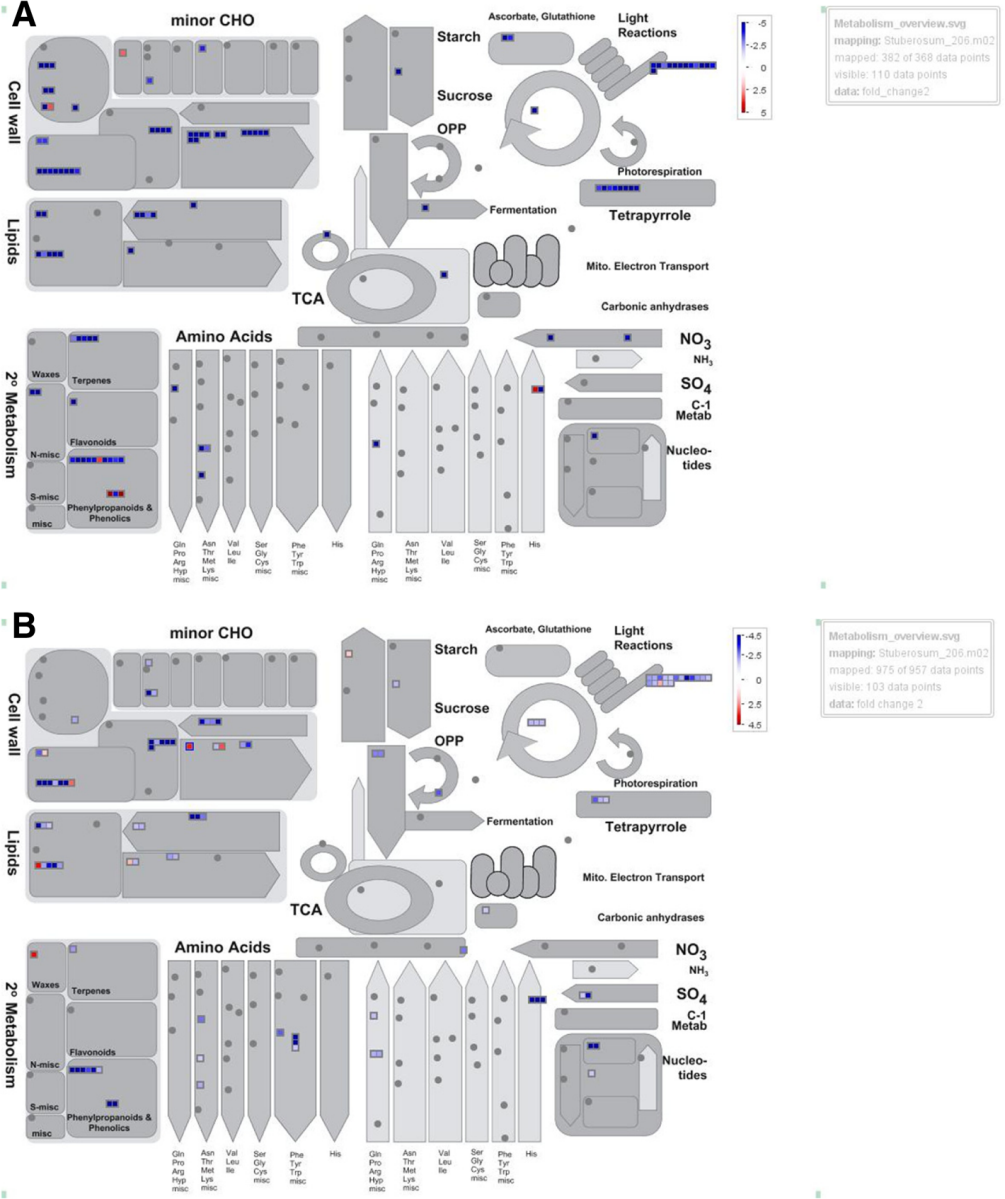
MapMan overview of DEGs annotated as contributing to metabolic pathways in Atlantic (**a**) and Waneta (**b**). Up-regulated DEGs (transcript abundance higher in Lso + samples than Lso- samples) appear in red and down-regulated genes appear in blue. Fold changes are expressed on a logarithmic scale. DEGs in the photosynthesis pathway, carbohydrate metabolism and glycolysis were primarily down-regulated in response to Lso infection in both Atlantic and Waneta

In both the Atlantic and Waneta comparisons, photosynthesis-related DEGs were primarily down-regulated in response to Lso infection. These included genes annotated as functioning in light reactions, tetrapyrrole synthesis, and the Calvin cycle. In particular, several chlorophyll-binding proteins were down regulated in both Waneta and Atlantic. Only one gene in Waneta was up-regulated in those pathways; this gene is annotated as an ATP synthase gamma chain (Fig. 3 and Additional file 1: Table S6).

Similarly, DEGs involved in carbohydrate metabolism and transport were primarily down-regulated in both varieties in response to Lso infection. Interestingly, among them were several genes that typically function in controlling the balance between starch and sucrose (Fig. 3, Additional file 1: Table S7). For example, one gene annotated as UDP glycosyl transferase was down-regulated in Atlantic, whereas an invertase was down-regulated and a transferase was up- regulated in Waneta. Similarly, DEGs annotated as functioning in glycolysis were down-regulated in both varieties. Down-regulated genes in Atlantic included an aldehyde dehydrogenase, a malate synthase and an ATP citrate synthase, and in Waneta included a phosphoglycerate/biphosphoglycerate mutase and a ribose-5-phosphate isomerase. Other DEGs potentially involved in minor carbohydrate metabolism also were primarily down-regulated in both varieties. The one exception was a galactinol synthase that was up-regulated in Atlantic (Additional file 1: Table S7).

In both varieties, Lso infection induced changes in the expression of genes involved in cell wall synthesis and modification, as well as of genes encoding cell wall proteins (Table 2, Fig. 3). Among the 35 DEGs in Atlantic, most DEGs in these categories were down-regulated in response to Lso infection; only one was up-regulated and it encoded a cell wall protein (RGP3). Similarly in Waneta, there were 25 cell wall-related DEGs, the majority of which were down-regulated; only four were up-regulated in response to Lso infection (Table 2).

**Table 2:** Mapman analysis of DEGs related to cell wall synthesis in Atlantic and Waneta in response to Lso infection. Negative Fold Change values denote the fold decrease in transcript abundance (FPKM) in Lso + samples compared to Lso- samples, e.g., down-regulated genes in response to Lso infection; positive Fold Change values denote up-regulated genes. Bin name and descriptors are provided

Bin Name	Transcript id	Fold Change Atlantic	Fold Change Waneta	Gene Description
cell wall.cellulose synthesis	PGSC0003DMT400007050	−28.6	–	Cellulose synthase-like A1
cell wall.cellulose synthesis	PGSC0003DMT400026046	–	−5.78	Transferase, transferring glycosyl groups
cell wall.cellulose synthesis	PGSC0003DMT400068221	−10.36	–	UPA15
cell wall.modification	PGSC0003DMT400003608	−4.43	−2.07	Expansin
cell wall.modification	PGSC0003DMT400022769	−6.02	–	Expansin
cell wall.modification	PGSC0003DMT400025776	−5.35	–	Expansin11
cell wall.modification	PGSC0003DMT400038406	–	−10.23	Xyloglucan endotransglucosylase-hydrolase XTH3
cell wall.modification	PGSC0003DMT400038411	–	−16.06	Xyloglucan endotransglucosylase-hydrolase XTH3
cell wall.modification	PGSC0003DMT400038412	–	−8.31	Xyloglucan endotransglucosylase-hydrolase XTH3
cell wall.modification	PGSC0003DMT400044562	–	−34.42	Xyloglucan endotransglycosylase
cell wall.modification	PGSC0003DMT400047948	–	−5.33	Expansin
cell wall.modification	PGSC0003DMT400055138	−13.58	–	Xyloglucan endotransglucosylase-hydrolase XTH7
cell wall.modification	PGSC0003DMT400061841	−6.14	–	Xyloglucan endotransglucosylase/hydrolase protein A
cell wall.modification	PGSC0003DMT400063689	−49.43	2.82	Xyloglucan endotransglucosylase/hydrolase 1
cell wall.modification	PGSC0003DMT400067358	−3.78	–	Xyloglucan endotransglycosylase/hydrolase 16 protein
cell wall.modification	PGSC0003DMT400078182	−14.74	–	Expansin18
cell wall.precursor synthesis.AXS	PGSC0003DMT400018431	–	−4.09	UDP-apiose/xylose synthase
cell wall.precursor synthesis.UGD	PGSC0003DMT400003666	–	−2.46	UDP-glucose dehydrogenase 2
cell wall.precursor synthesis.GAE	PGSC0003DMT400020223	–	−19.16	UDP-glucuronate 5-epimerase
cell wall.precursor synthesis.GAE	PGSC0003DMT400029181	–	−2.58	UDP-glucuronate 5-epimerase
cell wall.cellulose synthesis.cellulose synthase	PGSC0003DMT400008183	–	−5.39	Glycosyltransferase, CAZy family GT2
cell wall.cellulose synthesis.cellulose synthase	PGSC0003DMT400008184	–	−14.79	Glycosyltransferase, CAZy family GT2
cell wall.cellulose synthesis.cellulose synthase	PGSC0003DMT400009764	–	−2.2	Cellulose synthase
cell wall.cellulose synthesis.cellulose synthase	PGSC0003DMT400030678	−30.63	−5.39	Cellulose synthase
cell wall.cellulose synthesis.cellulose synthase	PGSC0003DMT400073085	−4.76	–	Cellulose synthase catalytic subunit
cell wall.cellulose synthesis.COBRA	PGSC0003DMT400065840	–	−13.12	Protein COBRA
cell wall.cell wall proteins.AGPs.AGP	PGSC0003DMT400033202	−29.85	–	Fasciclin-like arabinogalactan protein 13
cell wall.cell wall proteins.AGPs.AGP	PGSC0003DMT400044769	−15.53	–	Fasciclin-like arabinogalactan protein 19
cell wall.cell wall proteins.AGPs.AGP	PGSC0003DMT400076660	−30.56	–	Fasciclin-like arabinogalactan protein 10
cell wall.cell wall proteins. Proline rich proteins	PGSC0003DMT400004126	−11.75	−2.09	Proline-rich protein
cell wall.cell wall proteins.LRR	PGSC0003DMT400015439	−7.07	–	Leucine-rich repeat/extensin
cell wall.cell wall proteins.LRR	PGSC0003DMT400043527	−6.26	–	Leucine-rich repeat family protein / extensin family protein
cell wall.cell wall proteins.RGP	PGSC0003DMT400039494	−5.13	–	GRP 2
cell wall.cell wall proteins.RGP	PGSC0003DMT400063316	3.38	–	GRP 2
cell wall.degradation.cellulases and beta −1,4-glucanases	PGSC0003DMT400008105	−8.23	–	Endo-1,4-beta-glucanase
cell wall.degradation.cellulases and beta −1,4-glucanases	PGSC0003DMT400009667	−11.21	–	Endo-1,4-beta-glucanase
cell wall.degradation.cellulases and beta −1,4-glucanases	PGSC0003DMT400012809	−9.03	3.45	Endo-beta-1,4-glucanase
cell wall.degradation.cellulases and beta −1,4-glucanases	PGSC0003DMT400015233	−10.73	–	Hydrolase, hydrolyzing O-glycosyl compounds
cell wall.degradation.cellulases and beta −1,4-glucanases	PGSC0003DMT400023082	−4.55	–	Endo-1,4-beta-glucanase
cell wall.degradation.cellulases and beta −1,4-glucanases	PGSC0003DMT400032028	−8.27	–	Endo-beta-1,4-D-glucanase
cell wall.degradation.mannan-xylose-arabinose-fucose	PGSC0003DMT400009055	−19.24	3.03	Endo-beta-mannanase
cell wall.degradation.mannan-xylose-arabinose-fucose	PGSC0003DMT400012716	−6.72	–	Xylanase Xyn2
cell wall.degradation.mannan-xylose-arabinose-fucose	PGSC0003DMT400076470	–	−1.92	LEXYL2 protein
cell wall.degradation.pectate lyases and polygalacturonases	PGSC0003DMT400001638	−4.7	–	Polygalacturonase
cell wall.degradation.pectate lyases and polygalacturonases	PGSC0003DMT400027955	−17.69	–	Pectate lyase
cell wall.degradation.pectate lyases and polygalacturonases	PGSC0003DMT400064881	−24.45	–	Dehydration-responsive protein RD22
cell wall.degradation.pectate lyases and polygalacturonases	PGSC0003DMT400076251	−8.1	−2.32	Pectase lyase
cell wall.degradation.pectate lyases and polygalacturonases	PGSC0003DMT400079602	−32.82	−3.43	Polygalacturonase-1 non-catalytic subunit beta
cell wall.pectin*esterases.PME	PGSC0003DMT400023725	–	−2.94	Pectinesterase 3
cell wall.pectin*esterases.PME	PGSC0003DMT400035577	–	1.71	Pectinesterase
cell wall.pectin*esterases.PME	PGSC0003DMT400016326	−3.55	–	Glutamyl-tRNA reductase
cell wall.pectin*esterases. Acetyl esterase	PGSC0003DMT400062026	−3.61	–	PAE

DEGs annotated as functioning in lipid metabolism also were identified: in Atlantic 13 of these DEGs were identified and all were down-regulated in response to Lso, whereas in Waneta, 15 DEGs were identified and all but two were down-regulated (Fig. 3, Additional file 1: Figure S2 and Table S8). Additionally, 11 Lso-responsive DEGs annotated as being involved in amino acid metabolism were identified in both comparisons, all of which were down-regulated (Fig. 3).

Most of the DEGs involved in secondary metabolism identified in both potato varieties were down-regulated, although some differences in the way genes were regulated were notable. For instance, in Atlantic there were five terpene-related DEGs and all were down-regulated, whereas in Waneta only one terpene-related DEG was identified and it was down-regulated. One flavonoid-related gene, annotated as an isoflavone reductase, was down-regulated in Atlantic whereas none were differentially expressed in Waneta. Nine (out of ten) phenylpropanoid related DEGs were down-regulated in Atlantic, whereas in Waneta all six phenylpropanoid related DEGs were down-regulated. Additionally three DEGs in the phenolic pathway were identified in Atlantic (two were up-regulated) and two in Waneta (both down-regulated) (Fig. 3, Additional file 1: Figure S2 and Table S9).

Interestingly, in Waneta, among the highly up-regulated genes were two genes annotated as encoding patatins (PGSC0003DMG400014104 and PGSC0003DMG402017090); however, the gene encoding patatin 3 (PGSC0003DMG400010022) was down-regulated. Patatins are glycoproteins that serve as the major storage protein in tubers. These proteins also are reported to have esterase/lipase activity [44]. Moreover, genes encoding other tuber-specific proteins such as PGSC0003DMG400007994 and PGSC0003DMG400010296 (Patellin-4) also were differentially regulated in Waneta, but these were down-regulated. The detection of tuber-specific DEGs in response to Lso infection in leaf samples was somewhat unexpected, because the functions of these genes in leaves are unknown.

### Plant stress response

**Fig. 4:**
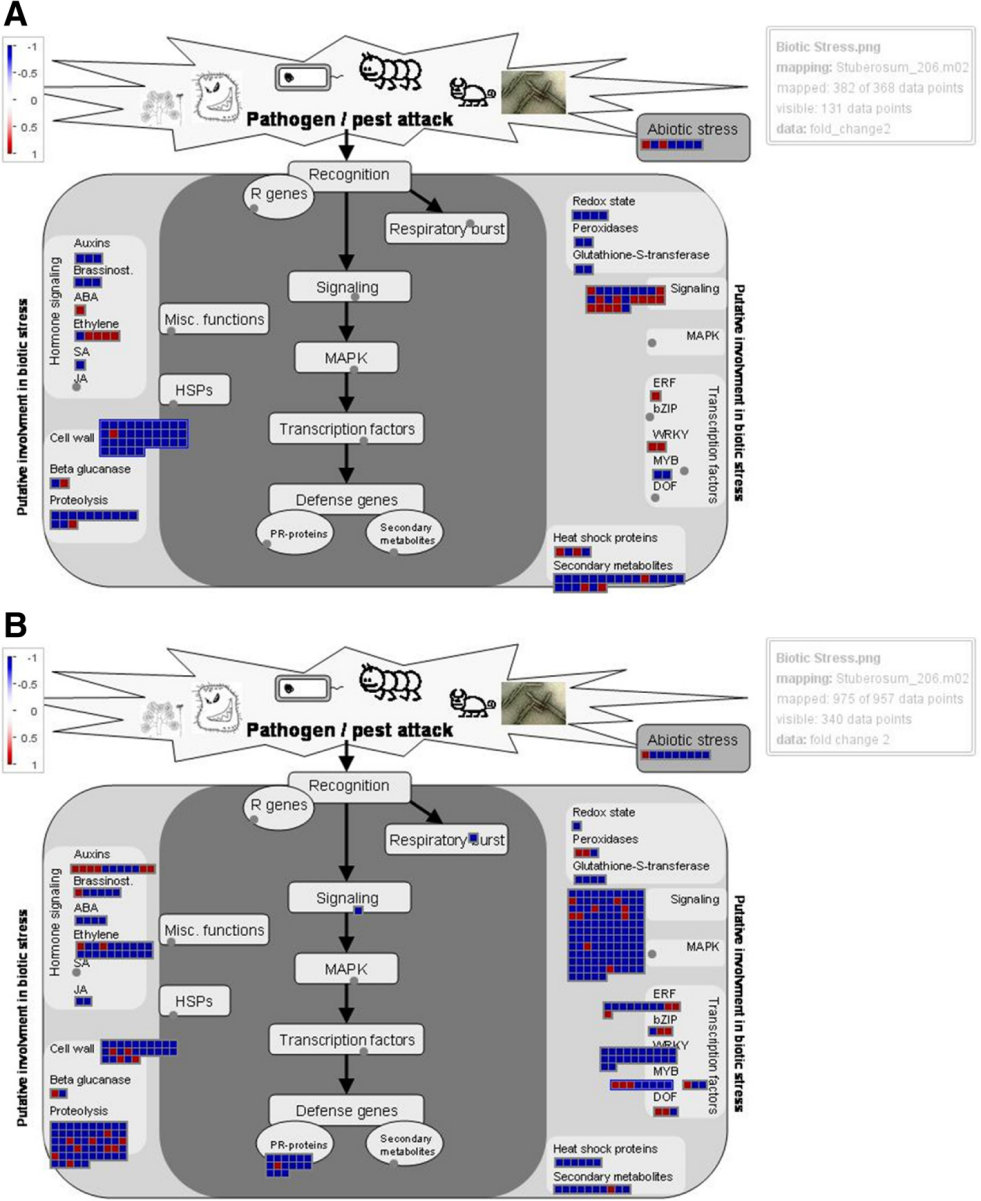
MapMan overview of DEGs annotated as contributing to biotic and abiotic stress-related pathways in Atlantic (**a**) and Waneta (**b**). Up-regulated DEGs (transcript abundance higher in Lso + samples than Lso- samples) appear in red and down-regulated genes appear in blue. Fold changes are expressed on a logarithmic scale

**Fig. 5:**
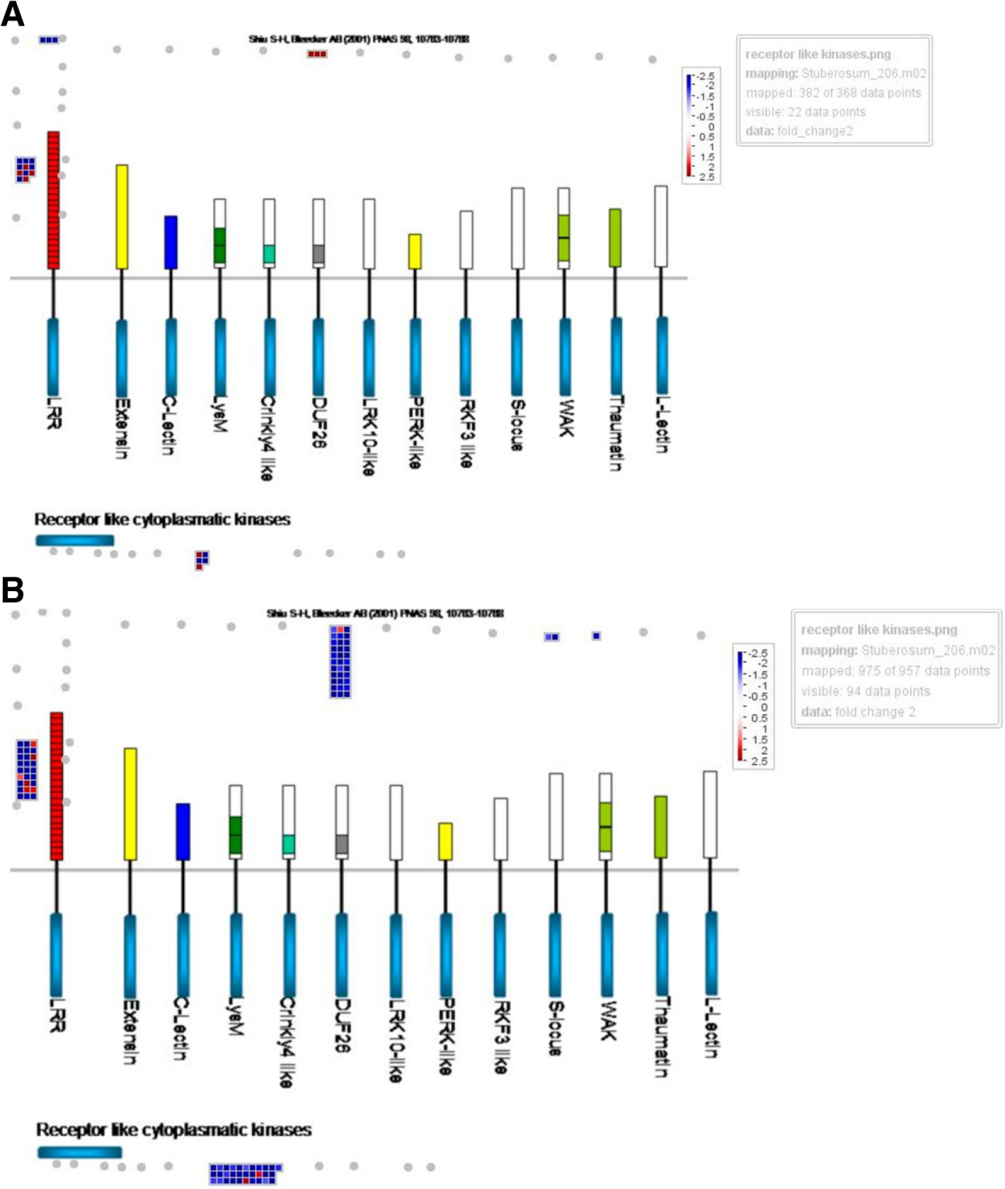
MapMan overview of DEGs annotated as receptor kinases in Atlantic (**a**) and Waneta (**b**). Up-regulated DEGs (transcript abundance higher in Lso + samples than Lso- samples) appear in red and down-regulated genes appear in blue. Fold changes are expressed on a logarithmic scale

As compared to DEGs involved in metabolism where a similar number of DEGs were identified, in both the Atlantic and Waneta comparisons in response to Lso (110 and 103, respectively) almost three times more DEGs involved in stress response were identified in the Waneta compared to Atlantic (340 and 131, respectively). Still, this amounted to about one third of the total DEGs identified in both the Waneta and Atlantic comparisons having a role in biotic/abiotic stress response. In this category, most of the DEGs were annotated as being involved in signaling, hormone pathways, transcription, defense against pathogens, or abiotic stress (Figs. 4 and 5).

In Waneta, 115 signaling-related DEGs were identified, of which only nine were up-regulated in response to Lso infection, whereas in Atlantic there were 22 DEGs, of which 12 were up-regulated (Additional file 1: Table S11). In both varieties, a large number of DEGs were annotated as encoding receptor-like kinases and genes involved in calcium signaling. GO enrichment analysis of Waneta down-regulated genes with high fold change (73 DEGs with more than 10× fold change) showed enrichment in GO terms associated with calcium ion transmembrane transporter activity (GO:0015085); calcium-transporting ATPase activity (GO:0005388); and ATPase activity, coupled to transmembrane movement of ions, phosphorylative mechanism (GO:0015662). MapMan identified a total of 35 DEGs involved in calcium signaling in Waneta and all were down-regulated, whereas the four DEGs involved in calcium signaling in Atlantic were up-regulated (Fig. 4, Additional file 1: Figure S3 and Table S11). For instance, in Waneta, 17 DEGs related to calcium signaling and13 DEGs annotated as calmodulins were identified, whereas in Atlantic only two DEGs involved in calcium binding and two DEGs annotated calmodulin genes were identified.

Similar receptor-like protein kinases were among the DEGs identified in Atlantic and Waneta in response to Lso infection (Fig. 5a, b). Among the Atlantic DEGs, three were annotated as DUF26, three as LRR III and 11 as LRR XI genes. The DEG with the highest difference in expression was the LRR gene, which was down-regulated 12 fold in Lso-infected Atlantic (PGSC0003DMG400013898, Hcr2-0B). This gene is a homolog of the tomato Cf-5 disease resistance gene [11], and LRR is an important gene family involved in plant microbe interactions. Among the DEGs in Waneta, 27 were annotated as DEGLRR XI and 33 as DUF26. Of these seven were up-regulated (Fig. 5b) in response to Lso infection. Among the DEGs in Waneta, one WAK and two S-locus glycoprotein-like genes were down-regulated in response to Lso. No DEGs in these categories were identified in Atlantic. Also in Waneta, 31 DEGS annotated as receptor-like cytoplasmic kinases were down-regulated, whereas in Atlantic, only five DEGs in this category were identified.

Lso-responsive DEGs involved in several hormone pathways were identified in both varieties. Overall, in Waneta, 41 DEGs related to auxin, gibberellic acid, brassinosteroids, cytokinin, JA, and SA synthesis, degradation, and/or signaling were identified. Thirty-two of these were down-regulated, whereas nine were up-regulated in Lso infected plants. The ethylene and auxin pathways were the hormone pathways with the highest number of DEGs (19 for ethylene, only two of which were up-regulated, and 15 for auxin, of which four were up-regulated). Additionally in Waneta, six DEGs involved in brassinosteroid signaling and two DEGs in jasmonic acid signaling were identified, whereas in Atlantic, there were 12 DEGs in these categories (Additional file 1: Figure S3 and Table S12).

Interestingly, although many hormonal pathways were affected by Lso infection in both potato varieties, the responses often were opposite. For example, in Waneta, Lso infection resulted in down-regulation in the expression of genes involved in abscisic acid (ABA) signaling, such as genes annotated as encoding GRAM domain-containing protein / ABA-responsive protein-related, and in genes involved in ethylene signaling such as *ERF-1,2,3,4,5,9.* However, in Atlantic, the expression of genes involved in these hormone signaling pathways were up-regulated (Fig. 4). Similarly, in Atlantic, a DEG involved in the salicylic acid (SA) synthesis (S-adenosyl-L-methionine:benzoic acid carboxyl methyltransferase) was down-regulated in response to Lso, whereas in Waneta, two LOX genes, involved in jasmonic acid (JA) synthesis were down-regulated. In addition to the genes recognized by MapMan in each hormonal signaling pathway, 13 down-regulated and five up-regulated DEGs in gibberellic acid signaling and five DEGs in cytokinin signaling in Waneta were identified. The gibberellic acid signaling pathway has been implicated in plant development and plant-microbe interactions [15, 16].

The majority of DEGs annotated as transcription factors in Waneta (125) were down-regulated, whereas there were only 25 such DEGs in Atlantic (Fig. 4, Additional file 1: Table S13). The most represented family among the differentially expressed transcription factors was the WRKY family, with 22 down-regulated genes in Waneta and two up-regulated genes in Atlantic. In Waneta, 11 DEGS related to ethylene signaling in the AP2 EREBP family were identified, eight were down-regulated, whereas in Atlantic only one DEG from this family was identified. Similarly, 14 DEGs annotated as GRAS genes were found in Waneta (11 were down-regulated), whereas no such DEGs found in Atlantic. GRAS genes are gibberellic acid-dependent transcriptional regulators.

GO enrichment analysis of the up-regulated DEGs in Waneta in response to Lso infection indicated they were enriched in terms related to nucleic acid binding transcription factor activity(GO:0001071), transcription factor activity, and sequence-specific DNA binding (GO:0003700). The same GO terms were enriched among the 74 up-regulated DEGs in Atlantic: however, it was interesting that these were not the same genes as those that were differentially expressed in Waneta.

Additionally, in Waneta, over 100 DEGs annotated as being associated with plant defense mechanisms were identified. Among these, 18 genes annotated as Avr9/Crf9 elicited proteins were down-regulated in Lso-infected plants, but one was up-regulated; five of these genes were also differentially expressed in Atlantic. Several Lso-responsive DEGs involved in proteolysis were identified in both Waneta and Atlantic: 55 vs 13 genes, respectively. Some of these DEGs were annotated as encoding F-box family proteins and ubiquitin-protein ligases. Such proteins have been shown to be vital to plant stress response. Similarly, 14 pathogenesis-related (PR) genes (most of them belonging to the TIR-NBS-LRR class) were down-regulated in Waneta in response to Lso infection, whereas only one PR gene was up-regulated. None of the genes encoding these putative disease resistance proteins was differentially expressed in Atlantic.

Several stress-associated Lso-responsive DEGs were identified. In Waneta, several genes encoding salt-responsive proteins were down-regulated, including the genes with the two highest fold changes (126× and 86×, respectively, encoding salt responsive protein 2 genes PGSC0003DMG400000332 and PGSC0003DMG400010713, as well as a stress associated protein 11 gene PGSC0003DMG400000512. A salt responsive protein 1 gene (PGSC0003DMG400022888) was down-regulated in both Waneta and Atlantic in response to Lso infection (2.85× and 2.97×, respectively). Moreover, a gene annotated as encoding a salt overly sensitive protein (PGSC0003DMG400010630) was highly induced (5.42×) in response to Lso infection in Atlantic.

### Quantitative RT-PCR validation

The expression of 15 genes in Atlantic and 14 genes in Waneta was verified by RT-qPCR, using Ef1α as the reference gene. Ten of these genes were differentially expressed in response to Lso infection in both Atlantic and Waneta, and the remainder of the genes were differentially expressed in only one of the cultivars. In each case, similar gene expression patterns were observed using both the RT-qPCR and the transcriptomic analyses (Table 3). The relative gene expression levels obtained by the RNA-seq and RT-qPCR methods were compared using RPKM and ∆Ct values for each variety. The Pearson product-moment correlation test showed a correlation between the result from the RNA-seq and the RT-qPCR methods in Atlantic (*r* = 0.659365899, *n* = 30, *P* = 6.41221E-05) and in Waneta (*r* = 0.724606906, *n* = 28, *P* = 1.03377E-05) with a significant *P* value. Therefore, the RT-qPCR data support the transcriptomic analyses presented above.

**Table 3:** Relative gene expression determined by real time RT-qPCR (2^-∆∆Ct^). Gene expression was normalized to the expression of Ef1α and is presented as mean value of the Lso + treated plants compared to the Lso- treated plants for each variety

GENE ID	Gene function	Mean 2^-△△Ct^ value in Atlantic	Mean 2^-△△Ct^ value in Waneta
Genes down-regulated in Atlantic and Waneta after Lso treatment			
PGSC0003DMG400012822	Stem-specific protein TSJT1	0.0291977	0.1709327
PGSC0003DMG400004301	Chlorophyll a,b binding protein type I	0.0536232	0.0844505
PGSC0003DMG400033084	Chlorophyll a/b-binding protein (cab-12)	0.1027244	0.0373299
PGSC0003DMG400009869	DNAJ	0.2373127	0.0143763
PGSC0003DMG400011751	2-oxoglutarate-dependent dioxygenase	0.02778	0.134926
PGSC0003DMG400012763	C-4 sterol methyl oxidase	0.0117969	0.0495508
Genes up-regulated in Atlantic and down-regulated in Waneta after Lso treatment			
PGSC0003DMG400006179	Nodulin family protein	9.564544	0.1504254
PGSC0003DMG400036566	Ethylene response factor 5	2.2506224	0.1328974
PGSC0003DMG400031457	Phenylalanine ammonia-lyase 1	11.298391	0.036989
PGSC0003DMG402007970	Conserved gene unknown function	30.013692	2.2496471
Genes regulated in Atlantic			
PGSC0003DMG400011740	SGA	0.0212225	
PGSC0003DMG400027453	Ribonuclease t2	0.1067257	
PGSC0003DMG400009513	Aspartic protease inhibitor 5	0.1006475	
PGSC0003DMG400032792	Calmodulin-binding protein	13.664482	
PGSC0003DMG400011633	WRKY-type transcription factor	4.8602585	
Genes regulated in Waneta			
PGSC0003DMG400000332	Salt responsive protein 2		0.0393988
PGSC0003DMG400010713	Salt responsive protein 2		0.0777253
PGSC0003DMG400019964	Conserved gene unknown function		0.3503861
PGSC0003DMG400025721	Conserved gene f unknown function		7.2729239

## Discussion

Zebra Chip disease is spreading quickly throughout the potato growing regions of the Americas and the world, without the availability of disease resistance in market varieties. In this study, next-generation transcriptomic sequencing was used to identify potato genes that are differentially expressed in response to infection by Lso. The design of the experiment compared Lso-infected and uninfected plants of two different potato varieties, the ZC sensitive Atlantic and the more ZC tolerant Waneta. Transcriptome-wide expression of potato genes in response to Lso infection at 3 wpi was determined separately for each variety, and differences between Atlantic and Waneta in transcriptomic responses are discussed. A total of 397 genes was differentially expressed in response to Lso infection in Atlantic as compared to 1027 genes in Waneta. Significantly, over 80% of the DEGs were down-regulated in both potato varieties. Although more DEGs were identified in Waneta than in Atlantic, it must be noted that one of the Atlantic Lso + samples was discarded and only two biological replicates were used in the Atlantic comparison. Thus, it is possible that the smaller number of replicates could have contributed to the identification of fewer DEGs.

In both varieties, primary and secondary metabolism were strongly altered by Lso infection: e.g., 1/3 of the DEGs identified in Atlantic and 1/10 of the DEGs in Waneta. In both varieties, many of the down-regulated DEGs were related to photosynthetic functions. The down-regulation of DEGs associated with photosynthesis in Atlantic was not surprising since yellow chlorosis on some of the upper leaves of the plants was observed by 3 wpi. Down-regulation of DEGs related to photosynthesis also were observed in Waneta although fewer changes in the expression of genes involved in light reaction and tetrapyrrole metabolism were detected, as compared to Atlantic. This probably reflects the 1–2 week delay in symptom development we observed in this study and reported previously [26]. These results suggest that although chlorosis was just beginning in Atlantic and not yet observed in Waneta plants at sampling 3 wpi, transcriptomic changes that would lead to the development of this symptom already were occurring.

Because carbohydrate metabolism and transport in plants are closely linked with photosynthesis regulation, reductions in these functions also were expected in Lso-infected plants. Changes in sugar transport and sucrose and starch metabolism following Lso infection have been reported previously in both the stems and tubers of potato plants [3, 54]. In these studies, the changes were linked to the development of ZC symptoms such as chlorosis in the leaves, the formation of aerial tubers, and the darkened medullary rays in the tubers particularly visible upon frying. Previously, it was suggested that Lso infected stems of potato plants may be reprogrammed to exhibit tuber-like physiological properties, including the accumulation of tuber storage proteins such as patatins [3]. In *Arabidopsis thaliana* members of the patatin-related family were found to be expressed in several different tissues [21] and potentially function in plant signal transduction as phospholipase A in response to auxin and pathogens [50]. The present study revealed that significant up-regulation in the expression of two of the patatin genes in the aerial tissues occurred by 3 wpi in Waneta, although the reason for this differential gene expression has yet to be determined.

Other metabolic pathways altered in response to Lso were cell wall synthesis and metabolism. Changes in cell wall metabolism associated with pathogen infection are common because the plant cell wall is the first barrier pathogens encounter following dispersal to plant surfaces. In contrast, Lso is inoculated into the sieve elements by psyllids and appears to be limited by the plant to phloem tissues. The plant cell wall is composed of carbohydrates, complex phenolic polymers, and structural proteins including receptors, which can modify the cell wall structure [49]. Changes in cell wall structure and composition in response to Lso infection could affect tissue structure and nutrient exchange between sink and source tissues. Therefore, the down-regulation of genes involved in cell wall metabolism or encoding cell wall structural proteins or other components could be an important element in ZC symptom development, including changes in cell growth, stunting of plant growth, and potentially development of aerial tubers. Previously, increased levels of total phenolic compounds or activity of polyphenol oxidase enzymes were reported in Lso-infected stems [3] and tubers [54]. It is interesting that we find these pathways up-regulated at the transcription level by 3 wpi, coincident with the onset of symptoms.

Genes involved in pathogen recognition, signaling, and defenses were differentially expressed in response to Lso infection in both varieties. Although more DEGs involved in stress responses were identified in Waneta than in Atlantic, in both varieties this category comprised ~1/3 of the DEGs identified. Receptor-like protein kinases are involved in many signaling pathways, but some of them, such as the LRR family, are involved in early steps of plant recognition of pathogenic signatures such as flagellin or lipopolysaccharides and defense. Although the transient expression of a conserved 22 AA-peptide of Lso flagellin did not induce cell death or Reactive Oxygen Species (ROS) in potato plants [20], it is possible that LRR mediates the interaction between Lso and plants through other bacterial signals. The expression levels of several LRR genes were either up- or down-regulated in each of the varieties. Other DEGs belonged to the DUF26 receptor kinase family, which is involved in oxidative stress and hormone and plant defenses signaling. Although the specific function of each protein in these families has not been determined in potato, the regulation of plant signaling mechanisms induced in recognition of and defense against Lso may be useful for controlling Lso infection in the future.

Effector Triggered Immunity (ETI) is a defense mechanism that is activated in response to specific pathogen effectors. In response to Lso infection, the expression of Avr9/Cf-9 genes were down-regulated. Avr9/Cf-9 function in the initial development of the defense response upon perception of pathogen molecules and are known to be up-regulated 15 to 30 min after infection [45]. The activation of ETI triggers a cascade of responses including calcium, MAPK and oxidative burst. Three percent of the DEGs in Waneta were related to calcium signaling. Calcium is involved in plant-microbe signaling in both symbiotic [24] and pathogenic interactions [41]. The down-regulation of signaling genes, ROS related enzymes, and Avr9/Cf-9 genes may indicate that Lso can efficiently repress plant defenses as suggested previously [8].

Following the perception of the stressor and signaling, plants regulate the expression of defenses. Many of the same phytohormone pathways were differentially regulated in Atlantic and Waneta, but not always in the same direction. The auxin and ethylene were the main phytohormone-signaling pathways similarly altered in response to Lso infection. Auxins are involved in regulation of the legume-rhizoba interactions [23], which is interesting given Lso as a member of the Rhizobiaceae is somewhat taxonomically related to rhizobia. The main differences between varieties were in the differential expression of genes related to the cytokinin and jasmonate pathways in Waneta, but not Atlantic; and the differential expression of genes in the salicylic acid pathway in Atlantic, but not Waneta. Hormones and the crosstalk between hormone signaling pathways play a critical role in plant perception and response to pathogen infection. Our results suggest that differences in susceptibility (apparent as rate of symptom development) between Atlantic and Waneta may be related to the observed differences in the regulation of some of the phytohormone pathways.

Overall, there were 41 DEGs that were regulated in the opposite direction in the two potato varieties. In addition to differences in phytohormone pathways, GO enrichment analysis identified enrichment in transcription factor terms among these genes (Additional file 1: Table S5B). The enrichment in DEGs that may be transcription factors in both Waneta and Atlantic is consistent with the extensive reprogramming of gene expression that was observed in plants following Lso infection.

In this study, plant responses to Lso infection were identified in two potato varieties with different degrees of susceptibility to ZC, and key differences in phytohormone signaling between both varieties were uncovered. However, it is important to note that in spite of the differences between Atlantic and Waneta in their responses to pathogen infection observed, both varieties are susceptible to Lso infection and ZC disease development. In both varieties, Lso populations grow slowly, but can be reliably detected by PCR 3 weeks after infection, with no difference in bacterial titer [26]. The main difference between the varieties is the rate of symptom development, which typically results in a one to several week delay in the onset of symptoms and the time to plant death in Waneta relative to Atlantic [26]. The present study was performed at three wpi in order to capture the transcriptomic differences between varieties that may be contributing to the rate of disease symptom development. Although earlier time points may reveal the initial pathogen recognition responses, 3 weeks after infection is a key time point in the development of Zebra Chip disease symptoms and thus in identifying the underlying causes.

Even though the specific Ca. Liberibacter virulence mechanisms triggering plant disease development are still somewhat unclear, some progress has been made in identifying pathogenicity and virulence traits (reviewed in [55]. Currently, it is unclear which factors play the most important role in the development of disease symptoms, i.e., whether symptoms are due primarily to the metabolic activity or virulence factors produced by the pathogen, the host plant responses to these, or some combination. Interestingly, recent findings suggest that disease symptoms in specific organs such as tubers may develop independently from the physiological changes in photosynthesis, nutrient transport, or metabolism occurring in the aerial plant organs. For example, one study revealed that plants infested with psyllids only 48 h before vine kill still developed symptoms in tubers left to mature on the ground for 1 month [46]. Moreover, tubers of plants infected just 4 days before harvest became infected despite the plants and tubers being asymptomatic and testing negative for Lso at harvest. Collectively, these findings demonstrate that despite the importance of understanding how whole plant physiology affects disease development, there is still much to be learned about the organ specific response to Lso infection.

This study is the first to observe the transcriptomic consequences of Lso infection in potato, and was focused on the time of symptom development and the responses of potato varieties differing in the rate of symptom development. The results of our study complement the few previous transcriptomic analyses of *Liberibacter*-citrus interactions which focused on citrus tree response to infection resulting in huanglongbing (citrus greening) disease [5, 29, 30, 57] or Lso-psyllid vector interactions which centered on the insect’s transcriptomic response to infection and/or the bacterial transcriptome in the insect [14, 22, 35, 56].

## Conclusion

This study provides insights into the complex network of changes that occur in potato plants following Lso infection at a timepoint coincident with the onset of symptom development. Those changes were characterized by a preponderance of genes being down-regulated. We identified multiple pathways that are responsive to Lso infection. Our analysis suggests that prior to disease symptom development, dramatic changes in transcriptome-wide gene expression have already occurred in the plant host that underly the physiological changes leading to symptom development. Substantial reprogramming of both primary and secondary plant metabolism were revealed, especially the down-regulation in the expression of genes related to photosynthesis and in the expression of genes involved in phytohormone regulation. The results support the hypothesis that Lso can repress plant physiology and metabolism as well as signaling and defense mechanisms leading to disease development.

## Additional file

Additional file 1: Table S1. Primers for qPCR.** Table S2**. DEGs in Atlantic. **Table S3**. DEGs in Waneta. **Table S4**. Common DEGs in Waneta and Atlantic. (A) 61 genes down regulated after Lso treatment in both varieties. (B) 9 genes up regulated after Lso treatment. (C) 5 genes up regulated in Waneta and down regulated in Atlantic. (D) 36 genes down regulated in Waneta and up regulated in Atlantic. **Table S5**. Gprofiler results for Go enrichment in the genes commonly regulated. (A) Go Term enrichment for the 70 genes regulated in the same direction. T-type BP: Biological Process, CC. (B) Go Term enrichment for the 41 genes regulated in opposite direction. **Table S6**. Photosynthesis-related DEGs in Waneta and Atlantic (mapman analysis). **Table S7**. List of carbohydrates metabolism DEGs in Waneta and Atlantic (mapman analysis). **Table S8**. List of lipid metabolism DEGs in Waneta and Atlantic (mapman analysis). **Table S9**. List of secondary metabolism DEGs in Waneta and Atlantic (mapman analysis). **Table S10**. Gprofiler analysis of genes down regulated in AT (A) and in Waneta (B). **Table S11**. Signaling-related DEGs in Waneta and Atlantic (mapman analysis). **Table S12**. Hormone-related DEGs in Waneta and Atlantic (mapman analysis). **Table S13**. DEGs Transcription factors in Waneta and Atlantic (mapman analysis). **Figure S1**. Volcano plot of differentially expressed genes between control (Lso-) and Lso-infected (Lso+) plants for A) Atlantic and B) Waneta. Volcano plots were obtained from cuffdiff2 output. **Figure S2**. Heat map showing the expression pattern of DEGs between the biological treatment Lso- and Lso + for the DEGs annotated as contributing to metabolic pathways in Mapman in Atlantic (left panel) and Waneta (right panel). **Figure S3**. MapMan overview of DEGs annotated as contributing to Regulation overview in Atlantic (A) and Waneta (B). (DOCX 588 kb)

## Abbreviations

DEGs: Differentially expressed genes
Lso: Candidatus Liberibacter solanacearum
ZC: Zebra Chip
