# Supplementary material for: Global gene expression in two potato cultivars in response to ‘Candidatus Liberibacter solanacearum’ infection
Source: BMC Genomics. 2017 Dec 11;18:960. doi: 10.1186/s12864-017-4313-2 (PMC5725879; doi:10.1186/s12864-017-4313-2)
Supplement: Additional file 1: Table S1. — Primers for qPCR. Table S2. DEGs in Atlantic. Table S3. DEGs in Waneta. Table S4. Common DEGs in Waneta and Atlantic. (A) 61 genes down regulated after Lso treatment in both varieties. (B) 9 genes up regulated after Lso treatment. (C) 5 genes up regulated in Waneta and down regulated in Atlantic. (D) 36 genes down regulated in Waneta and up regulated in Atlantic. Table S5. Gprofiler results for Go enrichment in the genes commonly regulated. (A) Go Term enrichment for the 70 genes regulated in the same direction. T-type BP: Biological Process, CC. (B) Go Term enrichment for the 41 genes regulated in opposite direction. Table S6. Photosynthesis-related DEGs in Waneta and Atlantic (mapman analysis). Table S7. List of carbohydrates metabolism DEGs in Waneta and Atlantic (mapman analysis). Table S8. List of lipid metabolism DEGs in Waneta and Atlantic (mapman analysis). Table S9. List of secondary metabolism DEGs in Waneta and Atlantic (mapman analysis). Table S10. Gprofiler analysis of genes down regulated in AT (A) and in Waneta (B). Table S11. Signaling-related DEGs in Waneta and Atlantic (mapman analysis). Table S12. Hormone-related DEGs in Waneta and Atlantic (mapman analysis). Table S13. DEGs Transcription factors in Waneta and Atlantic (mapman analysis). Figure S1. Volcano plot of differentially expressed genes between control (Lso-) and Lso-infected (Lso+) plants for A) Atlantic and B) Waneta. Volcano plots were obtained from cuffdiff2 output. Figure S2. Heat map showing the expression pattern of DEGs between the biological treatment Lso- and Lso + for the DEGs annotated as contributing to metabolic pathways in Mapman in Atlantic (left panel) and Waneta (right panel). Figure S3. MapMan overview of DEGs annotated as contributing to Regulation overview in Atlantic (A) and Waneta (B). (DOCX 588 kb) [file 12864_2017_4313_MOESM1_ESM.docx]

**Supplemetal Table 1.** Primers were designed and ordered from idtDNA ([www.idtDNA.com](http://www.idtDNA.com)).

| Primer name | transcript | gene ID | Sequence: |
| --- | --- | --- | --- |
| 33379-FW | PGSC0003DMT400033379 | PGSC0003DMG400012822 | GCCCATCAAGTCCTCAAAGA |
| 33379-RV |  |  | AGGGCAGCAAAGACTGTATC |
| 10985-FW | PGSC0003DMT400010985 | PGSC0003DMG400004301 | GGGCTTTGTGGAAGGATACA |
| 10985 -RV |  |  | TCATCAGCAAGGCCAAGAG |
| 83158-FW | PGSC0003DMT400083158 | PGSC0003DMG400033084 | CTCAGTGGATCATCCGGTAAAC |
| 83158-RV |  |  | GGAAGCCATTCACCTTTCTTTG |
| 86995-FW | PGSC0003DMT400086995 | PGSC0003DMG400036566 | CGGTGAACTCATCAGGCAATA |
| 86995-RV |  |  | GTGCCATCGGAATGACTCTT |
| 33236-FW | PGSC0003DMT400033236 | PGSC0003DMG400012763 | AACTGACAAGGGATTCCGATAC |
| 33236-RV |  |  | AGAGGTGCTGATCCATTAACC |
| 30676-FW | PGSC0003DMT400030676 | PGSC0003DMG400011751 | ATTCAGAGGAGCATCGTTACTG |
| 30676-RV |  |  | TCCCTATATCTTGGAGGGTTACT |
| 25543-FW | PGSC0003DMT400025543 | PGSC0003DMG400009869 | GAAACCGATCAGTGTTGGTAGA |
| 25543-RV |  |  | CCGCTCAATTAGGAGAGCTAAA |
| 80765-FW | PGSC0003DMT400080765 | PGSC0003DMG400031457 | GGAGCTTTCGAGGACGAATTA |
| 80765-RV |  |  | GATCCTGTTAGGAATCGAAGGG |
| 15811-FW | PGSC0003DMT400015811 | PGSC0003DMG400006179 | GTGTTCAATGGCTTGTCGTTAG |
| 15811-RV |  |  | GCGGTGTTCATCCATGTAGTA |
| 30650-FW | PGSC0003DMT400030650 | PGSC0003DMG400011740 | TCAACAACCTAACTCCGTTCTC |
| 30650-RV |  |  | CCTTCAATCCAACAGCCATTTC |
| 70621-FW | PGSC0003DMT400070621 | PGSC0003DMG400027453 | AAGTTCTGGCCATCGCTTAG |
| 70621-RV |  |  | GTAGAAGTACTCGTGAGGTGTTG |
| 24601-FW | PGSC0003DMT400024601. | PGSC0003DMG400009513 | CCATAGGGCAAGCAGATAACA |
| 24601-RV |  |  | CTCAGGGCAACGTAGACAAATA |
| 82749-FW | PGSC0003DMT400082749 | PGSC0003DMG400032792 | GTGGAACTGGACCTCGTATTG |
| 82749-RV |  |  | CACCCGAGGCGACAAATTA |
| 30390-FW | PGSC0003DMT400030390 | PGSC0003DMG400011633 | GACAAGGCAACAACAGACAAC |
| 30390-RV |  |  | GCACCGGAGCAGGATTATT |
| 20581-FW | PGSC0003DMT400020581 | PGSC0003DMG402007970 | CAAGCCCTTGGATTAAGCATTAC |
| 20581-RV |  |  | CGAAGTCAGATACGGGAGATTG |
| 55830-FW | PGSC0003DMT400055830 | PGSC0003DMG400021684 | CGTGATTGGTGAAGTGGTTTATG |
| 55830-RV |  |  | GCGTGTAATAGCTGCCTTCT |
| 875-FW | PGSC0003DMT400000875 | PGSC0003DMG400000332 | GTGTCTAGGCGATTTGGGTTAG |
| 875-RV |  |  | CGCCATCAACACACCTCTTAT |
| 27828-FW | PGSC0003DMT400027828 | PGSC0003DMG400010713 | TGGGTGAAAGACAGGTCTAAAG |
| 27828-RV |  |  | AAGTTGCTCTGGTCATCCTAAA |
| 51403FW | PGSC0003DMT400051403 | PGSC0003DMG400019964 | GGAGTTTGTCGAGGAGGATTT |
| 51403-RV |  |  | CACTACTTGATCTGCACCTACC |
| 66070-FW | PGSC0003DMT400066070 | PGSC0003DMG400025721 | TGATTTCGGTGGAGTTGGATTAG |
| 66070-RV |  |  | CGGTTGTAAACGGCTTTGTTC |

**Supplemental Table 2: list of genes differentially regulated in Atlantic.** Genes are sorted by regulation direction down indicate genes up regulated in Lso- treatment whereas up indicate genes up regulated in Lso+ treatment. The genes are also sorted by fold change. Sample 1 At Lso- and sample 2 At Lso+

| **Gene name** | **Fold change** | **direction** | **Total fpkm** | **q-value** | **Gene description** |
| --- | --- | --- | --- | --- | --- |
| SGT3.1 | 107.36 | DOWN | 172.55 | 0.00700526 | SGA |
| PGSC0003DMG400025492 | 78.68 | DOWN | 67.61 | 0.0119372 | Peroxidase |
| PGSC0003DMG400027453 | 78.38 | DOWN | 145.1 | 0.00700526 | Ribonuclease t2 |
| PGSC0003DMG400033084 | 63.23 | DOWN | 58.29 | 0.0194307 | Chlorophyll a/b-binding protein (cab-12) |
| PKPI-A5 | 62.32 | DOWN | 173.29 | 0.00700526 | Aspartic protease inhibitor 5 |
| PGSC0003DMG400011751 | 62.27 | DOWN | 1985.95 | 0.00700526 | 2-oxoglutarate-dependent dioxygenase |
| PGSC0003DMG400012763 | 57.32 | DOWN | 272.22 | 0.00700526 | C-4 sterol methyl oxidase |
| PGSC0003DMG402027210 | 55.36 | DOWN | 62.97 | 0.00700526 | Glucosyltransferase |
| PGSC0003DMG400024755 | 49.43 | DOWN | 224.66 | 0.00700526 | Xyloglucan endotransglucosylase/hydrolase 1 |
| PGSC0003DMG402027687 | 46.56 | DOWN | 37.43 | 0.00700526 | Wound-inducible carboxypeptidase |
| PGSC0003DMG400002880 | 40.68 | DOWN | 351.13 | 0.015972 | Proline-rich protein |
| PGSC0003DMG400002156 | 38.34 | DOWN | 212.98 | 0.00700526 | C-4 sterol methyl oxidase 2 |
| PGSC0003DMG400004789 | 37.64 | DOWN | 16.01 | 0.00700526 | Multicopper oxidase |
| PGSC0003DMG401024975 | 35.63 | DOWN | 69.43 | 0.0194307 | Conserved gene of unknown function |
| PGSC0003DMG400030995 | 32.82 | DOWN | 21.08 | 0.00700526 | Polygalacturonase-1 non-catalytic subunit beta |
| PGSC0003DMG400002689 | 31.03 | DOWN | 11.02 | 0.0230277 | Receptor kinase |
| PGSC0003DMG400011750 | 30.83 | DOWN | 219.39 | 0.00700526 | Cytochrome P-450 |
| PGSC0003DMG400028331 | 30.71 | DOWN | 27.21 | 0.00700526 | Zeatin O-glucosyltransferase |
| PGSC0003DMG400011752 | 30.63 | DOWN | 574.38 | 0.00700526 | Cellulose synthase |
| PGSC0003DMG400029811 | 30.56 | DOWN | 20.79 | 0.0259286 | Fasciclin-like arabinogalactan protein 10 |
| PGSC0003DMG400002880 | 30.44 | DOWN | 207.56 | 0.0194307 | Proline-rich protein |
| PGSC0003DMG400033123 | 30.02 | DOWN | 162.93 | 0.00700526 | Basic 7S globulin 2 small subunit |
| PGSC0003DMG400012749 | 29.85 | DOWN | 40.62 | 0.00700526 | Fasciclin-like arabinogalactan protein 13 |
| PGSC0003DMG400002722 | 28.6 | DOWN | 10.18 | 0.03141 | Cellulose synthase-like A1 |
| PGSC0003DMG400010470 | 28.14 | DOWN | 12.8 | 0.00700526 | Subtilase |
| PGSC0003DMG400012822 | 27.4 | DOWN | 297 | 0.00700526 | Stem-specific protein TSJT1 |
| PGSC0003DMG400006880 | 26.9 | DOWN | 57.53 | 0.00700526 | DRT100 |
| SGT1 | 26.8 | DOWN | 48.73 | 0.00700526 | UDP-galactose:solanidine galactosyltransferase |
| PGSC0003DMG401003179 | 25.19 | DOWN | 32.24 | 0.03141 | Conserved gene of unknown function |
| PGSC0003DMG400008811 | 24.86 | DOWN | 52.28 | 0.015972 | Major latex |
| PGSC0003DMG400024278 | 24.72 | DOWN | 84.21 | 0.00700526 | Phenylacetaldehyde synthase |
| PGSC0003DMG400030819 | 24.55 | DOWN | 54.94 | 0.00700526 | Arabinogalactan-protein |
| PGSC0003DMG400025194 | 24.45 | DOWN | 86.56 | 0.00700526 | Dehydration-responsive protein RD22 |
| PGSC0003DMG400033354 | 22.35 | DOWN | 68.04 | 0.0259286 | Sn-1 protein |
| PGSC0003DMG400001183 | 21.75 | DOWN | 12.19 | 0.0336489 | Glycosyltransferase family GT8 protein |
| PGSC0003DMG400015211 | 20.58 | DOWN | 12.43 | 0.00700526 | Beta-galactosidase |
| PGSC0003DMG402003479 | 19.3 | DOWN | 80.61 | 0.00700526 | Oxidoreductase, 2OG-Fe(II) oxygenase family protein |
| PGSC0003DMG400003521 | 19.24 | DOWN | 65.67 | 0.00700526 | Endo-beta-mannanase |
| PGSC0003DMG400009783 | 18.95 | DOWN | 96.14 | 0.00700526 | Proline-rich cell wall protein |
| PGSC0003DMG401025083 | 18.31 | DOWN | 55.19 | 0.00700526 | Peroxidase 12 |
| PGSC0003DMG400029239 | 18.06 | DOWN | 10.98 | 0.0289348 | Conserved gene of unknown function |
| PGSC0003DMG400010771 | 17.69 | DOWN | 58.64 | 0.00700526 | Pectate lyase |
| PGSC0003DMG400029575 | 17.64 | DOWN | 29.1 | 0.00700526 | Catechol oxidase B, chloroplastic |
| PGSC0003DMG400000208 | 17.42 | DOWN | 311.25 | 0.00700526 | Conserved gene of unknown function |
| PGSC0003DMG400026401 | 17.28 | DOWN | 364.36 | 0.00700526 | Delta(7)-sterol-C5(6)-desaturase |
| PGSC0003DMG400021683 | 17.21 | DOWN | 39.4 | 0.015972 | E3 ubiquitin-protein ligase RMA1H1 |
| PGSC0003DMG400000689 | 17.05 | DOWN | 13.57 | 0.00700526 | Glucan endo-1,3-beta-glucosidase |
| PGSC0003DMG400002414 | 17 | DOWN | 293.22 | 0.00700526 | Sn-2 protein |
| PGSC0003DMG400005580 | 16.8 | DOWN | 39.72 | 0.00700526 | Cytochrome P450 92B1 |
| PGSC0003DMG400029086 | 16.51 | DOWN | 1142.68 | 0.0477632 | Conserved gene of unknown function |
| PGSC0003DMG400016967 | 16.13 | DOWN | 7.45 | 0.00700526 | Conserved gene of unknown function |
| PGSC0003DMG400017135 | 15.88 | DOWN | 18.54 | 0.00700526 | Cytochrome P450 |
| PGSC0003DMG400031183 | 15.77 | DOWN | 16.9 | 0.00700526 | Conserved gene of unknown function |
| PGSC0003DMG400029856 | 15.66 | DOWN | 27.8 | 0.0119372 | N-acylneuraminate-9-phosphatase |
| PGSC0003DMG400017376 | 15.53 | DOWN | 82.43 | 0.00700526 | Fasciclin-like arabinogalactan protein 19 |
| PGSC0003DMG400019506 | 15.17 | DOWN | 41.22 | 0.00700526 | Thioredoxin II |
| PGSC0003DMG400004301 | 14.8 | DOWN | 160.46 | 0.00700526 | Chlorophyll a,b binding protein type I |
| PGSC0003DMG400000045 | 14.79 | DOWN | 82.75 | 0.00700526 | Water channel protein |
| EXPA2 | 14.74 | DOWN | 31.49 | 0.00700526 | Expansin18 |
| PGSC0003DMG400016971 | 14.72 | DOWN | 8.72 | 0.00700526 | Conserved gene of unknown function |
| PGSC0003DMG400022448 | 14.33 | DOWN | 41.75 | 0.015972 | Conserved gene of unknown function |
| PGSC0003DMG400013663 | 14.06 | DOWN | 66.72 | 0.00700526 | 3-hydroxy-3-methylglutaryl-coenzyme A reductase 1 |
| PGSC0003DMG400005409 | 13.91 | DOWN | 111.21 | 0.00700526 | Aspartic proteinase nepenthesin-1 |
| LHCB1-2 | 13.88 | DOWN | 183.42 | 0.00700526 | Chlorophyll a/b binding protein |
| PGSC0003DMG400012126 | 13.62 | DOWN | 13.61 | 0.03141 | MYB transcription factor MYB185 |
| PGSC0003DMG400021398 | 13.58 | DOWN | 207.21 | 0.00700526 | Xyloglucan endotransglucosylase-hydrolase XTH7 |
| PGSC0003DMG400009965 | 13.23 | DOWN | 52.94 | 0.00700526 | Pectinesterase |
| PGSC0003DMG400001320 | 12.87 | DOWN | 11.4 | 0.00700526 | Alpha-tubulin |
| PGSC0003DMG400005960 | 12.56 | DOWN | 16.66 | 0.00700526 | Glucosyltransferase |
| PGSC0003DMG400010221 | 12.38 | DOWN | 65.93 | 0.00700526 | Phospholipase A1 |
| PGSC0003DMG400024763 | 12.25 | DOWN | 22.45 | 0.0119372 | Conserved gene of unknown function |
| PGSC0003DMG400016240 | 12.14 | DOWN | 12.81 | 0.00700526 | Beta-hexosaminidase |
| PGSC0003DMG400006183 | 11.79 | DOWN | 30.3 | 0.00700526 | Aquaporin |
| GPP1 | 11.75 | DOWN | 259 | 0.00700526 | Proline-rich protein |
| EXPA7 | 11.59 | DOWN | 22.64 | 0.00700526 | Expansin9 |
| PGSC0003DMG400005656 | 11.35 | DOWN | 266.68 | 0.00700526 | Beta-mannosidase |
| PGSC0003DMG400003774 | 11.21 | DOWN | 79.12 | 0.0259286 | Endo-1,4-beta-glucanase |
| PGSC0003DMG402010026 | 11.01 | DOWN | 15.25 | 0.0119372 | Geraniol 10-hydroxylase |
| PGSC0003DMG400015194 | 10.91 | DOWN | 23.68 | 0.00700526 | Malate synthase |
| PGSC0003DMG400006461 | 10.83 | DOWN | 17.49 | 0.0194307 | GDSL-like Lipase/Acylhydrolase family protein |
| PGSC0003DMG400025487 | 10.82 | DOWN | 272.45 | 0.0259286 | Isocitrate lyase |
| PGSC0003DMG400028604 | 10.82 | DOWN | 16.65 | 0.015972 | Protein kinase |
| STADC | 10.76 | DOWN | 173.19 | 0.00700526 | Arginine decarboxylase |
| PGSC0003DMG400005943 | 10.73 | DOWN | 20.85 | 0.00700526 | Hydrolase, hydrolyzing O-glycosyl compounds |
| PGSC0003DMG400028406 | 10.61 | DOWN | 58.68 | 0.00700526 | Zinc finger protein |
| PGSC0003DMG400001252 | 10.37 | DOWN | 181.88 | 0.00700526 | Leucine-rich repeat protein |
| PGSC0003DMG400026523 | 10.36 | DOWN | 36.43 | 0.015972 | UPA15 |
| PGSC0003DMG400026280 | 10.18 | DOWN | 22.59 | 0.00700526 | Aromatic amino acid decarboxylase 2 |
| PGSC0003DMG400001743 | 10.13 | DOWN | 19.58 | 0.0411649 | Conserved gene of unknown function |
| PGSC0003DMG400008137 | 9.82 | DOWN | 53.02 | 0.00700526 | Glucosyltransferase |
| PGSC0003DMG400013553 | 9.77 | DOWN | 27.25 | 0.015972 | Conserved gene of unknown function |
| PGSC0003DMG400013198 | 9.73 | DOWN | 4.32 | 0.0259286 | Systemin receptor SR160 |
| PGSC0003DMG400007244 | 9.62 | DOWN | 27.29 | 0.015972 | GDSL-lipase 1 |
| PGSC0003DMG400015156 | 9.62 | DOWN | 18.75 | 0.00700526 | Conserved gene of unknown function |
| PGSC0003DMG400011783 | 9.5 | DOWN | 14.85 | 0.0119372 | Cembratrienol synthase 2a |
| PGSC0003DMG400000432 | 9.42 | DOWN | 90.2 | 0.00700526 | Glucosyltransferase |
| PGSC0003DMG400031790 | 9.31 | DOWN | 12.94 | 0.0194307 | Epoxide hydrolase |
| PGSC0003DMG400004317 | 9.28 | DOWN | 17.04 | 0.0336489 | Stellacyanin |
| PGSC0003DMG400007458 | 9.17 | DOWN | 139.21 | 0.00700526 | RAV |
| PGSC0003DMG400020906 | 9.08 | DOWN | 29.98 | 0.0119372 | Plasma membrane intrinsic protein |
| PGSC0003DMG400025875 | 9.08 | DOWN | 7.98 | 0.03141 | Conserved gene of unknown function |
| PGSC0003DMG400004992 | 9.03 | DOWN | 7.49 | 0.0259286 | Endo-beta-1,4-glucanase |
| PGSC0003DMG402001926 | 9.01 | DOWN | 26.9 | 0.00700526 | LATD/NIP |
| ABC2 | 8.73 | DOWN | 6.66 | 0.0119372 | Pleiotropic drug resistance protein 1 |
| PGSC0003DMG400020968 | 8.69 | DOWN | 62.79 | 0.0119372 | Flavonol 4'-sulfotransferase |
| PGSC0003DMG400009011 | 8.62 | DOWN | 9.23 | 0.0360705 | Conserved gene of unknown function |
| PGSC0003DMG400003691 | 8.58 | DOWN | 14.22 | 0.0388355 | Phenylcoumaran benzylic ether reductase |
| PGSC0003DMG400013485 | 8.55 | DOWN | 16.05 | 0.00700526 | ATP-citrate synthase |
| PGSC0003DMG400003371 | 8.53 | DOWN | 14.11 | 0.0119372 | Arabinogalactan protein 2 |
| PGSC0003DMG400004972 | 8.51 | DOWN | 5.37 | 0.049757 | Receptor kinase |
| PGSC0003DMG400023751 | 8.5 | DOWN | 172.07 | 0.00700526 | Basic blue copper protein |
| PGSC0003DMG400015673 | 8.42 | DOWN | 69.45 | 0.0194307 | Geranylgeranyl pyrophosphate synthase 1 |
| PGSC0003DMG400026586 | 8.33 | DOWN | 18.08 | 0.00700526 | Cytochrome P450 |
| PGSC0003DMG400005884 | 8.32 | DOWN | 126.9 | 0.00700526 | Conserved gene of unknown function |
| PGSC0003DMG400025628 | 8.32 | DOWN | 11.15 | 0.0360705 | Pyridoxal-dependent decarboxylase, C-terminal sheet domain containing protein |
| PGSC0003DMG400005931 | 8.31 | DOWN | 241.76 | 0.00700526 | Sterol delta-7 reductase DWF5 |
| PGSC0003DMG400012288 | 8.27 | DOWN | 45.55 | 0.00700526 | Endo-beta-1,4-D-glucanase |
| PGSC0003DMG401001341 | 8.26 | DOWN | 16.66 | 0.0119372 | Conserved gene of unknown function |
| PGSC0003DMG400003124 | 8.23 | DOWN | 19.1 | 0.0360705 | Endo-1,4-beta-glucanase |
| PGSC0003DMG400006713 | 8.19 | DOWN | 29.49 | 0.00700526 | Cembratrienol synthase 2a |
| PGSC0003DMG400019347 | 8.16 | DOWN | 20.1 | 0.0411649 | Chlorophyllase 2 |
| PGSC0003DMG400030102 | 8.15 | DOWN | 144.47 | 0.0119372 | Conserved gene of unknown function |
| PGSC0003DMG400020564 | 8.14 | DOWN | 32.95 | 0.00700526 | Avr9/Cf-9 rapidly elicited protein 216 |
| PGSC0003DMG400029645 | 8.1 | DOWN | 31.49 | 0.00700526 | Pectase lyase |
| PGSC0003DMG400038332 | 8.05 | DOWN | 15.45 | 0.00700526 | Subtilisin-like protease |
| PGSC0003DMG400006712 | 8.01 | DOWN | 40.79 | 0.00700526 | Neryl diphosphate synthase 1 |
| PGSC0003DMG400013010 | 8 | DOWN | 277.09 | 0.00700526 | 24K germin |
| PGSC0003DMG400007309 | 7.96 | DOWN | 45.64 | 0.00700526 | 3beta-hydroxysteroid-dehydrogenase/decarboxylase isoform 2 |
| PGSC0003DMG400015591 | 7.89 | DOWN | 34.81 | 0.0259286 | Nitrate transporter |
| PGSC0003DMG402026767 | 7.86 | DOWN | 27.79 | 0.0119372 | Delta 1-pyrroline-5-carboxylate synthetase |
| PGSC0003DMG400023382 | 7.84 | DOWN | 22.91 | 0.00700526 | Cytochrome P450 77A2 |
| PGSC0003DMG400028959 | 7.83 | DOWN | 9.29 | 0.0194307 | Multicopper oxidase |
| PGSC0003DMG400033044 | 7.83 | DOWN | 27.23 | 0.0434749 | Gip1 |
| PGSC0003DMG400016063 | 7.82 | DOWN | 57.19 | 0.00700526 | Conserved gene of unknown function |
| PGSC0003DMG400015552 | 7.77 | DOWN | 13.64 | 0.015972 | Beta-ketoacyl-coa synthase family protein |
| PGSC0003DMG400021122 | 7.73 | DOWN | 15.28 | 0.03141 | Conserved gene of unknown function |
| PGSC0003DMG402015792 | 7.65 | DOWN | 21.41 | 0.00700526 | Short-chain dehydrogenase |
| PGSC0003DMG400033126 | 7.63 | DOWN | 28.2 | 0.00700526 | Aspartate kinase |
| PGSC0003DMG400020084 | 7.43 | DOWN | 47.32 | 0.00700526 | Zinc finger protein |
| PGSC0003DMG400002519 | 7.41 | DOWN | 91.32 | 0.00700526 | Zinc finger protein |
| PGSC0003DMG400015005 | 7.41 | DOWN | 35.57 | 0.00700526 | Heavy metal-associated domain containing protein |
| PGSC0003DMG401025689 | 7.35 | DOWN | 44.6 | 0.00700526 | Conserved gene of unknown function |
| PGSC0003DMG400013537 | 7.33 | DOWN | 52.56 | 0.0230277 | Proline-rich protein |
| PGSC0003DMG400042273 | 7.26 | DOWN | 53.14 | 0.00700526 | 4,5-DOPA dioxygenase extradiol |
| PGSC0003DMG400008300 | 7.24 | DOWN | 62.97 | 0.00700526 | Chlorophyll a/b binding protein |
| AN34 | 7.17 | DOWN | 185.48 | 0.00700526 | Annexin p34 |
| PGSC0003DMG400004086 | 7.13 | DOWN | 4.77 | 0.0119372 | Heat shock |
| PGSC0003DMG400006027 | 7.07 | DOWN | 11.77 | 0.015972 | Leucine-rich repeat/extensin |
| PGSC0003DMG400018238 | 7.02 | DOWN | 81.93 | 0.00700526 | Hydrolase |
| PGSC0003DMG400028650 | 6.96 | DOWN | 80.15 | 0.0194307 | Fatty acid desaturase |
| PGSC0003DMG400027340 | 6.9 | DOWN | 20.11 | 0.00700526 | Cytochrome P450 77A1 |
| PGSC0003DMG400011431 | 6.83 | DOWN | 4.36 | 0.0434749 | Anthranilate phosphoribosyltransferase |
| PGSC0003DMG400012202 | 6.8 | DOWN | 30.32 | 0.00700526 | Cytochrome P450 fatty acid omega-hydroxylase |
| PGSC0003DMG400004955 | 6.72 | DOWN | 6.16 | 0.0230277 | Xylanase Xyn2 |
| PGSC0003DMG400016880 | 6.68 | DOWN | 70.16 | 0.00700526 | Gene of unknown function |
| PGSC0003DMG400020622 | 6.67 | DOWN | 85.33 | 0.00700526 | Elicitor-inducible protein EIG-J7 |
| PGSC0003DMG400031529 | 6.64 | DOWN | 18.51 | 0.03141 | Glycine-rich protein |
| LHCB1-3 | 6.64 | DOWN | 175.21 | 0.00700526 | Chlorophyll a/b binding protein |
| PGSC0003DMG402015689 | 6.59 | DOWN | 60.96 | 0.015972 | Protein RSI-1 |
| PGSC0003DMG400011266 | 6.52 | DOWN | 20.57 | 0.0119372 | Catechol O-methyltransferase |
| PGSC0003DMG400024274 | 6.52 | DOWN | 138.1 | 0.00700526 | Cytochrome P450 monooxygenase |
| PGSC0003DMG400009921 | 6.46 | DOWN | 61.71 | 0.00700526 | Cysteine protease 14 |
| PGSC0003DMG400000757 | 6.43 | DOWN | 14.34 | 0.0336489 | Salicylic acid-binding protein 2 |
| PGSC0003DMG400025752 | 6.42 | DOWN | 41.87 | 0.00700526 | LOB domain-containing protein |
| PGSC0003DMG400004146 | 6.41 | DOWN | 4.77 | 0.0411649 | Receptor protein kinase CLAVATA1 |
| PI | 6.41 | DOWN | 465.54 | 0.00700526 | Defensin P322 |
| PGSC0003DMG400030251 | 6.4 | DOWN | 20.69 | 0.0360705 | Conserved gene of unknown function |
| PGSC0003DMG400020315 | 6.39 | DOWN | 32.43 | 0.00700526 | Glycerol-3-phosphate acyltransferase 6 |
| PGSC0003DMG400029159 | 6.39 | DOWN | 79.65 | 0.00700526 | Conserved gene of unknown function |
| LHCB1-5 | 6.38 | DOWN | 30.09 | 0.0194307 | Chlorophyll a/b binding protein |
| PGSC0003DMG400003123 | 6.36 | DOWN | 1254.16 | 0.0194307 | Fructose-bisphosphate aldolase |
| PGSC0003DMG400004736 | 6.34 | DOWN | 43.85 | 0.0194307 | P-rich protein EIG-I30 |
| PGSC0003DMG400029515 | 6.31 | DOWN | 21.98 | 0.00700526 | Myosin heavy chain, clone |
| PGSC0003DMG400016899 | 6.26 | DOWN | 15.25 | 0.00700526 | Leucine-rich repeat family protein / extensin family protein |
| PGSC0003DMG400023990 | 6.22 | DOWN | 19.63 | 0.00700526 | Conserved gene of unknown function |
| PGSC0003DMG400024060 | 6.14 | DOWN | 22.29 | 0.03141 | Xyloglucan endotransglucosylase/hydrolase protein A |
| PGSC0003DMG400001380 | 6.13 | DOWN | 45.25 | 0.00700526 | Pollen Ole e 1 allergen and extensin |
| PGSC0003DMG400013547 | 6.13 | DOWN | 16.56 | 0.0289348 | Sucrose sythase |
| PGSC0003DMG400000327 | 6.07 | DOWN | 99.54 | 0.00700526 | Ultraviolet-B-repressible protein |
| PGSC0003DMG400015356 | 6.05 | DOWN | 30.52 | 0.0119372 | NADPH:protochlorophyllide oxidoreductase |
| PGSC0003DMG400008299 | 6.04 | DOWN | 102.41 | 0.00700526 | Chlorophyll a/b binding protein |
| PGSC0003DMG400014507 | 6.03 | DOWN | 7.97 | 0.0119372 | BZIP protein |
| EXPA3 | 6.02 | DOWN | 53.26 | 0.0119372 | Expansin |
| PGSC0003DMG400011311 | 6.01 | DOWN | 20.83 | 0.00700526 | Epoxide hydrolase 1 |
| PGSC0003DMG400027013 | 6 | DOWN | 74.51 | 0.00700526 | Tetrapyrrole-binding protein, chloroplast |
| PGSC0003DMG400004342 | 5.97 | DOWN | 60.83 | 0.0230277 | O-methyltransferase |
| PGSC0003DMG400001283 | 5.96 | DOWN | 165.03 | 0.00700526 | O-methyltransferase 3 |
| PGSC0003DMG400000639 | 5.9 | DOWN | 45.23 | 0.00700526 | Delta 9 desaturase |
| PGSC0003DMG401022600 | 5.86 | DOWN | 24.38 | 0.0360705 | MYC1 |
| PGSC0003DMG400031365 | 5.86 | DOWN | 214.01 | 0.0119372 | Phenylalanine ammonia-lyase |
| PGSC0003DMG400025246 | 5.85 | DOWN | 48.31 | 0.0230277 | Histone H3.2 |
| PGSC0003DMG400004698 | 5.82 | DOWN | 55.09 | 0.00700526 | Aldehyde dehydrogenase |
| MAL2 | 5.81 | DOWN | 41.28 | 0.00700526 | Alpha-glucosidase |
| PGSC0003DMG400004291 | 5.61 | DOWN | 23.88 | 0.0119372 | Oligopeptide transporter |
| PGSC0003DMG400008381 | 5.57 | DOWN | 203.66 | 0.00700526 | Arabinogalactan peptide 14 |
| PGSC0003DMG400011317 | 5.56 | DOWN | 41.79 | 0.0477632 | Conserved gene of unknown function |
| PGSC0003DMG400002989 | 5.48 | DOWN | 23.84 | 0.00700526 | Strictosidine synthase |
| PGSC0003DMG400010117 | 5.48 | DOWN | 28.36 | 0.0119372 | HXXXD-type acyl-transferase family protein |
| AC58 | 5.48 | DOWN | 10.91 | 0.0360705 | Actin-58 |
| PGSC0003DMG402014186 | 5.47 | DOWN | 16.91 | 0.0259286 | ATP binding protein |
| PGSC0003DMG400026885 | 5.46 | DOWN | 57.91 | 0.00700526 | GI11736 |
| PGSC0003DMG400008564 | 5.43 | DOWN | 475.45 | 0.00700526 | Chlorophyll a-b binding protein 13, chloroplastic |
| PGSC0003DMG400014459 | 5.43 | DOWN | 374.17 | 0.00700526 | Phosphoethanolamine N-methyltransferase |
| PGSC0003DMG400023458 | 5.43 | DOWN | 113.94 | 0.00700526 | Phenylalanine ammonia-lyase |
| PGSC0003DMG402024557 | 5.41 | DOWN | 31.29 | 0.0119372 | Conserved gene of unknown function |
| PGSC0003DMG400013983 | 5.36 | DOWN | 40.77 | 0.0230277 | Wound/stress protein |
| EXPA1 | 5.35 | DOWN | 62.35 | 0.015972 | Expansin11 |
| PGSC0003DMG400021550 | 5.31 | DOWN | 90.97 | 0.00700526 | Adenosine kinase isoform 1T |
| PGSC0003DMG400010166 | 5.3 | DOWN | 79.56 | 0.00700526 | Anthocyanin permease |
| PGSC0003DMG400009869 | 5.29 | DOWN | 302.5 | 0.00700526 | DNAJ |
| PGSC0003DMG401032203 | 5.29 | DOWN | 121.28 | 0.0259286 | Extensin |
| PGSC0003DMG400029169 | 5.28 | DOWN | 112.04 | 0.0194307 | Conserved gene of unknown function |
| PGSC0003DMG402027501 | 5.27 | DOWN | 50.46 | 0.00700526 | Oligopeptide transporter |
| PGSC0003DMG400028601 | 5.27 | DOWN | 37.3 | 0.00700526 | Alpha-L-fucosidase 2 |
| PGSC0003DMG400010088 | 5.26 | DOWN | 14.2 | 0.0388355 | Glycosyltransferase family GT8 protein |
| PGSC0003DMG400024281 | 5.21 | DOWN | 1293.6 | 0.0454488 | Gamma aminobutyrate transaminase isoform2 |
| PGSC0003DMG400027892 | 5.2 | DOWN | 17.04 | 0.0119372 | Xylem serine proteinase 1 |
| GLN | 5.18 | DOWN | 21.54 | 0.00700526 | Glutamine synthetase |
| PGSC0003DMG400027509 | 5.18 | DOWN | 18.43 | 0.015972 | Protein ZINC INDUCED FACILITATOR-LIKE 1 |
| PGSC0003DMG400010512 | 5.16 | DOWN | 10.24 | 0.0230277 | Conserved gene of unknown function |
| PGSC0003DMG400015269 | 5.13 | DOWN | 15.9 | 0.0230277 | GRP 2 |
| DS2 | 5.11 | DOWN | 211.7 | 0.00700526 | ASR4 |
| PGSC0003DMG400000803 | 5.05 | DOWN | 13.86 | 0.049757 | Cytochrome B561 family protein |
| PGSC0003DMG400018356 | 5.04 | DOWN | 13.32 | 0.0388355 | Conserved gene of unknown function |
| PGSC0003DMG400003537 | 4.98 | DOWN | 48.23 | 0.00700526 | Beta-galactosidase |
| SN1 | 4.97 | DOWN | 782.46 | 0.015972 | Snakin-1 |
| PGSC0003DMG400024286 | 4.94 | DOWN | 12.62 | 0.0360705 | Ca2+ antiporter/cation exchanger |
| PGSC0003DMG400018930 | 4.93 | DOWN | 77.48 | 0.00700526 | Proteinase inhibitor I4, serpin |
| PGSC0003DMG400010025 | 4.89 | DOWN | 20.52 | 0.0194307 | Multicopper oxidase |
| PGSC0003DMG401006333 | 4.87 | DOWN | 40.11 | 0.0336489 | Histone H3.2 |
| PGSC0003DMG400021684 | 4.86 | DOWN | 240.66 | 0.00700526 | E3 ubiquitin-protein ligase RMA1H1 |
| PGSC0003DMG400014200 | 4.85 | DOWN | 36.9 | 0.00700526 | Flotillin-1 |
| PGSC0003DMG400027047 | 4.85 | DOWN | 373.02 | 0.0194307 | UPF0497 membrane protein |
| PGSC0003DMG400003243 | 4.82 | DOWN | 376.46 | 0.0230277 | Gene of unknown function |
| PGSC0003DMG400014250 | 4.82 | DOWN | 12.63 | 0.03141 | Serine carboxypeptidase |
| PGSC0003DMG400000484 | 4.81 | DOWN | 57.24 | 0.00700526 | Triose phosphate/phosphate translocator, non-green plastid, chloroplast |
| PGSC0003DMG400000963 | 4.79 | DOWN | 12.8 | 0.03141 | Ubiquitin-protein ligase |
| PGSC0003DMG400017513 | 4.78 | DOWN | 22.44 | 0.0477632 | Acetylornithine deacetylase |
| PGSC0003DMG400003543 | 4.76 | DOWN | 12.7 | 0.049757 | Serine-threonine protein kinase, plant-type |
| PGSC0003DMG400028426 | 4.76 | DOWN | 8.29 | 0.0289348 | Cellulose synthase catalytic subunit |
| PGSC0003DMG400000124 | 4.74 | DOWN | 32.89 | 0.015972 | RAB11B |
| PGSC0003DMG400021814 | 4.73 | DOWN | 32.27 | 0.0289348 | Conserved gene of unknown function |
| PGSC0003DMG400030172 | 4.72 | DOWN | 73.27 | 0.00700526 | Aspartic proteinase oryzasin-1 |
| PGSC0003DMG400000608 | 4.7 | DOWN | 9.24 | 0.0388355 | Polygalacturonase |
| NIR | 4.69 | DOWN | 72.9 | 0.00700526 | Nitrite reductase |
| PGSC0003DMG400002720 | 4.68 | DOWN | 68.92 | 0.00700526 | Delta14-sterol reductase |
| PGSC0003DMG400028701 | 4.67 | DOWN | 37.91 | 0.015972 | Zinc finger protein |
| PGSC0003DMG401020115 | 4.64 | DOWN | 59.27 | 0.0434749 | Fimbrin |
| PGSC0003DMG400026516 | 4.57 | DOWN | 64.46 | 0.015972 | Conserved gene of unknown function |
| PGSC0003DMG400008938 | 4.55 | DOWN | 11.39 | 0.0477632 | Endo-1,4-beta-glucanase |
| PGSC0003DMG400007787 | 4.54 | DOWN | 77.55 | 0.00700526 | Chlorophyll a-b binding protein 8, chloroplastic |
| PGSC0003DMG400004639 | 4.53 | DOWN | 640.63 | 0.00700526 | Photosystem II 5 kDa protein, chloroplastic |
| PGSC0003DMG400023341 | 4.52 | DOWN | 25.34 | 0.0289348 | AG-motif binding protein-1 |
| PGSC0003DMG400014172 | 4.51 | DOWN | 23.71 | 0.0411649 | Transferase, transferring glycosyl groups |
| PGSC0003DMG400010165 | 4.5 | DOWN | 14.77 | 0.0336489 | Anthocyanin permease |
| PGSC0003DMG400006149 | 4.44 | DOWN | 2578.56 | 0.0411649 | Chlorophyll a-b binding protein 4, chloroplastic |
| EXPA5 | 4.43 | DOWN | 80.61 | 0.03141 | Expansin |
| PGSC0003DMG400010900 | 4.43 | DOWN | 64.5 | 0.0119372 | Cytosolic acetoacetyl-coenzyme A thiolase |
| PGSC0003DMG400027180 | 4.39 | DOWN | 93.18 | 0.0434749 | Anthocyanin 5-aromatic acyltransferase |
| PGSC0003DMG400012024 | 4.36 | DOWN | 17.97 | 0.0454488 | O-methyltransferase |
| PGSC0003DMG400024575 | 4.33 | DOWN | 39.49 | 0.0259286 | Microtubule-associated protein TORTIFOLIA1 |
| PGSC0003DMG400025972 | 4.3 | DOWN | 121.56 | 0.00700526 | 14 kDa proline-rich protein DC2.15 |
| PGSC0003DMG400000724 | 4.28 | DOWN | 13.21 | 0.0289348 | Serine/threonine-protein kinase PBS1 |
| PGSC0003DMG400026540 | 4.27 | DOWN | 204.7 | 0.0360705 | Conserved gene of unknown function |
| PGSC0003DMG400004161 | 4.24 | DOWN | 21.58 | 0.0259286 | Conserved gene of unknown function |
| PGSC0003DMG400007188 | 4.24 | DOWN | 290.46 | 0.0119372 | Desaturase |
| PGSC0003DMG400012100 | 4.18 | DOWN | 892.25 | 0.0454488 | Major latex |
| PGSC0003DMG400024619 | 4.17 | DOWN | 20.49 | 0.0194307 | Conserved gene of unknown function |
| PGSC0003DMG400004158 | 4.16 | DOWN | 37.08 | 0.0119372 | Ca2+ antiporter/cation exchanger |
| PGSC0003DMG400009973 | 4.11 | DOWN | 34.39 | 0.0259286 | Conserved gene of unknown function |
| PGSC0003DMG400003626 | 4.07 | DOWN | 215.36 | 0.00700526 | Lactoylglutathione lyase |
| PGSC0003DMG400028137 | 4.07 | DOWN | 86.77 | 0.0119372 | Fasciclin-like arabinogalactan protein 14 |
| PGSC0003DMG400018332 | 4.06 | DOWN | 15.55 | 0.049757 | DNA binding protein |
| PGSC0003DMG400012931 | 4.05 | DOWN | 10.38 | 0.0434749 | Conserved gene of unknown function |
| PGSC0003DMG402016112 | 4.04 | DOWN | 40.55 | 0.0454488 | Sinapyl alcohol dehydrogenase 2 |
| PGSC0003DMG400013461 | 4.02 | DOWN | 57 | 0.0119372 | Chlorophyll a-b binding protein 3C, chloroplastic |
| PGSC0003DMG400031775 | 4 | DOWN | 82.76 | 0.015972 | ROX1 |
| PGSC0003DMG400017540 | 3.99 | DOWN | 30.64 | 0.0411649 | BHLH071 |
| PGSC0003DMG400013995 | 3.97 | DOWN | 31.64 | 0.015972 | Serine carboxypeptidase III |
| PGSC0003DMG400006448 | 3.94 | DOWN | 266.53 | 0.0194307 | Caffeoyl-CoA O-methyltransferase |
| PGSC0003DMG400032510 | 3.94 | DOWN | 23.89 | 0.03141 | L-ascorbate oxidase |
| PGSC0003DMG400005896 | 3.93 | DOWN | 7.63 | 0.0411649 | Protein kinase |
| PGSC0003DMG400030587 | 3.92 | DOWN | 104.25 | 0.0336489 | Nonspecific lipid-transfer protein |
| PGSC0003DMG400016249 | 3.91 | DOWN | 36.75 | 0.0289348 | Anthranilate N-benzoyltransferase protein |
| PGSC0003DMG400001879 | 3.89 | DOWN | 81.7 | 0.0230277 | Annexin p35 |
| PGSC0003DMG400014862 | 3.87 | DOWN | 19.18 | 0.0336489 | Subtilisin-like protease |
| PGSC0003DMG400002386 | 3.85 | DOWN | 76.27 | 0.00700526 | Conserved gene of unknown function |
| PGSC0003DMG400006176 | 3.82 | DOWN | 52.18 | 0.0119372 | Transcription factor |
| PGSC0003DMG400020742 | 3.82 | DOWN | 201.53 | 0.00700526 | Aquaporin |
| PGSC0003DMG400010821 | 3.8 | DOWN | 89.71 | 0.03141 | Nucleic acid binding protein |
| PGSC0003DMG400027916 | 3.79 | DOWN | 149.52 | 0.015972 | Inositol-3-phosphate synthase |
| PGSC0003DMG400030834 | 3.79 | DOWN | 53.49 | 0.015972 | Thylakoid lumenal 29 kDa protein, chloroplastic |
| PGSC0003DMG400026189 | 3.78 | DOWN | 56.01 | 0.00700526 | Xyloglucan endotransglycosylase/hydrolase 16 protein |
| PGSC0003DMG400027276 | 3.76 | DOWN | 186.23 | 0.0194307 | Mg protoporphyrin IX chelatase |
| PGSC0003DMG400016155 | 3.75 | DOWN | 51.51 | 0.0259286 | 7-transmembrane G-protein-coupled receptor |
| PGSC0003DMG400009399 | 3.71 | DOWN | 67.41 | 0.0194307 | Glutaredoxin-C9 |
| PGSC0003DMG400024748 | 3.67 | DOWN | 169.99 | 0.0434749 | Histone H2A.1 |
| PGSC0003DMG400003724 | 3.64 | DOWN | 30.27 | 0.0119372 | Myosin heavy chain, striated muscle |
| PGSC0003DMG401025767 | 3.64 | DOWN | 93.38 | 0.00700526 | Cinnamyl alcohol dehydrogenase |
| PGSC0003DMG400026224 | 3.63 | DOWN | 53.5 | 0.0477632 | Methionine rich arabinogalactan |
| PGSC0003DMG401024140 | 3.61 | DOWN | 117.45 | 0.0388355 | PAE |
| PGSC0003DMG400016695 | 3.6 | DOWN | 265.12 | 0.0194307 | Chlorophyll a-b binding protein 50, chloroplastic |
| PGSC0003DMG400028467 | 3.6 | DOWN | 57.7 | 0.0259286 | Trehalose-6-phosphate synthase |
| PGSC0003DMG400000231 | 3.56 | DOWN | 110.19 | 0.0336489 | Aspartic proteinase nepenthesin-1 |
| PGSC0003DMG400006381 | 3.55 | DOWN | 93.4 | 0.00700526 | Glutamyl-tRNA reductase |
| PGSC0003DMG402017934 | 3.55 | DOWN | 46.23 | 0.0336489 | Pectinesterase |
| PGSC0003DMG400023764 | 3.55 | DOWN | 50.1 | 0.0289348 | Globulin |
| PGSC0003DMG400010408 | 3.54 | DOWN | 27.91 | 0.0454488 | Oxidoreductase |
| PGSC0003DMG400005901 | 3.53 | DOWN | 47.34 | 0.0411649 | Aspartic proteinase nepenthesin-2 |
| PGSC0003DMG400007562 | 3.53 | DOWN | 13.32 | 0.0477632 | SBT2 protein |
| PGSC0003DMG400012762 | 3.53 | DOWN | 96.59 | 0.0411649 | Glutathione-s-transferase theta, gst |
| PGSC0003DMG400002018 | 3.51 | DOWN | 183.58 | 0.00700526 | Glutathione S-transferase omega |
| PGSC0003DMG400013412 | 3.45 | DOWN | 87.81 | 0.03141 | Chlorophyll a-b binding protein 3C |
| PGSC0003DMG400024310 | 3.35 | DOWN | 88.95 | 0.049757 | Conserved gene of unknown function |
| PGSC0003DMG400021372 | 3.33 | DOWN | 74 | 0.0289348 | Fasciclin-like arabinogalactan protein 14 |
| PGSC0003DMG400013631 | 3.25 | DOWN | 59.06 | 0.0336489 | DNA-3-methyladenine glycosylase |
| PGSC0003DMG400009959 | 3.17 | DOWN | 222.29 | 0.0454488 | Ornithine decarboxylase |
| PGSC0003DMG400013879 | 3.15 | DOWN | 80.53 | 0.0360705 | Quinone reductase family protein |
| PGSC0003DMG400012591 | 3.13 | DOWN | 322.78 | 0.0388355 | Chlorophyll a-b binding protein CP24 10A, chloroplastic |
| PGSC0003DMG400000326 | 3.12 | DOWN | 648.62 | 0.0434749 | Ultraviolet-B-repressible protein |
| PGSC0003DMG400008472 | 3.06 | DOWN | 287.02 | 0.0336489 | W-3 desaturase |
| PGSC0003DMG402025083 | 3.03 | DOWN | 45 | 0.049757 | Peroxidase |
| PGSC0003DMG400019510 | 2.94 | DOWN | 365.17 | 0.0477632 | Geranylgeranyl diphosphate reductase, chloroplastic |
| PGSC0003DMG402007970 | 26.87 | UP | 89.28 | 0.00700526 | Conserved gene of unknown function |
| PGSC0003DMG400025433 | 18.95 | UP | 25.3 | 0.0194307 | Conserved gene of unknown function |
| PGSC0003DMG400006954 | 10.88 | UP | 17.15 | 0.0289348 | C2H2L domain class transcription factor |
| PGSC0003DMG400027914 | 8.96 | UP | 24.09 | 0.00700526 | Ascorbate oxidase |
| PGSC0003DMG400005515 | 8.77 | UP | 24.65 | 0.0119372 | Ascorbate oxidase |
| PGSC0003DMG400032792 | 8.69 | UP | 51.11 | 0.00700526 | Calmodulin-binding protein |
| PGSC0003DMG400022775 | 7.28 | UP | 12.76 | 0.0230277 | CMPG1b |
| PGSC0003DMG400016462 | 6.62 | UP | 76.08 | 0.00700526 | Conserved gene of unknown function |
| PGSC0003DMG400020697 | 6.36 | UP | 10.74 | 0.00700526 | Conserved gene of unknown function |
| PGSC0003DMG400017334 | 6.35 | UP | 57.12 | 0.00700526 | Heat shock factor protein |
| PGSC0003DMG400001221 | 6.29 | UP | 191.99 | 0.00700526 | EARLY flowering 4 protein |
| PGSC0003DMG400011633 | 6.19 | UP | 176.39 | 0.015972 | WRKY-type transcription factor |
| PGSC0003DMG400019971 | 6.07 | UP | 138.5 | 0.00700526 | Circadian clock-associated FKF1 |
| PGSC0003DMG400005835 | 5.94 | UP | 119.84 | 0.0388355 | WRKY transcription factor-30 |
| PGSC0003DMG400015468 | 5.9 | UP | 7.88 | 0.0289348 | Sugar transporter |
| PGSC0003DMG400008313 | 5.86 | UP | 197.91 | 0.00700526 | Conserved gene of unknown function |
| PGSC0003DMG400014771 | 5.67 | UP | 70.35 | 0.00700526 | Tyramine hydroxycinnamoyl transferase |
| PGSC0003DMG400015534 | 5.41 | UP | 35.16 | 0.0454488 | ZPT2-13 |
| CBF1 | 5.32 | UP | 50.68 | 0.0194307 | AP2 domain CBF protein |
| PGSC0003DMG403025788 | 5.23 | UP | 29.87 | 0.0388355 | Gene of unknown function |
| PGSC0003DMG400010491 | 5.13 | UP | 96.44 | 0.00700526 | Glucan endo-1,3-beta-D-glucosidase |
| PGSC0003DMG400028048 | 5.08 | UP | 199.42 | 0.00700526 | Pectin methylesterase inhibitor isoform |
| PGSC0003DMG400006905 | 4.73 | UP | 19.18 | 0.0194307 | Gene of unknown function |
| PGSC0003DMG400002910 | 4.71 | UP | 17.51 | 0.00700526 | Cf-2.1 |
| PGSC0003DMG400006311 | 4.64 | UP | 31.61 | 0.00700526 | Homeobox-leucine zipper protein HAT14 |
| PGSC0003DMG400004401 | 4.6 | UP | 16.51 | 0.0259286 | Kinase |
| PGSC0003DMG400003250 | 4.53 | UP | 25.69 | 0.049757 | Conserved gene of unknown function |
| PGSC0003DMG400014850 | 4.53 | UP | 236.51 | 0.00700526 | Proline-rich protein |
| PGSC0003DMG400007790 | 4.47 | UP | 183.5 | 0.00700526 | Metal ion binding protein |
| PGSC0003DMG400000584 | 4.42 | UP | 114.51 | 0.00700526 | Pseudo-response regulator 5 |
| PGSC0003DMG400006735 | 4.37 | UP | 674.53 | 0.0230277 | DNAJ heat shock N-terminal domain-containing protein |
| PGSC0003DMG400029046 | 4.36 | UP | 25.99 | 0.0259286 | Aromatic amino acid decarboxylase 1B |
| PGSC0003DMG400006179 | 4.35 | UP | 229.74 | 0.0119372 | Nodulin family protein |
| PGSC0003DMG400022312 | 4.34 | UP | 139.45 | 0.00700526 | Zinc finger protein |
| PGSC0003DMG400020844 | 4.28 | UP | 122.74 | 0.0230277 | Conserved gene of unknown function |
| PGSC0003DMG400028337 | 4.13 | UP | 19.89 | 0.0454488 | Gene of unknown function |
| PGSC0003DMG400025023 | 4.11 | UP | 60 | 0.0259286 | Avr9/Cf-9 rapidly elicited protein 20 |
| PGSC0003DMG400018925 | 4.1 | UP | 64.61 | 0.0119372 | Polyphenol oxidase B, chloroplastic |
| PGSC0003DMG400036566 | 4.09 | UP | 71.9 | 0.0434749 | Ethylene response factor 5 |
| PGSC0003DMG400021331 | 4.07 | UP | 46.75 | 0.0289348 | PEN1 |
| PGSC0003DMG400028818 | 4.02 | UP | 89.07 | 0.0411649 | Transcription factor |
| PGSC0003DMG400002221 | 4 | UP | 39.45 | 0.0360705 | Deacetylase |
| PGSC0003DMG400030534 | 3.99 | UP | 25 | 0.03141 | Wall-associated kinase |
| PGSC0003DMG400005899 | 3.94 | UP | 694.44 | 0.0360705 | CCR4-associated factor |
| PGSC0003DMG400025022 | 3.94 | UP | 54.85 | 0.0360705 | Avr9/Cf-9 rapidly elicited protein 20 |
| PGSC0003DMG400003229 | 3.9 | UP | 126.84 | 0.00700526 | Conserved gene of unknown function |
| PGSC0003DMG400009955 | 3.87 | UP | 29.15 | 0.049757 | Syringolide-induced protein 14-1-1 |
| PGSC0003DMG400040046 | 3.84 | UP | 107.33 | 0.015972 | Ethylene response factor 5 |
| PGSC0003DMG400005822 | 3.74 | UP | 45.12 | 0.015972 | Potassium channel tetramerization domain-containing protein |
| PGSC0003DMG400025558 | 3.72 | UP | 14.87 | 0.0336489 | ATP binding protein |
| PGSC0003DMG402018475 | 3.69 | UP | 131.64 | 0.03141 | ABA 8'-hydroxylase CYP707A1 |
| PGSC0003DMG400025414 | 3.69 | UP | 40.96 | 0.00700526 | Zinc finger protein CONSTANS-LIKE 15 |
| PGSC0003DMG400030364 | 3.62 | UP | 90.36 | 0.015972 | Avr9/Cf-9 rapidly elicited protein 284 |
| PGSC0003DMG400031457 | 3.58 | UP | 334.17 | 0.03141 | Phenylalanine ammonia-lyase 1 |
| PGSC0003DMG400020843 | 3.52 | UP | 768.93 | 0.0119372 | Photoassimilate-responsive protein PAR |
| PGSC0003DMG400033858 | 3.49 | UP | 474.83 | 0.00700526 | Starch phosphorylase L |
| PGSC0003DMG400036774 | 3.48 | UP | 116.67 | 0.0434749 | Conserved gene of unknown function |
| PGSC0003DMG400006378 | 3.45 | UP | 31.88 | 0.0259286 | Multidrug resistance pump |
| PGSC0003DMG400008202 | 3.39 | UP | 167.87 | 0.0360705 | Calmodulin binding protein |
| PGSC0003DMG400024627 | 3.38 | UP | 253.37 | 0.00700526 | GRP 2 |
| PGSC0003DMG400010351 | 3.37 | UP | 15.32 | 0.0194307 | Serine-threonine protein kinase, plant-type |
| PGSC0003DMG400004312 | 3.35 | UP | 81.96 | 0.0336489 | 9-cis-epoxycarotenoid dioxygenase |
| PGSC0003DMG400025473 | 3.3 | UP | 238.02 | 0.0230277 | CBS domain containing protein |
| ERF6 | 3.29 | UP | 276.99 | 0.0336489 | Ethylene response factor 5 |
| PGSC0003DMG400032273 | 3.26 | UP | 189.05 | 0.0336489 | N-acetyltransferase |
| PGSC0003DMG400014766 | 3.22 | UP | 123.43 | 0.0289348 | 41 kD chloroplast nucleoid DNA binding protein (CND41) |
| GOLS3 | 3.17 | UP | 78.81 | 0.0259286 | Galactinol synthase |
| PGSC0003DMG400033632 | 3.12 | UP | 25.16 | 0.0289348 | Cf-2.2 |
| PGSC0003DMG400041402 | 3.09 | UP | 74.78 | 0.03141 | Basic 7S globulin 2 small subunit |
| PGSC0003DMG400010724 | 3.06 | UP | 84.18 | 0.0434749 | ERF transcription factor 4 |
| PGSC0003DMG400035478 | 3.03 | UP | 352.08 | 0.0336489 | Conserved gene of unknown function |
| PGSC0003DMG400003217 | 3.02 | UP | 590.93 | 0.0477632 | Avr9/Cf-9 rapidly elicited protein 65 |
| PGSC0003DMG400012201 | 3.02 | UP | 82.58 | 0.0411649 | Potassium channel tetramerization domain-containing protein |
| PGSC0003DMG402003983 | 2.92 | UP | 63.18 | 0.0454488 | ATP binding protein |

**Supplemental Table 3: list of genes differentially regulated in Waneta.** Genes are sorted by regulation direction down indicate genes up regulated in Lso- treatment whereas up indicate genes up regulated in Lso+ treatment. The genes are also sorted by fold change. Sample 1 :Waneta Lso- compare to sample2: Waneta-Lso+

| **Gene name** | **Fold change** | **direction** | **Total fpkm** | **q-value** | **Gene description** |
| --- | --- | --- | --- | --- | --- |
| PGSC0003DMG400000332 | 126.53 | DOWN | 158.94 | 0.002151 | Salt responsive protein 2 |
| PGSC0003DMG400010713 | 86.79 | DOWN | 466.04 | 0.002151 | Salt responsive protein 2 |
| PGSC0003DMG400043507 | 45.19 | DOWN | 4.55 | 0.002151 | Cation-transporting atpase plant |
| PGSC0003DMG400021325 | 41.56 | DOWN | 18.8 | 0.002151 | U-box protein |
| PGSC0003DMG400009116 | 34.87 | DOWN | 15.68 | 0.002151 | Membrane receptor 1 |
| XTH1 | 34.42 | DOWN | 24.18 | 0.002151 | Xyloglucan endotransglycosylase |
| PGSC0003DMG400016497 | 34.09 | DOWN | 46.21 | 0.002151 | Conserved gene of unknown function |
| PGSC0003DMG400016326 | 27.21 | DOWN | 4.14 | 0.0377582 | Ring finger protein |
| PGSC0003DMG400028153 | 25.02 | DOWN | 336.75 | 0.002151 | Hin1 |
| PGSC0003DMG400008711 | 22.94 | DOWN | 7.07 | 0.002151 | Avr9/Cf-9 rapidly elicited protein 76 |
| PGSC0003DMG400021684 | 21.91 | DOWN | 183.84 | 0.002151 | E3 ubiquitin-protein ligase RMA1H1 |
| PGSC0003DMG400002899 | 20.03 | DOWN | 7.4 | 0.0057258 | AP2/ERF domain-containing transcription factor |
| PGSC0003DMG400030640 | 19.42 | DOWN | 36.23 | 0.002151 | Conserved gene of unknown function |
| PGSC0003DMG400007816 | 19.16 | DOWN | 13.9 | 0.002151 | UDP-glucuronate 5-epimerase |
| PGSC0003DMG400008438 | 18.82 | DOWN | 3.9 | 0.0073051 | Metal ion binding protein |
| PGSC0003DMG400022775 | 18.39 | DOWN | 12.7 | 0.002151 | CMPG1b |
| PGSC0003DMG400015533 | 18.3 | DOWN | 11.79 | 0.0040151 | ZPT2-13 |
| PGSC0003DMG400005649 | 17.63 | DOWN | 592.32 | 0.002151 | Calmodulin-binding protein |
| PGSC0003DMG402008903 | 17.62 | DOWN | 91.26 | 0.002151 | Conserved gene of unknown function |
| PGSC0003DMG400018942 | 17.35 | DOWN | 68.21 | 0.002151 | Autoinhibited calcium ATPase |
| PGSC0003DMG400010509 | 17.25 | DOWN | 132.18 | 0.002151 | Axi 1 protein |
| PGSC0003DMG400025163 | 16.79 | DOWN | 35.18 | 0.002151 | Conserved gene of unknown function |
| PGSC0003DMG400011249 | 16.28 | DOWN | 33.48 | 0.002151 | F-box family protein |
| PGSC0003DMG400014823 | 16.06 | DOWN | 14.04 | 0.030665 | Xyloglucan endotransglucosylase-hydrolase XTH3 |
| PGSC0003DMG400017772 | 15.8 | DOWN | 11.31 | 0.002151 | Arabinogalactan-protein |
| PGSC0003DMG400009021 | 15.79 | DOWN | 4.18 | 0.0103049 | Gibberellin 2-oxidase |
| PGSC0003DMG400030673 | 15.7 | DOWN | 3.34 | 0.0057258 | Gene of unknown function |
| PGSC0003DMG401021514 | 15.33 | DOWN | 15.92 | 0.0057258 | Receptor protein kinase |
| PGSC0003DMG400005899 | 15.04 | DOWN | 452.53 | 0.002151 | CCR4-associated factor |
| PGSC0003DMG400018623 | 15.03 | DOWN | 17.12 | 0.002151 | Mads box protein |
| PGSC0003DMG400015668 | 15.01 | DOWN | 4.17 | 0.0040151 | Transcription factor |
| PGSC0003DMG400043830 | 14.88 | DOWN | 3.74 | 0.002151 | Autoinhibited calcium ATPase |
| TSF | 14.82 | DOWN | 4.24 | 0.0177243 | Tuber-specific and sucrose-responsive element binding factor |
| PGSC0003DMG401003156 | 14.79 | DOWN | 6.96 | 0.0073051 | Glycosyltransferase, CAZy family GT2 |
| PGSC0003DMG400003217 | 14.66 | DOWN | 417.08 | 0.002151 | Avr9/Cf-9 rapidly elicited protein 65 |
| PGSC0003DMG400003228 | 14.63 | DOWN | 51.99 | 0.002151 | SAUR family protein |
| PGSC0003DMG400007820 | 14.19 | DOWN | 69.43 | 0.002151 | Stress induced protein |
| STP2C | 14.07 | DOWN | 54.79 | 0.002151 | Protein phosphatase 2C |
| PGSC0003DMG400002524 | 13.9 | DOWN | 1115.02 | 0.002151 | Conserved gene of unknown function |
| PGSC0003DMG400004766 | 13.75 | DOWN | 20.38 | 0.0073051 | GEM 5 |
| PGSC0003DMG400017757 | 13.53 | DOWN | 15.3 | 0.002151 | Arabinogalactan-protein |
| PGSC0003DMG400011633 | 13.23 | DOWN | 257.95 | 0.002151 | WRKY-type transcription factor |
| PGSC0003DMG400046726 | 13.21 | DOWN | 78.33 | 0.002151 | F-box family protein |
| PGSC0003DMG400010439 | 13.14 | DOWN | 41.77 | 0.002151 | CHP-rich zinc finger protein |
| PGSC0003DMG400025627 | 13.12 | DOWN | 35.68 | 0.002151 | Protein COBRA |
| PGSC0003DMG400024703 | 12.76 | DOWN | 200.73 | 0.002151 | SRC2 |
| PGSC0003DMG400003518 | 12.72 | DOWN | 28.58 | 0.002151 | Conserved gene of unknown function |
| PGSC0003DMG400033123 | 12.31 | DOWN | 21.47 | 0.002151 | Basic 7S globulin 2 small subunit |
| PGSC0003DMG400013898 | 12.3 | DOWN | 14.82 | 0.002151 | Hcr2-0B |
| PGSC0003DMG400036566 | 12.27 | DOWN | 168.33 | 0.002151 | Ethylene response factor 5 |
| PGSC0003DMG400031457 | 12.17 | DOWN | 454.78 | 0.002151 | Phenylalanine ammonia-lyase 1 |
| PGSC0003DMG400026436 | 12.02 | DOWN | 5.73 | 0.002151 | Serine-threonine protein kinase, plant-type |
| PGSC0003DMG400002607 | 11.98 | DOWN | 26.17 | 0.002151 | Conserved gene of unknown function |
| PKPI-A5 | 11.89 | DOWN | 4.96 | 0.0271321 | Aspartic protease inhibitor 5 |
| PGSC0003DMG400016638 | 11.86 | DOWN | 191.51 | 0.002151 | Sorghum bicolor protein targeted either to mitochondria or chloroplast proteins T50848 |
| PGSC0003DMG402021514 | 11.82 | DOWN | 55.62 | 0.0088917 | Gene of unknown function |
| PGSC0003DMG400016149 | 11.71 | DOWN | 39.57 | 0.002151 | Avr9/Cf-9 rapidly elicited protein 75 |
| PGSC0003DMG400003008 | 11.51 | DOWN | 1.86 | 0.002151 | Kinesin |
| ERF6 | 11.46 | DOWN | 275.49 | 0.002151 | Ethylene response factor 5 |
| PGSC0003DMG400030827 | 11.45 | DOWN | 5.65 | 0.002151 | Phi-1 protein |
| PGSC0003DMG400030309 | 11.44 | DOWN | 10.87 | 0.002151 | Auxin-regulated protein |
| PGSC0003DMG400004037 | 11.17 | DOWN | 6.87 | 0.002151 | ATP binding protein |
| PGSC0003DMG400011073 | 10.99 | DOWN | 19.61 | 0.002151 | Avr9/Cf-9 rapidly elicited protein 137 |
| PGSC0003DMG400011633 | 10.76 | DOWN | 22.41 | 0.0342702 | WRKY-type transcription factor |
| PGSC0003DMG400027781 | 10.7 | DOWN | 32.31 | 0.002151 | Conserved gene of unknown function |
| PGSC0003DMG400028198 | 10.54 | DOWN | 500.85 | 0.002151 | VQ |
| PGSC0003DMG400014184 | 10.51 | DOWN | 28.92 | 0.0457077 | Nudix hydrolase 17, mitochondrial |
| PGSC0003DMG400014823 | 10.23 | DOWN | 8.88 | 0.0282933 | Xyloglucan endotransglucosylase-hydrolase XTH3 |
| PGSC0003DMG400009028 | 10.17 | DOWN | 4.79 | 0.002151 | Conserved gene of unknown function |
| PGSC0003DMG400011633 | 10.14 | DOWN | 63.5 | 0.002151 | WRKY-type transcription factor |
| PGSC0003DMG400013744 | 10.07 | DOWN | 107.24 | 0.002151 | Ethylene-responsive element binding factor |
| PGSC0003DMG400005515 | 9.91 | DOWN | 31.02 | 0.002151 | Ascorbate oxidase |
| PGSC0003DMG400013488 | 9.91 | DOWN | 14.93 | 0.002151 | F-box/kelch protein |
| PGSC0003DMG400005909 | 9.81 | DOWN | 28.46 | 0.002151 | Regulator of gene silencing |
| PGSC0003DMG400025244 | 9.8 | DOWN | 13.98 | 0.002151 | Pentatricopeptide repeat-containing protein |
| PGSC0003DMG400030608 | 9.72 | DOWN | 175.89 | 0.002151 | Calcium-binding protein CML24 |
| PGSC0003DMG400028337 | 9.66 | DOWN | 19.84 | 0.002151 | Gene of unknown function |
| PGSC0003DMG400035643 | 9.64 | DOWN | 523.93 | 0.002151 | Drought-induced protein SDi |
| PGSC0003DMG400017301 | 9.62 | DOWN | 12.2 | 0.002151 | ER glycerol-phosphate acyltransferase |
| PGSC0003DMG400024100 | 9.57 | DOWN | 101.3 | 0.002151 | Conserved gene of unknown function |
| PGSC0003DMG400030165 | 9.56 | DOWN | 194.33 | 0.002151 | Avr9/Cf-9 rapidly elicited protein 180 |
| PGSC0003DMG400009078 | 9.49 | DOWN | 12.63 | 0.002151 | Receptor serine/threonine kinase |
| PGSC0003DMG400019232 | 9.47 | DOWN | 65.43 | 0.002151 | GRAS2 |
| PGSC0003DMG400009869 | 9.43 | DOWN | 233.19 | 0.002151 | DNAJ |
| PGSC0003DMG401021514 | 9.24 | DOWN | 14.28 | 0.002151 | Receptor protein kinase |
| PGSC0003DMG400004766 | 9.13 | DOWN | 181.2 | 0.002151 | GEM 5 |
| PGSC0003DMG400030809 | 9.11 | DOWN | 142.89 | 0.002151 | Phi-1 protein |
| PGSC0003DMG400040046 | 9.08 | DOWN | 100.12 | 0.002151 | Ethylene response factor 5 |
| PGSC0003DMG400005456 | 9.01 | DOWN | 215.39 | 0.002151 | Protein phosphatase 2c |
| PGSC0003DMG400016261 | 8.93 | DOWN | 9.2 | 0.002151 | Conserved gene of unknown function |
| PGSC0003DMG400016892 | 8.9 | DOWN | 72.56 | 0.002151 | Conserved gene of unknown function |
| PGSC0003DMG400013928 | 8.81 | DOWN | 26.99 | 0.002151 | Cytokinin-regulated kinase 1 |
| PGSC0003DMG400012822 | 8.8 | DOWN | 82.85 | 0.002151 | Stem-specific protein TSJT1 |
| PGSC0003DMG400032273 | 8.77 | DOWN | 196.29 | 0.002151 | N-acetyltransferase |
| PGSC0003DMG400004284 | 8.75 | DOWN | 83.17 | 0.002151 | Disease resistance protein |
| PGSC0003DMG400012201 | 8.69 | DOWN | 109.12 | 0.002151 | Potassium channel tetramerization domain-containing protein |
| PGSC0003DMG400047273 | 8.6 | DOWN | 4.3 | 0.0040151 | Autoinhibited calcium ATPase |
| PGSC0003DMG400033317 | 8.6 | DOWN | 203.19 | 0.002151 | Avr9/Cf-9 rapidly elicited protein 180 |
| PGSC0003DMG401030312 | 8.59 | DOWN | 12.15 | 0.002151 | ZPT2-12 |
| PGSC0003DMG402018475 | 8.56 | DOWN | 169.34 | 0.002151 | ABA 8'-hydroxylase CYP707A1 |
| PAL-1 | 8.56 | DOWN | 123.46 | 0.002151 | Phenylalanine ammonia-lyase |
| SGT3.1 | 8.55 | DOWN | 3.69 | 0.0057258 | SGA |
| PGSC0003DMG400009734 | 8.43 | DOWN | 15.3 | 0.002151 | Conserved gene of unknown function |
| PGSC0003DMG402018475 | 8.42 | DOWN | 59.86 | 0.002151 | ABA 8'-hydroxylase CYP707A1 |
| PGSC0003DMG400024586 | 8.35 | DOWN | 17.43 | 0.0057258 | PBng143 |
| PGSC0003DMG400014823 | 8.31 | DOWN | 14.37 | 0.0191534 | Xyloglucan endotransglucosylase-hydrolase XTH3 |
| PGSC0003DMG402025869 | 8.29 | DOWN | 65.72 | 0.002151 | C2 domain-containing protein |
| PGSC0003DMG400005844 | 8.25 | DOWN | 591.39 | 0.002151 | B2 protein |
| PGSC0003DMG400011751 | 8.24 | DOWN | 125.38 | 0.002151 | 2-oxoglutarate-dependent dioxygenase |
| PGSC0003DMG400007200 | 8.2 | DOWN | 170.71 | 0.002151 | VQ |
| PGSC0003DMG400044116 | 8.19 | DOWN | 65.12 | 0.002151 | Beta-glucan-binding protein 4 |
| PGSC0003DMG400014184 | 8.13 | DOWN | 104.14 | 0.002151 | Nudix hydrolase 17, mitochondrial |
| PGSC0003DMG400010022 | 8.13 | DOWN | 62.86 | 0.002151 | Patatin 3 |
| PGSC0003DMG400027914 | 8.04 | DOWN | 36.45 | 0.002151 | Ascorbate oxidase |
| PGSC0003DMG400023957 | 7.94 | DOWN | 20.19 | 0.002151 | Prephenate dehydrogenase |
| PGSC0003DMG400009007 | 7.92 | DOWN | 104.55 | 0.002151 | PIT1 |
| PGSC0003DMG400027584 | 7.87 | DOWN | 18.83 | 0.002151 | Gene of unknown function |
| PGSC0003DMG400005835 | 7.85 | DOWN | 45.15 | 0.002151 | WRKY transcription factor-30 |
| PGSC0003DMG400021902 | 7.8 | DOWN | 23.94 | 0.002151 | Squamosa promoter-binding protein |
| PGSC0003DMG400030828 | 7.79 | DOWN | 10.03 | 0.002151 | Phi-1 protein |
| PGSC0003DMG400000211 | 7.78 | DOWN | 651.44 | 0.002151 | WRKY transcription factor |
| PGSC0003DMG400013022 | 7.68 | DOWN | 261.1 | 0.002151 | Calcium-transporting ATPase 2, plasma membrane-type |
| ERF3 | 7.66 | DOWN | 496.78 | 0.002151 | Ethylene responsive element binding protein C2 |
| PGSC0003DMG400021331 | 7.64 | DOWN | 108.17 | 0.002151 | PEN1 |
| LOX3.1 | 7.62 | DOWN | 120.98 | 0.002151 | Lipoxygenase |
| PGSC0003DMG400017946 | 7.61 | DOWN | 18.87 | 0.002151 | S locus glycoprotein |
| PGSC0003DMG400005401 | 7.6 | DOWN | 284.9 | 0.002151 | Sigma factor binding protein 1 |
| PGSC0003DMG400014771 | 7.6 | DOWN | 262.25 | 0.002151 | Tyramine hydroxycinnamoyl transferase |
| PGSC0003DMG400009115 | 7.58 | DOWN | 287.41 | 0.002151 | Membrane receptor 1 |
| PGSC0003DMG400021574 | 7.52 | DOWN | 61.28 | 0.002151 | 21kD protein |
| PGSC0003DMG400003250 | 7.52 | DOWN | 29.29 | 0.002151 | Conserved gene of unknown function |
| PGSC0003DMG400020564 | 7.4 | DOWN | 5.49 | 0.002151 | Avr9/Cf-9 rapidly elicited protein 216 |
| NCED1 | 7.36 | DOWN | 45.91 | 0.002151 | 9-cis-epoxycarotenoid dioxygenase |
| PGSC0003DMG400030462 | 7.35 | DOWN | 42.01 | 0.002151 | Avr9/Cf-9 rapidly elicited protein 216 |
| PGSC0003DMG400020261 | 7.35 | DOWN | 3.54 | 0.0243888 | Polcalcin Jun o |
| PGSC0003DMG400024148 | 7.31 | DOWN | 2.57 | 0.0414827 | Serine-threonine protein kinase, plant-type |
| PGSC0003DMG400021778 | 7.25 | DOWN | 10.47 | 0.0040151 | Zinc finger protein |
| PGSC0003DMG400022616 | 7.19 | DOWN | 34.22 | 0.002151 | Conserved gene of unknown function |
| PGSC0003DMG400009932 | 7.19 | DOWN | 240.55 | 0.002151 | MRNA, 952 bp sequence |
| PGSC0003DMG400001853 | 7.19 | DOWN | 94.18 | 0.002151 | NAK-type protein kinase |
| PGSC0003DMG400027046 | 7.17 | DOWN | 74.31 | 0.002151 | DC1 domain containing protein |
| PGSC0003DMG400032778 | 7.16 | DOWN | 2.87 | 0.0425183 | Conserved gene of unknown function |
| PGSC0003DMG400021777 | 7.16 | DOWN | 31.01 | 0.002151 | Zinc finger protein |
| PGSC0003DMG400006890 | 7.14 | DOWN | 97.15 | 0.002151 | Conserved gene of unknown function |
| PGSC0003DMG400004860 | 7.05 | DOWN | 151.87 | 0.002151 | Auxin-regulated dual specificity cytosolic kinase |
| PGSC0003DMG400029371 | 7.03 | DOWN | 101.3 | 0.002151 | DNA-binding protein NtWRKY3 |
| PGSC0003DMG400047346 | 6.99 | DOWN | 151.1 | 0.002151 | BRASSINOSTEROID INSENSITIVE 1-associated receptor kinase 1 |
| PGSC0003DMG400026232 | 6.98 | DOWN | 88.71 | 0.002151 | Avr9/Cf-9 rapidly elicited protein 1 |
| PGSC0003DMG400028575 | 6.98 | DOWN | 2.61 | 0.002151 | Verticillium wilt disease resistance protein |
| PGSC0003DMG400025023 | 6.95 | DOWN | 42.65 | 0.002151 | Avr9/Cf-9 rapidly elicited protein 20 |
| PGSC0003DMG400014774 | 6.94 | DOWN | 8.07 | 0.002151 | N-hydroxycinnamoyl-CoA:tyramine N-hydroxycinnamoyl transferase THT7-1 |
| PGSC0003DMG400026384 | 6.92 | DOWN | 14.24 | 0.0040151 | Serine/threonine protein kinase family protein |
| PGSC0003DMG400028797 | 6.89 | DOWN | 24.43 | 0.002151 | Calcium-binding allergen Ole e 8 |
| PGSC0003DMG400020909 | 6.88 | DOWN | 20.77 | 0.002151 | ATP binding protein |
| PGSC0003DMG400029050 | 6.84 | DOWN | 5.3 | 0.002151 | Aromatic amino acid decarboxylase 1B |
| PGSC0003DMG400016063 | 6.82 | DOWN | 37.86 | 0.002151 | Conserved gene of unknown function |
| PGSC0003DMG400014779 | 6.81 | DOWN | 8.16 | 0.0103049 | Aromatic amino acid decarboxylase 1A |
| PGSC0003DMG400041402 | 6.79 | DOWN | 39.12 | 0.002151 | Basic 7S globulin 2 small subunit |
| PGSC0003DMG402007388 | 6.77 | DOWN | 99.41 | 0.002151 | MRNA, 1346 bp sequence |
| PGSC0003DMG400002618 | 6.74 | DOWN | 41.63 | 0.002151 | Conserved gene of unknown function |
| PGSC0003DMG400007244 | 6.73 | DOWN | 33.23 | 0.002151 | GDSL-lipase 1 |
| PGSC0003DMG400016928 | 6.72 | DOWN | 9.98 | 0.002151 | Conserved gene of unknown function |
| PGSC0003DMG401025150 | 6.6 | DOWN | 12.9 | 0.002151 | Conserved gene of unknown function |
| PGSC0003DMG400004822 | 6.6 | DOWN | 2.18 | 0.002151 | Oxidoreductase |
| PGSC0003DMG400002291 | 6.6 | DOWN | 7.01 | 0.002151 | Triacylglycerol lipase |
| PGSC0003DMG400013802 | 6.59 | DOWN | 3.56 | 0.002151 | Serine-threonine protein kinase, plant-type |
| PGSC0003DMG400004705 | 6.55 | DOWN | 85.24 | 0.002151 | Conserved gene of unknown function |
| PGSC0003DMG401007388 | 6.53 | DOWN | 89.99 | 0.002151 | MRNA, 1346 bp sequence |
| PGSC0003DMG400020608 | 6.49 | DOWN | 458.7 | 0.002151 | DNA-binding protein 3 |
| PGSC0003DMG400018182 | 6.49 | DOWN | 56.96 | 0.002151 | Ring finger protein |
| PGSC0003DMG400033363 | 6.42 | DOWN | 10.26 | 0.002151 | Gene of unknown function |
| PGSC0003DMG400011326 | 6.41 | DOWN | 178.46 | 0.002151 | Ring finger protein |
| PGSC0003DMG400007886 | 6.38 | DOWN | 41.27 | 0.002151 | Glycerol-3-phosphate transporter / glycerol 3-phosphate permease, putative |
| PGSC0003DMG401029345 | 6.35 | DOWN | 7.67 | 0.0342702 | Isoform 2 of TMV resistance protein N |
| PGSC0003DMG400028152 | 6.33 | DOWN | 6.96 | 0.0088917 | Hin1 |
| PGSC0003DMG400001923 | 6.32 | DOWN | 188.6 | 0.002151 | Matrix metalloprotease 1 |
| PGSC0003DMG400021802 | 6.31 | DOWN | 81.59 | 0.002151 | Scarecrow |
| PGSC0003DMG400024781 | 6.29 | DOWN | 351.63 | 0.002151 | GRAS4 |
| PGSC0003DMG400017065 | 6.28 | DOWN | 1.89 | 0.01619 | Disease resistance protein RGA1 |
| PGSC0003DMG400005745 | 6.25 | DOWN | 893.95 | 0.002151 | Calcium-binding allergen Ole e 8 |
| PGSC0003DMG400002134 | 6.23 | DOWN | 5.34 | 0.002151 | Clathrin assembly protein |
| PGSC0003DMG400000512 | 6.23 | DOWN | 40.91 | 0.002151 | Stress-associated protein 11 |
| PGSC0003DMG400026346 | 6.17 | DOWN | 107.25 | 0.002151 | F-box family protein |
| PGSC0003DMG400028185 | 6.16 | DOWN | 4.74 | 0.0040151 | Metal ion binding protein |
| PGSC0003DMG400027176 | 6.14 | DOWN | 661.09 | 0.002151 | Transcription factor |
| STTHT | 6.1 | DOWN | 27.07 | 0.002151 | Tyramine hydroxycinnamoyl transferase |
| PGSC0003DMG400010173 | 6.08 | DOWN | 148.62 | 0.002151 | Avr9/Cf-9 rapidly elicited protein 140 |
| PGSC0003DMG400019398 | 6.07 | DOWN | 844.18 | 0.002151 | Ubiquitin carrier protein |
| PGSC0003DMG400008202 | 6.03 | DOWN | 349.81 | 0.002151 | Calmodulin binding protein |
| STWIPK | 6.03 | DOWN | 808.76 | 0.002151 | MAP kinase |
| PGSC0003DMG400045370 | 6 | DOWN | 10.96 | 0.002151 | Basic 7S globulin 2 small subunit |
| PGSC0003DMG400002930 | 6 | DOWN | 24.15 | 0.002151 | Jasmonate ZIM-domain protein 1 |
| PONI2 | 5.97 | DOWN | 327.56 | 0.002151 | Membrane protein |
| PGSC0003DMG400031105 | 5.95 | DOWN | 9.71 | 0.0103049 | Hydroxyproline-rich glycoprotein GAS31 |
| PGSC0003DMG400012763 | 5.94 | DOWN | 14.48 | 0.002151 | C-4 sterol methyl oxidase |
| PGSC0003DMG400011071 | 5.93 | DOWN | 16.07 | 0.002151 | Kinase |
| PGSC0003DMG400015492 | 5.92 | DOWN | 3.71 | 0.002151 | ATP binding protein |
| PGSC0003DMG401021549 | 5.92 | DOWN | 218.96 | 0.002151 | Phenylalanine ammonia-lyase |
| PGSC0003DMG400022151 | 5.89 | DOWN | 31.35 | 0.002151 | Chaperone protein DNAj |
| PGSC0003DMG400046292 | 5.86 | DOWN | 67.6 | 0.002151 | Conserved gene of unknown function |
| PGSC0003DMG400029046 | 5.84 | DOWN | 19.98 | 0.002151 | Aromatic amino acid decarboxylase 1B |
| PGSC0003DMG400006268 | 5.81 | DOWN | 7.72 | 0.002151 | Disease resistance protein SlVe2 |
| PGSC0003DMG400019884 | 5.81 | DOWN | 44.17 | 0.002151 | WRKY transcription factor |
| PGSC0003DMG400002507 | 5.81 | DOWN | 8.03 | 0.002151 | Zinc finger protein |
| PGSC0003DMG400010049 | 5.78 | DOWN | 80.62 | 0.002151 | Transferase, transferring glycosyl groups |
| PGSC0003DMG400014345 | 5.76 | DOWN | 218.07 | 0.002151 | Acyl-CoA synthetase |
| PGSC0003DMG400010132 | 5.76 | DOWN | 139.25 | 0.002151 | Conserved gene of unknown function |
| PGSC0003DMG400000067 | 5.75 | DOWN | 400.27 | 0.002151 | Late embryogenesis (Lea) |
| PGSC0003DMG400025755 | 5.72 | DOWN | 120.05 | 0.002151 | RING-H2 finger protein |
| PGSC0003DMG400010225 | 5.66 | DOWN | 14.51 | 0.002151 | Cysteine protease |
| PGSC0003DMG400005390 | 5.6 | DOWN | 26.69 | 0.002151 | Calmodulin-binding protein |
| PGSC0003DMG402027023 | 5.54 | DOWN | 190.12 | 0.002151 | Nam 1 |
| PGSC0003DMG400025836 | 5.53 | DOWN | 11.54 | 0.002151 | Conserved gene of unknown function |
| PGSC0003DMG400019578 | 5.51 | DOWN | 1139.01 | 0.002151 | Conserved gene of unknown function |
| PGSC0003DMG402018257 | 5.49 | DOWN | 6.61 | 0.044629 | Leucine-rich repeat-containing protein |
| PGSC0003DMG400003982 | 5.47 | DOWN | 12.02 | 0.002151 | ATP binding protein |
| PGSC0003DMG400023680 | 5.46 | DOWN | 91.16 | 0.002151 | Conserved gene of unknown function |
| PGSC0003DMG400030829 | 5.45 | DOWN | 7.72 | 0.002151 | Phi-1 protein |
| PGSC0003DMG400019232 | 5.44 | DOWN | 15.35 | 0.0088917 | GRAS2 |
| PGSC0003DMG400011752 | 5.39 | DOWN | 53.77 | 0.002151 | Cellulose synthase |
| PGSC0003DMG400013696 | 5.39 | DOWN | 2.56 | 0.0040151 | Cytochrome P450 |
| PGSC0003DMG401003156 | 5.39 | DOWN | 8.16 | 0.002151 | Glycosyltransferase, CAZy family GT2 |
| PGSC0003DMG400012160 | 5.34 | DOWN | 69.64 | 0.002151 | WRKY transcription factor-30 |
| EXLA1 | 5.33 | DOWN | 89.4 | 0.002151 | Expansin |
| PGSC0003DMG400021508 | 5.31 | DOWN | 683.1 | 0.002151 | C2H2-type zinc finger protein |
| PGSC0003DMG400005036 | 5.31 | DOWN | 21.29 | 0.002151 | Cotton fiber expressed protein 1 |
| PGSC0003DMG400033084 | 5.29 | DOWN | 9.48 | 0.002151 | Chlorophyll a/b-binding protein (cab-12) |
| PGSC0003DMG400006255 | 5.2 | DOWN | 4.95 | 0.002151 | Verticillium wilt disease resistance protein |
| PGSC0003DMG400025214 | 5.19 | DOWN | 31.86 | 0.002151 | Ankyrin repeat-containing protein |
| PGSC0003DMG400026261 | 5.19 | DOWN | 3.97 | 0.01619 | ATERF-2/ATERF2/ERF2 |
| PGSC0003DMG402017902 | 5.18 | DOWN | 37.92 | 0.002151 | Boron transporter |
| PGSC0003DMG402003983 | 5.17 | DOWN | 47.75 | 0.002151 | ATP binding protein |
| PGSC0003DMG400017969 | 5.17 | DOWN | 1.83 | 0.002151 | Hcr2-0A |
| PGSC0003DMG400017756 | 5.13 | DOWN | 15.47 | 0.002151 | Arabinogalactan-protein |
| PGSC0003DMG400001396 | 5.13 | DOWN | 26.92 | 0.002151 | Avr9/Cf-9 rapidly elicited protein 231 |
| PGSC0003DMG400042481 | 5.11 | DOWN | 6.2 | 0.0073051 | Circadian clock coupling factor ZGT |
| PGSC0003DMG400008386 | 5.1 | DOWN | 99.73 | 0.002151 | ATELC |
| PGSC0003DMG400000811 | 5.08 | DOWN | 369.46 | 0.002151 | RAV transcription factor |
| PGSC0003DMG400028467 | 5.08 | DOWN | 102.51 | 0.002151 | Trehalose-6-phosphate synthase |
| PGSC0003DMG400003527 | 5.07 | DOWN | 63.81 | 0.002151 | Disease resistance protein RGA2 |
| PGSC0003DMG400007073 | 5.05 | DOWN | 149.42 | 0.002151 | Epidermis-specific secreted glycoprotein EP1 |
| PGSC0003DMG400011016 | 5.04 | DOWN | 94.62 | 0.002151 | Soul heme-binding family protein |
| PGSC0003DMG400006041 | 5.02 | DOWN | 15.54 | 0.002151 | RING-H2 finger protein ATL1L |
| PGSC0003DMG400026623 | 5 | DOWN | 97.54 | 0.002151 | Conserved gene of unknown function |
| PGSC0003DMG400010410 | 4.99 | DOWN | 17.28 | 0.002151 | Calcium-binding protein CML23 |
| PGSC0003DMG400006905 | 4.99 | DOWN | 16.74 | 0.002151 | Gene of unknown function |
| PGSC0003DMG400030892 | 4.96 | DOWN | 84.66 | 0.002151 | Calmodulin |
| PGSC0003DMG400030119 | 4.95 | DOWN | 8.01 | 0.002151 | Serine-threonine protein kinase, plant-type |
| PGSC0003DMG400022383 | 4.94 | DOWN | 168.96 | 0.002151 | Conserved gene of unknown function |
| PGSC0003DMG400026127 | 4.93 | DOWN | 64.1 | 0.002151 | Beta-ketoacyl-coa synthase family protein |
| PGSC0003DMG400013482 | 4.93 | DOWN | 267 | 0.002151 | Conserved gene of unknown function |
| PGSC0003DMG400018690 | 4.89 | DOWN | 7.57 | 0.002151 | Conserved gene of unknown function |
| PGSC0003DMG400021122 | 4.87 | DOWN | 10.19 | 0.002151 | Conserved gene of unknown function |
| PGSC0003DMG400024539 | 4.86 | DOWN | 38.39 | 0.002151 | ATEXO70E2 |
| PGSC0003DMG400022620 | 4.84 | DOWN | 133.78 | 0.002151 | Conserved gene of unknown function |
| PGSC0003DMG401003179 | 4.84 | DOWN | 9.69 | 0.0218313 | Conserved gene of unknown function |
| PGSC0003DMG400006309 | 4.84 | DOWN | 5.67 | 0.002151 | DC1 domain containing protein |
| PGSC0003DMG400021683 | 4.84 | DOWN | 4.08 | 0.0466816 | E3 ubiquitin-protein ligase RMA1H1 |
| PGSC0003DMG400017330 | 4.84 | DOWN | 8.13 | 0.002151 | Serine-threonine protein kinase, plant-type |
| PGSC0003DMG400031548 | 4.81 | DOWN | 27.64 | 0.002151 | Conserved gene of unknown function |
| PGSC0003DMG400020334 | 4.81 | DOWN | 9.96 | 0.002151 | Prephenate dehydrogenase |
| PGSC0003DMG400012655 | 4.77 | DOWN | 26.69 | 0.002151 | Conserved gene of unknown function |
| PGSC0003DMG400012753 | 4.77 | DOWN | 23.31 | 0.002151 | Conserved gene of unknown function |
| PGSC0003DMG400024149 | 4.76 | DOWN | 12.3 | 0.002151 | Receptor protein kinase |
| PGSC0003DMG400027185 | 4.75 | DOWN | 77.72 | 0.002151 | Hydrolase |
| PGSC0003DMG400018102 | 4.69 | DOWN | 213.39 | 0.002151 | Calmodulin-binding protein |
| PGSC0003DMG400027431 | 4.69 | DOWN | 24.37 | 0.0466816 | Conserved gene of unknown function |
| PGSC0003DMG400027468 | 4.69 | DOWN | 12.07 | 0.002151 | Leucine-rich repeat family protein |
| PGSC0003DMG400025022 | 4.67 | DOWN | 37.57 | 0.002151 | Avr9/Cf-9 rapidly elicited protein 20 |
| PGSC0003DMG400028984 | 4.66 | DOWN | 8.81 | 0.0435373 | Conserved gene of unknown function |
| PGSC0003DMG401027676 | 4.64 | DOWN | 35.57 | 0.002151 | Conserved gene of unknown function |
| PGSC0003DMG400005969 | 4.64 | DOWN | 25.38 | 0.002151 | Gene of unknown function |
| PGSC0003DMG400012571 | 4.62 | DOWN | 96.22 | 0.002151 | GTP cyclohydrolase II / 3,4-dihydroxy-2-butanone-4-phosphate synthase |
| PGSC0003DMG400025738 | 4.6 | DOWN | 77.76 | 0.002151 | Phytosulfokines |
| PGSC0003DMG400028846 | 4.59 | DOWN | 2.2 | 0.0191534 | Avr9/Cf-9 rapidly elicited protein 132 |
| PGSC0003DMG400024771 | 4.59 | DOWN | 74.76 | 0.002151 | Conserved gene of unknown function |
| PGSC0003DMG400000791 | 4.59 | DOWN | 65.78 | 0.002151 | Photoperiod responsive protein |
| PGSC0003DMG400016140 | 4.58 | DOWN | 174.66 | 0.002151 | Mitochondrial dicarboxylate carrier protein |
| PGSC0003DMG400024438 | 4.51 | DOWN | 3.96 | 0.002151 | Hcr2-p3 |
| PGSC0003DMG402018257 | 4.51 | DOWN | 29.61 | 0.002151 | Leucine-rich repeat-containing protein |
| PGSC0003DMG400013886 | 4.51 | DOWN | 120 | 0.002151 | S-locus-specific glycoprotein S6 |
| PGSC0003DMG400022414 | 4.5 | DOWN | 5.69 | 0.002151 | Fiber expressed protein |
| PGSC0003DMG400018651 | 4.49 | DOWN | 420.15 | 0.002151 | Amino acid transporter |
| PGSC0003DMG400020896 | 4.49 | DOWN | 4.43 | 0.002151 | Glucosyl/glucuronosyl transferases |
| PGSC0003DMG400028339 | 4.49 | DOWN | 12.91 | 0.002151 | Late blight resistance protein |
| PGSC0003DMG400000495 | 4.44 | DOWN | 45.92 | 0.002151 | C2H2L domain class transcription factor |
| 4CL2 | 4.38 | DOWN | 83.88 | 0.002151 | 4-coumarate--CoA ligase 2 |
| PGSC0003DMG400003982 | 4.38 | DOWN | 8.44 | 0.002151 | ATP binding protein |
| PGSC0003DMG400003306 | 4.38 | DOWN | 3.71 | 0.0132528 | Cytochrome P450 |
| ERF4 | 4.36 | DOWN | 139.97 | 0.002151 | Ethylene-responsive transcription factor 3 |
| PGSC0003DMG400019408 | 4.33 | DOWN | 5.66 | 0.030665 | WRKY transcription factor |
| PGSC0003DMG400011039 | 4.31 | DOWN | 850.7 | 0.002151 | Conserved gene of unknown function |
| PGSC0003DMG400017307 | 4.3 | DOWN | 1269.07 | 0.002151 | ATFP4 |
| PGSC0003DMG400003237 | 4.28 | DOWN | 2.34 | 0.002151 | Conserved gene of unknown function |
| PGSC0003DMG400005822 | 4.26 | DOWN | 28.16 | 0.002151 | Potassium channel tetramerization domain-containing protein |
| PGSC0003DMG400019872 | 4.25 | DOWN | 94.1 | 0.002151 | Adenylyl-sulfate reductase |
| PGSC0003DMG400006904 | 4.25 | DOWN | 6.36 | 0.0073051 | Gene of unknown function |
| PGSC0003DMG400028448 | 4.24 | DOWN | 57.64 | 0.002151 | Conserved gene of unknown function |
| PGSC0003DMG400026159 | 4.22 | DOWN | 181.53 | 0.002151 | Ccd1 |
| PGSC0003DMG400011750 | 4.2 | DOWN | 20.13 | 0.002151 | Cytochrome P-450 |
| PGSC0003DMG400011311 | 4.2 | DOWN | 4.36 | 0.0147116 | Epoxide hydrolase 1 |
| PGSC0003DMG400022193 | 4.18 | DOWN | 130.29 | 0.002151 | Pirin |
| PGSC0003DMG402016981 | 4.16 | DOWN | 1.12 | 0.01619 | Bacterial spot disease resistance protein 4 |
| PGSC0003DMG400012974 | 4.16 | DOWN | 32.35 | 0.002151 | Conserved gene of unknown function |
| PGSC0003DMG400034322 | 4.16 | DOWN | 54.45 | 0.002151 | Zinc finger protein |
| PGSC0003DMG400012154 | 4.15 | DOWN | 10.97 | 0.002151 | AP2 domain-containing transcription factor 9 |
| PGSC0003DMG400018045 | 4.15 | DOWN | 7.04 | 0.002151 | Hcr9-OR2B |
| PGSC0003DMG400018115 | 4.14 | DOWN | 28.56 | 0.002151 | ATP binding protein |
| PGSC0003DMG400002014 | 4.13 | DOWN | 8.65 | 0.002151 | Avr9/Cf-9 rapidly elicited protein 76 |
| PGSC0003DMG400047336 | 4.12 | DOWN | 4.98 | 0.0282933 | ACRE 132 |
| PGSC0003DMG401030920 | 4.1 | DOWN | 241.22 | 0.002151 | Cucumber peeling cupredoxin |
| PGSC0003DMG400007152 | 4.09 | DOWN | 154.67 | 0.002151 | UDP-apiose/xylose synthase |
| PGSC0003DMG400038332 | 4.08 | DOWN | 4.7 | 0.002151 | Subtilisin-like protease |
| PGSC0003DMG400023107 | 4.07 | DOWN | 50.51 | 0.002151 | Conserved gene of unknown function |
| PGSC0003DMG402006653 | 4.07 | DOWN | 40.22 | 0.002151 | Conserved gene of unknown function |
| PGSC0003DMG400014791 | 4.07 | DOWN | 11.73 | 0.002151 | Jumonji domain protein |
| PGSC0003DMG400011878 | 4.05 | DOWN | 3.09 | 0.0132528 | Serine-threonine protein kinase, plant-type |
| PGSC0003DMG400011347 | 4.05 | DOWN | 21.49 | 0.002151 | Src2 |
| PGSC0003DMG400000066 | 4.04 | DOWN | 152.89 | 0.002151 | Ethylene-responsive late embryogenesis |
| PGSC0003DMG400015448 | 4.04 | DOWN | 10.44 | 0.0425183 | MAPKKK5 |
| PGSC0003DMG400010870 | 4.04 | DOWN | 180.19 | 0.002151 | NAC domain protein |
| PGSC0003DMG400007613 | 4.03 | DOWN | 217.78 | 0.002151 | Alternative oxidase |
| PGSC0003DMG400041311 | 4.03 | DOWN | 25 | 0.002151 | Conserved gene of unknown function |
| PGSC0003DMG400025621 | 4.02 | DOWN | 26.98 | 0.002151 | NDR1 |
| PGSC0003DMG400011548 | 4.01 | DOWN | 75.97 | 0.002151 | Zinc finger DNA-binding protein |
| PGSC0003DMG400014776 | 4 | DOWN | 43.24 | 0.002151 | Tyramine hydroxycinnamoyl transferase |
| PGSC0003DMG400026821 | 3.99 | DOWN | 260.28 | 0.002151 | Ethylene-responsive transcription factor 4 |
| PGSC0003DMG400021454 | 3.99 | DOWN | 47.88 | 0.002151 | NHL25 (NDR1/HIN1-LIKE 25) |
| PGSC0003DMG400041467 | 3.99 | DOWN | 107.53 | 0.002151 | Zinc-finger protein |
| PGSC0003DMG400001422 | 3.97 | DOWN | 24.38 | 0.002151 | 60S ribosomal protein L34 |
| PGSC0003DMG400031140 | 3.97 | DOWN | 186.24 | 0.002151 | WRKY transcription factor |
| PGSC0003DMG400029207 | 3.97 | DOWN | 872.51 | 0.002151 | WRKY transcription factor 6 |
| CEVI16 | 3.95 | DOWN | 113.41 | 0.002151 | Cationic peroxidase |
| PGSC0003DMG400009955 | 3.93 | DOWN | 32.79 | 0.002151 | Syringolide-induced protein 14-1-1 |
| PGSC0003DMG400022836 | 3.91 | DOWN | 11 | 0.0103049 | Histone H3.2 |
| PGSC0003DMG400013797 | 3.9 | DOWN | 5.73 | 0.002151 | Protein kinase |
| PGSC0003DMG400014213 | 3.89 | DOWN | 11.13 | 0.002151 | ATP binding protein |
| PGSC0003DMG400022340 | 3.89 | DOWN | 7.15 | 0.0282933 | Ccd1 |
| PGSC0003DMG400043394 | 3.89 | DOWN | 93.67 | 0.002151 | Conserved gene of unknown function |
| PGSC0003DMG400019804 | 3.89 | DOWN | 1.67 | 0.0073051 | Disease resistance protein RGA2 |
| PGSC0003DMG400027338 | 3.87 | DOWN | 16.82 | 0.002151 | UDP-glucose:glucosyltransferase |
| PGSC0003DMG400007713 | 3.84 | DOWN | 84.64 | 0.002151 | Conserved gene of unknown function |
| PGSC0003DMG400031547 | 3.84 | DOWN | 8.86 | 0.002151 | Gene of unknown function |
| PGSC0003DMG400019824 | 3.83 | DOWN | 18.32 | 0.002151 | JA-induced WRKY protein |
| PGSC0003DMG400007804 | 3.82 | DOWN | 10.23 | 0.0057258 | Conserved gene of unknown function |
| PGSC0003DMG400027469 | 3.82 | DOWN | 11.44 | 0.002151 | Serine-threonine protein kinase, plant-type |
| PPACS1 | 3.8 | DOWN | 117.25 | 0.002151 | ACC synthase |
| PGSC0003DMG400002327 | 3.8 | DOWN | 9.72 | 0.002151 | Avr9/Cf-9 induced kinase 1 |
| PGSC0003DMG400003664 | 3.8 | DOWN | 56.18 | 0.002151 | Protein-methionine-s-oxide reductase |
| PGSC0003DMG400030394 | 3.79 | DOWN | 51.23 | 0.002151 | Conserved gene of unknown function |
| PGSC0003DMG400005898 | 3.78 | DOWN | 11.55 | 0.0073051 | Harpin-induced family protein |
| PGSC0003DMG400024613 | 3.78 | DOWN | 33.8 | 0.002151 | Protein ASC1 |
| PPS6 | 3.78 | DOWN | 147.71 | 0.002151 | Short chain dehydrogenase |
| PGSC0003DMG402030736 | 3.78 | DOWN | 170.36 | 0.002151 | Zinc finger family protein |
| PGSC0003DMG400014589 | 3.77 | DOWN | 1.61 | 0.0457077 | Calmodulin binding protein |
| PGSC0003DMG400004952 | 3.73 | DOWN | 100.44 | 0.002151 | Branched-chain amino acid aminotransferase |
| PGSC0003DMG400023731 | 3.73 | DOWN | 36.67 | 0.002151 | Glycosyltransferase |
| PGSC0003DMG400029237 | 3.72 | DOWN | 191.42 | 0.002151 | Gene of unknown function |
| PGSC0003DMG400000334 | 3.71 | DOWN | 6.08 | 0.0118276 | Calmodulin binding protein |
| PGSC0003DMG400008984 | 3.69 | DOWN | 8.78 | 0.002151 | Basic 7S globulin 2 small subunit |
| PGSC0003DMG400021114 | 3.69 | DOWN | 12.76 | 0.002151 | Ethylene-responsive nuclear protein |
| PGSC0003DMG400010296 | 3.66 | DOWN | 5.47 | 0.0088917 | Patellin-4 |
| PGSC0003DMG400028520 | 3.66 | DOWN | 488.21 | 0.002151 | WRKY transcription factor 1 |
| PGSC0003DMG400033904 | 3.61 | DOWN | 10.35 | 0.0366624 | SANT/MYB domain protein |
| PGSC0003DMG400008337 | 3.6 | DOWN | 66.41 | 0.002151 | MYB21 |
| PGSC0003DMG400027692 | 3.59 | DOWN | 379.61 | 0.002151 | Calcium-binding protein CAST |
| PGSC0003DMG400030808 | 3.58 | DOWN | 14.13 | 0.002151 | Phi-1 protein |
| PGSC0003DMG400003948 | 3.58 | DOWN | 190.94 | 0.002151 | Stress-associated protein 6 |
| PGSC0003DMG400003084 | 3.58 | DOWN | 65.44 | 0.002151 | Two-component response regulator ARR8 |
| PGSC0003DMG400001659 | 3.57 | DOWN | 113.67 | 0.002151 | DNA binding protein |
| PGSC0003DMG400006652 | 3.57 | DOWN | 6.96 | 0.002151 | Hcr9-OR2A |
| PGSC0003DMG400005992 | 3.54 | DOWN | 141.45 | 0.002151 | Conserved gene of unknown function |
| PGSC0003DMG400027470 | 3.53 | DOWN | 12.46 | 0.0057258 | ATP binding protein |
| PGSC0003DMG400028097 | 3.53 | DOWN | 52.56 | 0.002151 | Short-chain type alcohol dehydrogenase |
| PGSC0003DMG400016006 | 3.52 | DOWN | 433.4 | 0.002151 | Pti4 |
| PGSC0003DMG400014200 | 3.49 | DOWN | 11.43 | 0.002151 | Flotillin-1 |
| PGSC0003DMG400014367 | 3.49 | DOWN | 12.71 | 0.044629 | Gene of unknown function |
| STWRKY8 | 3.48 | DOWN | 723.05 | 0.002151 | Double WRKY type transfactor |
| PGSC0003DMG400016529 | 3.48 | DOWN | 96.36 | 0.002151 | Uncharacterized Cys-rich domain |
| PGSC0003DMG400003999 | 3.46 | DOWN | 11.51 | 0.002151 | ATP binding protein |
| PGSC0003DMG400017936 | 3.45 | DOWN | 2744.72 | 0.002151 | Late embryogenis abundant protein 5 |
| PGSC0003DMG400006000 | 3.45 | DOWN | 5.75 | 0.0295229 | Receptor kinase |
| PGSC0003DMG400016011 | 3.44 | DOWN | 17.58 | 0.002151 | Calmodulin |
| PGSC0003DMG400030113 | 3.44 | DOWN | 9.86 | 0.002151 | Leucine-rich repeat receptor kinase |
| PGSC0003DMG400024749 | 3.44 | DOWN | 94.33 | 0.002151 | Rapid alkalinization factor 2 |
| PGSC0003DMG400030995 | 3.43 | DOWN | 2.18 | 0.0282933 | Polygalacturonase-1 non-catalytic subunit beta |
| LOX1.5 | 3.4 | DOWN | 12.03 | 0.002151 | Lipoxygenase |
| PGSC0003DMG400024323 | 3.39 | DOWN | 94.78 | 0.002151 | Conserved gene of unknown function |
| ERF3 | 3.39 | DOWN | 207.57 | 0.002151 | Ethylene response factor 3 |
| PGSC0003DMG400027570 | 3.37 | DOWN | 51.44 | 0.002151 | Gene of unknown function |
| PGSC0003DMG400027124 | 3.37 | DOWN | 24.78 | 0.002151 | Serine/threonine-protein kinase CCR3 |
| PGSC0003DMG400033334 | 3.36 | DOWN | 15.59 | 0.002151 | Bacterial spot disease resistance protein 4 |
| AAP2 | 3.34 | DOWN | 412.39 | 0.002151 | Amino acid transporter |
| PAT | 3.34 | DOWN | 571.11 | 0.002151 | Polyubiquitin |
| PGSC0003DMG400017278 | 3.33 | DOWN | 26.6 | 0.002151 | Receptor-like kinase |
| PGSC0003DMG400022954 | 3.32 | DOWN | 5.35 | 0.002151 | Glutamate receptor 2 plant |
| PGSC0003DMG400011038 | 3.32 | DOWN | 11.52 | 0.002151 | Short chain alcohol dehydrogenase |
| PGSC0003DMG400017190 | 3.31 | DOWN | 34.71 | 0.0377582 | 1-aminocyclopropane-1-carboxylate oxidase homolog |
| PGSC0003DMG400033632 | 3.3 | DOWN | 11.58 | 0.002151 | Cf-2.2 |
| PGSC0003DMG400032792 | 3.29 | DOWN | 23.41 | 0.002151 | Calmodulin-binding protein |
| PGSC0003DMG400013356 | 3.29 | DOWN | 40.58 | 0.002151 | GRAS1 |
| PGSC0003DMG400007376 | 3.29 | DOWN | 2.56 | 0.0478271 | Ubiquitin-protein ligase |
| PGSC0003DMG400017062 | 3.28 | DOWN | 17.34 | 0.002151 | Disease resistance protein RGA1 |
| PGSC0003DMG400000555 | 3.28 | DOWN | 259.44 | 0.002151 | Nam 1 |
| PGSC0003DMG400005837 | 3.24 | DOWN | 15.64 | 0.002151 | AP2/ERF domain-containing transcription factor |
| PGSC0003DMG400001333 | 3.24 | DOWN | 161.55 | 0.002151 | Calcium ion binding protein |
| PGSC0003DMG400014770 | 3.24 | DOWN | 95.11 | 0.002151 | N-hydroxycinnamoyl-CoA:tyramine N-hydroxycinnamoyl transferase THT7-8 |
| CKX3 | 3.23 | DOWN | 10.16 | 0.002151 | Cytokinin oxidase/dehydrogenase |
| PGSC0003DMG400008538 | 3.23 | DOWN | 25.01 | 0.002151 | Dehydration-responsive element binding protein 3 |
| PGSC0003DMG400007947 | 3.23 | DOWN | 167.93 | 0.002151 | WRKY transcription factor 2 |
| PGSC0003DMG400024661 | 3.22 | DOWN | 8.93 | 0.002151 | Brassinosteroid LRR receptor kinase |
| PGSC0003DMG400008564 | 3.22 | DOWN | 431.3 | 0.002151 | Chlorophyll a-b binding protein 13, chloroplastic |
| PGSC0003DMG400019413 | 3.22 | DOWN | 24.14 | 0.002151 | Conserved gene of unknown function |
| PGSC0003DMG400029151 | 3.19 | DOWN | 61.9 | 0.002151 | Conserved gene of unknown function |
| PGSC0003DMG400004940 | 3.18 | DOWN | 45.72 | 0.002151 | Conserved gene of unknown function |
| PGSC0003DMG400012120 | 3.17 | DOWN | 26.99 | 0.002151 | Conserved gene of unknown function |
| PGSC0003DMG400016663 | 3.17 | DOWN | 12.6 | 0.0073051 | Conserved gene of unknown function |
| PGSC0003DMG400000957 | 3.16 | DOWN | 3.53 | 0.044629 | ATP binding protein |
| PGSC0003DMG400007915 | 3.16 | DOWN | 7.88 | 0.0103049 | Conserved gene of unknown function |
| PGSC0003DMG402024074 | 3.16 | DOWN | 52.37 | 0.002151 | Conserved gene of unknown function |
| PGSC0003DMG401011550 | 3.13 | DOWN | 70.68 | 0.002151 | Conserved gene of unknown function |
| PGSC0003DMG402021549 | 3.12 | DOWN | 277.16 | 0.002151 | Phenylalanine ammonia-lyase |
| PGSC0003DMG400003626 | 3.11 | DOWN | 77.94 | 0.002151 | Lactoylglutathione lyase |
| PGSC0003DMG400011169 | 3.11 | DOWN | 1394.56 | 0.002151 | Senescence-associated protein |
| PGSC0003DMG400025931 | 3.09 | DOWN | 68.32 | 0.0354738 | GTP-binding protein alpha subunit, gna |
| PGSC0003DMG400006022 | 3.09 | DOWN | 2.5 | 0.0243888 | Kinase |
| PGSC0003DMG400001444 | 3.08 | DOWN | 48.35 | 0.002151 | Isoform 2 of Probable galacturonosyltransferase 13 |
| PGSC0003DMG401029843 | 3.06 | DOWN | 66.47 | 0.002151 | Glucosyltransferase |
| PGSC0003DMG400022448 | 3.05 | DOWN | 18.48 | 0.0204365 | Conserved gene of unknown function |
| PGSC0003DMG400046804 | 3.05 | DOWN | 139.1 | 0.002151 | Mitochondrial substrate carrier family protein isoform 1 |
| PGSC0003DMG400009113 | 3.04 | DOWN | 344.63 | 0.002151 | Gene of unknown function |
| PGSC0003DMG400017358 | 3.03 | DOWN | 22.53 | 0.0040151 | Receptor-like kinase |
| PGSC0003DMG400015310 | 3.02 | DOWN | 8.14 | 0.0191534 | Calmodulin-binding protein |
| PGSC0003DMG400030364 | 3.01 | DOWN | 43.86 | 0.002151 | Avr9/Cf-9 rapidly elicited protein 284 |
| PGSC0003DMG401021043 | 3 | DOWN | 3.27 | 0.0040151 | NBS-LRR resistance protein |
| PGSC0003DMG400029028 | 2.99 | DOWN | 8.95 | 0.0414827 | 2-oxoglutarate-dependent dioxygenase |
| PGSC0003DMG400030102 | 2.99 | DOWN | 63.94 | 0.0073051 | Conserved gene of unknown function |
| PGSC0003DMG400027174 | 2.99 | DOWN | 28.72 | 0.002151 | Protein phosphatase 2c |
| PGSC0003DMG400030509 | 2.99 | DOWN | 23.02 | 0.002151 | Ribose-5-phosphate isomerase |
| PGSC0003DMG400022543 | 2.98 | DOWN | 11.95 | 0.0073051 | ATP binding protein |
| PGSC0003DMG400002908 | 2.98 | DOWN | 78.84 | 0.002151 | Conserved gene of unknown function |
| RBOHC | 2.98 | DOWN | 619.63 | 0.002151 | Respiratory burst oxidase homolog protein C |
| PGSC0003DMG400027951 | 2.96 | DOWN | 4.15 | 0.0342702 | Conserved gene of unknown function |
| PGSC0003DMG400018930 | 2.96 | DOWN | 17.85 | 0.002151 | Proteinase inhibitor I4, serpin |
| PGSC0003DMG400006217 | 2.95 | DOWN | 173.15 | 0.002151 | C2 domain-containing protein |
| PGSC0003DMG400016257 | 2.95 | DOWN | 140.78 | 0.002151 | Conserved gene of unknown function |
| PGSC0003DMG400010032 | 2.95 | DOWN | 63.97 | 0.002151 | WAK-like kinase |
| PGSC0003DMG400005245 | 2.94 | DOWN | 68.69 | 0.002151 | Caspase |
| PGSC0003DMG400030618 | 2.94 | DOWN | 47.21 | 0.002151 | Conserved gene of unknown function |
| PGSC0003DMG400003105 | 2.94 | DOWN | 23.41 | 0.002151 | Glutamyl-tRNA reductase |
| PGSC0003DMG400009177 | 2.94 | DOWN | 16.21 | 0.002151 | Pectinesterase 3 |
| NAC | 2.93 | DOWN | 231.28 | 0.002151 | NAC domain protein |
| PGSC0003DMG400029520 | 2.93 | DOWN | 50.48 | 0.002151 | Serine/threonine-protein kinase PBS1 |
| PGSC0003DMG400007122 | 2.92 | DOWN | 200.17 | 0.002151 | Arogenate dehydratase |
| PGSC0003DMG400023028 | 2.92 | DOWN | 28.35 | 0.002151 | Calcium-binding protein |
| PGSC0003DMG400014555 | 2.92 | DOWN | 10.09 | 0.0435373 | Conserved gene of unknown function |
| LHCB1-2 | 2.91 | DOWN | 72.13 | 0.002151 | Chlorophyll a/b binding protein |
| PGSC0003DMG400019725 | 2.91 | DOWN | 43.05 | 0.0040151 | Conserved gene of unknown function |
| PGSC0003DMG400013405 | 2.91 | DOWN | 25.97 | 0.002151 | THM18 protein |
| PGSC0003DMG400003369 | 2.9 | DOWN | 362.52 | 0.002151 | Conserved gene of unknown function |
| PGSC0003DMG400025267 | 2.9 | DOWN | 123.98 | 0.002151 | Conserved gene of unknown function |
| PGSC0003DMG400007634 | 2.9 | DOWN | 22.71 | 0.002151 | Serine-threonine protein kinase, plant-type |
| NAC2 | 2.89 | DOWN | 378.76 | 0.002151 | NAC domain protein NAC2 |
| PGSC0003DMG400013803 | 2.89 | DOWN | 30.38 | 0.002151 | Serine-threonine protein kinase, plant-type |
| PGSC0003DMG400021948 | 2.88 | DOWN | 3.09 | 0.0040151 | Cc-nbs-lrr resistance protein |
| PGSC0003DMG400033651 | 2.88 | DOWN | 6.04 | 0.002151 | Nbs-lrr resistance protein |
| PGSC0003DMG400027047 | 2.88 | DOWN | 116.87 | 0.002151 | UPF0497 membrane protein |
| PGSC0003DMG400022063 | 2.88 | DOWN | 26.45 | 0.002151 | WRKY1a transcription factor |
| PGSC0003DMG400017976 | 2.87 | DOWN | 34.36 | 0.002151 | Reticuline oxidase |
| PGSC0003DMG400009946 | 2.86 | DOWN | 113.05 | 0.002151 | Conserved gene of unknown function |
| PGSC0003DMG400004703 | 2.85 | DOWN | 11.66 | 0.0204365 | Conserved gene of unknown function |
| PGSC0003DMG400002166 | 2.85 | DOWN | 9.27 | 0.0103049 | Glutathione S-transferase |
| PGSC0003DMG400004301 | 2.84 | DOWN | 89.99 | 0.002151 | Chlorophyll a,b binding protein type I |
| PGSC0003DMG400030144 | 2.84 | DOWN | 7.55 | 0.0040151 | Kinase |
| PGSC0003DMG400017747 | 2.82 | DOWN | 28.3 | 0.002151 | Conserved gene of unknown function |
| PGSC0003DMG400012318 | 2.82 | DOWN | 24.25 | 0.002151 | WRKY transcription factor |
| PGSC0003DMG400025931 | 2.81 | DOWN | 62.83 | 0.002151 | GTP-binding protein alpha subunit, gna |
| PGSC0003DMG400011872 | 2.79 | DOWN | 73.27 | 0.002151 | Glucosyltransferase |
| PGSC0003DMG400014894 | 2.79 | DOWN | 10.04 | 0.0103049 | Membrane protein |
| PGSC0003DMG400018752 | 2.78 | DOWN | 1.6 | 0.0401819 | Serine-threonine protein kinase, plant-type |
| PGSC0003DMG400002910 | 2.77 | DOWN | 22.28 | 0.002151 | Cf-2.1 |
| PGSC0003DMG400029415 | 2.76 | DOWN | 6.59 | 0.0103049 | Nematode resistance protein |
| PGSC0003DMG400033667 | 2.76 | DOWN | 13.59 | 0.002151 | Serine-threonine protein kinase, plant-type |
| PGSC0003DMG401005729 | 2.75 | DOWN | 10.4 | 0.0377582 | Wall-associated kinase |
| PGSC0003DMG400028287 | 2.74 | DOWN | 170.68 | 0.002151 | S-adenosylmethionine synthase |
| PGSC0003DMG400017843 | 2.73 | DOWN | 34.46 | 0.002151 | Conserved gene of unknown function |
| PGSC0003DMG400038970 | 2.73 | DOWN | 37.16 | 0.0073051 | Conserved gene of unknown function |
| PGSC0003DMG400029885 | 2.73 | DOWN | 81.52 | 0.002151 | Kinase |
| PGSC0003DMG400009181 | 2.73 | DOWN | 12.1 | 0.002151 | Leucine-rich repeat receptor protein kinase EXS |
| PGSC0003DMG400026332 | 2.73 | DOWN | 96.75 | 0.002151 | NAC-domain protein |
| PGSC0003DMG400019726 | 2.72 | DOWN | 11.09 | 0.002151 | Conserved gene of unknown function |
| DREB1 | 2.71 | DOWN | 37.46 | 0.002151 | AP2/ERF domain-containing transcription factor |
| PGSC0003DMG400021533 | 2.71 | DOWN | 46.32 | 0.002151 | Hspc200 |
| PGSC0003DMG400004409 | 2.71 | DOWN | 441.65 | 0.002151 | Protein kinase 1b |
| PGSC0003DMG400027945 | 2.71 | DOWN | 191.1 | 0.002151 | RIN4c protein |
| PGSC0003DMG400003618 | 2.7 | DOWN | 16.37 | 0.0088917 | Conserved gene of unknown function |
| PGSC0003DMG400009399 | 2.69 | DOWN | 50.21 | 0.0040151 | Glutaredoxin-C9 |
| PGSC0003DMG400000311 | 2.69 | DOWN | 29.39 | 0.002151 | Protein cbxX, chromosomal |
| PGSC0003DMG400012848 | 2.67 | DOWN | 16.78 | 0.0295229 | AO (L-ASPARTATE OXIDASE); L-aspartate oxidase |
| PGSC0003DMG400015552 | 2.67 | DOWN | 6.5 | 0.0088917 | Beta-ketoacyl-coa synthase family protein |
| PGSC0003DMG400024486 | 2.67 | DOWN | 150.16 | 0.002151 | Conserved gene of unknown function |
| PGSC0003DMG400028751 | 2.67 | DOWN | 13.22 | 0.0342702 | Conserved gene of unknown function |
| PGSC0003DMG400029544 | 2.67 | DOWN | 35.58 | 0.0318847 | Ring finger protein |
| PGSC0003DMG400024912 | 2.66 | DOWN | 16.98 | 0.002151 | Conserved gene of unknown function |
| PGSC0003DMG400032272 | 2.66 | DOWN | 72.98 | 0.002151 | N-acetyltransferase |
| PGSC0003DMG400015835 | 2.65 | DOWN | 4.7 | 0.0489636 | Receptor kinase |
| PGSC0003DMG400025441 | 2.64 | DOWN | 27.09 | 0.0271321 | Conserved gene of unknown function |
| PGSC0003DMG400017334 | 2.64 | DOWN | 36.01 | 0.002151 | Heat shock factor protein |
| PGSC0003DMG400028222 | 2.63 | DOWN | 54.83 | 0.002151 | KED |
| PGSC0003DMG400012296 | 2.62 | DOWN | 77.02 | 0.002151 | Avr9/Cf-9 rapidly elicited protein 194 |
| PGSC0003DMG400040729 | 2.62 | DOWN | 20.23 | 0.002151 | FERONIA receptor-like kinase |
| PGSC0003DMG400029519 | 2.62 | DOWN | 49.69 | 0.002151 | High mobility group family |
| PGSC0003DMG400007603 | 2.62 | DOWN | 268.06 | 0.002151 | I-box binding factor |
| PGSC0003DMG400009238 | 2.62 | DOWN | 81.67 | 0.002151 | Leucine-rich repeat family protein |
| PGSC0003DMG400012798 | 2.6 | DOWN | 43.07 | 0.0073051 | Conserved gene of unknown function |
| PGSC0003DMG400006729 | 2.59 | DOWN | 6.04 | 0.0132528 | ATP binding protein |
| PGSC0003DMG400011222 | 2.58 | DOWN | 225.17 | 0.002151 | UDP-glucuronate 5-epimerase |
| PGSC0003DMG400010724 | 2.57 | DOWN | 79.1 | 0.002151 | ERF transcription factor 4 |
| PGSC0003DMG400012110 | 2.56 | DOWN | 326.51 | 0.002151 | Phosphatidylcholine transfer protein |
| PGSC0003DMG400029544 | 2.56 | DOWN | 32.93 | 0.0103049 | Ring finger protein |
| PGSC0003DMG400002596 | 2.55 | DOWN | 552.2 | 0.002151 | Developmental protein |
| PGSC0003DMG400020099 | 2.55 | DOWN | 458.95 | 0.002151 | RPM1 interacting protein 4 transcript 2 |
| PGSC0003DMG400032118 | 2.54 | DOWN | 46.18 | 0.002151 | CDC48-interacting UBX-domain protein |
| PGSC0003DMG400029894 | 2.54 | DOWN | 24.79 | 0.002151 | Cytochrome P450 hydroxylase |
| PGSC0003DMG402028569 | 2.54 | DOWN | 11.16 | 0.0088917 | Phosphoglycerate/bisphosphoglycerate mutase |
| PGSC0003DMG400002153 | 2.54 | DOWN | 131.68 | 0.002151 | Zinc finger protein |
| PGSC0003DMG400018515 | 2.52 | DOWN | 162.93 | 0.002151 | Conserved gene of unknown function |
| PGSC0003DMG400010759 | 2.52 | DOWN | 128.77 | 0.002151 | Gene of unknown function |
| PGSC0003DMG400013180 | 2.52 | DOWN | 20.06 | 0.0330512 | Pentatricopeptide repeat-containing protein |
| PGSC0003DMG400001708 | 2.52 | DOWN | 35.07 | 0.0040151 | RING-H2 finger protein ATL1Q |
| PGSC0003DMG400005597 | 2.51 | DOWN | 118.83 | 0.002151 | MTD1 |
| PGSC0003DMG402016495 | 2.51 | DOWN | 17.15 | 0.002151 | Stem 28 kDa glycoprotein |
| PGSC0003DMG400031755 | 2.5 | DOWN | 39.59 | 0.002151 | Optic atrophy 3 protein |
| AOX | 2.49 | DOWN | 231.34 | 0.002151 | Alternative oxidase |
| PGSC0003DMG400041458 | 2.49 | DOWN | 2.93 | 0.0435373 | Leucine-rich repeat containing protein |
| PGSC0003DMG400004158 | 2.48 | DOWN | 14.12 | 0.0147116 | Ca2+ antiporter/cation exchanger |
| PGSC0003DMG400035478 | 2.48 | DOWN | 478.85 | 0.002151 | Conserved gene of unknown function |
| PGSC0003DMG400001302 | 2.48 | DOWN | 80.7 | 0.002151 | CW14 protein |
| PGSC0003DMG400006814 | 2.47 | DOWN | 576.84 | 0.002151 | AN1-like transcription factor |
| PGSC0003DMG400001756 | 2.46 | DOWN | 36.55 | 0.0204365 | TIR/NBS/LRR protein |
| PGSC0003DMG400001448 | 2.46 | DOWN | 118.08 | 0.002151 | UDP-glucose dehydrogenase 2 |
| PGSC0003DMG400022633 | 2.44 | DOWN | 23.34 | 0.002151 | Ubiquitin-protein ligase |
| PGSC0003DMG400009686 | 2.43 | DOWN | 9.58 | 0.0103049 | Bacterial spot disease resistance protein 4 |
| PGSC0003DMG404026432 | 2.43 | DOWN | 59.59 | 0.0088917 | Tir-nbs-lrr resistance protein |
| PGSC0003DMG400019432 | 2.42 | DOWN | 72.27 | 0.002151 | Calcium ion binding protein |
| PGSC0003DMG400008149 | 2.42 | DOWN | 305.76 | 0.002151 | Calcium-dependent protein kinase 4 |
| PGSC0003DMG400018184 | 2.42 | DOWN | 21.61 | 0.002151 | Carbohydrate binding protein |
| PGSC0003DMG400018457 | 2.42 | DOWN | 17.64 | 0.002151 | Serine-threonine protein kinase, plant-type |
| PGSC0003DMG400009530 | 2.42 | DOWN | 117.35 | 0.002151 | WRKY transcription factor 3 |
| PGSC0003DMG400001932 | 2.41 | DOWN | 205.92 | 0.0040151 | 6-phosphogluconate dehydrogenase, decarboxylating |
| PGSC0003DMG400019721 | 2.41 | DOWN | 53.3 | 0.002151 | Receptor serine/threonine kinase |
| PGSC0003DMG400030234 | 2.41 | DOWN | 181.26 | 0.002151 | SAUR family protein |
| PGSC0003DMG402020367 | 2.39 | DOWN | 10.7 | 0.002151 | Conserved gene of unknown function |
| PGSC0003DMG400025625 | 2.39 | DOWN | 20.76 | 0.0218313 | Nutrient reservoir |
| PGSC0003DMG400026230 | 2.39 | DOWN | 12.99 | 0.002151 | Pentatricopeptide repeat-containing protein |
| PGSC0003DMG400021555 | 2.39 | DOWN | 20.18 | 0.002151 | UDP-glucosyltransferase |
| PGSC0003DMG400000711 | 2.38 | DOWN | 9.68 | 0.0271321 | Basic helix-loop-helix protein BHLH7 |
| PGSC0003DMG400001081 | 2.38 | DOWN | 8.07 | 0.002151 | NBS-LRR protein |
| PGSC0003DMG400024055 | 2.38 | DOWN | 25.68 | 0.002151 | Nematode resistance |
| PGSC0003DMG401006653 | 2.37 | DOWN | 7.74 | 0.0499455 | Disease resistance protein |
| PGSC0003DMG400014094 | 2.36 | DOWN | 90.11 | 0.0132528 | 2,4-dienoyl-CoA reductase |
| PGSC0003DMG400019618 | 2.36 | DOWN | 46.81 | 0.002151 | CCR4-associated factor |
| PGSC0003DMG400015574 | 2.36 | DOWN | 56.71 | 0.002151 | Conserved gene of unknown function |
| PGSC0003DMG400026401 | 2.36 | DOWN | 46.04 | 0.002151 | Delta(7)-sterol-C5(6)-desaturase |
| PGSC0003DMG400027530 | 2.36 | DOWN | 34.36 | 0.002151 | Gene of unknown function |
| PGSC0003DMG400023814 | 2.36 | DOWN | 67.23 | 0.002151 | Kinase family protein |
| PGSC0003DMG400011624 | 2.35 | DOWN | 94.62 | 0.002151 | Conserved gene of unknown function |
| PGSC0003DMG400020505 | 2.35 | DOWN | 523.6 | 0.002151 | Photosystem I reaction center subunit X psaK |
| PGSC0003DMG400008941 | 2.34 | DOWN | 9.3 | 0.0499455 | Conserved gene of unknown function |
| PGSC0003DMG400041071 | 2.34 | DOWN | 6.25 | 0.0057258 | Kinase |
| PGSC0003DMG400026593 | 2.34 | DOWN | 17.2 | 0.002151 | Ubiquitin-protein ligase |
| PGSC0003DMG400025266 | 2.32 | DOWN | 714.78 | 0.002151 | Conserved gene of unknown function |
| PGSC0003DMG401010796 | 2.32 | DOWN | 11.16 | 0.002151 | Conserved gene of unknown function |
| PGSC0003DMG400005762 | 2.32 | DOWN | 99.49 | 0.002151 | Gene of unknown function |
| PGSC0003DMG400028390 | 2.32 | DOWN | 17.14 | 0.0147116 | Gene of unknown function |
| PGSC0003DMG400005538 | 2.32 | DOWN | 12.58 | 0.002151 | Hcr2-0A |
| PGSC0003DMG400029645 | 2.32 | DOWN | 9.34 | 0.0218313 | Pectase lyase |
| PGSC0003DMG400003566 | 2.31 | DOWN | 27.76 | 0.002151 | ARO1 2 |
| PGSC0003DMG400037697 | 2.31 | DOWN | 188.77 | 0.002151 | AT14A |
| PGSC0003DMG400026433 | 2.31 | DOWN | 12.78 | 0.002151 | ATP binding protein |
| PGSC0003DMG400021421 | 2.31 | DOWN | 4.45 | 0.0118276 | Calcium-transporting ATPase 2, plasma membrane-type |
| PGSC0003DMG400007124 | 2.31 | DOWN | 10.13 | 0.0389556 | Conserved gene of unknown function |
| PGSC0003DMG401019715 | 2.31 | DOWN | 20.17 | 0.0499455 | Conserved gene of unknown function |
| PGSC0003DMG400024853 | 2.31 | DOWN | 243.31 | 0.002151 | GRAS6 |
| PGSC0003DMG400028611 | 2.31 | DOWN | 61.41 | 0.002151 | Peroxisomal membrane protein pmp34 |
| PGSC0003DMG400025126 | 2.31 | DOWN | 59.51 | 0.002151 | Phospholipid-transporting ATPase 9 |
| PGSC0003DMG400007865 | 2.31 | DOWN | 21.08 | 0.002151 | Sugar transporter |
| HMG2 | 2.3 | DOWN | 44.12 | 0.002151 | 3-hydroxy-3-methylglutaryl coenzyme A reductase |
| PGSC0003DMG400017106 | 2.3 | DOWN | 12.71 | 0.002151 | ATP binding protein |
| PGSC0003DMG400022562 | 2.3 | DOWN | 181.97 | 0.002151 | Calcium-dependent protein kinase |
| PGSC0003DMG400028672 | 2.3 | DOWN | 21.8 | 0.0271321 | Protein MKS1 |
| PGSC0003DMG400020618 | 2.3 | DOWN | 119.6 | 0.002151 | Tropinone reductase homolog |
| PGSC0003DMG400007375 | 2.29 | DOWN | 770.37 | 0.002151 | Chlorophyll a/b-binding protein PS II-Type I |
| PGSC0003DMG400030680 | 2.29 | DOWN | 42.69 | 0.002151 | Conserved gene of unknown function |
| PGSC0003DMG400025379 | 2.29 | DOWN | 209.33 | 0.002151 | Gene of unknown function |
| PGSC0003DMG400014403 | 2.28 | DOWN | 31.88 | 0.002151 | Formin 5 |
| PGSC0003DMG400014566 | 2.28 | DOWN | 24.39 | 0.002151 | Transcription factor |
| PGSC0003DMG400009902 | 2.28 | DOWN | 46.7 | 0.0147116 | Ubiquitin-protein ligase |
| PGSC0003DMG403011204 | 2.27 | DOWN | 85.73 | 0.002151 | Conserved gene of unknown function |
| PGSC0003DMG400002156 | 2.26 | DOWN | 32.08 | 0.0040151 | C-4 sterol methyl oxidase 2 |
| PGSC0003DMG400008163 | 2.26 | DOWN | 274.59 | 0.002151 | Calcium-binding allergen Ole e |
| PGSC0003DMG400007787 | 2.26 | DOWN | 61.93 | 0.002151 | Chlorophyll a-b binding protein 8, chloroplastic |
| PGSC0003DMG400001895 | 2.26 | DOWN | 39.52 | 0.002151 | Conserved gene of unknown function |
| PGSC0003DMG400029898 | 2.26 | DOWN | 287.36 | 0.002151 | Conserved gene of unknown function |
| PGSC0003DMG400032160 | 2.26 | DOWN | 868.45 | 0.0073051 | Conserved gene of unknown function |
| PGSC0003DMG400001049 | 2.26 | DOWN | 18.76 | 0.002151 | Gene of unknown function |
| PGSC0003DMG400021993 | 2.25 | DOWN | 11.99 | 0.0103049 | BRASSINOSTEROID INSENSITIVE 1-associated receptor kinase 1 |
| PGSC0003DMG400018145 | 2.25 | DOWN | 40.57 | 0.0040151 | Enzyme inhibitor |
| PGSC0003DMG400016880 | 2.25 | DOWN | 14.3 | 0.0147116 | Gene of unknown function |
| PGSC0003DMG400012613 | 2.25 | DOWN | 53.08 | 0.002151 | RING-finger protein |
| PGSC0003DMG400022888 | 2.25 | DOWN | 72.85 | 0.002151 | Salt responsive protein 1 |
| PGSC0003DMG400032563 | 2.24 | DOWN | 10.63 | 0.0389556 | Conserved gene of unknown function |
| PGSC0003DMG403020240 | 2.24 | DOWN | 20.29 | 0.030665 | Glycerophosphodiester phosphodiesterase |
| PGSC0003DMG400006842 | 2.24 | DOWN | 3.43 | 0.0318847 | SBT4B protein |
| PGSC0003DMG402006771 | 2.23 | DOWN | 13.52 | 0.0103049 | Conserved gene of unknown function |
| PGSC0003DMG400008849 | 2.23 | DOWN | 26.72 | 0.0073051 | Monovalent cation:proton antiporter |
| PGSC0003DMG400012020 | 2.23 | DOWN | 555.86 | 0.002151 | Pectin methlyesterase inhibitor protein 1 |
| PGSC0003DMG400025972 | 2.22 | DOWN | 63.94 | 0.0040151 | 14 kDa proline-rich protein DC2.15 |
| PGSC0003DMG400030661 | 2.22 | DOWN | 47.75 | 0.002151 | ATP binding protein |
| PGSC0003DMG400014210 | 2.22 | DOWN | 584.21 | 0.002151 | DnaJ protein |
| PGSC0003DMG400025756 | 2.22 | DOWN | 38.55 | 0.002151 | Protein phosphatase 2c |
| PGSC0003DMG400024491 | 2.22 | DOWN | 5.07 | 0.0377582 | Spotted leaf protein |
| PGSC0003DMG400030698 | 2.22 | DOWN | 62.19 | 0.002151 | Stress induced protein |
| PGSC0003DMG400021213 | 2.21 | DOWN | 91.56 | 0.002151 | Argonaute protein group |
| PGSC0003DMG400009919 | 2.21 | DOWN | 22.3 | 0.0282933 | Zinc finger family protein |
| PGSC0003DMG400003822 | 2.2 | DOWN | 7.97 | 0.002151 | Cellulose synthase |
| PGSC0003DMG402016888 | 2.19 | DOWN | 25.65 | 0.002151 | 1-acyl-sn-glycerol-3-phosphate acyltransferase 4 |
| PGSC0003DMG400014095 | 2.19 | DOWN | 1369.42 | 0.0040151 | 2,4-dienoyl-CoA reductase |
| PGSC0003DMG400014098 | 2.19 | DOWN | 34.4 | 0.002151 | Actin binding protein |
| PGSC0003DMG402022984 | 2.19 | DOWN | 49.13 | 0.0204365 | Conserved gene of unknown function |
| PGSC0003DMG400029701 | 2.19 | DOWN | 45.72 | 0.002151 | DNA-binding WRKY; VQ |
| PGSC0003DMG400020365 | 2.18 | DOWN | 93.36 | 0.002151 | Conserved gene of unknown function |
| PGSC0003DMG400029159 | 2.18 | DOWN | 35.73 | 0.0366624 | Conserved gene of unknown function |
| PGSC0003DMG400006454 | 2.17 | DOWN | 23.66 | 0.0132528 | Conserved gene of unknown function |
| PGSC0003DMG400024260 | 2.17 | DOWN | 20.58 | 0.002151 | Receptor protein kinase zmpk1 |
| PGSC0003DMG400000730 | 2.17 | DOWN | 124.63 | 0.0057258 | Transcription factor |
| PGSC0003DMG400004804 | 2.16 | DOWN | 13.28 | 0.002151 | Conserved gene of unknown function |
| PGSC0003DMG400011336 | 2.16 | DOWN | 372.89 | 0.002151 | Hypoxia-responsive family protein |
| PGSC0003DMG400017223 | 2.16 | DOWN | 65.91 | 0.002151 | Protein 2 |
| PGSC0003DMG400021131 | 2.15 | DOWN | 97.53 | 0.0040151 | AAA ATPase |
| PGSC0003DMG400003115 | 2.15 | DOWN | 63.71 | 0.0040151 | Chloroplast methionine sulfoxide reductase B2 |
| PGSC0003DMG400037159 | 2.15 | DOWN | 6.69 | 0.0271321 | Leucine-rich repeat containing protein |
| PGSC0003DMG400004458 | 2.15 | DOWN | 31.7 | 0.0057258 | Light-harvesting complex I protein Lhca5 |
| PGSC0003DMG400003383 | 2.15 | DOWN | 37.15 | 0.0103049 | Protein SUR2 |
| 28S_rRNA | 2.14 | DOWN | 88.92 | 0.002151 | 28S_rRNA |
| PGSC0003DMG400021409 | 2.14 | DOWN | 20.1 | 0.0040151 | Amino acid binding protein |
| PGSC0003DMG400027167 | 2.13 | DOWN | 89.49 | 0.002151 | AG-motif binding protein-2 |
| PGSC0003DMG400026671 | 2.13 | DOWN | 214.19 | 0.002151 | Arginine decarboxylase |
| PGSC0003DMG400027431 | 2.13 | DOWN | 35.38 | 0.0330512 | Conserved gene of unknown function |
| PGSC0003DMG400006133 | 2.13 | DOWN | 112.59 | 0.002151 | DGCR |
| PGSC0003DMG400006995 | 2.13 | DOWN | 293.46 | 0.002151 | PRIB5 protein |
| PGSC0003DMG400026155 | 2.13 | DOWN | 12.54 | 0.0103049 | S-locus-specific glycoprotein S6 |
| PGSC0003DMG400014607 | 2.12 | DOWN | 57.9 | 0.002151 | Aspartyl protease family protein |
| PGSC0003DMG400010579 | 2.12 | DOWN | 52.89 | 0.002151 | Conserved gene of unknown function |
| PGSC0003DMG400025613 | 2.12 | DOWN | 475.57 | 0.002151 | Protein kinase APK1B, chloroplast |
| PGSC0003DMG400031156 | 2.11 | DOWN | 24.57 | 0.002151 | Ankyrin repeat-containing protein |
| PGSC0003DMG400033126 | 2.11 | DOWN | 13.89 | 0.0057258 | Aspartate kinase |
| PGSC0003DMG400028459 | 2.11 | DOWN | 92.08 | 0.002151 | BRASSINAZOLE-RESISTANT 2 protein |
| PGSC0003DMG400014051 | 2.11 | DOWN | 459.1 | 0.002151 | Drought-induced protein 1 |
| PGSC0003DMG400014214 | 2.1 | DOWN | 168.22 | 0.002151 | Conserved gene of unknown function |
| PGSC0003DMG400028579 | 2.1 | DOWN | 77.46 | 0.002151 | Gene of unknown function |
| PGSC0003DMG401029824 | 2.1 | DOWN | 44.07 | 0.002151 | Glucosyltransferase |
| PGSC0003DMG400030320 | 2.1 | DOWN | 68.94 | 0.002151 | GTP1/OBG family member |
| PGSC0003DMG400011180 | 2.1 | DOWN | 17.61 | 0.0057258 | Potassium channel tetramerization domain-containing protein |
| PGSC0003DMG400025884 | 2.1 | DOWN | 46.3 | 0.0040151 | U-box domain-containing protein 25 |
| PGSC0003DMG400015015 | 2.1 | DOWN | 59.73 | 0.002151 | WRKY transcription factor |
| PGSC0003DMG400001307 | 2.09 | DOWN | 18.54 | 0.002151 | Conserved gene of unknown function |
| PGSC0003DMG400031860 | 2.09 | DOWN | 13.43 | 0.0073051 | Conserved gene of unknown function |
| GPP1 | 2.09 | DOWN | 31.77 | 0.002151 | Proline-rich protein |
| PGSC0003DMG400012421 | 2.08 | DOWN | 120.78 | 0.002151 | Alpha-expansin 12 |
| PRP1 | 2.08 | DOWN | 526.07 | 0.0103049 | Glutathion S-transferase |
| PGSC0003DMG400003208 | 2.08 | DOWN | 11.96 | 0.0088917 | Phosphatidylcholine transfer protein |
| PGSC0003DMG400002426 | 2.08 | DOWN | 4.14 | 0.0457077 | Resistance gene |
| ERF1 | 2.07 | DOWN | 93.76 | 0.002151 | ERF transcription factor |
| EXPA5 | 2.07 | DOWN | 48.41 | 0.0088917 | Expansin |
| PGSC0003DMG400022312 | 2.07 | DOWN | 30.42 | 0.0073051 | Zinc finger protein |
| PGSC0003DMG400003181 | 2.06 | DOWN | 84.21 | 0.002151 | Beta-glucosidase 08 |
| PGSC0003DMG400023344 | 2.06 | DOWN | 554.31 | 0.002151 | Chlorophyll a-b binding protein 6A, chloroplastic |
| PGSC0003DMG400010504 | 2.06 | DOWN | 12.61 | 0.0204365 | Conserved gene of unknown function |
| PGSC0003DMG400014558 | 2.06 | DOWN | 59.48 | 0.0191534 | Conserved gene of unknown function |
| PGSC0003DMG400031365 | 2.06 | DOWN | 33.21 | 0.002151 | Phenylalanine ammonia-lyase |
| PGSC0003DMG400027449 | 2.06 | DOWN | 63.5 | 0.002151 | Trehalose-6-phosphate synthase |
| PCM1 | 2.05 | DOWN | 718.99 | 0.0040151 | Calmodulin |
| PGSC0003DMG402006194 | 2.05 | DOWN | 60.55 | 0.002151 | Early-responsive to dehydration 7 |
| PGSC0003DMG400002504 | 2.05 | DOWN | 92.54 | 0.002151 | PRA1 family protein F3 |
| PGSC0003DMG400003955 | 2.04 | DOWN | 40.28 | 0.002151 | Pentatricopeptide repeat-containing protein |
| PGSC0003DMG400007458 | 2.04 | DOWN | 65.97 | 0.002151 | RAV |
| PGSC0003DMG400019253 | 2.04 | DOWN | 64.77 | 0.002151 | S-adenosylmethionine-dependent methyltransferase |
| PGSC0003DMG400027241 | 2.04 | DOWN | 16.1 | 0.0457077 | Zinc finger protein |
| PGSC0003DMG400006631 | 2.03 | DOWN | 63.91 | 0.002151 | 60S ribosomal protein L34 |
| PGSC0003DMG400028607 | 2.03 | DOWN | 24.11 | 0.002151 | Ca2+ antiporter/cation exchanger |
| PGSC0003DMG400018446 | 2.03 | DOWN | 36.33 | 0.0073051 | Cinnamyl alcohol dehydrogenase |
| PGSC0003DMG400033029 | 2.03 | DOWN | 61.76 | 0.0425183 | Enhanced disease susceptibility 1 protein |
| PGSC0003DMG400030882 | 2.03 | DOWN | 45.85 | 0.002151 | Plant viral-response family protein |
| PGSC0003DMG400013524 | 2.03 | DOWN | 15.36 | 0.0177243 | Protein kinase family protein |
| PGSC0003DMG400016290 | 2.03 | DOWN | 149.19 | 0.0040151 | Serine/threonine-protein phosphatase |
| PGSC0003DMG400007258 | 2.03 | DOWN | 126.34 | 0.002151 | Tyrosine specific protein phosphatase and dual specificity protein phosphatase |
| STADC | 2.02 | DOWN | 32.58 | 0.002151 | Arginine decarboxylase |
| PGSC0003DMG400012380 | 2.02 | DOWN | 35.05 | 0.0040151 | ATEXO70E1 |
| PGSC0003DMG400009114 | 2.02 | DOWN | 34.32 | 0.0103049 | BRASSINOSTEROID INSENSITIVE 1-associated receptor kinase 1 |
| PGSC0003DMG400029518 | 2.02 | DOWN | 335.25 | 0.002151 | Conserved gene of unknown function |
| PGSC0003DMG402001984 | 2.01 | DOWN | 36.86 | 0.0118276 | Charged multivesicular body protein |
| PGSC0003DMG400031861 | 2.01 | DOWN | 38.85 | 0.002151 | Conserved gene of unknown function |
| PGSC0003DMG402017989 | 2.01 | DOWN | 161.06 | 0.002151 | Cyclic nucleotide-gated calmodulin-binding ion channel |
| PGSC0003DMG400022075 | 2.01 | DOWN | 107.92 | 0.002151 | Cytochrome P450 |
| PGSC0003DMG400004291 | 2.01 | DOWN | 17.62 | 0.0057258 | Oligopeptide transporter |
| PGSC0003DMG400011745 | 2.01 | DOWN | 147.17 | 0.002151 | VQ motif-containing protein |
| PGSC0003DMG400029105 | 2.01 | DOWN | 42.62 | 0.002151 | Zinc finger family protein |
| PGSC0003DMG400019526 | 2.01 | DOWN | 223.88 | 0.002151 | Zinc finger protein |
| PGSC0003DMG400011730 | 2 | DOWN | 106.4 | 0.002151 | Conserved gene of unknown function |
| PGSC0003DMG400022727 | 2 | DOWN | 17.37 | 0.0088917 | Conserved gene of unknown function |
| PGSC0003DMG401030325 | 2 | DOWN | 58.55 | 0.002151 | Transcription factor |
| PGSC0003DMG400003924 | 1.99 | DOWN | 27.44 | 0.002151 | Amino acid binding protein |
| PGSC0003DMG401018530 | 1.99 | DOWN | 55.98 | 0.0040151 | Carboxylesterase np |
| PGSC0003DMG400009146 | 1.99 | DOWN | 84.87 | 0.0295229 | Chaperone protein dnaJ |
| PGSC0003DMG400008300 | 1.99 | DOWN | 34.31 | 0.0177243 | Chlorophyll a/b binding protein |
| PGSC0003DMG400013367 | 1.99 | DOWN | 44.58 | 0.0435373 | Conserved gene of unknown function |
| PGSC0003DMG400029315 | 1.98 | DOWN | 34.87 | 0.002151 | Conserved gene of unknown function |
| PGSC0003DMG400018238 | 1.98 | DOWN | 23.73 | 0.0330512 | Hydrolase |
| PGSC0003DMG400030220 | 1.98 | DOWN | 37.69 | 0.0057258 | Protein cbxX, chromosomal |
| PGSC0003DMG400023414 | 1.97 | DOWN | 139.06 | 0.002151 | LEM3 (Ligand-effect modulator 3) family protein |
| PGSC0003DMG400030029 | 1.97 | DOWN | 27.22 | 0.002151 | Peptide-N4-(N-acetyl-beta-glucosaminyl)asparagine amidase A |
| PGSC0003DMG401027186 | 1.97 | DOWN | 88.92 | 0.002151 | Plastidic phosphate translocator2 |
| PGSC0003DMG400008472 | 1.97 | DOWN | 150.36 | 0.002151 | W-3 desaturase |
| PGSC0003DMG400024281 | 1.96 | DOWN | 380.97 | 0.0118276 | Gamma aminobutyrate transaminase isoform2 |
| PGSC0003DMG400005213 | 1.96 | DOWN | 56.65 | 0.0040151 | Protein phosphatase-2C |
| PGSC0003DMG400001474 | 1.95 | DOWN | 75.34 | 0.002151 | Amino acid transporter |
| PGSC0003DMG400013007 | 1.95 | DOWN | 104.25 | 0.0057258 | Conserved gene of unknown function |
| PGSC0003DMG400017230 | 1.95 | DOWN | 31.32 | 0.002151 | Conserved gene of unknown function |
| PGSC0003DMG400006179 | 1.95 | DOWN | 141.28 | 0.0040151 | Nodulin family protein |
| PGSC0003DMG400012138 | 1.95 | DOWN | 857.72 | 0.0073051 | N-rich protein |
| PGSC0003DMG400022294 | 1.94 | DOWN | 39.62 | 0.0057258 | Glycerophosphodiesterase |
| PGSC0003DMG400008381 | 1.93 | DOWN | 232.04 | 0.0103049 | Arabinogalactan peptide 14 |
| PGSC0003DMG400020384 | 1.93 | DOWN | 155.89 | 0.0040151 | Conserved gene of unknown function |
| PGSC0003DMG400032820 | 1.93 | DOWN | 386.43 | 0.002151 | Protein phosphatase 2c |
| PGSC0003DMG400017518 | 1.93 | DOWN | 1169.14 | 0.0088917 | Zinc-binding protein |
| PGSC0003DMG400016695 | 1.92 | DOWN | 277.26 | 0.002151 | Chlorophyll a-b binding protein 50, chloroplastic |
| PGSC0003DMG400031471 | 1.92 | DOWN | 47.78 | 0.0040151 | Leucine-rich repeat family protein |
| PGSC0003DMG400029738 | 1.92 | DOWN | 122.58 | 0.0478271 | LEXYL2 protein |
| PGSC0003DMG400008936 | 1.92 | DOWN | 44.04 | 0.002151 | Transcription factor |
| PGSC0003DMG400011052 | 1.92 | DOWN | 27.7 | 0.0204365 | Zinc finger protein |
| ERF5 | 1.91 | DOWN | 607.59 | 0.002151 | Ethylene response factor 4 |
| PGSC0003DMG400016973 | 1.91 | DOWN | 38.68 | 0.0073051 | F-box and wd40 domain protein |
| PGSC0003DMG400022263 | 1.91 | DOWN | 90.05 | 0.0073051 | Fructose-bisphosphate aldolase |
| PGSC0003DMG400024772 | 1.91 | DOWN | 106.65 | 0.002151 | Protein phosphatase 2c |
| PGSC0003DMG400009005 | 1.91 | DOWN | 29.06 | 0.0243888 | Triacylglycerol lipase |
| STWRKY | 1.91 | DOWN | 245.26 | 0.002151 | WRKY-type DNA binding protein |
| PGSC0003DMG400002756 | 1.9 | DOWN | 50.31 | 0.002151 | Beta-fructofuranosidase |
| PGSC0003DMG400003964 | 1.9 | DOWN | 79.51 | 0.002151 | GT2 protein |
| PGSC0003DMG400009995 | 1.9 | DOWN | 22.48 | 0.01619 | Phosphate transporter |
| PGSC0003DMG400004639 | 1.9 | DOWN | 577.19 | 0.0073051 | Photosystem II 5 kDa protein, chloroplastic |
| PGSC0003DMG400021435 | 1.9 | DOWN | 35.3 | 0.0088917 | Protein PHLOEM PROTEIN 2-LIKE A10 |
| PGSC0003DMG400045432 | 1.89 | DOWN | 30.25 | 0.0103049 | Spotted leaf protein |
| PGSC0003DMG400012635 | 1.88 | DOWN | 83.7 | 0.0040151 | Acetylglucosaminyltransferase |
| PGSC0003DMG400004501 | 1.88 | DOWN | 167.97 | 0.002151 | BRASSINAZOLE-RESISTANT 1 protein |
| PGSC0003DMG400030503 | 1.88 | DOWN | 57.8 | 0.0243888 | Conserved gene of unknown function |
| PGSC0003DMG400003123 | 1.88 | DOWN | 437.02 | 0.002151 | Fructose-bisphosphate aldolase |
| PGSC0003DMG400027513 | 1.88 | DOWN | 149.41 | 0.0230721 | Gene of unknown function |
| PGSC0003DMG400002169 | 1.88 | DOWN | 250.16 | 0.002151 | Glutathione-S-transferase |
| PGSC0003DMG400021354 | 1.88 | DOWN | 133.94 | 0.002151 | NC domain-containing protein |
| PGSC0003DMG400005805 | 1.88 | DOWN | 1306.55 | 0.0132528 | Photosystem I reaction center subunit |
| PGSC0003DMG400031235 | 1.88 | DOWN | 45.55 | 0.0466816 | Protein kinase atsik |
| PGSC0003DMG400024944 | 1.88 | DOWN | 115.92 | 0.0073051 | Protein phosphatase 2c |
| PGSC0003DMG400030410 | 1.88 | DOWN | 49.68 | 0.002151 | Protein phosphatase 2C 4 |
| PGSC0003DMG400002386 | 1.87 | DOWN | 57.25 | 0.0118276 | Conserved gene of unknown function |
| PGSC0003DMG400017149 | 1.87 | DOWN | 53.12 | 0.030665 | Conserved gene of unknown function |
| PGSC0003DMG400006640 | 1.87 | DOWN | 105.1 | 0.0057258 | Spotted leaf protein |
| PGSC0003DMG400018632 | 1.86 | DOWN | 15.47 | 0.0132528 | F-box family protein |
| PGSC0003DMG400022039 | 1.86 | DOWN | 98.58 | 0.0147116 | FKBP-type peptidyl-prolyl cis-trans isomerase 4, chloroplastic |
| PGSC0003DMG400030405 | 1.86 | DOWN | 315.06 | 0.0103049 | Heat shock cognate 70 kDa protein 1 |
| PGSC0003DMG400018677 | 1.86 | DOWN | 145.27 | 0.0073051 | Lectin kinase |
| PGSC0003DMG400013804 | 1.86 | DOWN | 22.88 | 0.0466816 | Receptor protein kinase |
| PGSC0003DMG400002370 | 1.86 | DOWN | 157.64 | 0.002151 | Vesicle-associated membrane protein |
| PGSC0003DMG400012012 | 1.85 | DOWN | 492.6 | 0.0057258 | Fructose-bisphosphate aldolase |
| PGSC0003DMG400003655 | 1.84 | DOWN | 147.6 | 0.0057258 | Aspartic proteinase nepenthesin-1 |
| PGSC0003DMG400020139 | 1.84 | DOWN | 143.29 | 0.0103049 | Auxin-induced protein 22B |
| PGSC0003DMG400032113 | 1.84 | DOWN | 1118.95 | 0.044629 | Conserved gene of unknown function |
| PGSC0003DMG400014678 | 1.84 | DOWN | 25.1 | 0.0243888 | Serine/threonine-protein kinase cx32 |
| PGSC0003DMG400008301 | 1.83 | DOWN | 1155.73 | 0.0342702 | Chlorophyll a/b binding protein |
| PGSC0003DMG400025814 | 1.83 | DOWN | 79.34 | 0.0040151 | CXE carboxylesterase |
| PGSC0003DMG400030209 | 1.82 | DOWN | 59.05 | 0.0040151 | BRASSINOSTEROID INSENSITIVE 1-associated receptor kinase 1 |
| PGSC0003DMG400026794 | 1.82 | DOWN | 61.72 | 0.0040151 | Conserved gene of unknown function |
| PGSC0003DMG400002826 | 1.82 | DOWN | 87.03 | 0.0132528 | Kelch repeat-containing F-box family protein |
| PGSC0003DMG400030035 | 1.82 | DOWN | 105.59 | 0.0132528 | Receptor protein kinase |
| PGSC0003DMG401001531 | 1.81 | DOWN | 32.02 | 0.0457077 | Conserved gene of unknown function |
| PTT#2-1 | 1.81 | DOWN | 457.43 | 0.0103049 | Twi1 protein |
| PGSC0003DMG400024961 | 1.81 | DOWN | 75.01 | 0.0040151 | WRKY domain class transcription factor |
| PGSC0003DMG400031852 | 1.8 | DOWN | 26.84 | 0.0466816 | Conserved gene of unknown function |
| PGSC0003DMG400000639 | 1.8 | DOWN | 25.65 | 0.0366624 | Delta 9 desaturase |
| PGSC0003DMG400011981 | 1.8 | DOWN | 228.01 | 0.0057258 | Gene of unknown function |
| PGSC0003DMG400044745 | 1.8 | DOWN | 59.19 | 0.0366624 | Kinase |
| PGSC0003DMG400010050 | 1.8 | DOWN | 210.15 | 0.0103049 | Proline oxidase/dehydrogenase 1 |
| PGSC0003DMG400016217 | 1.8 | DOWN | 118.62 | 0.002151 | Ring finger protein |
| PGSC0003DMG400004898 | 1.8 | DOWN | 717.91 | 0.0147116 | Transcription factor JERF1 |
| PGSC0003DMG400028462 | 1.79 | DOWN | 46.3 | 0.0057258 | ACRE 276 |
| PGSC0003DMG400019883 | 1.79 | DOWN | 54.91 | 0.0057258 | Chitinase |
| PGSC0003DMG400002038 | 1.79 | DOWN | 20.17 | 0.0230721 | Kinase |
| PGSC0003DMG400021116 | 1.79 | DOWN | 27.17 | 0.0230721 | UDP-glucoronosyl/UDP-glucosyl transferase family protein |
| PGSC0003DMG400026323 | 1.79 | DOWN | 43.63 | 0.0118276 | UDP-glucose:glucosyltransferase |
| PGSC0003DMG400003126 | 1.78 | DOWN | 132.62 | 0.0132528 | AG-motif binding protein-4 |
| PGSC0003DMG400010631 | 1.78 | DOWN | 511.21 | 0.0088917 | Calcium-binding allergen Ole e |
| PGSC0003DMG400003520 | 1.78 | DOWN | 41.11 | 0.0103049 | Conserved gene of unknown function |
| PGSC0003DMG400022893 | 1.78 | DOWN | 88.55 | 0.0132528 | Elicitor-inducible cytochrome P450 |
| PGSC0003DMG400024667 | 1.78 | DOWN | 41 | 0.0243888 | Glycosyltransferase |
| PGSC0003DMG400010401 | 1.78 | DOWN | 47.87 | 0.0330512 | Homeodomain leucine-zipper 1 |
| PGSC0003DMG400027319 | 1.77 | DOWN | 121.47 | 0.0073051 | Conserved gene of unknown function |
| PGSC0003DMG400024708 | 1.77 | DOWN | 260.02 | 0.0118276 | Rubber elongation factor protein |
| PGSC0003DMG400008205 | 1.76 | DOWN | 61.15 | 0.0040151 | C2H2L domain class transcription factor |
| PGSC0003DMG400020141 | 1.76 | DOWN | 122.76 | 0.0295229 | Photosystem II reaction center W protein, chloroplastic |
| PGSC0003DMG400005931 | 1.76 | DOWN | 87.06 | 0.0177243 | Sterol delta-7 reductase DWF5 |
| PGSC0003DMG400026322 | 1.76 | DOWN | 56.42 | 0.0191534 | UDP-glucose:glucosyltransferase |
| PGSC0003DMG400000185 | 1.75 | DOWN | 271.03 | 0.0132528 | ATP binding protein |
| PGSC0003DMG400029920 | 1.75 | DOWN | 69.33 | 0.0191534 | Conserved gene of unknown function |
| PGSC0003DMG400030683 | 1.74 | DOWN | 76.56 | 0.0118276 | Arogenate dehydrogenase |
| PGSC0003DMG402009727 | 1.73 | DOWN | 121.08 | 0.01619 | Calcium ion binding protein |
| PGSC0003DMG401018380 | 1.73 | DOWN | 57.69 | 0.0258259 | Diacylglycerol kinase variant A |
| PGSC0003DMG400030181 | 1.73 | DOWN | 246.03 | 0.0132528 | Esterase/lipase/thioesterase family protein |
| PGSC0003DMG400015424 | 1.73 | DOWN | 43.85 | 0.0204365 | Mature anther-specific protein LAT61 |
| PGSC0003DMG401013639 | 1.73 | DOWN | 191.3 | 0.0258259 | RNA recognition motif-containing protein |
| PGSC0003DMG400007317 | 1.72 | DOWN | 23.78 | 0.0466816 | Cationic amino acid transporter |
| PGSC0003DMG400022172 | 1.71 | DOWN | 122.75 | 0.0230721 | Ccaat-binding transcription factor subunit A |
| PGSC0003DMG400004930 | 1.71 | DOWN | 68.77 | 0.0478271 | Conserved gene of unknown function |
| PGSC0003DMG400026212 | 1.71 | DOWN | 430.27 | 0.0204365 | Conserved gene of unknown function |
| PGSC0003DMG400008639 | 1.71 | DOWN | 56.04 | 0.0489636 | Galactose mutarotase |
| PGSC0003DMG400031120 | 1.71 | DOWN | 126.93 | 0.0230721 | Glycine-rich protein A3 |
| PGSC0003DMG400027276 | 1.71 | DOWN | 120.14 | 0.0282933 | Mg protoporphyrin IX chelatase |
| PGSC0003DMG400031454 | 1.71 | DOWN | 75.47 | 0.0389556 | UDP-glucose:glucosyltransferase |
| APSR1 | 1.7 | DOWN | 68.45 | 0.0425183 | Adenylyl-sulfate reductase |
| PGSC0003DMG400027429 | 1.7 | DOWN | 73.3 | 0.0282933 | CONSTANS interacting protein 6 |
| PGSC0003DMG400012224 | 1.7 | DOWN | 143.84 | 0.0377582 | Electron carrier |
| PGSC0003DMG400000114 | 1.69 | DOWN | 128.49 | 0.0243888 | Phosphatidylinositol 3-and 4-kinase family protein |
| PGSC0003DMG400013545 | 1.69 | DOWN | 89.41 | 0.0318847 | Protein kinase APK1B, chloroplast |
| PGSC0003DMG400018428 | 1.68 | DOWN | 28.03 | 0.0354738 | Bacterial spot disease resistance protein 4 |
| PGSC0003DMG400009699 | 1.68 | DOWN | 105.54 | 0.0218313 | Guanylate kinase |
| PGSC0003DMG400035823 | 1.67 | DOWN | 207.86 | 0.0204365 | Amino acid transporter |
| PGSC0003DMG400000493 | 1.67 | DOWN | 399.43 | 0.0318847 | Carbonic anhydrase |
| LHCB1-3 | 1.67 | DOWN | 228.01 | 0.0435373 | Chlorophyll a/b binding protein |
| PGSC0003DMG400012315 | 1.67 | DOWN | 180.72 | 0.0499455 | Conserved gene of unknown function |
| PGSC0003DMG400029832 | 1.67 | DOWN | 40.91 | 0.030665 | Sugar-dependent1 |
| PGSC0003DMG400005713 | 1.66 | DOWN | 60.82 | 0.0330512 | Conserved gene of unknown function |
| PGSC0003DMG400032498 | 1.66 | DOWN | 92.41 | 0.0489636 | Protein kinase |
| PGSC0003DMG400019975 | 1.65 | DOWN | 55.36 | 0.0366624 | Ankyrin repeat-containing protein |
| PGSC0003DMG400011213 | 1.65 | DOWN | 46.77 | 0.0389556 | Eukaryotic translation initiation factor 3 subunit |
| PGSC0003DMG400029921 | 1.65 | DOWN | 59.83 | 0.0457077 | GRAS9 |
| PGSC0003DMG400016603 | 1.65 | DOWN | 40.8 | 0.044629 | MAC/Perforin domain containing protein |
| PGSC0003DMG400009959 | 1.65 | DOWN | 108.32 | 0.0330512 | Ornithine decarboxylase |
| PGSC0003DMG400000328 | 1.64 | DOWN | 290.48 | 0.0295229 | Charged multivesicular body protein 2a |
| PGSC0003DMG400029999 | 1.64 | DOWN | 35.41 | 0.0466816 | Conserved gene of unknown function |
| PGSC0003DMG400003429 | 1.63 | DOWN | 63.42 | 0.0435373 | Atypical receptor-like kinase 1 |
| PGSC0003DMG400018517 | 1.63 | DOWN | 467.13 | 0.0342702 | Fad NAD binding oxidoreductases |
| PGSC0003DMG400027771 | 1.63 | DOWN | 117.48 | 0.0318847 | Shaggy-like kinase 6 (Nsk6) |
| PGSC0003DMG400011012 | 1.62 | DOWN | 281.13 | 0.0389556 | Glutathione-S-transferase |
| PGSC0003DMG400015767 | 10.51 | UP | 5.35 | 0.002151 | Myb-like transcription factor |
| PGSC0003DMG400026035 | 9.47 | UP | 11.06 | 0.002151 | AP2/ERF domain-containing transcription factor |
| PGSC0003DMG400008453 | 9.08 | UP | 3.25 | 0.0499455 | Conserved gene of unknown function |
| PGSC0003DMG400026186 | 6.75 | UP | 2.46 | 0.0088917 | Indole-3-acetic acid-amido synthetase GH3.6 |
| PGSC0003DMG400026023 | 6.72 | UP | 5.79 | 0.002151 | Nuc-1 negative regulatory protein preg |
| PGSC0003DMG400014104 | 6.36 | UP | 21.71 | 0.002151 | Patatin-2-Kuras 4 |
| PGSC0003DMG402017090 | 6.24 | UP | 6.73 | 0.0425183 | Patatin-04/09 |
| PGSC0003DMG400028188 | 5.76 | UP | 8.33 | 0.0118276 | Calmodulin binding protein |
| PGSC0003DMG400005823 | 5.48 | UP | 12.6 | 0.002151 | Zinc finger protein |
| PGSC0003DMG400017168 | 4.76 | UP | 6.41 | 0.0057258 | Conserved gene of unknown function |
| PGSC0003DMG400022709 | 4.76 | UP | 10.99 | 0.002151 | GRAS family transcription factor |
| PGSC0003DMG400024368 | 4.69 | UP | 47.82 | 0.002151 | Conserved gene of unknown function |
| PGSC0003DMG400005486 | 4.45 | UP | 11.11 | 0.002151 | Nucleic acid binding protein |
| PGSC0003DMG400002620 | 4.42 | UP | 8.21 | 0.002151 | AP2 domain class transcription factor |
| PGSC0003DMG400028535 | 4.41 | UP | 43.98 | 0.002151 | Conserved gene of unknown function |
| PGSC0003DMG400016499 | 4.15 | UP | 5.63 | 0.0057258 | Conserved gene of unknown function |
| PGSC0003DMG400024058 | 3.98 | UP | 6.2 | 0.0088917 | Gene of unknown function |
| PGSC0003DMG400030663 | 3.92 | UP | 11.48 | 0.01619 | Hydroxyproline-rich glycoprotein |
| PGSC0003DMG400019964 | 3.87 | UP | 60.48 | 0.002151 | Conserved gene of unknown function |
| PGSC0003DMG400025721 | 3.74 | UP | 217.34 | 0.002151 | Conserved gene of unknown function |
| PGSC0003DMG400001668 | 3.73 | UP | 16.14 | 0.0073051 | SAUR family protein |
| PGSC0003DMG402023841 | 3.67 | UP | 2.1 | 0.0401819 | Sterol desaturase |
| PGSC0003DMG400016455 | 3.62 | UP | 69.63 | 0.002151 | Conserved gene of unknown function |
| PGSC0003DMG400020968 | 3.6 | UP | 22.38 | 0.002151 | Flavonol 4'-sulfotransferase |
| PGSC0003DMG400023666 | 3.54 | UP | 8.33 | 0.0230721 | Nucleic acid binding protein |
| PGSC0003DMG400015465 | 3.45 | UP | 4.29 | 0.0132528 | Conserved gene of unknown function |
| PGSC0003DMG400004992 | 3.45 | UP | 2.88 | 0.0318847 | Endo-beta-1,4-glucanase |
| PGSC0003DMG400001614 | 3.43 | UP | 10.76 | 0.0088917 | SAUR family protein |
| PGSC0003DMG400022751 | 3.4 | UP | 3.96 | 0.002151 | Molybdopterin cofactor sulfurase |
| PGSC0003DMG400004257 | 3.36 | UP | 303.17 | 0.002151 | F-box family protein |
| PGSC0003DMG400026264 | 3.26 | UP | 22.07 | 0.002151 | Homeobox-leucine zipper protein |
| PGSC0003DMG400016790 | 3.25 | UP | 78.95 | 0.002151 | Homeodomain-leucine zipper protein 56 |
| PGSC0003DMG400026616 | 3.22 | UP | 20.27 | 0.002151 | Wound-induced protein |
| PGSC0003DMG400001858 | 3.17 | UP | 156.48 | 0.002151 | Conserved gene of unknown function |
| ENT3 | 3.14 | UP | 34.88 | 0.002151 | Equilibrative nucleoside transporter |
| PGSC0003DMG400005442 | 3.13 | UP | 16.16 | 0.002151 | Uncharacterized GPI-anchored protein |
| PGSC0003DMG400001543 | 3.12 | UP | 7.27 | 0.0057258 | Conserved gene of unknown function |
| PGSC0003DMG400003521 | 3.03 | UP | 21.33 | 0.002151 | Endo-beta-mannanase |
| PGSC0003DMG400000363 | 3.02 | UP | 7.95 | 0.0088917 | Avr9/Cf-9 rapidly elicited protein 146 |
| PGSC0003DMG400014504 | 2.98 | UP | 12 | 0.002151 | Conserved gene of unknown function |
| PGSC0003DMG400025091 | 2.96 | UP | 21.74 | 0.0354738 | Conserved gene of unknown function |
| PGSC0003DMG400021821 | 2.95 | UP | 50.31 | 0.002151 | Conserved gene of unknown function |
| PGSC0003DMG400007516 | 2.93 | UP | 14.23 | 0.002151 | Conserved gene of unknown function |
| PGSC0003DMG400000280 | 2.92 | UP | 16.58 | 0.0057258 | GATA transcription factor 21 |
| GA2OX1 | 2.91 | UP | 10.92 | 0.0040151 | Gibberellin 2-oxidase 1 |
| PGSC0003DMG400041029 | 2.89 | UP | 88.82 | 0.002151 | Bcl-2-associated athanogene |
| PGSC0003DMG400001060 | 2.87 | UP | 5.4 | 0.0073051 | Brassinosteroid hydroxylase |
| PGSC0003DMG400000069 | 2.85 | UP | 15.29 | 0.0103049 | Plant cell wall protein SlTFR88 |
| PGSC0003DMG400006444 | 2.84 | UP | 11.35 | 0.0040151 | Calmodulin binding protein |
| PGSC0003DMG400009405 | 2.84 | UP | 7.48 | 0.0499455 | Ring finger protein |
| PGSC0003DMG400024755 | 2.82 | UP | 27.58 | 0.002151 | Xyloglucan endotransglucosylase/hydrolase 1 |
| PGSC0003DMG400001339 | 2.79 | UP | 10.08 | 0.0040151 | Protein 1 |
| PGSC0003DMG400005442 | 2.79 | UP | 48.81 | 0.030665 | Uncharacterized GPI-anchored protein |
| PGSC0003DMG401019811 | 2.78 | UP | 46.59 | 0.0103049 | Conserved gene of unknown function |
| PGSC0003DMG400004934 | 2.78 | UP | 9.34 | 0.0218313 | UPA25 |
| PGSC0003DMG400000415 | 2.73 | UP | 18.43 | 0.0057258 | Conserved gene of unknown function |
| PGSC0003DMG400025470 | 2.72 | UP | 19.78 | 0.002151 | Aspartic proteinase nepenthesin-1 |
| PGSC0003DMG400031168 | 2.69 | UP | 18.62 | 0.002151 | Cyclin-dependent protein kinase |
| PGSC0003DMG400018113 | 2.67 | UP | 3.82 | 0.0435373 | R2r3-myb transcription factor |
| PGSC0003DMG400005716 | 2.65 | UP | 372.27 | 0.002151 | Conserved gene of unknown function |
| PGSC0003DMG400016608 | 2.65 | UP | 55.84 | 0.002151 | Transcription factor |
| PGSC0003DMG402002024 | 2.64 | UP | 9.47 | 0.0295229 | Zinc finger protein |
| PGSC0003DMG400010342 | 2.63 | UP | 42.14 | 0.002151 | Conserved gene of unknown function |
| PGSC0003DMG400026363 | 2.62 | UP | 60.88 | 0.002151 | Conserved gene of unknown function |
| PGSC0003DMG400022264 | 2.6 | UP | 4.33 | 0.0057258 | LRR receptor-like serine/threonine-protein kinase |
| PGSC0003DMG400022341 | 2.58 | UP | 10.22 | 0.0147116 | Suberization-associated anionic peroxidase 2 |
| PGSC0003DMG400000443 | 2.52 | UP | 14.4 | 0.002151 | Conserved gene of unknown function |
| PGSC0003DMG400005633 | 2.5 | UP | 22.97 | 0.0040151 | Conserved gene of unknown function |
| PGSC0003DMG400025867 | 2.5 | UP | 60.38 | 0.002151 | Serine-threonine protein kinase, plant-type |
| PGSC0003DMG400001667 | 2.49 | UP | 31.95 | 0.0073051 | SAUR family protein |
| PGSC0003DMG400016148 | 2.48 | UP | 547.03 | 0.002151 | Homeodomain protein Hfi22 |
| PGSC0003DMG400026345 | 2.45 | UP | 26.8 | 0.002151 | Conserved gene of unknown function |
| PGSC0003DMG400030535 | 2.45 | UP | 9.72 | 0.0073051 | Wall-associated kinase |
| PGSC0003DMG400047314 | 2.43 | UP | 14.22 | 0.0282933 | Transposase |
| PGSC0003DMG400022120 | 2.43 | UP | 18.74 | 0.0057258 | YUP8H12R.23 protein |
| PGSC0003DMG400009378 | 2.42 | UP | 20.49 | 0.0243888 | DNA binding protein |
| PPCK1B | 2.42 | UP | 57.6 | 0.002151 | PEPC kinase 1b |
| PGSC0003DMG400007749 | 2.42 | UP | 29.8 | 0.002151 | Transcription factor |
| PGSC0003DMG400012972 | 2.4 | UP | 19.83 | 0.0282933 | Conserved gene of unknown function |
| PGSC0003DMG400001915 | 2.4 | UP | 16.18 | 0.002151 | Flavonoid 3-hydroxylase |
| PGSC0003DMG400009843 | 2.4 | UP | 4.11 | 0.030665 | Receptor kinase |
| PGSC0003DMG402007970 | 2.39 | UP | 27.67 | 0.002151 | Conserved gene of unknown function |
| PGSC0003DMG400030663 | 2.39 | UP | 43.96 | 0.0073051 | Hydroxyproline-rich glycoprotein |
| PGSC0003DMG400019960 | 2.38 | UP | 50.41 | 0.002151 | Tellurite resistance protein tehA |
| PPCK1A | 2.35 | UP | 30.51 | 0.002151 | PEP carboxylase kinase |
| PGSC0003DMG400031868 | 2.34 | UP | 10.1 | 0.0132528 | Conserved gene of unknown function |
| PGSC0003DMG400025144 | 2.33 | UP | 68.47 | 0.0057258 | Conserved gene of unknown function |
| PGSC0003DMG400022738 | 2.33 | UP | 5.72 | 0.0377582 | TCP domain class transcription factor |
| PGSC0003DMG400027333 | 2.32 | UP | 9.88 | 0.0218313 | Leucoanthocyanidin dioxygenase |
| PGSC0003DMG400018128 | 2.32 | UP | 9.08 | 0.0230721 | Protein phosphatase 2c |
| PGSC0003DMG400020427 | 2.29 | UP | 14.62 | 0.0218313 | DNA binding protein |
| PGSC0003DMG400026959 | 2.26 | UP | 15.16 | 0.0230721 | C3HL domain class transcription factor |
| PGSC0003DMG400023051 | 2.22 | UP | 291.25 | 0.002151 | Conserved gene of unknown function |
| PGSC0003DMG400031752 | 2.21 | UP | 6.94 | 0.0147116 | Leucine-rich repeat receptor kinase |
| PGSC0003DMG400004649 | 2.19 | UP | 24.39 | 0.0204365 | GRAS family transcription factor |
| PGSC0003DMG400011313 | 2.18 | UP | 28.56 | 0.01619 | Conserved gene of unknown function |
| PGSC0003DMG400013557 | 2.18 | UP | 39.85 | 0.0118276 | UDP-sugar:glycosyltransferase |
| PGSC0003DMG400012438 | 2.14 | UP | 9.76 | 0.0401819 | Conserved gene of unknown function |
| PGSC0003DMG400012993 | 2.14 | UP | 95.47 | 0.002151 | Conserved gene of unknown function |
| PGSC0003DMG400008146 | 2.14 | UP | 6.35 | 0.0073051 | Leucine-rich repeat receptor kinase |
| PGSC0003DMG400025434 | 2.12 | UP | 7.77 | 0.0204365 | Conserved gene of unknown function |
| PGSC0003DMG400019518 | 2.12 | UP | 4.54 | 0.0354738 | Pseudo response regulator |
| PGSC0003DMG400014150 | 2.11 | UP | 6.68 | 0.030665 | Kinase family protein |
| PGSC0003DMG400014156 | 2.1 | UP | 60.97 | 0.002151 | Aspartic proteinase nepenthesin-1 |
| PGSC0003DMG400030946 | 2.1 | UP | 75.04 | 0.002151 | BZIP transcription factor |
| PGSC0003DMG400003956 | 2.1 | UP | 20.63 | 0.0057258 | Conserved gene of unknown function |
| PGSC0003DMG400015301 | 2.09 | UP | 228.55 | 0.0258259 | Conserved gene of unknown function |
| PGSC0003DMG400026571 | 2.09 | UP | 55.07 | 0.0040151 | Conserved gene of unknown function |
| PGSC0003DMG400000129 | 2.09 | UP | 147.27 | 0.002151 | SAUR family protein |
| PGSC0003DMG400006700 | 2.08 | UP | 477.84 | 0.002151 | Conserved gene of unknown function |
| PGSC0003DMG400012531 | 2.08 | UP | 74.47 | 0.002151 | Conserved gene of unknown function |
| PGSC0003DMG400012795 | 2.07 | UP | 33.45 | 0.002151 | Cytochrome P450 |
| PGSC0003DMG400013286 | 2.07 | UP | 72.77 | 0.002151 | Ubiquitin-protein ligase |
| PGSC0003DMG400027875 | 2.05 | UP | 9.24 | 0.044629 | ATP synthase gamma chain |
| PGSC0003DMG400014384 | 2.05 | UP | 19.35 | 0.0318847 | Conserved gene of unknown function |
| PGSC0003DMG401012192 | 2.05 | UP | 378.63 | 0.0040151 | Zinc finger protein |
| PGSC0003DMG400017523 | 2.04 | UP | 47.27 | 0.0088917 | Conserved gene of unknown function |
| PGSC0003DMG400019861 | 2.03 | UP | 14.35 | 0.0271321 | BHLH transcription factor Upa20 |
| PGSC0003DMG400005626 | 2.02 | UP | 48.04 | 0.0057258 | DNA binding protein |
| PGSC0003DMG400043193 | 2.02 | UP | 22.91 | 0.0132528 | Ring finger protein |
| PGSC0003DMG400017254 | 2.01 | UP | 9.18 | 0.0271321 | Conserved gene of unknown function |
| PGSC0003DMG400000584 | 2.01 | UP | 66.47 | 0.002151 | Pseudo-response regulator 5 |
| PGSC0003DMG400017373 | 1.99 | UP | 150.93 | 0.002151 | Conserved gene of unknown function |
| PGSC0003DMG400015880 | 1.98 | UP | 31.37 | 0.0057258 | DNA binding protein |
| PGSC0003DMG400005491 | 1.98 | UP | 20.25 | 0.0073051 | Gene of unknown function |
| SGT2 | 1.98 | UP | 19.95 | 0.0073051 | UDP-glucose:solanidine glucosyltransferase |
| PGSC0003DMG400016725 | 1.97 | UP | 17.28 | 0.0354738 | Conserved gene of unknown function |
| PGSC0003DMG400001252 | 1.97 | UP | 90.02 | 0.0466816 | Leucine-rich repeat protein |
| PGSC0003DMG400024937 | 1.97 | UP | 10.56 | 0.0425183 | SCL domain class transcription factor |
| PGSC0003DMG400025414 | 1.97 | UP | 16.43 | 0.0204365 | Zinc finger protein CONSTANS-LIKE 15 |
| PGSC0003DMG400006081 | 1.94 | UP | 49.89 | 0.01619 | Conserved gene of unknown function |
| PGSC0003DMG400027475 | 1.94 | UP | 41.04 | 0.0040151 | CONSTANS |
| PGSC0003DMG400007965 | 1.94 | UP | 22.24 | 0.0377582 | Triacylglycerol lipase |
| PGSC0003DMG400003849 | 1.93 | UP | 133.68 | 0.002151 | GID1-like gibberellin receptor |
| PGSC0003DMG400003701 | 1.92 | UP | 89.89 | 0.002151 | BZIP domain class transcription factor |
| PGSC0003DMG400017582 | 1.92 | UP | 28.7 | 0.0330512 | Conserved gene of unknown function |
| PGSC0003DMG400022760 | 1.91 | UP | 35.7 | 0.0103049 | Conserved gene of unknown function |
| PGSC0003DMG400029516 | 1.91 | UP | 96.11 | 0.0204365 | Conserved gene of unknown function |
| PGSC0003DMG400024219 | 1.91 | UP | 137.1 | 0.002151 | Inositol-1,4,5-triphosphate-5-phosphatase |
| PGSC0003DMG400028048 | 1.91 | UP | 83.5 | 0.0057258 | Pectin methylesterase inhibitor isoform |
| PGSC0003DMG400019971 | 1.9 | UP | 88.84 | 0.002151 | Circadian clock-associated FKF1 |
| PGSC0003DMG400021991 | 1.9 | UP | 145.17 | 0.002151 | GID1-like gibberellin receptor |
| PGSC0003DMG400014824 | 1.89 | UP | 102.11 | 0.0147116 | Glycerol-3-phosphate transporter |
| PGSC0003DMG400018670 | 1.87 | UP | 129.12 | 0.002151 | Amino acid transporter |
| PGSC0003DMG400008020 | 1.87 | UP | 27.57 | 0.0191534 | Conserved gene of unknown function |
| PGSC0003DMG400021347 | 1.87 | UP | 37.58 | 0.0118276 | UDP-glucosyltransferase |
| PGSC0003DMG400010491 | 1.86 | UP | 77.84 | 0.0103049 | Glucan endo-1,3-beta-D-glucosidase |
| DIR1 | 1.85 | UP | 92.12 | 0.0147116 | Dirigent 1 |
| PGSC0003DMG400004057 | 1.85 | UP | 52.79 | 0.0040151 | ORF16-lacZ fusion protein |
| PGSC0003DMG400022762 | 1.82 | UP | 72.58 | 0.0088917 | F-box family protein |
| PGSC0003DMG400033168 | 1.82 | UP | 34.29 | 0.0118276 | Gene of unknown function |
| PGSC0003DMG400003697 | 1.8 | UP | 113.21 | 0.0057258 | Conserved gene of unknown function |
| PGSC0003DMG400021917 | 1.8 | UP | 28.32 | 0.030665 | Gene of unknown function |
| PGSC0003DMG400001221 | 1.79 | UP | 114.9 | 0.0401819 | EARLY flowering 4 protein |
| PGSC0003DMG400000140 | 1.76 | UP | 22.88 | 0.0230721 | ATP binding protein |
| PGSC0003DMG400020844 | 1.75 | UP | 64.27 | 0.0330512 | Conserved gene of unknown function |
| PGSC0003DMG400014545 | 1.74 | UP | 65.29 | 0.0282933 | Conserved gene of unknown function |
| PGSC0003DMG400011125 | 1.74 | UP | 251.17 | 0.0295229 | Gene of unknown function |
| PGSC0003DMG400003229 | 1.73 | UP | 78.17 | 0.0118276 | Conserved gene of unknown function |
| PGSC0003DMG400008052 | 1.71 | UP | 123.23 | 0.0230721 | Conserved gene of unknown function |
| PGSC0003DMG402000198 | 1.71 | UP | 50.2 | 0.0499455 | Conserved gene of unknown function |
| PGSC0003DMG400013680 | 1.71 | UP | 68.59 | 0.030665 | Pectinesterase |
| PGSC0003DMG400000350 | 1.71 | UP | 57.3 | 0.0425183 | Zinc finger CCCH domain-containing protein 20 |
| PGSC0003DMG400009762 | 1.7 | UP | 59.99 | 0.0366624 | Cytochrome P450 |
| PGSC0003DMG400029729 | 1.7 | UP | 45.48 | 0.0354738 | Dof zinc finger protein |
| PGSC0003DMG400016223 | 1.7 | UP | 103.85 | 0.0230721 | Peroxidase 17 |
| PGSC0003DMG400030625 | 1.7 | UP | 112.17 | 0.0295229 | Receptor protein kinase CLAVATA1 |
| PGSC0003DMG400015925 | 1.69 | UP | 331.55 | 0.0489636 | Amine oxidase |
| PGSC0003DMG402019696 | 1.69 | UP | 298.33 | 0.0377582 | Conserved gene of unknown function |
| PGSC0003DMG402013540 | 1.68 | UP | 59.81 | 0.0435373 | Starch synthase VI |
| PGSC0003DMG400016697 | 1.67 | UP | 91.72 | 0.0191534 | Eukaryotic translation initiation factor |
| PGSC0003DMG400018641 | 1.67 | UP | 127.05 | 0.0230721 | S-adenosyl-methionine-sterol-C-methyltransferase |
| PGSC0003DMG400004062 | 1.66 | UP | 172.83 | 0.0282933 | DOF domain class transcription factor |
| PGSC0003DMG400005130 | 1.64 | UP | 62.17 | 0.0425183 | Conserved gene of unknown function |
| PGSC0003DMG400002044 | 1.6 | UP | 58.2 | 0.0478271 | Transcription factor |

**Supplemental Table 4: list of 111 genes differentially regulated in both Waneta and Atlantic after Lso treatment. Supplemental** **Table 4A** List of the 61 genes down regulated after Lso treatment in both varieties. **Supplemental** **Table 4B** List of the 9 genes up regulated after Lso treatment. **Supplemental** **Table 4C** list of the 5 genes up regulated in Waneta and down regulated in Atlantic. **Supplemental** **Table 4D** list of the 36 genes down regulated in Waneta and up regulated in Atlantic.

**Supplemental** **Table 4A** List of the 61 genes down regulated after Lso treatment in both varieties.

| **transcript** | **Fold Change in Waneta** | **Direction** | **Fold Change in Atlantic** | **Gene Description** |
| --- | --- | --- | --- | --- |
| PGSC0003DMT400001709 | 1.8 | DOWN | 5.9 | Delta 9 desaturase |
| PGSC0003DMT400003608 | 2.07 | DOWN | 4.43 | Expansin |
| PGSC0003DMT400004126 | 2.09 | DOWN | 11.75 | Proline-rich protein |
| PGSC0003DMT400004181 | 2.02 | DOWN | 10.76 | Arginine decarboxylase |
| PGSC0003DMT400005519 | 2.26 | DOWN | 38.34 | C-4 sterol methyl oxidase 2 |
| PGSC0003DMT400006132 | 1.87 | DOWN | 3.85 | Conserved gene of unknown function |
| PGSC0003DMT400008103 | 1.88 | DOWN | 6.36 | Fructose-bisphosphate aldolase |
| PGSC0003DMT400008240 | 4.84 | DOWN | 25.19 | Conserved gene of unknown function |
| PGSC0003DMT400009330 | 3.11 | DOWN | 4.07 | Lactoylglutathione lyase |
| PGSC0003DMT400010643 | 2.48 | DOWN | 4.16 | Ca2+ antiporter/cation exchanger |
| PGSC0003DMT400010957 | 2.01 | DOWN | 5.61 | Oligopeptide transporter |
| PGSC0003DMT400010985 | 2.84 | DOWN | 14.8 | Chlorophyll a,b binding protein type I |
| PGSC0003DMT400011811 | 1.9 | DOWN | 4.53 | Photosystem II 5 kDa protein, chloroplastic |
| PGSC0003DMT400015184 | 1.76 | DOWN | 8.31 | Sterol delta-7 reductase DWF5 |
| PGSC0003DMT400018704 | 6.73 | DOWN | 9.62 | GDSL-lipase 1 |
| PGSC0003DMT400019296 | 2.04 | DOWN | 9.17 | RAV |
| PGSC0003DMT400020100 | 2.26 | DOWN | 4.54 | Chlorophyll a-b binding protein 8, chloroplastic |
| PGSC0003DMT400021389 | 2.91 | DOWN | 13.88 | Chlorophyll a/b binding protein |
| PGSC0003DMT400021391 | 1.99 | DOWN | 7.24 | Chlorophyll a/b binding protein |
| PGSC0003DMT400021604 | 1.93 | DOWN | 5.57 | Arabinogalactan peptide 14 |
| PGSC0003DMT400021824 | 1.97 | DOWN | 3.06 | W-3 desaturase |
| PGSC0003DMT400022072 | 3.22 | DOWN | 5.43 | Chlorophyll a-b binding protein 13, chloroplastic |
| PGSC0003DMT400024317 | 2.69 | DOWN | 3.71 | Glutaredoxin-C9 |
| PGSC0003DMT400024601 | 11.89 | DOWN | 62.32 | Aspartic protease inhibitor 5 |
| PGSC0003DMT400025543 | 9.43 | DOWN | 5.29 | DNAJ |
| PGSC0003DMT400025792 | 1.65 | DOWN | 3.17 | Ornithine decarboxylase |
| PGSC0003DMT400029422 | 4.2 | DOWN | 6.01 | Epoxide hydrolase 1 |
| PGSC0003DMT400030650 | 8.55 | DOWN | 107.36 | SGA |
| PGSC0003DMT400030674 | 4.2 | DOWN | 30.83 | Cytochrome P-450 |
| PGSC0003DMT400030676 | 8.24 | DOWN | 62.27 | 2-oxoglutarate-dependent dioxygenase |
| PGSC0003DMT400030678 | 5.39 | DOWN | 30.63 | Cellulose synthase |
| PGSC0003DMT400033236 | 5.94 | DOWN | 57.32 | C-4 sterol methyl oxidase |
| PGSC0003DMT400033379 | 8.8 | DOWN | 27.4 | Stem-specific protein TSJT1 |
| PGSC0003DMT400036833 | 3.49 | DOWN | 4.85 | Flotillin-1 |
| PGSC0003DMT400040198 | 2.67 | DOWN | 7.77 | Beta-ketoacyl-coa synthase family protein |
| PGSC0003DMT400041444 | 6.82 | DOWN | 7.82 | Conserved gene of unknown function |
| PGSC0003DMT400043054 | 1.92 | DOWN | 3.6 | Chlorophyll a-b binding protein 50, chloroplastic |
| PGSC0003DMT400043474 | 2.25 | DOWN | 6.68 | Gene of unknown function |
| PGSC0003DMT400046955 | 1.98 | DOWN | 7.02 | Hydrolase |
| PGSC0003DMT400048713 | 2.96 | DOWN | 4.93 | Proteinase inhibitor I4, serpin |
| PGSC0003DMT400052998 | 7.4 | DOWN | 8.14 | Avr9/Cf-9 rapidly elicited protein 216 |
| PGSC0003DMT400054413 | 4.87 | DOWN | 7.73 | Conserved gene of unknown function |
| PGSC0003DMT400055828 | 4.84 | DOWN | 17.21 | E3 ubiquitin-protein ligase RMA1H1 |
| PGSC0003DMT400055830 | 21.91 | DOWN | 4.86 | E3 ubiquitin-protein ligase RMA1H1 |
| PGSC0003DMT400057819 | 3.05 | DOWN | 14.33 | Conserved gene of unknown function |
| PGSC0003DMT400062385 | 1.96 | DOWN | 5.21 | Gamma aminobutyrate transaminase isoform2 |
| PGSC0003DMT400066798 | 2.22 | DOWN | 4.3 | 14 kDa proline-rich protein DC2.15 |
| PGSC0003DMT400067881 | 2.36 | DOWN | 17.28 | Delta(7)-sterol-C5(6)-desaturase |
| PGSC0003DMT400069577 | 2.88 | DOWN | 4.85 | UPF0497 membrane protein |
| PGSC0003DMT400070176 | 1.71 | DOWN | 3.76 | Mg protoporphyrin IX chelatase |
| PGSC0003DMT400073263 | 5.08 | DOWN | 3.6 | Trehalose-6-phosphate synthase |
| PGSC0003DMT400074969 | 2.18 | DOWN | 6.39 | Conserved gene of unknown function |
| PGSC0003DMT400076251 | 2.32 | DOWN | 8.1 | Pectase lyase |
| PGSC0003DMT400077388 | 2.99 | DOWN | 8.15 | Conserved gene of unknown function |
| PGSC0003DMT400079602 | 3.43 | DOWN | 32.82 | Polygalacturonase-1 non-catalytic subunit beta |
| PGSC0003DMT400080548 | 2.06 | DOWN | 5.86 | Phenylalanine ammonia-lyase |
| PGSC0003DMT400083158 | 5.29 | DOWN | 63.23 | Chlorophyll a/b-binding protein (cab-12) |
| PGSC0003DMT400083215 | 12.31 | DOWN | 30.02 | Basic 7S globulin 2 small subunit |
| PGSC0003DMT400083218 | 2.11 | DOWN | 7.63 | Aspartate kinase |
| PGSC0003DMT400088761 | 4.08 | DOWN | 8.05 | Subtilisin-like protease |
| PGSC0003DMT400092927 | 1.67 | DOWN | 6.64 | Chlorophyll a/b binding protein |

**Supplemental** **Table 4B** List of the 9 genes up regulated after Lso treatment.

| **Transcript** | **Fold Change in Waneta** | **Direction** | **Fold Change in Atlantic** | **Gene Description** |
| --- | --- | --- | --- | --- |
| PGSC0003DMT400020581 | 2.39 | UP | **26.87** | Conserved gene of unknown function |
| PGSC0003DMT400001573 | 2.01 | UP | 4.42 | Pseudo-response regulator 5 |
| PGSC0003DMT400003078 | 1.79 | UP | 6.29 | EARLY flowering 4 protein |
| PGSC0003DMT400008367 | 1.73 | UP | 3.9 | Conserved gene of unknown function |
| PGSC0003DMT400027199 | 1.86 | UP | 5.13 | Glucan endo-1,3-beta-D-glucosidase |
| PGSC0003DMT400051416 | 1.9 | UP | 6.07 | Circadian clock-associated FKF1 |
| PGSC0003DMT400053731 | 1.75 | UP | 4.28 | Conserved gene of unknown function |
| PGSC0003DMT400065377 | 1.97 | UP | 3.69 | Zinc finger protein CONSTANS-LIKE 15 |
| PGSC0003DMT400072092 | 1.91 | UP | 5.08 | Pectin methylesterase inhibitor isoform |

**Supplemental** **Table 4C** list of the 5 genes up regulated in Waneta and down regulated in Atlantic

| **Transcript** | **Fold Change in Waneta** | **Direction in Waneta** | **Fold Change in Atlantic** | **Direction In Atlantic** | **Gene Description** |
| --- | --- | --- | --- | --- | --- |
| PGSC0003DMT400063689 | 2.82 | UP | 49.43 | DOWN | Xyloglucan endotransglucosylase/hydrolase 1 |
| PGSC0003DMT400009055 | 3.03 | UP | 19.24 | DOWN | Endo-beta-mannanase |
| PGSC0003DMT400003166 | 1.97 | UP | 10.37 | DOWN | Leucine-rich repeat protein |
| PGSC0003DMT400012809 | 3.45 | UP | 9.03 | DOWN | Endo-beta-1,4-glucanase |
| PGSC0003DMT400054066 | 3.6 | UP | 8.69 | DOWN | Flavonol 4'-sulfotransferase |

**Supplemental** **Table 4 D** list of the 36 genes down regulated in Waneta and up regulated in Atlantic.

| **Transcript** | **Fold Change in Waneta** | **Direction in Waneta** | **Fold Change in Atlantic** | **Direction in Atlantic** | **Gene Description** |
| --- | --- | --- | --- | --- | --- |
| PGSC0003DMT400007537 | 2.77 | DOWN | 4.71 | UP | Cf-2.1 |
| PGSC0003DMT400008346 | 14.66 | DOWN | 3.02 | UP | Avr9/Cf-9 rapidly elicited protein 65 |
| PGSC0003DMT400008416 | 7.52 | DOWN | 4.53 | UP | Conserved gene of unknown function |
| PGSC0003DMT400010170 | 5.17 | DOWN | 2.92 | UP | ATP binding protein |
| PGSC0003DMT400014068 | 9.91 | DOWN | 8.77 | UP | Ascorbate oxidase |
| PGSC0003DMT400014928 | 4.26 | DOWN | 3.74 | UP | Potassium channel tetramerization domain-containing protein |
| PGSC0003DMT400014949 | 7.85 | DOWN | 5.94 | UP | WRKY transcription factor-30 |
| PGSC0003DMT400015091 | 15.04 | DOWN | 3.94 | UP | CCR4-associated factor |
| PGSC0003DMT400015811 | 1.95 | DOWN | 4.35 | UP | Nodulin family protein |
| PGSC0003DMT400017780 | 4.99 | DOWN | 4.73 | UP | Gene of unknown function |
| PGSC0003DMT400021177 | 6.03 | DOWN | 3.39 | UP | Calmodulin binding protein |
| PGSC0003DMT400025786 | 3.93 | DOWN | 3.87 | UP | Syringolide-induced protein 14-1-1 |
| PGSC0003DMT400027849 | 2.57 | DOWN | 3.06 | UP | ERF transcription factor 4 |
| PGSC0003DMT400027937 | 11.46 | DOWN | 3.29 | UP | Ethylene response factor 5 |
| PGSC0003DMT400030390 | 13.23 | DOWN | 6.19 | UP | WRKY-type transcription factor |
| PGSC0003DMT400031803 | 8.69 | DOWN | 3.02 | UP | Potassium channel tetramerization domain-containing protein |
| PGSC0003DMT400038283 | 7.6 | DOWN | 5.67 | UP | Tyramine hydroxycinnamoyl transferase |
| PGSC0003DMT400044683 | 2.64 | DOWN | 6.35 | UP | Heat shock factor protein |
| PGSC0003DMT400047537 | 8.56 | DOWN | 3.69 | UP | ABA 8'-hydroxylase CYP707A1 |
| PGSC0003DMT400054965 | 7.64 | DOWN | 4.07 | UP | PEN1 |
| PGSC0003DMT400057463 | 2.07 | DOWN | 4.34 | UP | Zinc finger protein |
| PGSC0003DMT400058621 | 18.39 | DOWN | 7.28 | UP | CMPG1b |
| PGSC0003DMT400064403 | 4.67 | DOWN | 3.94 | UP | Avr9/Cf-9 rapidly elicited protein 20 |
| PGSC0003DMT400064404 | 6.95 | DOWN | 4.11 | UP | Avr9/Cf-9 rapidly elicited protein 20 |
| PGSC0003DMT400071761 | 8.04 | DOWN | 8.96 | UP | Ascorbate oxidase |
| PGSC0003DMT400072840 | 9.66 | DOWN | 4.13 | UP | Gene of unknown function |
| PGSC0003DMT400074709 | 5.84 | DOWN | 4.36 | UP | Aromatic amino acid decarboxylase 1B |
| PGSC0003DMT400078065 | 3.01 | DOWN | 3.62 | UP | Avr9/Cf-9 rapidly elicited protein 284 |
| PGSC0003DMT400080765 | 12.17 | DOWN | 3.58 | UP | Phenylalanine ammonia-lyase 1 |
| PGSC0003DMT400082122 | 8.77 | DOWN | 3.26 | UP | N-acetyltransferase |
| PGSC0003DMT400082749 | 3.29 | DOWN | 8.69 | UP | Calmodulin-binding protein |
| PGSC0003DMT400083857 | 3.3 | DOWN | 3.12 | UP | Cf-2.2 |
| PGSC0003DMT400085907 | 2.48 | DOWN | 3.03 | UP | Conserved gene of unknown function |
| PGSC0003DMT400086995 | 12.27 | DOWN | 4.09 | UP | Ethylene response factor 5 |
| PGSC0003DMT400090475 | 9.08 | DOWN | 3.84 | UP | Ethylene response factor 5 |
| PGSC0003DMT400091831 | 6.79 | DOWN | 3.09 | UP | Basic 7S globulin 2 small subunit |

**Suplemental Table** **5:** Gprofiler results for Go enrichment in the genes commonly regulated **Suplemental Table 5A:** Go Term enrichment for the 70 genes regulated in the same direction. T-type BP: Biological Process, CC

| p-value | term ID | t type | t name and depth in group Q&T list |
| --- | --- | --- | --- |
| 7.21E-11 | GO:0018298 | BP | protein-chromophore linkage 1 PGSC0003DMT400092927,PGSC0003DMT400083158,PGSC0003DMT400020100,PGSC0003DMT400021389,PGSC0003DMT400021391,PGSC0003DMT400022072,PGSC0003DMT400010985,PGSC0003DMT400043054 |
| 5.00E-02 | GO:0016053 | BP | organic acid biosynthetic process 1 PGSC0003DMT400080548,PGSC0003DMT400083218,PGSC0003DMT400001709,PGSC0003DMT400005519,PGSC0003DMT400033236,PGSC0003DMT400067881,PGSC0003DMT400040198 |
| 5.00E-02 | GO:0046394 | BP | carboxylic acid biosynthetic process 2 PGSC0003DMT400080548,PGSC0003DMT400083218,PGSC0003DMT400001709,PGSC0003DMT400005519,PGSC0003DMT400033236,PGSC0003DMT400067881,PGSC0003DMT400040198 |
| 1.16E-02 | GO:0072330 | BP | monocarboxylic acid biosynthetic process 3 PGSC0003DMT400080548,PGSC0003DMT400001709,PGSC0003DMT400005519,PGSC0003DMT400033236,PGSC0003DMT400067881,PGSC0003DMT400040198 |
| 1.06E-02 | GO:0006633 | BP | fatty acid biosynthetic process 4 PGSC0003DMT400001709,PGSC0003DMT400005519,PGSC0003DMT400033236,PGSC0003DMT400067881,PGSC0003DMT400040198 |
| 6.20E-06 | GO:0006091 | BP | generation of precursor metabolites and energy 1 PGSC0003DMT400092927,PGSC0003DMT400083158,PGSC0003DMT400020100,PGSC0003DMT400021389,PGSC0003DMT400021391,PGSC0003DMT400022072,PGSC0003DMT400008103,PGSC0003DMT400010985,PGSC0003DMT400070176,PGSC0003DMT400043054 |
| 6.14E-06 | GO:0015979 | BP | photosynthesis 1 PGSC0003DMT400092927,PGSC0003DMT400083158,PGSC0003DMT400020100,PGSC0003DMT400021389,PGSC0003DMT400021391,PGSC0003DMT400022072,PGSC0003DMT400010985,PGSC0003DMT400070176,PGSC0003DMT400043054 |
| 5.08E-07 | GO:0019684 | BP | photosynthesis, light reaction 2 PGSC0003DMT400092927,PGSC0003DMT400083158,PGSC0003DMT400020100,PGSC0003DMT400021389,PGSC0003DMT400021391,PGSC0003DMT400022072,PGSC0003DMT400010985,PGSC0003DMT400070176,PGSC0003DMT400043054 |
| 7.21E-11 | GO:0009765 | BP | photosynthesis, light harvesting 3 PGSC0003DMT400092927,PGSC0003DMT400083158,PGSC0003DMT400020100,PGSC0003DMT400021389,PGSC0003DMT400021391,PGSC0003DMT400022072,PGSC0003DMT400010985,PGSC0003DMT400043054 |
| 1.27E-02 | GO:0009536 | CC | plastid 1 PGSC0003DMT400092927,PGSC0003DMT400074969,PGSC0003DMT400083158,PGSC0003DMT400020100,PGSC0003DMT400021389,PGSC0003DMT400021391,PGSC0003DMT400022072,PGSC0003DMT400010985,PGSC0003DMT400070176,PGSC0003DMT400043054 |
| 8.90E-03 | GO:0009507 | CC | chloroplast 2 PGSC0003DMT400092927,PGSC0003DMT400074969,PGSC0003DMT400083158,PGSC0003DMT400020100,PGSC0003DMT400021389,PGSC0003DMT400021391,PGSC0003DMT400022072,PGSC0003DMT400010985,PGSC0003DMT400070176,PGSC0003DMT400043054 |
| 9.23E-03 | GO:0016020 | CC | membrane 1 PGSC0003DMT400092927,PGSC0003DMT400015184,PGSC0003DMT400083158,PGSC0003DMT400020100,PGSC0003DMT400021389,PGSC0003DMT400021391,PGSC0003DMT400022072,PGSC0003DMT400030678,PGSC0003DMT400001709,PGSC0003DMT400003608,PGSC0003DMT400005519,PGSC0003DMT400033236,PGSC0003DMT400010643,PGSC0003DMT400010957,PGSC0003DMT400010985,PGSC0003DMT400067881,PGSC0003DMT400069577,PGSC0003DMT400070176,PGSC0003DMT400040198,PGSC0003DMT400043054 |
| 3.27E-02 | GO:0044425 | CC | membrane part 2 PGSC0003DMT400092927,PGSC0003DMT400083158,PGSC0003DMT400020100,PGSC0003DMT400021389,PGSC0003DMT400021391,PGSC0003DMT400022072,PGSC0003DMT400030678,PGSC0003DMT400001709,PGSC0003DMT400005519,PGSC0003DMT400033236,PGSC0003DMT400010643,PGSC0003DMT400010985,PGSC0003DMT400067881,PGSC0003DMT400069577,PGSC0003DMT400043054 |
| 6.02E-03 | GO:0031224 | CC | intrinsic component of membrane 3 PGSC0003DMT400092927,PGSC0003DMT400083158,PGSC0003DMT400020100,PGSC0003DMT400021389,PGSC0003DMT400021391,PGSC0003DMT400022072,PGSC0003DMT400030678,PGSC0003DMT400001709,PGSC0003DMT400005519,PGSC0003DMT400033236,PGSC0003DMT400010643,PGSC0003DMT400010985,PGSC0003DMT400067881,PGSC0003DMT400069577,PGSC0003DMT400043054 |
| 4.21E-03 | GO:0016021 | CC | integral component of membrane 4 PGSC0003DMT400092927,PGSC0003DMT400083158,PGSC0003DMT400020100,PGSC0003DMT400021389,PGSC0003DMT400021391,PGSC0003DMT400022072,PGSC0003DMT400030678,PGSC0003DMT400001709,PGSC0003DMT400005519,PGSC0003DMT400033236,PGSC0003DMT400010643,PGSC0003DMT400010985,PGSC0003DMT400067881,PGSC0003DMT400069577,PGSC0003DMT400043054 |
| 2.72E-04 | GO:0098796 | CC | membrane protein complex 1 PGSC0003DMT400092927,PGSC0003DMT400083158,PGSC0003DMT400020100,PGSC0003DMT400021389,PGSC0003DMT400021391,PGSC0003DMT400022072,PGSC0003DMT400010985,PGSC0003DMT400043054 |
| 1.44E-05 | GO:0009579 | CC | thylakoid 1 PGSC0003DMT400092927,PGSC0003DMT400074969,PGSC0003DMT400083158,PGSC0003DMT400020100,PGSC0003DMT400021389,PGSC0003DMT400021391,PGSC0003DMT400022072,PGSC0003DMT400010985,PGSC0003DMT400043054 |
| 2.65E-05 | GO:0044436 | CC | thylakoid part 2 PGSC0003DMT400092927,PGSC0003DMT400083158,PGSC0003DMT400020100,PGSC0003DMT400021389,PGSC0003DMT400021391,PGSC0003DMT400022072,PGSC0003DMT400010985,PGSC0003DMT400043054 |
| 1.49E-05 | GO:0034357 | CC | photosynthetic membrane 2 PGSC0003DMT400092927,PGSC0003DMT400083158,PGSC0003DMT400020100,PGSC0003DMT400021389,PGSC0003DMT400021391,PGSC0003DMT400022072,PGSC0003DMT400010985,PGSC0003DMT400043054 |
| 2.31E-08 | GO:0009521 | CC | photosystem 3 PGSC0003DMT400092927,PGSC0003DMT400083158,PGSC0003DMT400020100,PGSC0003DMT400021389,PGSC0003DMT400021391,PGSC0003DMT400022072,PGSC0003DMT400010985,PGSC0003DMT400043054 |
| 2.45E-09 | GO:0009523 | CC | photosystem II 4 PGSC0003DMT400092927,PGSC0003DMT400083158,PGSC0003DMT400020100,PGSC0003DMT400021389,PGSC0003DMT400021391,PGSC0003DMT400022072,PGSC0003DMT400010985,PGSC0003DMT400043054 |
| 1.02E-10 | GO:0009522 | CC | photosystem I 4 PGSC0003DMT400092927,PGSC0003DMT400083158,PGSC0003DMT400020100,PGSC0003DMT400021389,PGSC0003DMT400021391,PGSC0003DMT400022072,PGSC0003DMT400010985,PGSC0003DMT400043054 |
| 1.43E-02 | GO:0046906 | MF | tetrapyrrole binding 1 PGSC0003DMT400092927,PGSC0003DMT400083158,PGSC0003DMT400020100,PGSC0003DMT400021389,PGSC0003DMT400021391,PGSC0003DMT400022072,PGSC0003DMT400030674,PGSC0003DMT400010985,PGSC0003DMT400043054 |
| 8.60E-11 | GO:0016168 | MF | chlorophyll binding 2 PGSC0003DMT400092927,PGSC0003DMT400083158,PGSC0003DMT400020100,PGSC0003DMT400021389,PGSC0003DMT400021391,PGSC0003DMT400022072,PGSC0003DMT400010985,PGSC0003DMT400043054 |

**Suplemental Table 5 B:** Go Term enrichment for the 41 genes regulated in opposite direction.

| p-value | term ID | t type | t name and depth in group Q&T list |  |
| --- | --- | --- | --- | --- |
|  |  |  |  |  |
| 3.19E-02 | GO:0051260 | BP | protein homooligomerization 1 PGSC0003DMT400014928,PGSC0003DMT400031803 |  |
| 5.00E-02 | GO:0001071 | MF | nucleic acid binding transcription factor activity 1 PGSC0003DMT400086995,PGSC0003DMT400090475,PGSC0003DMT400014949,PGSC0003DMT400044683,PGSC0003DMT400027849,PGSC0003DMT400027937,PGSC0003DMT400030390 |  |
| 5.00E-02 | GO:0003700 | MF | transcription factor activity, sequence-specific DNA binding 2 PGSC0003DMT400086995,PGSC0003DMT400090475,PGSC0003DMT400014949,PGSC0003DMT400044683,PGSC0003DMT400027849,PGSC0003DMT400027937,PGSC0003DMT400030390 |  |

**Supplemental table 6:** List of photosynthesis related DEGs in Waneta and Atlantic after Lso treatment. (Transcript in mapman analysis)

| **BinCode** | **BinName** | **id** | **description** | **Fold change AT** | **fold change Waneta** |
| --- | --- | --- | --- | --- | --- |
| 1.1.1.1 | PS.lightreaction.photosystem II.LHC-II | pgsc0003dmp400007654|pacid:24379960 | moderately similar to ( 477) AT2G05100 | Symbols: LHCB2.1, LHCB2 | LHCB2.1; chlorophyll binding | chr2:1823449-1824331 REVERSEhighly similar to ( 513) CB23_TOBAC Chlorophyll a-b binding protein 36, chloroplast precursor (LHCII type I CAB-36) (LHCP) - Nicotiana tabacum (Common tobacco)moderately similar to ( 464) loc_os03g39610 12003.m35304 protein chlorophyll a-b binding protein, chloroplast precursor, putative, expressedChloroa_b-bind no original description | -14.8 | -2.84 |
| 1.1.1.1 | PS.lightreaction.photosystem II.LHC-II | pgsc0003dmp400007919|pacid:24417446 | moderately similar to ( 275) AT1G45474 | Symbols: LHCA5 | LHCA5; pigment binding | chr1:17179353-17180439 FORWARDweakly similar to ( 129) CB12_PETHY Chlorophyll a-b binding protein, chloroplast precursor (LHCI type II CAB) - Petunia hybrida (Petunia)moderately similar to ( 246) loc_os02g52650 12002.m10295 protein chlorophyll a-b binding protein 4, chloroplast precursor, putative, expressedChloroa_b-bind no original description | - | -2.15 |
| 1.1.1.1 | PS.lightreaction.photosystem II.LHC-II | pgsc0003dmp400010908|pacid:24417422 | moderately similar to ( 474) AT3G27690 | Symbols: LHCB2.4, LHCB2.3, LHCB2 | LHCB2.3; chlorophyll binding | chr3:10256002-10256921 FORWARDhighly similar to ( 502) CB23_TOBAC Chlorophyll a-b binding protein 36, chloroplast precursor (LHCII type I CAB-36) (LHCP) - Nicotiana tabacum (Common tobacco)moderately similar to ( 469) loc_os03g39610 12003.m35304 protein chlorophyll a-b binding protein, chloroplast precursor, putative, expressedChloroa_b-bind no original description | -4.44 | - |
| 1.1.1.1 | PS.lightreaction.photosystem II.LHC-II | pgsc0003dmp400013055|pacid:24392150 | moderately similar to ( 365) AT1G29930 | Symbols: CAB1, AB140, CAB140, LHCB1.3 | CAB1 (CHLOROPHYLL A/B BINDING PROTEIN 1); chlorophyll binding | chr1:10478071-10478874 FORWARDmoderately similar to ( 413) CB21_TOBAC Chlorophyll a-b binding protein 16, chloroplast precursor (LHCII type I CAB-16) (LHCP) - Nicotiana tabacum (Common tobacco)moderately similar to ( 371) loc_os01g41710 12001.m42672 protein chlorophyll a-b binding protein 2, chloroplast precursor, putative, expressedChloroa_b-bind no original description | - | -2.29 |
| 1.1.1.1 | PS.lightreaction.photosystem II.LHC-II | pgsc0003dmp400013707|pacid:24380656 | moderately similar to ( 414) AT1G61520 | Symbols: LHCA3 | LHCA3; chlorophyll binding | chr1:22700493-22701149 FORWARDweakly similar to ( 146) CB12_PETHY Chlorophyll a-b binding protein, chloroplast precursor (LHCI type II CAB) - Petunia hybrida (Petunia)moderately similar to ( 362) loc_os02g10390 12002.m33354 protein chlorophyll a-b binding protein 8, chloroplast precursor, putative, expressedChloroa_b-bind no original description | -4.54 | -2.26 |
| 1.1.1.1 | PS.lightreaction.photosystem II.LHC-II | pgsc0003dmp400014555|pacid:24428969 | moderately similar to ( 444) AT2G34430 | Symbols: LHB1B1, LHCB1.4 | LHB1B1; chlorophyll binding | chr2:14524818-14525618 FORWARDmoderately similar to ( 482) CB22_TOBAC Chlorophyll a-b binding protein 21, chloroplast precursor (LHCII type I CAB-21) (LHCP) - Nicotiana tabacum (Common tobacco)moderately similar to ( 436) loc_os01g52240 12001.m11398 protein chlorophyll a-b binding protein 2, chloroplast precursor, putative, expressedChloroa_b-bind no original description | -13.88 | -2.91 |
| 1.1.1.1 | PS.lightreaction.photosystem II.LHC-II | pgsc0003dmp400014556|pacid:24427169 | moderately similar to ( 438) AT2G34430 | Symbols: LHB1B1, LHCB1.4 | LHB1B1; chlorophyll binding | chr2:14524818-14525618 FORWARDmoderately similar to ( 477) CB22_TOBAC Chlorophyll a-b binding protein 21, chloroplast precursor (LHCII type I CAB-21) (LHCP) - Nicotiana tabacum (Common tobacco)moderately similar to ( 429) loc_os01g52240 12001.m11398 protein chlorophyll a-b binding protein 2, chloroplast precursor, putative, expressedChloroa_b-bind no original description | -6.04 | - |
| 1.1.1.1 | PS.lightreaction.photosystem II.LHC-II | pgsc0003dmp400014557|pacid:24426445 | moderately similar to ( 437) AT1G29930 | Symbols: CAB1, AB140, CAB140, LHCB1.3 | CAB1 (CHLOROPHYLL A/B BINDING PROTEIN 1); chlorophyll binding | chr1:10478071-10478874 FORWARDmoderately similar to ( 478) CB22_TOBAC Chlorophyll a-b binding protein 21, chloroplast precursor (LHCII type I CAB-21) (LHCP) - Nicotiana tabacum (Common tobacco)moderately similar to ( 432) loc_os01g52240 12001.m11398 protein chlorophyll a-b binding protein 2, chloroplast precursor, putative, expressedChloroa_b-bind no original description | -7.24 | -1.99 |
| 1.1.1.1 | PS.lightreaction.photosystem II.LHC-II | pgsc0003dmp400014558|pacid:24427843 | moderately similar to ( 447) AT1G29930 | Symbols: CAB1, AB140, CAB140, LHCB1.3 | CAB1 (CHLOROPHYLL A/B BINDING PROTEIN 1); chlorophyll binding | chr1:10478071-10478874 FORWARDmoderately similar to ( 492) CB22_TOBAC Chlorophyll a-b binding protein 21, chloroplast precursor (LHCII type I CAB-21) (LHCP) - Nicotiana tabacum (Common tobacco)moderately similar to ( 440) loc_os01g41710 12001.m42672 protein chlorophyll a-b binding protein 2, chloroplast precursor, putative, expressedChloroa_b-bind no original description | - | -1.83 |
| 1.1.1.1 | PS.lightreaction.photosystem II.LHC-II | pgsc0003dmp400014577|pacid:24426556 | moderately similar to ( 447) AT1G29930 | Symbols: CAB1, AB140, CAB140, LHCB1.3 | CAB1 (CHLOROPHYLL A/B BINDING PROTEIN 1); chlorophyll binding | chr1:10478071-10478874 FORWARDmoderately similar to ( 487) CB22_TOBAC Chlorophyll a-b binding protein 21, chloroplast precursor (LHCII type I CAB-21) (LHCP) - Nicotiana tabacum (Common tobacco)moderately similar to ( 439) loc_os01g52240 12001.m11398 protein chlorophyll a-b binding protein 2, chloroplast precursor, putative, expressedChloroa_b-bind no original description | -6.38 | - |
| 1.1.1.1 | PS.lightreaction.photosystem II.LHC-II | pgsc0003dmp400015035|pacid:24381198 | moderately similar to ( 460) AT5G54270 | Symbols: LHCB3, LHCB3*1 | LHCB3 (LIGHT-HARVESTING CHLOROPHYLL B-BINDING PROTEIN 3); structural molecule | chr5:22038424-22039383 FORWARDmoderately similar to ( 451) CB23_HORVU Chlorophyll a-b binding protein of LHCII type III, chloroplast precursor (CAB) - Hordeum vulgare (Barley)moderately similar to ( 455) loc_os07g37550 12007.m079717 protein chlorophyll a-b binding protein of LHCII type III, chloroplast precursor, putative, expressedChloroa_b-bind no original description | -5.43 | -3.22 |
| 1.1.1.1 | PS.lightreaction.photosystem II.LHC-II | pgsc0003dmp400022301|pacid:24420299 | moderately similar to ( 369) AT1G15820 | Symbols: LHCB6, CP24 | LHCB6 (LIGHT HARVESTING COMPLEX PSII SUBUNIT 6); chlorophyll binding | chr1:5446685-5447676 REVERSEmoderately similar to ( 367) CB4_SPIOL Chlorophyll a-b binding protein CP24, chloroplast precursor - Spinacia oleracea (Spinach)moderately similar to ( 346) loc_os04g38410 12004.m08831 protein chlorophyll a-b binding protein CP24, chloroplast precursor, putative, expressedChloroa_b-bind no original description | -3.13 | - |
| 1.1.1.1 | PS.lightreaction.photosystem II.LHC-II | pgsc0003dmp400023739|pacid:24403722 | moderately similar to ( 446) AT1G29930 | Symbols: CAB1, AB140, CAB140, LHCB1.3 | CAB1 (CHLOROPHYLL A/B BINDING PROTEIN 1); chlorophyll binding | chr1:10478071-10478874 FORWARDmoderately similar to ( 489) CB25_TOBAC Chlorophyll a-b binding protein 50, chloroplast precursor (LHCII type I CAB-50) (LHCP) - Nicotiana tabacum (Common tobacco)moderately similar to ( 446) loc_os01g41710 12001.m42672 protein chlorophyll a-b binding protein 2, chloroplast precursor, putative, expressedChloroa_b-bind no original description | -3.45 | - |
| 1.1.1.1 | PS.lightreaction.photosystem II.LHC-II | pgsc0003dmp400023813|pacid:24403383 | moderately similar to ( 291) AT2G34430 | Symbols: LHB1B1, LHCB1.4 | LHB1B1; chlorophyll binding | chr2:14524818-14525618 FORWARDmoderately similar to ( 314) CB2A_SPIOL Chlorophyll a-b binding protein, chloroplast precursor (LHCII type I CAB) (LHCP) - Spinacia oleracea (Spinach)moderately similar to ( 308) loc_os01g52240 12001.m11398 protein chlorophyll a-b binding protein 2, chloroplast precursor, putative, expressedChloroa_b-bind no original description | -4.02 | - |
| 1.1.1.1 | PS.lightreaction.photosystem II.LHC-II | pgsc0003dmp400040356|pacid:24386769 | moderately similar to ( 360) AT3G54890 | Symbols: LHCA1 | LHCA1; chlorophyll binding | chr3:20339881-20340922 REVERSEweakly similar to ( 120) CB21_SINAL Chlorophyll a-b binding protein 1, chloroplast precursor (LHCII type I CAB-1) (LHCP) - Sinapis alba (White mustard) (Brassica hirta)moderately similar to ( 345) loc_os06g21590 12006.m32064 protein chlorophyll a-b binding protein 6A, chloroplast precursor, putative, expressedChloroa_b-bind no original description | - | -2.06 |
| 1.1.1.1 | PS.lightreaction.photosystem II.LHC-II | pgsc0003dmp400055858|pacid:24394945 | moderately similar to ( 437) AT3G47470 | Symbols: LHCA4, CAB4 | LHCA4 (LIGHT-HARVESTING CHLOROPHYLL-PROTEIN COMPLEX I SUBUNIT A4); chlorophyll binding | chr3:17493622-17494773 REVERSEmoderately similar to ( 226) CB12_PETHY Chlorophyll a-b binding protein, chloroplast precursor (LHCI type II CAB) - Petunia hybrida (Petunia)moderately similar to ( 387) loc_os08g33820 12008.m080044 protein chlorophyll a-b binding protein 4, chloroplast precursor, putative, expressedChloroa_b-bind no original description | -63.23 | -5.29 |
| 1.1.1.1 | PS.lightreaction.photosystem II.LHC-II | pgsc0003dmp400064602|pacid:24428847 | moderately similar to ( 444) AT1G29930 | Symbols: CAB1, AB140, CAB140, LHCB1.3 | CAB1 (CHLOROPHYLL A/B BINDING PROTEIN 1); chlorophyll binding | chr1:10478071-10478874 FORWARDmoderately similar to ( 485) CB22_TOBAC Chlorophyll a-b binding protein 21, chloroplast precursor (LHCII type I CAB-21) (LHCP) - Nicotiana tabacum (Common tobacco)moderately similar to ( 436) loc_os01g52240 12001.m11398 protein chlorophyll a-b binding protein 2, chloroplast precursor, putative, expressedChloroa_b-bind no original description | -6.64 | -1.67 |
| 1.1.1.2 | PS.lightreaction.photosystem II.PSII polypeptide subunits | pgsc0003dmp400034952|pacid:24397056 | weakly similar to ( 126) AT2G30570 | Symbols: PSBW | PSBW (PHOTOSYSTEM II REACTION CENTER W) | chr2:13019326-13020053 REVERSEweakly similar to ( 116) PSBW_SPIOL Photosystem II reaction center W protein, chloroplast precursor (PSII 6.1 kDa protein) - Spinacia oleracea (Spinach)PsbW no original description | - | -1.76 |
| 1.1.2.2 | PS.lightreaction.photosystem I.PSI polypeptide subunits | pgsc0003dmp400010291|pacid:24390309 | weakly similar to ( 188) AT5G64040 | Symbols: PSAN | PSAN; calmodulin binding | chr5:25628690-25629409 REVERSEweakly similar to ( 155) PSAN_MAIZE Photosystem I reaction center subunit N, chloroplast precursor (PSI-N) (Fragment) - Zea mays (Maize)weakly similar to ( 164) loc_os12g08770 12012.m04863 protein photosystem I reaction center subunit N, chloroplast precursor, putative, expressedPsaN no original description | - | -1.88 |
| 1.1.2.2 | PS.lightreaction.photosystem I.PSI polypeptide subunits | pgsc0003dmp400035620|pacid:24391361 | weakly similar to ( 170) AT1G30380 | Symbols: PSAK | PSAK (photosystem I subunit K) | chr1:10722325-10723013 FORWARDweakly similar to ( 156) PSAK_MEDSA Photosystem I reaction center subunit psaK, chloroplast precursor (Photosystem I subunit X) (PSI-K) - Medicago sativa (Alfalfa)weakly similar to ( 124) loc_os07g05480 12007.m079603 protein photosystem I reaction center subunit psaK, chloroplast precursor, putative, expressedPSI_PSAK no original description | - | -2.35 |
| 1.1.4.4 | PS.lightreaction.ATP synthase.gamma chain | pgsc0003dmp400048475|pacid:24378809 | moderately similar to ( 450) AT4G04640 | Symbols: ATPC1 | ATPC1; enzyme regulator | chr4:2350761-2351882 REVERSEmoderately similar to ( 471) ATPG_TOBAC ATP synthase gamma chain, chloroplast precursor (EC 3.6.3.14) - Nicotiana tabacum (Common tobacco)moderately similar to ( 434) loc_os07g32880 12007.m07550 protein ATP synthase gamma chain, chloroplast precursor, putative, expressedATP-synt PRK05621 AtpG no original description | - | 2.05 |
| 1.1.40 | PS.lightreaction.cyclic electron flow-chlororespiration | pgsc0003dmp400015179|pacid:24406197 | moderately similar to ( 376) AT1G64770 | Symbols: NDF2 | NDF2 (NDH-DEPENDENT CYCLIC ELECTRON FLOW 1) | chr1:24057549-24058565 FORWARDmoderately similar to ( 357) loc_os03g06230 12003.m06143 protein expressed protein no original description | - | -1.71 |
| 1.1.5.2 | PS.lightreaction.other electron carrier (ox/red).ferredoxin | pgsc0003dmp400021623|pacid:24390430 | moderately similar to ( 204) AT3G16250 | Symbols: NDF4 | NDF4 (NDH-DEPENDENT CYCLIC ELECTRON FLOW 1); electron carrier/ iron-sulfur cluster binding | chr3:5507091-5508320 REVERSEweakly similar to ( 184) loc_os07g30670 12007.m079681 protein electron carrier/ electron transporter/ iron ion binding protein, putative, expressed no original description | - | -1.7 |

**Supplemental table 7:** List of carbohydrates metabolism DEGs in Waneta and Atlantic after Lso treatment. (mapman analysis)

| **BinCode** | **BinName** | **id** | **description** | **Fold change AT** | **Fold change Waneta** |
| --- | --- | --- | --- | --- | --- |
| 3.1.1.2 | minor CHO metabolism.raffinose family.galactinol synthases.putative | pgsc0003dmp400006276|pacid:24427350 | highly similar to ( 537) AT2G47180 | Symbols: AtGolS1 | AtGolS1 (Arabidopsis thaliana galactinol synthase 1); transferase, transferring glycosyl groups / transferase, transferring hexosyl groups | chr2:19369049-19370372 REVERSEhighly similar to ( 518) loc_os03g20120 12003.m07424 protein galactinol synthase 3, putative, expressedGlyco_transf_8 no original description | 3.17 | - |
| 3.2.1 | minor CHO metabolism.trehalose.TPS | pgsc0003dmp400047742|pacid:24415559 | nearly identical (1154) AT2G18700 | Symbols: ATTPS11, TPS11, ATTPSB | ATTPS11; transferase, transferring glycosyl groups | chr2:8109043-8111799 FORWARDnearly identical (1041) loc_os02g54820 12002.m33906 protein trehalose-6-phosphate synthase, putative, expressedGlyco_transf_20 OtsA Trehalose_PPase PRK10117 OtsB no original description | - | -2.06 |
| 3.2.3 | minor CHO metabolism.trehalose.potential TPS/TPP | pgsc0003dmp400049556|pacid:24425038 | nearly identical (1359) AT1G06410 | Symbols: ATTPS7, TPS7, ATTPSA | ATTPS7; alpha,alpha-trehalose-phosphate synthase (UDP-forming)/ transferase, transferring glycosyl groups / trehalose-phosphatase | chr1:1955413-1958153 FORWARDnearly identical (1287) loc_os01g54560 12001.m150713 protein trehalose synthase, putative, expressedGlyco_transf_20 OtsA Trehalose_PPase PRK10117 OtsB no original description | -3.6 | -5.08 |
| 3.4.3 | minor CHO metabolism.myo-inositol.InsP Synthases | pgsc0003dmp400048544|pacid:24382266 | highly similar to ( 933) AT5G10170 | Symbols: ATMIPS3, MIPS3 | MIPS3 (MYO-INOSITOL-1-PHOSTPATE SYNTHASE 3); binding / catalytic/ inositol-3-phosphate synthase | chr5:3187538-3190161 REVERSEhighly similar to ( 988) INO1_TOBAC Inositol-3-phosphate synthase (EC 5.5.1.4) (Myo-inositol-1-phosphate synthase) (MI-1-P synthase) (IPS) - Nicotiana tabacum (Common tobacco)highly similar to ( 913) loc_os03g09250 12003.m35064 protein inositol-3-phosphate synthase, putative, expressedNAD_binding_5 INO1 Inos-1-P_synth no original description | -3.79 | - |
| 4.1.12 | glycolysis.cytosolic branch.phosphoglycerate mutase | pgsc0003dmp400049761|pacid:24421585 | moderately similar to ( 412) AT3G05170 | Symbols: | phosphoglycerate/bisphosphoglycerate mutase family protein | chr3:1466738-1468219 FORWARDmoderately similar to ( 377) loc_os05g04960 12005.m05025 protein glycerolphosphate mutase, putative, expressedphoE PGAM no original description | - | -2.54 |
| 5.10 | fermentation.aldehyde dehydrogenase | pgsc0003dmp400008325|pacid:24380598 | nearly identical (1022) AT3G66658 | Symbols: ALDH22a1 | ALDH22a1 (Aldehyde Dehydrogenase 22a1); 3-chloroallyl aldehyde dehydrogenase/ oxidoreductase | chr3:2095471-2099013 REVERSEmoderately similar to ( 223) BADH_SPIOL Betaine-aldehyde dehydrogenase, chloroplast precursor (EC 1.2.1.8) (BADH) - Spinacia oleracea (Spinach)nearly identical (1041) loc_os07g48920 12007.m09101 protein betaine-aldehyde dehydrogenase, chloroplast precursor, putative, expressedAldedh PutA gabD2 PRK10090 gabD1 gabD PRK09847 PRK03137 PRK11904 PRK11905 COG4230 astD putA no original description | -5.82 | - |
| 6.2 | gluconeogenesis / glyoxylate cycle.malate synthase | pgsc0003dmp400026656|pacid:24405949 | highly similar to ( 945) AT5G03860 | Symbols: MLS | MLS (MALATE SYNTHASE); malate synthase | chr5:1032276-1034527 REVERSEnearly identical (1002) MASY_RICCO Malate synthase, glyoxysomal (EC 2.3.3.9) - Ricinus communis (Castor bean)highly similar to ( 785) loc_os04g40990 12004.m09079 protein malate synthase, glyoxysomal, putative, expressedmalate_synt_A PRK09255 Malate_synthase malate_synt AceB no original description | -10.91 | - |
| 7.2.4 | OPP.non-reductive PP.ribose 5-phosphate isomerase | pgsc0003dmp400053078|pacid:24388129 | moderately similar to ( 344) AT1G71100 | Symbols: RSW10 | RSW10 (RADIAL SWELLING 10); ribose-5-phosphate isomerase | chr1:26814726-26815529 FORWARDmoderately similar to ( 288) loc_os07g08030 12007.m05261 protein ribose-5-phosphate isomerase, putative, expressedRPI_A PRK00702 RpiA Rib_5-P_isom_A no original description | - | -2.99 |
| 8.2.11 | TCA / org transformation.other organic acid transformatons.atp-citrate lyase | pgsc0003dmp400023850|pacid:24388686 | highly similar to ( 751) AT1G60810 | Symbols: ACLA-2 | ACLA-2; ATP citrate synthase | chr1:22388691-22390993 REVERSEhighly similar to ( 717) loc_os12g37870 12012.m07573 protein ATP-citrate synthase, putative, expressedSucC sucC no original description | -8.55 | - |

**Supplemental table 8:** List of lipid metabolism DEGs in Waneta and Atlantic after Lso treatment. (mapman analysis)

| **BinCode** | **Biname** | **ID** | **descirption** | Fold Change in AT | Fold Change in Waneta |
| --- | --- | --- | --- | --- | --- |
| 11.3 | lipid metabolism.Phospholipid synthesis | pgsc0003dmp400025476|pacid:24380046 | highly similar to ( 798) AT3G18000 | Symbols: NMT1, XPL1, PEAMT | XPL1 (XIPOTL 1); methyltransferase/ phosphoethanolamine N-methyltransferase | chr3:6154578-6157331 FORWARDhighly similar to ( 798) PEAM1_ARATH Phosphoethanolamine N-methyltransferase 1 (EC 2.1.1.103) (PEAMT 1) (AtNMT1) - Arabidopsis thaliana (Mouse-ear cress)highly similar to ( 791) loc_os01g50030 12001.m11187 protein phosphoethanolamine N-methyltransferase, putative, expressedCfa UbiE Methyltransf_11 PRK08317 ubiE no original description | -5.43 | - |
| 11.3 | lipid metabolism.Phospholipid synthesis | pgsc0003dmp400030233|pacid:24415029 | highly similar to ( 578) AT1G06520 | Symbols: ATGPAT1, GPAT1 | GPAT1 (GLYCEROL-3-PHOSPHATE ACYLTRANSFERASE 1); 1-acylglycerol-3-phosphate O-acyltransferase/ acyltransferase | chr1:1994170-1996067 REVERSEmoderately similar to ( 484) loc_os01g44069 12001.m10665 protein glycerol-3-phosphate acyltransferase 1, putative, expressed no original description | - | -9.62 |
| 11.3 | lipid metabolism.Phospholipid synthesis | pgsc0003dmp400035276|pacid:24401933 | highly similar to ( 703) AT2G38110 | Symbols: ATGPAT6, GPAT6 | GPAT6 (GLYCEROL-3-PHOSPHATE ACYLTRANSFERASE 6); 1-acylglycerol-3-phosphate O-acyltransferase/ acyltransferase | chr2:15952816-15955364 REVERSEhighly similar to ( 653) loc_os01g63580 12001.m12474 protein glycerol-3-phosphate acyltransferase 8, putative, expressed no original description | -6.39 | - |
| 11.8 | lipid metabolism.'exotics'(steroids, squalene etc) | pgsc0003dmp400024478|pacid:24428470 | moderately similar to ( 315) AT4G27270 | Symbols: | quinone reductase family protein | chr4:13661458-13663243 REVERSEmoderately similar to ( 298) loc_os08g04460 12008.m04589 protein minor allergen Alt a 7, putative, expressedPRK03767 WrbA no original description | -3.15 | - |
| 11.8 | lipid metabolism.'exotics'(steroids, squalene etc) | pgsc0003dmp400036398|pacid:24399197 | moderately similar to ( 219) AT3G45070 | Symbols: | sulfotransferase family protein | chr3:16486378-16487349 REVERSEmoderately similar to ( 217) loc_os02g45890 12002.m09626 protein flavonol 4-sulfotransferase, putative, expressedSulfotransfer_1 no original description | -8.69 | 3.6 |
| 11.1.10 | lipid metabolism.FA synthesis and FA elongation.beta ketoacyl CoA synthase | pgsc0003dmp400027264|pacid:24408447 | highly similar to ( 644) AT1G07720 | Symbols: KCS3 | KCS3 (3-KETOACYL-COA SYNTHASE 3); acyltransferase/ catalytic/ transferase, transferring acyl groups other than amino-acyl groups | chr1:2390970-2392406 REVERSEhighly similar to ( 516) loc_os10g33370 12010.m06165 protein acyltransferase, putative, expressedFAE1_CUT1_RppA CHS_like BcsA KAS_III FabH no original description | -7.77 | -2.67 |
| 11.1.10 | lipid metabolism.FA synthesis and FA elongation.beta ketoacyl CoA synthase | pgsc0003dmp400045336|pacid:24394042 | highly similar to ( 879) AT2G26640 | Symbols: KCS11 | KCS11 (3-KETOACYL-COA SYNTHASE 11); acyltransferase/ catalytic/ transferase, transferring acyl groups other than amino-acyl groups | chr2:11330094-11331623 FORWARDhighly similar to ( 857) loc_os05g49900 12005.m09060 protein fatty acid elongase, putative, expressedFAE1_CUT1_RppA CHS_like BcsA FabH no original description | - | -4.93 |
| 11.1.8 | lipid metabolism.FA synthesis and FA elongation.acyl coa ligase | pgsc0003dmp400025273|pacid:24405399 | highly similar to ( 789) AT3G48990 | Symbols: | AMP-dependent synthetase and ligase family protein | chr3:18159031-18161294 REVERSEweakly similar to ( 172) 4CL1_TOBAC 4-coumarate--CoA ligase 1 (EC 6.2.1.12) (4CL 1) (4-coumaroyl-CoA synthase 1) - Nicotiana tabacum (Common tobacco)highly similar to ( 622) loc_os04g58710 12004.m35525 protein peroxisomal-coenzyme A synthetase, putative, expressedCaiC PRK05852 AMP-binding PRK07656 PRK03640 Acs PRK08316 PRK06187 PRK07514 PRK08315 PRK05605 PRK07770 PRK07684 PRK07529 PRK08314 PRK12583 PRK09089 PRK06839 PRK06710 caiC PRK06145 PRK06087 PRK08276 PRK04319 PRK07788 PRK07470 PRK09088 FAA1 PRK08162 PRK05677 EntE PRK07059 PRK08974 PRK06178 PRK07786 PRK06188 PRK06155 PRK12492 PRK07787 PRK07798 PRK12406 PRK07771 PRK08751 PRK08279 entE PRK06219 PRK06164 PRK07867 PRK06018 PRK06060 PRK07008 PRK07638 EntF PRK02530 PRK08633 PRK02167 PRK03429 PRK09029 PRK00851 PRK10524 PRK03582 PRK07445 PRK04813 PRK07768 PRK09192 PRK06368 PRK03613 PRK02239 PRK07824 PRK01291 PRK03914 PRK04664 PRK12316 PRK06814 PRK12467 acsA PRK10252 PRK05691 PRK05620 PRK08180 PRK09274 PRK08043 PRK05850 PRK08308 PRK05857 PRK06334 PRK03584 PRK12476 PRK07769 PRK05851 PRK12582 no original description | - | -5.76 |
| 11.2.1 | lipid metabolism.FA desaturation.desaturase | pgsc0003dmp400001266|pacid:24402942 | moderately similar to ( 444) AT3G15850 | Symbols: FAD5, FADB, JB67, ADS3 | FAD5 (FATTY ACID DESATURASE 5); 16:0 monogalactosyldiacylglycerol desaturase/ oxidoreductase | chr3:5359087-5360998 FORWARDOLE1 Delta9-FADS-like FA_desaturase no original description | -5.9 | -1.8 |
| 11.2.3 | lipid metabolism.FA desaturation.omega 3 desaturase | pgsc0003dmp400014857|pacid:24397237 | highly similar to ( 624) AT5G05580 | Symbols: FAD8 | FAD8 (FATTY ACID DESATURASE 8); omega-3 fatty acid desaturase | chr5:1664331-1666345 FORWARDhighly similar to ( 643) FAD3C_RICCO Omega-3 fatty acid desaturase, chloroplast precursor (EC 1.14.19.-) - Ricinus communis (Castor bean)highly similar to ( 557) loc_os03g18070 12003.m07232 protein omega-3 fatty acid desaturase, chloroplast precursor, putative, expressedDelta12-FADS-like FA_desaturase DesA no original description | -3.06 | -1.97 |
| 11.2.3 | lipid metabolism.FA desaturation.omega 3 desaturase | pgsc0003dmp400049918|pacid:24379698 | highly similar to ( 517) AT3G12120 | Symbols: FAD2 | FAD2 (FATTY ACID DESATURASE 2); delta12-fatty acid dehydrogenase/ omega-6 fatty acid desaturase | chr3:3860592-3861743 REVERSEhighly similar to ( 528) FD6E2_SOYBN Omega-6 fatty acid desaturase, endoplasmic reticulum isozyme 2 (EC 1.14.19.-) - Glycine max (Soybean)moderately similar to ( 477) loc_os02g48560 12002.m100054 protein omega-6 fatty acid desaturase, endoplasmic reticulum isozyme 2, putative, expressedDelta12-FADS-like FA_desaturase DesA no original description | -6.96 | - |
| 11.2.4 | lipid metabolism.FA desaturation.omega 6 desaturase | pgsc0003dmp400049918|pacid:24379698 | highly similar to ( 517) AT3G12120 | Symbols: FAD2 | FAD2 (FATTY ACID DESATURASE 2); delta12-fatty acid dehydrogenase/ omega-6 fatty acid desaturase | chr3:3860592-3861743 REVERSEhighly similar to ( 528) FD6E2_SOYBN Omega-6 fatty acid desaturase, endoplasmic reticulum isozyme 2 (EC 1.14.19.-) - Glycine max (Soybean)moderately similar to ( 477) loc_os02g48560 12002.m100054 protein omega-6 fatty acid desaturase, endoplasmic reticulum isozyme 2, putative, expressedDelta12-FADS-like FA_desaturase DesA no original description | -6.96 | - |
| 11.3.1 | lipid metabolism.Phospholipid synthesis.1-acylglycerol-3-phosphate O-acyltransferase | pgsc0003dmp400029514|pacid:24384132 | moderately similar to ( 291) AT1G75020 | Symbols: LPAT4 | LPAT4 (LYSOPHOSPHATIDYL ACYLTRANSFERASE 4); acyltransferase | chr1:28171779-28173357 FORWARDweakly similar to ( 127) LPAT2_BRAOL 1-acyl-sn-glycerol-3-phosphate acyltransferase 2 (EC 2.3.1.51) (Lysophosphatidyl acyltransferase 2) - Brassica oleracea (Wild cabbage)moderately similar to ( 283) loc_os01g57360 12001.m11887 protein 1-acyl-sn-glycerol-3-phosphate acyltransferase 4, putative, expressed no original description | - | -2.19 |
| 11.3.5 | lipid metabolism.Phospholipid synthesis.diacylglycerol kinase | pgsc0003dmp400032027|pacid:24416850 | moderately similar to ( 304) AT2G20900 | Symbols: DGK5, ATDGK5 | diacylglycerol kinase, putative | chr2:8989412-8992798 REVERSEmoderately similar to ( 285) loc_os04g54200 12004.m10312 protein diacylglycerol kinase, putative, expressedDAGKa no original description | - | -1.73 |
| 11.8.1 | lipid metabolism.'exotics' (steroids, squalene etc).sphingolipids | pgsc0003dmp400006053|pacid:24389294 | moderately similar to ( 315) AT1G69640 | Symbols: SBH1 | SBH1 (SPHINGOID BASE HYDROXYLASE 1); catalytic/ sphingosine hydroxylase | chr1:26193933-26195466 REVERSEmoderately similar to ( 289) loc_os06g12250 12006.m05942 protein protein SUR2, putative, expressedERG3 no original description | - | -2.15 |
| 11.8.1 | lipid metabolism.'exotics' (steroids, squalene etc).sphingolipids | pgsc0003dmp400042608|pacid:24404117 | moderately similar to ( 280) AT1G13580 | Symbols: LAG13 | LAG13 (LAG1 LONGEVITY ASSURANCE HOMOLOG 3) | chr1:4645006-4646765 REVERSEmoderately similar to ( 282) ASCL1_ORYSA ASC1-like protein 1 (Alternaria stem canker resistance-like protein 1) - Oryza sativa (Rice)moderately similar to ( 282) loc_os02g37080 12002.m08796 protein ASC1-like protein 1, putative, expressedLAG1 TLC LAG1 no original description | - | -3.78 |
| 11.8.2 | lipid metabolism.'exotics' (steroids, squalene etc).methylsterol monooxygenase | pgsc0003dmp400003857|pacid:24397173 | moderately similar to ( 421) AT2G29390 | Symbols: ATSMO2 | SMO2-2 (STEROL 4-ALPHA-METHYL-OXIDASE 2-2); 4-alpha-methyl-delta7-sterol-4alpha-methyl oxidase/ C-4 methylsterol oxidase | chr2:12610758-12611929 REVERSEmoderately similar to ( 402) loc_os07g01150 12007.m079590 protein C-4 methylsterol oxidase, putative, expressedERG3 no original description | -38.34 | -2.26 |
| 11.8.2 | lipid metabolism.'exotics' (steroids, squalene etc).methylsterol monooxygenase | pgsc0003dmp400022612|pacid:24417753 | moderately similar to ( 377) AT4G12110 | Symbols: SMO1-1, ATSMO1, ATSMO1-1 | SMO1-1 (STEROL-4ALPHA-METHYL OXIDASE 1-1); 4,4-dimethyl-9beta,19-cyclopropylsterol-4alpha-methyl oxidase/ C-4 methylsterol oxidase/ catalytic | chr4:7254197-7256004 FORWARDmoderately similar to ( 329) loc_os10g39810 12010.m065330 protein C-4 methylsterol oxidase, putative, expressedERG3 FA_hydroxylase no original description | -57.32 | -5.94 |
| 11.8.4 | lipid metabolism.'exotics' (steroids, squalene etc).3-beta hydroxysteroid dehydrogenase/isomerase | pgsc0003dmp400012944|pacid:24424642 | highly similar to ( 576) AT2G26260 | Symbols: AT3BETAHSD/D2 | AT3BETAHSD/D2 (3BETA-HYDROXYSTEROID-DEHYDROGENASE/DECARBOXYLASE ISOFORM 2); 3-beta-hydroxy-delta5-steroid dehydrogenase/ sterol-4-alpha-carboxylate 3-dehydrogenase (decarboxylating) | chr2:11178586-11182872 FORWARDhighly similar to ( 535) loc_os03g29170 12003.m35269 protein sterol-4-alpha-carboxylate 3-dehydrogenase, decarboxylating, putative, expressed3Beta_HSD WcaG Epimerase RfbB NAD_binding_4 no original description | -7.96 | - |
| 11.9.2.1 | lipid metabolism.lipid degradation.lipases.triacylglycerol lipase | pgsc0003dmp400014010|pacid:24383078 | moderately similar to ( 469) AT5G42930 | Symbols: | triacylglycerol lipase | chr5:17210738-17214152 REVERSEmoderately similar to ( 434) loc_os02g52830 12002.m10313 protein triacylglycerol lipase, putative, expressedLipase_3 Lipase_3 Lipase no original description | - | 1.94 |
| 11.9.2.1 | lipid metabolism.lipid degradation.lipases.triacylglycerol lipase | pgsc0003dmp400015835|pacid:24418570 | highly similar to ( 551) AT1G02660 | Symbols: | lipase class 3 family protein | chr1:572187-574746 REVERSEmoderately similar to ( 437) loc_os01g15000 12001.m42633 protein triacylglycerol lipase, putative, expressedLipase_3 Lipase_3 Lipase no original description | - | -1.91 |
| 11.9.2.1 | lipid metabolism.lipid degradation.lipases.triacylglycerol lipase | pgsc0003dmp400018071|pacid:24426770 | moderately similar to ( 441) AT4G18550 | Symbols: | lipase class 3 family protein | chr4:10225006-10226862 REVERSEmoderately similar to ( 414) loc_os01g46290 12001.m10827 protein triacylglycerol lipase, putative, expressedLipase_3 Lipase_3 Lipase no original description | -12.38 | - |

**Supplemental table 9:** List of scecondary metabolism DEGs in Waneta and Atlantic after Lso treatment. (mapman analysis)

| **BinCode** | **BinName** | **id** | **description** | **Fold change AT** | **Fold Change Waneta** |
| --- | --- | --- | --- | --- | --- |
| 16.1.1 | secondary metabolism.isoprenoids.non-mevalonate pathway | pgsc0003dmp400033908|pacid:24404067 | highly similar to ( 746) AT1G74470 | Symbols: | geranylgeranyl reductase | chr1:27991248-27992845 FORWARDhighly similar to ( 664) loc_os02g51080 12002.m33869 protein geranylgeranyl hydrogenase, putative, expressedFixC UbiH no original description | -2.94 | - |
| 16.1.1.10 | secondary metabolism.isoprenoids.non-mevalonate pathway.geranylgeranyl pyrophosphate synthase | pgsc0003dmp400027464|pacid:24408573 | moderately similar to ( 422) AT4G36810 | Symbols: GGPS1 | GGPS1 (GERANYLGERANYL PYROPHOSPHATE SYNTHASE 1); farnesyltranstransferase | chr4:17343513-17344628 FORWARDmoderately similar to ( 426) GGPPS_SINAL Geranylgeranyl pyrophosphate synthetase, chloroplast precursor (GGPP synthetase) (GGPS) [Includes: Dimethylallyltranstransferase (EC 2.5.1.1); Geranyltranstransferase (EC 2.5.1.10); Farnesyltranstransferase (EC 2.5.1.29)] - Sinapis albmoderately similar to ( 351) loc_os01g14630 12001.m08066 protein geranylgeranyl pyrophosphate synthetase 1, chloroplast precursor, putative, expressedpolyprenyl_synt Trans_IPPS_HT IspA PRK10581 Trans_IPPS PRK10888 Isoprenoid_Biosyn_C1 no original description | -8.42 | - |
| 16.1.2.1 | secondary metabolism.isoprenoids.mevalonate pathway.acetyl-CoA C-acyltransferase | pgsc0003dmp400019262|pacid:24386753 | highly similar to ( 593) AT5G48230 | Symbols: EMB1276, ACAT2 | ACAT2 (ACETOACETYL-COA THIOLASE 2); acetyl-CoA C-acetyltransferase/ catalytic | chr5:19552570-19555030 REVERSEhighly similar to ( 544) loc_os09g07830 12009.m059940 protein acetyl-CoA acetyltransferase, cytosolic 1, putative, expressedthiolase PRK08235 PRK06954 PRK07800 PRK05790 PRK06845 PRK05656 PRK07025 PRK07491 PRK09051 PRK07124 Thiolase_N PRK08041 PRK06366 PaaJ PRK06205 PRK06633 PRK09050 PRK06689 PRK08234 PRK09052 fadA PRK07661 PRK06445 PRK08131 PRK07801 PRK06504 PRK07851 PRK08242 PRK08170 PRK07108 fadI PRK07850 PRK06025 PRK06690 nondecarbox_cond_enzymes PRK09268 Thiolase_C SCP-x_thiolase no original description | -4.43 | - |
| 16.1.2.3 | secondary metabolism.isoprenoids.mevalonate pathway.HMG-CoA reductase | pgsc0003dmp400006164|pacid:24424892 | highly similar to ( 822) AT1G76490 | Symbols: HMG1, HMGR1 | HMG1 (HYDROXY METHYLGLUTARYL COA REDUCTASE 1); hydroxymethylglutaryl-CoA reductase | chr1:28695801-28698206 FORWARDnearly identical (1076) HMDH2_SOLTU 3-hydroxy-3-methylglutaryl-coenzyme A reductase 2 (EC 1.1.1.34) (HMG-CoA reductase 2) (HMG2.2) - Solanum tuberosum (Potato)highly similar to ( 751) loc_os08g40180 12008.m07998 protein 3-hydroxy-3-methylglutaryl-coenzyme A reductase 3, putative, expressedHMG-CoA_red HMG-CoA_reductase_classI HMG-CoA_reductase HMG1 HMG-CoA_reductase_classII no original description | - | -2.3 |
| 16.1.2.3 | secondary metabolism.isoprenoids.mevalonate pathway.HMG-CoA reductase | pgsc0003dmp400024174|pacid:24425005 | highly similar to ( 672) AT1G76490 | Symbols: HMG1, HMGR1 | HMG1 (HYDROXY METHYLGLUTARYL COA REDUCTASE 1); hydroxymethylglutaryl-CoA reductase | chr1:28695801-28698206 FORWARDhighly similar to ( 763) HMDH1_SOLTU 3-hydroxy-3-methylglutaryl-coenzyme A reductase 1 (EC 1.1.1.34) (HMG-CoA reductase 1) (HMGR1) (HMGR) - Solanum tuberosum (Potato)highly similar to ( 653) loc_os08g40180 12008.m07998 protein 3-hydroxy-3-methylglutaryl-coenzyme A reductase 3, putative, expressedHMG-CoA_red HMG-CoA_reductase_classI HMG-CoA_reductase HMG1 no original description | -14.06 | - |
| 16.1.5 | secondary metabolism.isoprenoids.terpenoids | pgsc0003dmp400020890|pacid:24396351 | moderately similar to ( 343) AT5G23960 | Symbols: ATTPS21, TPS21 | TPS21 (TERPENE SYNTHASE 21); (-)-E-beta-caryophyllene synthase/ alpha-humulene synthase | chr5:8092969-8095128 FORWARDmoderately similar to ( 471) DCS1_GOSHI (+)-delta-cadinene synthase (EC 4.2.3.13) (D-cadinene synthase) - Gossypium hirsutum (Upland cotton)moderately similar to ( 303) loc_os01g23530 12001.m08826 protein sesquiterpene cyclase, putativeTerpene_cyclase_plant_C1 Terpene_synth_C Terpene_cyclase_C1 Terpene_synth Isoprenoid_Biosyn_C1 no original description | -9.5 | - |
| 16.10 | secondary metabolism.simple phenols | pgsc0003dmp400009720|pacid:24383894 | highly similar to ( 808) AT4G39830 | Symbols: | L-ascorbate oxidase, putative | chr4:18479103-18481184 FORWARDhighly similar to ( 586) ASO_CUCMA L-ascorbate oxidase precursor (EC 1.10.3.3) (Ascorbase) (ASO) - Cucurbita maxima (Pumpkin) (Winter squash)highly similar to ( 728) loc_os09g20090 12009.m05216 protein L-ascorbate oxidase precursor, putative, expressedSufI Cu-oxidase_3 Cu-oxidase Cu-oxidase_2 no original description | 8.77 | -9.91 |
| 16.10 | secondary metabolism.simple phenols | pgsc0003dmp400048540|pacid:24383469 | highly similar to ( 711) AT4G39830 | Symbols: | L-ascorbate oxidase, putative | chr4:18479103-18481184 FORWARDhighly similar to ( 508) ASO_TOBAC L-ascorbate oxidase precursor (EC 1.10.3.3) (Ascorbase) (ASO) - Nicotiana tabacum (Common tobacco)highly similar to ( 646) loc_os09g20090 12009.m05216 protein L-ascorbate oxidase precursor, putative, expressedSufI Cu-oxidase Cu-oxidase_2 Cu-oxidase_3 no original description | 8.96 | -8.04 |
| 16.10 | secondary metabolism.simple phenols | pgsc0003dmp400055552|pacid:24409397 | highly similar to ( 764) AT5G21105 | Symbols: | L-ascorbate oxidase/ copper ion binding / oxidoreductase | chr5:7174321-7177409 FORWARDnearly identical (1016) ASO_TOBAC L-ascorbate oxidase precursor (EC 1.10.3.3) (Ascorbase) (ASO) - Nicotiana tabacum (Common tobacco)highly similar to ( 722) loc_os06g37080 12006.m08243 protein L-ascorbate oxidase precursor, putative, expressedSufI Cu-oxidase Cu-oxidase_3 Cu-oxidase_2 no original description | -3.94 | - |
| 16.2 | secondary metabolism.phenylpropanoids | pgsc0003dmp400017915|pacid:24406727 | highly similar to ( 517) AT5G23940 | Symbols: EMB3009 | EMB3009 (embryo defective 3009); transferase/ transferase, transferring acyl groups other than amino-acyl groups | chr5:8076616-8079677 REVERSEweakly similar to ( 117) HCBT1_DIACA Anthranilate N-benzoyltransferase protein 1 (EC 2.3.1.144) (Anthranilate N-hydroxycinnamoyl/benzoyltransferase 1) - Dianthus caryophyllus (Carnation) (Clove pink)moderately similar to ( 397) loc_os08g44840 12008.m08456 protein acyltransferase, putative, expressedTransferase no original description | -5.48 | - |
| 16.2 | secondary metabolism.phenylpropanoids | pgsc0003dmp400021270|pacid:24406171 | weakly similar to ( 151) AT4G35160 | Symbols: | O-methyltransferase family 2 protein | chr4:16730989-16732808 REVERSEmoderately similar to ( 216) 7OMT9_MEDSA Isoflavone-7-O-methyltransferase 9 (EC 2.1.1.150) (Isoflavone-O-methyltransferase 9) (7 IOMT-9) - Medicago sativa (Alfalfa)weakly similar to ( 186) loc_os08g35310 12008.m07520 protein isoflavone-7-O-methytransferase 9, putativeMethyltransf_2 no original description | -4.36 | - |
| 16.2 | secondary metabolism.phenylpropanoids | pgsc0003dmp400028384|pacid:24408645 | moderately similar to ( 341) AT1G65450 | Symbols: | transferase family protein | chr1:24318035-24318895 FORWARDweakly similar to ( 152) HCBT2_DIACA Anthranilate N-benzoyltransferase protein 2 (EC 2.3.1.144) (Anthranilate N-hydroxycinnamoyl/benzoyltransferase 2) - Dianthus caryophyllus (Carnation) (Clove pink)weakly similar to ( 192) loc_os11g31090 12011.m07014 protein transferase, putative, expressedTransferase no original description | -3.91 | - |
| 16.2 | secondary metabolism.phenylpropanoids | pgsc0003dmp400044499|pacid:24397594 | moderately similar to ( 217) AT4G35160 | Symbols: | O-methyltransferase family 2 protein | chr4:16730989-16732808 REVERSEmoderately similar to ( 290) 7OMT6_MEDSA Isoflavone-7-O-methyltransferase 6 (EC 2.1.1.150) (Isoflavone-O-methyltransferase 6) (7-IOMT-6) - Medicago sativa (Alfalfa)moderately similar to ( 272) loc_os08g35310 12008.m07520 protein isoflavone-7-O-methytransferase 9, putativeMethyltransf_2 no original description | -7.35 | - |
| 16.2 | secondary metabolism.phenylpropanoids | pgsc0003dmp400047231|pacid:24389851 | highly similar to ( 627) AT2G39980 | Symbols: | transferase family protein | chr2:16688437-16689885 REVERSEvery weakly similar to (94.0) HCBT1_DIACA Anthranilate N-benzoyltransferase protein 1 (EC 2.3.1.144) (Anthranilate N-hydroxycinnamoyl/benzoyltransferase 1) - Dianthus caryophyllus (Carnation) (Clove pink)moderately similar to ( 439) loc_os01g63480 12001.m12464 protein AER, putative, expressedTransferase no original description | -4.39 | - |
| 16.2.1.1 | secondary metabolism.phenylpropanoids.lignin biosynthesis.PAL | pgsc0003dmp400037349|pacid:24398732 | nearly identical (1195) AT2G37040 | Symbols: pal1, ATPAL1 | pal1 (Phe ammonia lyase 1); phenylalanine ammonia-lyase | chr2:15557602-15560237 REVERSEnearly identical (1345) PAL1_TOBAC Phenylalanine ammonia-lyase (EC 4.3.1.5) - Nicotiana tabacum (Common tobacco)nearly identical (1095) loc_os02g41650 12002.m33781 protein phenylalanine ammonia-lyase, putative, expressedPAL PAL-HAL HutH PRK09367 no original description | - | -5.92 |
| 16.2.1.1 | secondary metabolism.phenylpropanoids.lignin biosynthesis.PAL | pgsc0003dmp400037350|pacid:24399918 | highly similar to ( 671) AT2G37040 | Symbols: pal1, ATPAL1 | pal1 (Phe ammonia lyase 1); phenylalanine ammonia-lyase | chr2:15557602-15560237 REVERSEhighly similar to ( 741) PAL1_SOLTU Phenylalanine ammonia-lyase 1 (EC 4.3.1.5) - Solanum tuberosum (Potato)highly similar to ( 637) loc_os04g43800 12004.m09339 protein phenylalanine ammonia-lyase, putative, expressedPAL PAL-HAL HutH PRK09367 no original description | - | -3.12 |
| 16.2.1.1 | secondary metabolism.phenylpropanoids.lignin biosynthesis.PAL | pgsc0003dmp400037388|pacid:24399493 | nearly identical (1187) AT2G37040 | Symbols: pal1, ATPAL1 | pal1 (Phe ammonia lyase 1); phenylalanine ammonia-lyase | chr2:15557602-15560237 REVERSEnearly identical (1361) PAL1_SOLTU Phenylalanine ammonia-lyase 1 (EC 4.3.1.5) - Solanum tuberosum (Potato)nearly identical (1092) loc_os04g43800 12004.m09339 protein phenylalanine ammonia-lyase, putative, expressedPAL PAL-HAL HutH PRK09367 no original description | - | -8.56 |
| 16.2.1.1 | secondary metabolism.phenylpropanoids.lignin biosynthesis.PAL | pgsc0003dmp400040591|pacid:24386469 | nearly identical (1160) AT3G53260 | Symbols: PAL2, ATPAL2 | PAL2; phenylalanine ammonia-lyase | chr3:19744256-19746619 REVERSEnearly identical (1192) PAL3_TOBAC Phenylalanine ammonia-lyase (EC 4.3.1.5) - Nicotiana tabacum (Common tobacco)nearly identical (1072) loc_os04g43800 12004.m09339 protein phenylalanine ammonia-lyase, putative, expressedPAL PAL-HAL HutH PRK09367 no original description | -5.43 | - |
| 16.2.1.1 | secondary metabolism.phenylpropanoids.lignin biosynthesis.PAL | pgsc0003dmp400054760|pacid:24405735 | highly similar to ( 776) AT2G37040 | Symbols: pal1, ATPAL1 | pal1 (Phe ammonia lyase 1); phenylalanine ammonia-lyase | chr2:15557602-15560237 REVERSEhighly similar to ( 885) PAL1_SOLTU Phenylalanine ammonia-lyase 1 (EC 4.3.1.5) - Solanum tuberosum (Potato)highly similar to ( 732) loc_os04g43800 12004.m09339 protein phenylalanine ammonia-lyase, putative, expressedPAL PAL-HAL HutH PRK09367 no original description | 3.58 | -12.17 |
| 16.2.1.10 | secondary metabolism.phenylpropanoids.lignin biosynthesis.CAD | pgsc0003dmp400028141|pacid:24407640 | moderately similar to ( 468) AT4G39330 | Symbols: ATCAD9, CAD9 | CAD9 (CINNAMYL ALCOHOL DEHYDROGENASE 9); binding / catalytic/ oxidoreductase/ zinc ion binding | chr4:18291268-18292740 FORWARDhighly similar to ( 536) MTDH_FRAAN Probable mannitol dehydrogenase (EC 1.1.1.255) (NAD-dependent mannitol dehydrogenase) - Fragaria ananassa (Strawberry)moderately similar to ( 417) loc_os10g29470 12010.m05819 protein mannitol dehydrogenase, putative, expressedAdhP PRK09422 AdhC Tdh Qor ADH_N ADH_zinc_N tdh PRK10309 no original description | -4.04 | - |
| 16.2.1.10 | secondary metabolism.phenylpropanoids.lignin biosynthesis.CAD | pgsc0003dmp400032136|pacid:24404875 | highly similar to ( 541) AT4G39330 | Symbols: ATCAD9, CAD9 | CAD9 (CINNAMYL ALCOHOL DEHYDROGENASE 9); binding / catalytic/ oxidoreductase/ zinc ion binding | chr4:18291268-18292740 FORWARDhighly similar to ( 511) MTDH_MEDSA Probable mannitol dehydrogenase (EC 1.1.1.255) (NAD-dependent mannitol dehydrogenase) - Medicago sativa (Alfalfa)moderately similar to ( 424) loc_os10g29470 12010.m05819 protein mannitol dehydrogenase, putative, expressedAdhP PRK09422 AdhC Tdh Qor ADH_N tdh ADH_zinc_N PRK10083 no original description | - | -2.03 |
| 16.2.1.10 | secondary metabolism.phenylpropanoids.lignin biosynthesis.CAD | pgsc0003dmp400044639|pacid:24419275 | moderately similar to ( 489) AT3G19450 | Symbols: CAD4, ATCAD4, CAD, CAD-C | ATCAD4; cinnamyl-alcohol dehydrogenase | chr3:6744859-6747005 FORWARDhighly similar to ( 575) CADH4_TOBAC Cinnamyl alcohol dehydrogenase (EC 1.1.1.195) (CAD) - Nicotiana tabacum (Common tobacco)moderately similar to ( 487) loc_os02g09490 12002.m06246 protein cinnamyl alcohol dehydrogenase, putative, expressedAdhP PRK09422 AdhC Tdh Qor ADH_N tdh ADH_zinc_N no original description | -3.64 | - |
| 16.2.1.3 | secondary metabolism.phenylpropanoids.lignin biosynthesis.4CL | pgsc0003dmp400025029|pacid:24403960 | highly similar to ( 790) AT3G21240 | Symbols: 4CL2, AT4CL2 | 4CL2 (4-COUMARATE:COA LIGASE 2); 4-coumarate-CoA ligase | chr3:7454497-7457314 REVERSEnearly identical (1072) 4CL2_SOLTU 4-coumarate--CoA ligase 2 (EC 6.2.1.12) (4CL 2) (4-coumaroyl-CoA synthase 2) - Solanum tuberosum (Potato)highly similar to ( 752) loc_os02g08100 12002.m06156 protein 4-coumarate--CoA ligase 1, putative, expressedCaiC AMP-binding PRK08316 PRK08315 PRK05605 PRK07656 Acs PRK06187 PRK07770 PRK08314 PRK06710 PRK03640 PRK06178 PRK07684 PRK07059 PRK09089 PRK12583 PRK07786 PRK08974 PRK05677 PRK08276 PRK06087 PRK07470 PRK12492 PRK07529 PRK06188 PRK06839 PRK08751 PRK06145 FAA1 PRK08162 caiC PRK07514 PRK07788 EntE PRK07771 PRK07798 PRK06219 PRK09088 PRK04319 PRK12406 PRK06155 PRK08633 PRK06164 PRK07638 entE PRK07787 PRK06018 PRK05852 PRK06368 PRK06060 PRK07008 PRK09192 PRK08279 PRK07867 PRK00851 PRK10524 PRK05691 EntF PRK06814 PRK06334 PRK09029 PRK07768 PRK03429 PRK02530 PRK03582 PRK09274 PRK12476 PRK07445 PRK05850 PRK12316 PRK04813 PRK05620 PRK03613 PRK03914 PRK07769 PRK05857 PRK01291 PRK02239 PRK08308 PRK03584 PRK02167 PRK04664 PRK07824 PRK08180 PRK08043 PRK12467 acsA PRK10252 no original description | - | -4.38 |
| 16.2.1.6 | secondary metabolism.phenylpropanoids.lignin biosynthesis.CCoAOMT | pgsc0003dmp400011444|pacid:24401997 | moderately similar to ( 263) AT1G67980 | Symbols: CCOAMT | CCoAMT; caffeoyl-CoA O-methyltransferase | chr1:25488239-25488934 FORWARDmoderately similar to ( 249) CAMT_MEDSA Caffeoyl-CoA O-methyltransferase (EC 2.1.1.104) (Trans-caffeoyl-CoA 3-O-methyltransferase) (CCoAMT) (CCoAOMT) - Medicago sativa (Alfalfa)moderately similar to ( 248) loc_os06g06980 12006.m31989 protein caffeoyl-CoA O-methyltransferase 1, putative, expressedMethyltransf_3 COG4122 no original description | -3.94 | - |
| 16.4.1 | secondary metabolism.N misc.alkaloid-like | pgsc0003dmp400005368|pacid:24406961 | highly similar to ( 615) AT1G08470 | Symbols: | strictosidine synthase family protein | chr1:2682262-2683977 REVERSEweakly similar to ( 151) STSY_CATRO Strictosidine synthase precursor (EC 4.3.3.2) - Catharanthus roseus (Rosy periwinkle) (Madagascar periwinkle)highly similar to ( 503) loc_os03g53950 12003.m10360 protein strictosidine synthase precursor, putative, expressedStr_synth no original description | -5.48 | - |
| 16.4.1 | secondary metabolism.N misc.alkaloid-like | pgsc0003dmp400042004|pacid:24379139 | highly similar to ( 593) AT2G20340 | Symbols: | tyrosine decarboxylase, putative | chr2:8779804-8782490 FORWARDhighly similar to ( 674) TYDC4_PETCR Tyrosine decarboxylase 4 (EC 4.1.1.25) - Petroselinum crispum (Parsley) (Petroselinum hortense)highly similar to ( 585) loc_os07g25590 12007.m06837 protein tyrosine decarboxylase 1, putative, expressedPyridoxal_deC GadB no original description | -24.72 | - |
| 16.7 | secondary metabolism.wax | pgsc0003dmp400041236|pacid:24378293 | highly similar to ( 682) AT1G02205 | Symbols: CER1 | CER1 (ECERIFERUM 1); octadecanal decarbonylase | chr1:418818-422154 FORWARDhighly similar to ( 696) loc_os10g33250 12010.m21915 protein CER1, putative, expressed no original description | - | 3.67 |
| 16.8.5.1 | secondary metabolism.flavonoids.isoflavones.isoflavone reductase | pgsc0003dmp400006583|pacid:24384261 | moderately similar to ( 415) AT4G39230 | Symbols: | isoflavone reductase, putative | chr4:18266024-18267604 REVERSEmoderately similar to ( 421) IFRH_SOLTU Isoflavone reductase homolog (EC 1.3.1.-) (CP100) - Solanum tuberosum (Potato)moderately similar to ( 375) loc_os06g27770 12006.m07322 protein isoflavone reductase, putative, expressedNmrA no original description | -8.58 | - |

**Supplemental table 10:** Gprofiler analysis of genes down regulated in AT (table A) and in Waneta table (B)

**Supplemental table 10A**

| p-value | term ID | t type | t group | t name | Q&T list |
| --- | --- | --- | --- | --- | --- |
| 0.05 | GO:0019202 | MF | 18 | amino acid kinase activity | PGSC0003DMT400083218,PGSC0003DMT400068829 |
| 0.0285 | GO:0016757 | MF | 15 | transferase activity, transferring glycosyl groups | PGSC0003DMT400072824,PGSC0003DMT400073085,PGSC0003DMT400015281,PGSC0003DMT400043474,PGSC0003DMT400021018,PGSC0003DMT400026177,PGSC0003DMT400055138,PGSC0003DMT400001149,PGSC0003DMT400030650,PGSC0003DMT400030670,PGSC0003DMT400030678,PGSC0003DMT400003004,PGSC0003DMT400061841,PGSC0003DMT400063689,PGSC0003DMT400035264,PGSC0003DMT400067358,PGSC0003DMT400069972 |
| 0.00773 | GO:0016762 | MF | 13 | xyloglucan:xyloglucosyl transferase activity | PGSC0003DMT400055138,PGSC0003DMT400061841,PGSC0003DMT400063689,PGSC0003DMT400067358 |
| 0.000000253 | GO:0046906 | MF | 5 | tetrapyrrole binding | PGSC0003DMT400092927,PGSC0003DMT400079219,PGSC0003DMT400015740,PGSC0003DMT400083158,PGSC0003DMT400044144,PGSC0003DMT400020100,PGSC0003DMT400021389,PGSC0003DMT400021390,PGSC0003DMT400021391,PGSC0003DMT400021422,PGSC0003DMT400022072,PGSC0003DMT400025979,PGSC0003DMT400030674,PGSC0003DMT400060106,PGSC0003DMT400031804,PGSC0003DMT400062367,PGSC0003DMT400032791,PGSC0003DMT400064554,PGSC0003DMT400064553,PGSC0003DMT400034893,PGSC0003DMT400035007,PGSC0003DMT400065518,PGSC0003DMT400066399,PGSC0003DMT400010985,PGSC0003DMT400068373,PGSC0003DMT400069485,PGSC0003DMT400070317,PGSC0003DMT400014232,PGSC0003DMT400043054 |
| 7.97E-15 | GO:0016168 | MF | 5 | chlorophyll binding | PGSC0003DMT400092927,PGSC0003DMT400015740,PGSC0003DMT400083158,PGSC0003DMT400020100,PGSC0003DMT400021389,PGSC0003DMT400021390,PGSC0003DMT400021391,PGSC0003DMT400021422,PGSC0003DMT400022072,PGSC0003DMT400032791,PGSC0003DMT400034893,PGSC0003DMT400035007,PGSC0003DMT400010985,PGSC0003DMT400043054 |
| 0.0183 | GO:0045548 | MF | 8 | phenylalanine ammonia-lyase activity | PGSC0003DMT400080548,PGSC0003DMT400060308 |
| 6.39E-12 | GO:0003824 | MF | 7 | catalytic activity | PGSC0003DMT400092702,PGSC0003DMT400088761,PGSC0003DMT400072824,PGSC0003DMT400073020,PGSC0003DMT400073085,PGSC0003DMT400073263,PGSC0003DMT400073632,PGSC0003DMT400073645,PGSC0003DMT400073749,PGSC0003DMT400073854,PGSC0003DMT400074510,PGSC0003DMT400075158,PGSC0003DMT400076054,PGSC0003DMT400076209,PGSC0003DMT400076251,PGSC0003DMT400076776,PGSC0003DMT400077579,PGSC0003DMT400077762,PGSC0003DMT400079219,PGSC0003DMT400014415,PGSC0003DMT400080548,PGSC0003DMT400015087,PGSC0003DMT400015093,PGSC0003DMT400081311,PGSC0003DMT400015184,PGSC0003DMT400015233,PGSC0003DMT400015281,PGSC0003DMT400082388,PGSC0003DMT400016326,PGSC0003DMT400016512,PGSC0003DMT400016545,PGSC0003DMT400083215,PGSC0003DMT400083218,PGSC0003DMT400017205,PGSC0003DMT400017206,PGSC0003DMT400043474,PGSC0003DMT400044144,PGSC0003DMT400018519,PGSC0003DMT400018704,PGSC0003DMT400018853,PGSC0003DMT400019555,PGSC0003DMT400045152,PGSC0003DMT400045665,PGSC0003DMT400046204,PGSC0003DMT400021018,PGSC0003DMT400021824,PGSC0003DMT400023082,PGSC0003DMT400049809,PGSC0003DMT400050229,PGSC0003DMT400050234,PGSC0003DMT400024317,PGSC0003DMT400024601,PGSC0003DMT400051730,PGSC0003DMT400025691,PGSC0003DMT400025792,PGSC0003DMT400025805,PGSC0003DMT400025974,PGSC0003DMT400025979,PGSC0003DMT400052335,PGSC0003DMT400026177,PGSC0003DMT400026230,PGSC0003DMT400026489,PGSC0003DMT400052998,PGSC0003DMT400026984,PGSC0003DMT400027149,PGSC0003DMT400029318,PGSC0003DMT400029422,PGSC0003DMT400027955,PGSC0003DMT400054066,PGSC0003DMT400028288,PGSC0003DMT400055138,PGSC0003DMT400055490,PGSC0003DMT400056145,PGSC0003DMT400000651,PGSC0003DMT400001149,PGSC0003DMT400030650,PGSC0003DMT400030670,PGSC0003DMT400030674,PGSC0003DMT400030676,PGSC0003DMT400030678,PGSC0003DMT400060106,PGSC0003DMT400001638,PGSC0003DMT400001709,PGSC0003DMT400030762,PGSC0003DMT400001833,PGSC0003DMT400060308,PGSC0003DMT400001912,PGSC0003DMT400060733,PGSC0003DMT400031370,PGSC0003DMT400003004,PGSC0003DMT400003243,PGSC0003DMT400003336,PGSC0003DMT400061841,PGSC0003DMT400062026,PGSC0003DMT400004181,PGSC0003DMT400031804,PGSC0003DMT400062367,PGSC0003DMT400062382,PGSC0003DMT400062385,PGSC0003DMT400032028,PGSC0003DMT400005519,PGSC0003DMT400063689,PGSC0003DMT400006459,PGSC0003DMT400033232,PGSC0003DMT400033236,PGSC0003DMT400006944,PGSC0003DMT400007043,PGSC0003DMT400064554,PGSC0003DMT400064553,PGSC0003DMT400007732,PGSC0003DMT400034324,PGSC0003DMT400008103,PGSC0003DMT400008105,PGSC0003DMT400065512,PGSC0003DMT400035264,PGSC0003DMT400065518,PGSC0003DMT400035274,PGSC0003DMT400008936,PGSC0003DMT400009055,PGSC0003DMT400009096,PGSC0003DMT400035460,PGSC0003DMT400035542,PGSC0003DMT400065841,PGSC0003DMT400009330,PGSC0003DMT400065996,PGSC0003DMT400009667,PGSC0003DMT400066217,PGSC0003DMT400036034,PGSC0003DMT400066399,PGSC0003DMT400036366,PGSC0003DMT400036789,PGSC0003DMT400010618,PGSC0003DMT400036974,PGSC0003DMT400067358,PGSC0003DMT400067600,PGSC0003DMT400011102,PGSC0003DMT400067881,PGSC0003DMT400037473,PGSC0003DMT400037696,PGSC0003DMT400068373,PGSC0003DMT400068829,PGSC0003DMT400011970,PGSC0003DMT400012216,PGSC0003DMT400038500,PGSC0003DMT400069897,PGSC0003DMT400069972,PGSC0003DMT400012716,PGSC0003DMT400012752,PGSC0003DMT400012809,PGSC0003DMT400070176,PGSC0003DMT400070317,PGSC0003DMT400070621,PGSC0003DMT400039131,PGSC0003DMT400039287,PGSC0003DMT400071195,PGSC0003DMT400039333,PGSC0003DMT400039494,PGSC0003DMT400013830,PGSC0003DMT400071700,PGSC0003DMT400071770,PGSC0003DMT400039734,PGSC0003DMT400014232,PGSC0003DMT400040198,PGSC0003DMT400040485,PGSC0003DMT400040847,PGSC0003DMT400041566,PGSC0003DMT400041850,PGSC0003DMT400041876 |
| 0.0000712 | GO:0016491 | MF | 7 | oxidoreductase activity | PGSC0003DMT400092702,PGSC0003DMT400073749,PGSC0003DMT400074510,PGSC0003DMT400076054,PGSC0003DMT400079219,PGSC0003DMT400015184,PGSC0003DMT400082388,PGSC0003DMT400016326,PGSC0003DMT400083218,PGSC0003DMT400044144,PGSC0003DMT400018519,PGSC0003DMT400018853,PGSC0003DMT400045665,PGSC0003DMT400021824,PGSC0003DMT400050229,PGSC0003DMT400050234,PGSC0003DMT400024317,PGSC0003DMT400025805,PGSC0003DMT400025974,PGSC0003DMT400025979,PGSC0003DMT400026984,PGSC0003DMT400030674,PGSC0003DMT400030676,PGSC0003DMT400060106,PGSC0003DMT400001709,PGSC0003DMT400031804,PGSC0003DMT400062367,PGSC0003DMT400005519,PGSC0003DMT400033236,PGSC0003DMT400007043,PGSC0003DMT400064554,PGSC0003DMT400064553,PGSC0003DMT400065518,PGSC0003DMT400008936,PGSC0003DMT400035542,PGSC0003DMT400066217,PGSC0003DMT400036034,PGSC0003DMT400066399,PGSC0003DMT400067881,PGSC0003DMT400068373,PGSC0003DMT400068829,PGSC0003DMT400011970,PGSC0003DMT400012216,PGSC0003DMT400070317,PGSC0003DMT400039734,PGSC0003DMT400014232,PGSC0003DMT400040847,PGSC0003DMT400041566 |
| 0.0155 | GO:0016627 | MF | 7 | oxidoreductase activity, acting on the CH-CH group of donors | PGSC0003DMT400015184,PGSC0003DMT400050234,PGSC0003DMT400026984,PGSC0003DMT400007043,PGSC0003DMT400039734 |
| 0.00208 | GO:0016628 | MF | 7 | oxidoreductase activity, acting on the CH-CH group of donors, NAD or NADP as acceptor | PGSC0003DMT400015184,PGSC0003DMT400050234,PGSC0003DMT400007043,PGSC0003DMT400039734 |
| 0.00706 | GO:0016829 | MF | 7 | lyase activity | PGSC0003DMT400076251,PGSC0003DMT400080548,PGSC0003DMT400017206,PGSC0003DMT400027955,PGSC0003DMT400030762,PGSC0003DMT400060308,PGSC0003DMT400004181,PGSC0003DMT400062382,PGSC0003DMT400007732,PGSC0003DMT400008103,PGSC0003DMT400065512,PGSC0003DMT400009330,PGSC0003DMT400067600 |
| 0.00213 | GO:0016787 | MF | 7 | hydrolase activity | PGSC0003DMT400088761,PGSC0003DMT400073020,PGSC0003DMT400073632,PGSC0003DMT400073854,PGSC0003DMT400076209,PGSC0003DMT400076776,PGSC0003DMT400077579,PGSC0003DMT400014415,PGSC0003DMT400015093,PGSC0003DMT400015233,PGSC0003DMT400016545,PGSC0003DMT400083215,PGSC0003DMT400018704,PGSC0003DMT400019555,PGSC0003DMT400045152,PGSC0003DMT400046204,PGSC0003DMT400023082,PGSC0003DMT400049809,PGSC0003DMT400024601,PGSC0003DMT400051730,PGSC0003DMT400025691,PGSC0003DMT400052335,PGSC0003DMT400026489,PGSC0003DMT400027149,PGSC0003DMT400055138,PGSC0003DMT400056145,PGSC0003DMT400000651,PGSC0003DMT400001638,PGSC0003DMT400001833,PGSC0003DMT400003336,PGSC0003DMT400061841,PGSC0003DMT400062026,PGSC0003DMT400032028,PGSC0003DMT400063689,PGSC0003DMT400006459,PGSC0003DMT400008105,PGSC0003DMT400035274,PGSC0003DMT400009055,PGSC0003DMT400009096,PGSC0003DMT400035460,PGSC0003DMT400009667,PGSC0003DMT400036366,PGSC0003DMT400036974,PGSC0003DMT400067358,PGSC0003DMT400037696,PGSC0003DMT400038500,PGSC0003DMT400012716,PGSC0003DMT400012809,PGSC0003DMT400070621,PGSC0003DMT400039131,PGSC0003DMT400071195,PGSC0003DMT400039333,PGSC0003DMT400013830,PGSC0003DMT400071700,PGSC0003DMT400041850 |
| 0.0267 | GO:0008233 | MF | 7 | peptidase activity | PGSC0003DMT400088761,PGSC0003DMT400077579,PGSC0003DMT400015093,PGSC0003DMT400083215,PGSC0003DMT400019555,PGSC0003DMT400024601,PGSC0003DMT400025691,PGSC0003DMT400027149,PGSC0003DMT400000651,PGSC0003DMT400036366,PGSC0003DMT400036974,PGSC0003DMT400038500,PGSC0003DMT400039131,PGSC0003DMT400071195,PGSC0003DMT400013830,PGSC0003DMT400071700 |
| 0.000000899 | GO:0016798 | MF | 7 | hydrolase activity, acting on glycosyl bonds | PGSC0003DMT400014415,PGSC0003DMT400015233,PGSC0003DMT400023082,PGSC0003DMT400055138,PGSC0003DMT400001638,PGSC0003DMT400001833,PGSC0003DMT400061841,PGSC0003DMT400032028,PGSC0003DMT400063689,PGSC0003DMT400008105,PGSC0003DMT400009055,PGSC0003DMT400009096,PGSC0003DMT400035460,PGSC0003DMT400009667,PGSC0003DMT400067358,PGSC0003DMT400037696,PGSC0003DMT400012716,PGSC0003DMT400012809,PGSC0003DMT400039333,PGSC0003DMT400041850 |
| 0.00000178 | GO:0004553 | MF | 7 | hydrolase activity, hydrolyzing O-glycosyl compounds | PGSC0003DMT400014415,PGSC0003DMT400015233,PGSC0003DMT400023082,PGSC0003DMT400055138,PGSC0003DMT400001638,PGSC0003DMT400001833,PGSC0003DMT400061841,PGSC0003DMT400032028,PGSC0003DMT400063689,PGSC0003DMT400008105,PGSC0003DMT400009055,PGSC0003DMT400009096,PGSC0003DMT400009667,PGSC0003DMT400067358,PGSC0003DMT400037696,PGSC0003DMT400012716,PGSC0003DMT400012809,PGSC0003DMT400039333,PGSC0003DMT400041850 |
| 0.0000259 | GO:0008810 | MF | 7 | cellulase activity | PGSC0003DMT400023082,PGSC0003DMT400032028,PGSC0003DMT400008105,PGSC0003DMT400009667,PGSC0003DMT400012809 |
| 0.0132 | GO:0005507 | MF | 10 | copper ion binding | PGSC0003DMT400074510,PGSC0003DMT400082388,PGSC0003DMT400045665,PGSC0003DMT400025805,PGSC0003DMT400025974,PGSC0003DMT400061040,PGSC0003DMT400011028,PGSC0003DMT400012216 |
| 0.000062 | GO:0008171 | MF | 11 | O-methyltransferase activity | PGSC0003DMT400075158,PGSC0003DMT400016512,PGSC0003DMT400029318,PGSC0003DMT400031370,PGSC0003DMT400003243,PGSC0003DMT400065996,PGSC0003DMT400011102 |
| 0.0111 | GO:0047746 | MF | 21 | chlorophyllase activity | PGSC0003DMT400049809,PGSC0003DMT400056145 |

**Supplemental table 10B**

| p-value | term ID | t name |  |
| --- | --- | --- | --- |
| 0.00085 | GO:0043565 | sequence-specific DNA binding | PGSC0003DMG400028520,PGSC0003DMG400029207,PGSC0003DMG400029371,PGSC0003DMG400031140,PGSC0003DMG400005835,PGSC0003DMG402007388,PGSC0003DMG400017334,PGSC0003DMG400007947,PGSC0003DMG400019408,PGSC0003DMG400009530,PGSC0003DMG400019824,PGSC0003DMG400019884,PGSC0003DMG400020608,PGSC0003DMG400010401,PGSC0003DMG400021895,PGSC0003DMG400022063,PGSC0003DMG400022172,PGSC0003DMG400000211,PGSC0003DMG400011633,PGSC0003DMG400012160,PGSC0003DMG400001659,PGSC0003DMG400012318,PGSC0003DMG400024961,PGSC0003DMG400003126,PGSC0003DMG400027167,PGSC0003DMG400015015,PGSC0003DMG400016769 |
| 4.11E-05 | GO:0050366 | tyramine N-feruloyltransferase activity | PGSC0003DMG400014770,PGSC0003DMG400014771,PGSC0003DMG400014774,PGSC0003DMG400014776,PGSC0003DMG400014778 |
| 1.46E-08 | GO:0001071 | nucleic acid binding transcription factor activity | PGSC0003DMG400036566,PGSC0003DMG400040046,PGSC0003DMG400028459,PGSC0003DMG400028520,PGSC0003DMG400029207,PGSC0003DMG400029371,PGSC0003DMG400031140,PGSC0003DMG400005835,PGSC0003DMG400005837,PGSC0003DMG400034322,PGSC0003DMG402007388,PGSC0003DMG400017334,PGSC0003DMG400007458,PGSC0003DMG400007947,PGSC0003DMG400008538,PGSC0003DMG400019408,PGSC0003DMG400009530,PGSC0003DMG400019824,PGSC0003DMG400019884,PGSC0003DMG400020608,PGSC0003DMG400010401,PGSC0003DMG400010724,PGSC0003DMG400010750,PGSC0003DMG400010753,PGSC0003DMG400010870,PGSC0003DMG400021895,PGSC0003DMG400022063,PGSC0003DMG400022823,PGSC0003DMG400000211,PGSC0003DMG400011633,PGSC0003DMG400000711,PGSC0003DMG400000811,PGSC0003DMG400012154,PGSC0003DMG400012160,PGSC0003DMG400001659,PGSC0003DMG400012318,PGSC0003DMG400002507,PGSC0003DMG400024853,PGSC0003DMG400024961,PGSC0003DMG400002899,PGSC0003DMG400003126,PGSC0003DMG400013744,PGSC0003DMG400026035,PGSC0003DMG400026232,PGSC0003DMG400026261,PGSC0003DMG400014417,PGSC0003DMG400026461,PGSC0003DMG400004501,PGSC0003DMG400026821,PGSC0003DMG400004898,PGSC0003DMG400027167,PGSC0003DMG400027176,PGSC0003DMG400015015,PGSC0003DMG400015424,PGSC0003DMG400015668,PGSC0003DMG400016003,PGSC0003DMG400016004,PGSC0003DMG400016006,PGSC0003DMG400016769 |
| 1.46E-08 | GO:0003700 | transcription factor activity, sequence-specific DNA binding | PGSC0003DMG400036566,PGSC0003DMG400040046,PGSC0003DMG400028459,PGSC0003DMG400028520,PGSC0003DMG400029207,PGSC0003DMG400029371,PGSC0003DMG400031140,PGSC0003DMG400005835,PGSC0003DMG400005837,PGSC0003DMG400034322,PGSC0003DMG402007388,PGSC0003DMG400017334,PGSC0003DMG400007458,PGSC0003DMG400007947,PGSC0003DMG400008538,PGSC0003DMG400019408,PGSC0003DMG400009530,PGSC0003DMG400019824,PGSC0003DMG400019884,PGSC0003DMG400020608,PGSC0003DMG400010401,PGSC0003DMG400010724,PGSC0003DMG400010750,PGSC0003DMG400010753,PGSC0003DMG400010870,PGSC0003DMG400021895,PGSC0003DMG400022063,PGSC0003DMG400022823,PGSC0003DMG400000211,PGSC0003DMG400011633,PGSC0003DMG400000711,PGSC0003DMG400000811,PGSC0003DMG400012154,PGSC0003DMG400012160,PGSC0003DMG400001659,PGSC0003DMG400012318,PGSC0003DMG400002507,PGSC0003DMG400024853,PGSC0003DMG400024961,PGSC0003DMG400002899,PGSC0003DMG400003126,PGSC0003DMG400013744,PGSC0003DMG400026035,PGSC0003DMG400026232,PGSC0003DMG400026261,PGSC0003DMG400014417,PGSC0003DMG400026461,PGSC0003DMG400004501,PGSC0003DMG400026821,PGSC0003DMG400004898,PGSC0003DMG400027167,PGSC0003DMG400027176,PGSC0003DMG400015015,PGSC0003DMG400015424,PGSC0003DMG400015668,PGSC0003DMG400016003,PGSC0003DMG400016004,PGSC0003DMG400016006,PGSC0003DMG400016769 |
| 0.0117 | GO:0016407 | acetyltransferase activity | PGSC0003DMG400038970,PGSC0003DMG400032272,PGSC0003DMG400032273,PGSC0003DMG400014770,PGSC0003DMG400014771,PGSC0003DMG400014774,PGSC0003DMG400014776,PGSC0003DMG400014778 |
| 0.00734 | GO:0016410 | N-acyltransferase activity | PGSC0003DMG400038970,PGSC0003DMG400032272,PGSC0003DMG400032273,PGSC0003DMG400014770,PGSC0003DMG400014771,PGSC0003DMG400014774,PGSC0003DMG400014776,PGSC0003DMG400014778 |
| 0.00438 | GO:0008080 | N-acetyltransferase activity | PGSC0003DMG400038970,PGSC0003DMG400032272,PGSC0003DMG400032273,PGSC0003DMG400014770,PGSC0003DMG400014771,PGSC0003DMG400014774,PGSC0003DMG400014776,PGSC0003DMG400014778 |
| 0.025 | GO:0016840 | carbon-nitrogen lyase activity | PGSC0003DMG400031365,PGSC0003DMG400031457,PGSC0003DMG401021549,PGSC0003DMG402021549,PGSC0003DMG402021564 |
| 0.000195 | GO:0016841 | ammonia-lyase activity | PGSC0003DMG400031365,PGSC0003DMG400031457,PGSC0003DMG401021549,PGSC0003DMG402021549,PGSC0003DMG402021564 |
| 0.00199 | GO:0045548 | phenylalanine ammonia-lyase activity | PGSC0003DMG400031365,PGSC0003DMG401021549,PGSC0003DMG402021564 |
| 0.00529 | GO:0005488 | binding | PGSC0003DMG400042498,PGSC0003DMG400043507,PGSC0003DMG400043830,PGSC0003DMG400044745,PGSC0003DMG400045432,PGSC0003DMG400036566,PGSC0003DMG400037159,PGSC0003DMG400046726,PGSC0003DMG400047336,PGSC0003DMG400047346,PGSC0003DMG400038332,PGSC0003DMG400040046,PGSC0003DMG400040729,PGSC0003DMG400041071,PGSC0003DMG400041311,PGSC0003DMG400041458,PGSC0003DMG400041467,PGSC0003DMG400028185,PGSC0003DMG400028287,PGSC0003DMG400028339,PGSC0003DMG400028390,PGSC0003DMG400028462,PGSC0003DMG400028520,PGSC0003DMG402028569,PGSC0003DMG400028575,PGSC0003DMG400028579,PGSC0003DMG400028797,PGSC0003DMG400028846,PGSC0003DMG400029046,PGSC0003DMG400029050,PGSC0003DMG400029105,PGSC0003DMG400029207,PGSC0003DMG401029345,PGSC0003DMG400029371,PGSC0003DMG400029415,PGSC0003DMG400029519,PGSC0003DMG400029520,PGSC0003DMG400029544,PGSC0003DMG400029645,PGSC0003DMG400029885,PGSC0003DMG400029894,PGSC0003DMG400029920,PGSC0003DMG400030035,PGSC0003DMG400030058,PGSC0003DMG400030113,PGSC0003DMG400030119,PGSC0003DMG400030144,PGSC0003DMG400030209,PGSC0003DMG400030220,PGSC0003DMG401030312,PGSC0003DMG400030364,PGSC0003DMG400030405,PGSC0003DMG400030462,PGSC0003DMG400030608,PGSC0003DMG400030661,PGSC0003DMG400030680,PGSC0003DMG400030892,PGSC0003DMG401030920,PGSC0003DMG400031140,PGSC0003DMG400031156,PGSC0003DMG400031235,PGSC0003DMG400005649,PGSC0003DMG401005729,PGSC0003DMG400031471,PGSC0003DMG400005745,PGSC0003DMG400005805,PGSC0003DMG400031547,PGSC0003DMG400031548,PGSC0003DMG400005822,PGSC0003DMG400005825,PGSC0003DMG400005835,PGSC0003DMG400005837,PGSC0003DMG400005899,PGSC0003DMG400005909,PGSC0003DMG400005969,PGSC0003DMG400031860,PGSC0003DMG400031861,PGSC0003DMG400006000,PGSC0003DMG400006022,PGSC0003DMG400006041,PGSC0003DMG400032118,PGSC0003DMG400006217,PGSC0003DMG400006255,PGSC0003DMG400006268,PGSC0003DMG400032498,PGSC0003DMG400006309,PGSC0003DMG400032555,PGSC0003DMG400033084,PGSC0003DMG400033126,PGSC0003DMG400006640,PGSC0003DMG400006652,PGSC0003DMG401006653,PGSC0003DMG402006653,PGSC0003DMG400033334,PGSC0003DMG400033363,PGSC0003DMG400006729,PGSC0003DMG400006764,PGSC0003DMG400033632,PGSC0003DMG400006814,PGSC0003DMG400033651,PGSC0003DMG400033667,PGSC0003DMG400033685,PGSC0003DMG400033904,PGSC0003DMG400034322,PGSC0003DMG400016973,PGSC0003DMG402016981,PGSC0003DMG400007122,PGSC0003DMG400017062,PGSC0003DMG400017065,PGSC0003DMG400007152,PGSC0003DMG400017106,PGSC0003DMG400017223,PGSC0003DMG400017278,PGSC0003DMG400007375,PGSC0003DMG400007376,PGSC0003DMG402007388,PGSC0003DMG400017330,PGSC0003DMG400017334,PGSC0003DMG400017358,PGSC0003DMG400007458,PGSC0003DMG400007603,PGSC0003DMG400007613,PGSC0003DMG400007614,PGSC0003DMG400007634,PGSC0003DMG400007787,PGSC0003DMG400007816,PGSC0003DMG400007915,PGSC0003DMG400007947,PGSC0003DMG400007994,PGSC0003DMG400017946,PGSC0003DMG400017969,PGSC0003DMG400017976,PGSC0003DMG400018045,PGSC0003DMG400008149,PGSC0003DMG400008163,PGSC0003DMG400008202,PGSC0003DMG400008205,PGSC0003DMG400018115,PGSC0003DMG400008298,PGSC0003DMG400008300,PGSC0003DMG400008301,PGSC0003DMG400008337,PGSC0003DMG400018182,PGSC0003DMG400018184,PGSC0003DMG402018257,PGSC0003DMG400008438,PGSC0003DMG400008538,PGSC0003DMG400008564,PGSC0003DMG400018428,PGSC0003DMG400018435,PGSC0003DMG400018446,PGSC0003DMG400018457,PGSC0003DMG402018475,PGSC0003DMG400008639,PGSC0003DMG400018623,PGSC0003DMG400018632,PGSC0003DMG400018677,PGSC0003DMG400018752,PGSC0003DMG400008936,PGSC0003DMG400018942,PGSC0003DMG400009007,PGSC0003DMG400009021,PGSC0003DMG400009078,PGSC0003DMG400009114,PGSC0003DMG400009115,PGSC0003DMG400009116,PGSC0003DMG400009181,PGSC0003DMG400009238,PGSC0003DMG400019408,PGSC0003DMG400019526,PGSC0003DMG400019618,PGSC0003DMG400009530,PGSC0003DMG400019721,PGSC0003DMG400019725,PGSC0003DMG400019726,PGSC0003DMG400019804,PGSC0003DMG400019824,PGSC0003DMG400019884,PGSC0003DMG400009686,PGSC0003DMG400009699,PGSC0003DMG402009727,PGSC0003DMG400009734,PGSC0003DMG400019975,PGSC0003DMG400009902,PGSC0003DMG400020139,PGSC0003DMG400009919,PGSC0003DMG400020261,PGSC0003DMG400010032,PGSC0003DMG400011180,PGSC0003DMG400020505,PGSC0003DMG400020541,PGSC0003DMG400020564,PGSC0003DMG400011222,PGSC0003DMG400020608,PGSC0003DMG400010401,PGSC0003DMG400010410,PGSC0003DMG400010439,PGSC0003DMG400010504,PGSC0003DMG400010631,PGSC0003DMG400010724,PGSC0003DMG400011326,PGSC0003DMG400010750,PGSC0003DMG400010753,PGSC0003DMG400020909,PGSC0003DMG400010859,PGSC0003DMG400010870,PGSC0003DMG401021043,PGSC0003DMG400021131,PGSC0003DMG400011012,PGSC0003DMG400011052,PGSC0003DMG400011071,PGSC0003DMG400021213,PGSC0003DMG400021325,PGSC0003DMG400021331,PGSC0003DMG400021409,PGSC0003DMG400021421,PGSC0003DMG400021508,PGSC0003DMG401021514,PGSC0003DMG400021533,PGSC0003DMG400021683,PGSC0003DMG400021684,PGSC0003DMG400021777,PGSC0003DMG400021778,PGSC0003DMG400022562,PGSC0003DMG400021895,PGSC0003DMG400021902,PGSC0003DMG400021948,PGSC0003DMG400021993,PGSC0003DMG400022727,PGSC0003DMG400011548,PGSC0003DMG400022063,PGSC0003DMG400022075,PGSC0003DMG400022775,PGSC0003DMG400000114,PGSC0003DMG400022172,PGSC0003DMG400022823,PGSC0003DMG400022836,PGSC0003DMG400000211,PGSC0003DMG400022893,PGSC0003DMG400022894,PGSC0003DMG400000311,PGSC0003DMG400011633,PGSC0003DMG400022340,PGSC0003DMG400023028,PGSC0003DMG400022383,PGSC0003DMG400000493,PGSC0003DMG400000495,PGSC0003DMG400000512,PGSC0003DMG400011750,PGSC0003DMG400011751,PGSC0003DMG400023344,PGSC0003DMG400000555,PGSC0003DMG400000711,PGSC0003DMG400000730,PGSC0003DMG400000791,PGSC0003DMG400011878,PGSC0003DMG400000811,PGSC0003DMG400000957,PGSC0003DMG400001081,PGSC0003DMG400023814,PGSC0003DMG400001333,PGSC0003DMG400012110,PGSC0003DMG400024055,PGSC0003DMG400001448,PGSC0003DMG400024148,PGSC0003DMG400024149,PGSC0003DMG400012154,PGSC0003DMG400012160,PGSC0003DMG400001659,PGSC0003DMG400012201,PGSC0003DMG400024260,PGSC0003DMG400001708,PGSC0003DMG400024281,PGSC0003DMG400001756,PGSC0003DMG400012224,PGSC0003DMG400001853,PGSC0003DMG400024438,PGSC0003DMG400012318,PGSC0003DMG400001923,PGSC0003DMG400001932,PGSC0003DMG400024491,PGSC0003DMG400002038,PGSC0003DMG400012421,PGSC0003DMG400024539,PGSC0003DMG400002134,PGSC0003DMG400002153,PGSC0003DMG400002156,PGSC0003DMG400002166,PGSC0003DMG400002167,PGSC0003DMG400002169,PGSC0003DMG400024661,PGSC0003DMG400024703,PGSC0003DMG400012589,PGSC0003DMG400002327,PGSC0003DMG400012613,PGSC0003DMG400002426,PGSC0003DMG400024771,PGSC0003DMG400002507,PGSC0003DMG400012763,PGSC0003DMG400024944,PGSC0003DMG400024961,PGSC0003DMG400025022,PGSC0003DMG400025023,PGSC0003DMG400002826,PGSC0003DMG400013022,PGSC0003DMG400002899,PGSC0003DMG400002908,PGSC0003DMG400002910,PGSC0003DMG400025126,PGSC0003DMG400013180,PGSC0003DMG400025214,PGSC0003DMG400003105,PGSC0003DMG400003126,PGSC0003DMG400003208,PGSC0003DMG400013405,PGSC0003DMG400003306,PGSC0003DMG400003383,PGSC0003DMG400013488,PGSC0003DMG400013524,PGSC0003DMG400003429,PGSC0003DMG400013545,PGSC0003DMG400003461,PGSC0003DMG400003527,PGSC0003DMG401013639,PGSC0003DMG400003566,PGSC0003DMG400025613,PGSC0003DMG400013696,PGSC0003DMG400003626,PGSC0003DMG400013744,PGSC0003DMG400013797,PGSC0003DMG400013802,PGSC0003DMG400013803,PGSC0003DMG400013804,PGSC0003DMG400025738,PGSC0003DMG400025755,PGSC0003DMG400025756,PGSC0003DMG400013886,PGSC0003DMG400013898,PGSC0003DMG400013928,PGSC0003DMG400003924,PGSC0003DMG400014051,PGSC0003DMG400003948,PGSC0003DMG402025869,PGSC0003DMG400003964,PGSC0003DMG400003982,PGSC0003DMG402003983,PGSC0003DMG400003984,PGSC0003DMG400025884,PGSC0003DMG400003999,PGSC0003DMG400004037,PGSC0003DMG400025931,PGSC0003DMG400014168,PGSC0003DMG400026023,PGSC0003DMG400026035,PGSC0003DMG400014213,PGSC0003DMG400026155,PGSC0003DMG400026159,PGSC0003DMG400026230,PGSC0003DMG400026232,PGSC0003DMG400004284,PGSC0003DMG400026261,PGSC0003DMG400004301,PGSC0003DMG400026332,PGSC0003DMG400026346,PGSC0003DMG400026384,PGSC0003DMG400026401,PGSC0003DMG400014417,PGSC0003DMG400004409,PGSC0003DMG404026432,PGSC0003DMG400026433,PGSC0003DMG400026436,PGSC0003DMG400026461,PGSC0003DMG400004458,PGSC0003DMG400026593,PGSC0003DMG400014566,PGSC0003DMG400014589,PGSC0003DMG400026821,PGSC0003DMG400014678,PGSC0003DMG400014779,PGSC0003DMG402027023,PGSC0003DMG400027046,PGSC0003DMG400004860,PGSC0003DMG400027124,PGSC0003DMG400004898,PGSC0003DMG400027167,PGSC0003DMG400027174,PGSC0003DMG400027176,PGSC0003DMG400004930,PGSC0003DMG400027241,PGSC0003DMG400015015,PGSC0003DMG400027468,PGSC0003DMG400027469,PGSC0003DMG400005213,PGSC0003DMG400027692,PGSC0003DMG400027771,PGSC0003DMG400027781,PGSC0003DMG400005390,PGSC0003DMG400005456,PGSC0003DMG400027914,PGSC0003DMG400005515,PGSC0003DMG400005538,PGSC0003DMG400015448,PGSC0003DMG400015492,PGSC0003DMG400015533,PGSC0003DMG400015668,PGSC0003DMG400015767,PGSC0003DMG400015835,PGSC0003DMG400016003,PGSC0003DMG400016004,PGSC0003DMG400016006,PGSC0003DMG400016011,PGSC0003DMG400016217,PGSC0003DMG400016326,PGSC0003DMG400016695,PGSC0003DMG400016769 |
| 0.05 | GO:0036094 | small molecule binding | PGSC0003DMG400043507,PGSC0003DMG400043830,PGSC0003DMG400044745,PGSC0003DMG400037159,PGSC0003DMG400047346,PGSC0003DMG400040729,PGSC0003DMG400041071,PGSC0003DMG400041311,PGSC0003DMG400041458,PGSC0003DMG400028287,PGSC0003DMG400028339,PGSC0003DMG402028569,PGSC0003DMG400029415,PGSC0003DMG400029520,PGSC0003DMG400029885,PGSC0003DMG400030035,PGSC0003DMG400030058,PGSC0003DMG400030113,PGSC0003DMG400030119,PGSC0003DMG400030144,PGSC0003DMG400030209,PGSC0003DMG400030220,PGSC0003DMG400030405,PGSC0003DMG400030462,PGSC0003DMG400031235,PGSC0003DMG401005729,PGSC0003DMG400031860,PGSC0003DMG400031861,PGSC0003DMG400006000,PGSC0003DMG400006022,PGSC0003DMG400032498,PGSC0003DMG400033126,PGSC0003DMG400033334,PGSC0003DMG400006729,PGSC0003DMG400006764,PGSC0003DMG400033651,PGSC0003DMG400033667,PGSC0003DMG402016981,PGSC0003DMG400017062,PGSC0003DMG400017106,PGSC0003DMG400017278,PGSC0003DMG400017330,PGSC0003DMG400017358,PGSC0003DMG400007634,PGSC0003DMG400017946,PGSC0003DMG400017976,PGSC0003DMG400008149,PGSC0003DMG400018184,PGSC0003DMG402018257,PGSC0003DMG400018428,PGSC0003DMG400018457,PGSC0003DMG400018677,PGSC0003DMG400018752,PGSC0003DMG400018942,PGSC0003DMG400009078,PGSC0003DMG400009114,PGSC0003DMG400009181,PGSC0003DMG400009238,PGSC0003DMG400019721,PGSC0003DMG400019725,PGSC0003DMG400019726,PGSC0003DMG400019804,PGSC0003DMG400009686,PGSC0003DMG400010032,PGSC0003DMG400020564,PGSC0003DMG400010504,PGSC0003DMG400020909,PGSC0003DMG400021131,PGSC0003DMG400011071,PGSC0003DMG400021421,PGSC0003DMG401021514,PGSC0003DMG400022562,PGSC0003DMG400021948,PGSC0003DMG400021993,PGSC0003DMG400000311,PGSC0003DMG400022383,PGSC0003DMG400011878,PGSC0003DMG400000957,PGSC0003DMG400001081,PGSC0003DMG400023814,PGSC0003DMG400024055,PGSC0003DMG400001448,PGSC0003DMG400024148,PGSC0003DMG400024149,PGSC0003DMG400024260,PGSC0003DMG400001853,PGSC0003DMG400001932,PGSC0003DMG400002038,PGSC0003DMG400024661,PGSC0003DMG400002327,PGSC0003DMG400002426,PGSC0003DMG400013022,PGSC0003DMG400025126,PGSC0003DMG400003105,PGSC0003DMG400013524,PGSC0003DMG400003429,PGSC0003DMG400013545,PGSC0003DMG400003461,PGSC0003DMG400003527,PGSC0003DMG401013639,PGSC0003DMG400025613,PGSC0003DMG400013797,PGSC0003DMG400013802,PGSC0003DMG400013803,PGSC0003DMG400013804,PGSC0003DMG400013886,PGSC0003DMG400013928,PGSC0003DMG400003982,PGSC0003DMG402003983,PGSC0003DMG400003999,PGSC0003DMG400004037,PGSC0003DMG400025931,PGSC0003DMG400014213,PGSC0003DMG400026155,PGSC0003DMG400026384,PGSC0003DMG400004409,PGSC0003DMG400026433,PGSC0003DMG400026436,PGSC0003DMG400014678,PGSC0003DMG400004860,PGSC0003DMG400027124,PGSC0003DMG400004930,PGSC0003DMG400027468,PGSC0003DMG400027469,PGSC0003DMG400027771,PGSC0003DMG400015448,PGSC0003DMG400015492,PGSC0003DMG400015835 |
| 0.00156 | GO:0097367 | carbohydrate derivative binding | PGSC0003DMG400043507,PGSC0003DMG400043830,PGSC0003DMG400044745,PGSC0003DMG400037159,PGSC0003DMG400047346,PGSC0003DMG400040729,PGSC0003DMG400041071,PGSC0003DMG400041311,PGSC0003DMG400041458,PGSC0003DMG400028287,PGSC0003DMG400028339,PGSC0003DMG402028569,PGSC0003DMG400029415,PGSC0003DMG400029520,PGSC0003DMG400029885,PGSC0003DMG400030035,PGSC0003DMG400030058,PGSC0003DMG400030113,PGSC0003DMG400030119,PGSC0003DMG400030144,PGSC0003DMG400030209,PGSC0003DMG400030220,PGSC0003DMG400030405,PGSC0003DMG400030462,PGSC0003DMG400031235,PGSC0003DMG401005729,PGSC0003DMG400031860,PGSC0003DMG400031861,PGSC0003DMG400006000,PGSC0003DMG400006022,PGSC0003DMG400032498,PGSC0003DMG400033334,PGSC0003DMG400006729,PGSC0003DMG400033651,PGSC0003DMG400033667,PGSC0003DMG402016981,PGSC0003DMG400017062,PGSC0003DMG400017106,PGSC0003DMG400017278,PGSC0003DMG400017330,PGSC0003DMG400017358,PGSC0003DMG400007634,PGSC0003DMG400017946,PGSC0003DMG400008149,PGSC0003DMG400018184,PGSC0003DMG402018257,PGSC0003DMG400018428,PGSC0003DMG400018457,PGSC0003DMG400018677,PGSC0003DMG400018752,PGSC0003DMG400018942,PGSC0003DMG400009078,PGSC0003DMG400009114,PGSC0003DMG400009181,PGSC0003DMG400009238,PGSC0003DMG400019721,PGSC0003DMG400019725,PGSC0003DMG400019726,PGSC0003DMG400019804,PGSC0003DMG400009686,PGSC0003DMG400010032,PGSC0003DMG400020564,PGSC0003DMG400010504,PGSC0003DMG400020909,PGSC0003DMG400021131,PGSC0003DMG400011071,PGSC0003DMG400021421,PGSC0003DMG401021514,PGSC0003DMG400022562,PGSC0003DMG400021948,PGSC0003DMG400021993,PGSC0003DMG400000311,PGSC0003DMG400011878,PGSC0003DMG400000957,PGSC0003DMG400001081,PGSC0003DMG400023814,PGSC0003DMG400024055,PGSC0003DMG400024148,PGSC0003DMG400024149,PGSC0003DMG400024260,PGSC0003DMG400001853,PGSC0003DMG400002038,PGSC0003DMG400024661,PGSC0003DMG400002327,PGSC0003DMG400002426,PGSC0003DMG400013022,PGSC0003DMG400025126,PGSC0003DMG400013524,PGSC0003DMG400003429,PGSC0003DMG400013545,PGSC0003DMG400003527,PGSC0003DMG400025613,PGSC0003DMG400013797,PGSC0003DMG400013802,PGSC0003DMG400013803,PGSC0003DMG400013804,PGSC0003DMG400013886,PGSC0003DMG400013928,PGSC0003DMG400003982,PGSC0003DMG402003983,PGSC0003DMG400003999,PGSC0003DMG400004037,PGSC0003DMG400025931,PGSC0003DMG400014213,PGSC0003DMG400026155,PGSC0003DMG400026384,PGSC0003DMG400004409,PGSC0003DMG400026433,PGSC0003DMG400026436,PGSC0003DMG400014678,PGSC0003DMG400004860,PGSC0003DMG400027124,PGSC0003DMG400004930,PGSC0003DMG400027468,PGSC0003DMG400027469,PGSC0003DMG400027771,PGSC0003DMG400015448,PGSC0003DMG400015492,PGSC0003DMG400015835 |
| 0.000565 | GO:0001882 | nucleoside binding | PGSC0003DMG400043507,PGSC0003DMG400043830,PGSC0003DMG400044745,PGSC0003DMG400037159,PGSC0003DMG400047346,PGSC0003DMG400040729,PGSC0003DMG400041071,PGSC0003DMG400041311,PGSC0003DMG400041458,PGSC0003DMG400028287,PGSC0003DMG400028339,PGSC0003DMG402028569,PGSC0003DMG400029415,PGSC0003DMG400029520,PGSC0003DMG400029885,PGSC0003DMG400030035,PGSC0003DMG400030058,PGSC0003DMG400030113,PGSC0003DMG400030119,PGSC0003DMG400030144,PGSC0003DMG400030209,PGSC0003DMG400030220,PGSC0003DMG400030405,PGSC0003DMG400030462,PGSC0003DMG400031235,PGSC0003DMG401005729,PGSC0003DMG400031860,PGSC0003DMG400031861,PGSC0003DMG400006000,PGSC0003DMG400006022,PGSC0003DMG400032498,PGSC0003DMG400033334,PGSC0003DMG400006729,PGSC0003DMG400033651,PGSC0003DMG400033667,PGSC0003DMG402016981,PGSC0003DMG400017062,PGSC0003DMG400017106,PGSC0003DMG400017278,PGSC0003DMG400017330,PGSC0003DMG400017358,PGSC0003DMG400007634,PGSC0003DMG400017946,PGSC0003DMG400008149,PGSC0003DMG400018184,PGSC0003DMG402018257,PGSC0003DMG400018428,PGSC0003DMG400018457,PGSC0003DMG400018677,PGSC0003DMG400018752,PGSC0003DMG400018942,PGSC0003DMG400009078,PGSC0003DMG400009114,PGSC0003DMG400009181,PGSC0003DMG400009238,PGSC0003DMG400019721,PGSC0003DMG400019725,PGSC0003DMG400019726,PGSC0003DMG400019804,PGSC0003DMG400009686,PGSC0003DMG400010032,PGSC0003DMG400020564,PGSC0003DMG400010504,PGSC0003DMG400020909,PGSC0003DMG400021131,PGSC0003DMG400011071,PGSC0003DMG400021421,PGSC0003DMG401021514,PGSC0003DMG400022562,PGSC0003DMG400021948,PGSC0003DMG400021993,PGSC0003DMG400000311,PGSC0003DMG400011878,PGSC0003DMG400000957,PGSC0003DMG400001081,PGSC0003DMG400023814,PGSC0003DMG400024055,PGSC0003DMG400024148,PGSC0003DMG400024149,PGSC0003DMG400024260,PGSC0003DMG400001853,PGSC0003DMG400002038,PGSC0003DMG400024661,PGSC0003DMG400002327,PGSC0003DMG400002426,PGSC0003DMG400013022,PGSC0003DMG400025126,PGSC0003DMG400013524,PGSC0003DMG400003429,PGSC0003DMG400013545,PGSC0003DMG400003527,PGSC0003DMG400025613,PGSC0003DMG400013797,PGSC0003DMG400013802,PGSC0003DMG400013803,PGSC0003DMG400013804,PGSC0003DMG400013886,PGSC0003DMG400013928,PGSC0003DMG400003982,PGSC0003DMG402003983,PGSC0003DMG400003999,PGSC0003DMG400004037,PGSC0003DMG400025931,PGSC0003DMG400014213,PGSC0003DMG400026155,PGSC0003DMG400026384,PGSC0003DMG400004409,PGSC0003DMG400026433,PGSC0003DMG400026436,PGSC0003DMG400014678,PGSC0003DMG400004860,PGSC0003DMG400027124,PGSC0003DMG400004930,PGSC0003DMG400027468,PGSC0003DMG400027469,PGSC0003DMG400027771,PGSC0003DMG400015448,PGSC0003DMG400015492,PGSC0003DMG400015835 |
| 0.000486 | GO:0001883 | purine nucleoside binding | PGSC0003DMG400043507,PGSC0003DMG400043830,PGSC0003DMG400044745,PGSC0003DMG400037159,PGSC0003DMG400047346,PGSC0003DMG400040729,PGSC0003DMG400041071,PGSC0003DMG400041311,PGSC0003DMG400041458,PGSC0003DMG400028287,PGSC0003DMG400028339,PGSC0003DMG402028569,PGSC0003DMG400029415,PGSC0003DMG400029520,PGSC0003DMG400029885,PGSC0003DMG400030035,PGSC0003DMG400030058,PGSC0003DMG400030113,PGSC0003DMG400030119,PGSC0003DMG400030144,PGSC0003DMG400030209,PGSC0003DMG400030220,PGSC0003DMG400030405,PGSC0003DMG400030462,PGSC0003DMG400031235,PGSC0003DMG401005729,PGSC0003DMG400031860,PGSC0003DMG400031861,PGSC0003DMG400006000,PGSC0003DMG400006022,PGSC0003DMG400032498,PGSC0003DMG400033334,PGSC0003DMG400006729,PGSC0003DMG400033651,PGSC0003DMG400033667,PGSC0003DMG402016981,PGSC0003DMG400017062,PGSC0003DMG400017106,PGSC0003DMG400017278,PGSC0003DMG400017330,PGSC0003DMG400017358,PGSC0003DMG400007634,PGSC0003DMG400017946,PGSC0003DMG400008149,PGSC0003DMG400018184,PGSC0003DMG402018257,PGSC0003DMG400018428,PGSC0003DMG400018457,PGSC0003DMG400018677,PGSC0003DMG400018752,PGSC0003DMG400018942,PGSC0003DMG400009078,PGSC0003DMG400009114,PGSC0003DMG400009181,PGSC0003DMG400009238,PGSC0003DMG400019721,PGSC0003DMG400019725,PGSC0003DMG400019726,PGSC0003DMG400019804,PGSC0003DMG400009686,PGSC0003DMG400010032,PGSC0003DMG400020564,PGSC0003DMG400010504,PGSC0003DMG400020909,PGSC0003DMG400021131,PGSC0003DMG400011071,PGSC0003DMG400021421,PGSC0003DMG401021514,PGSC0003DMG400022562,PGSC0003DMG400021948,PGSC0003DMG400021993,PGSC0003DMG400000311,PGSC0003DMG400011878,PGSC0003DMG400000957,PGSC0003DMG400001081,PGSC0003DMG400023814,PGSC0003DMG400024055,PGSC0003DMG400024148,PGSC0003DMG400024149,PGSC0003DMG400024260,PGSC0003DMG400001853,PGSC0003DMG400002038,PGSC0003DMG400024661,PGSC0003DMG400002327,PGSC0003DMG400002426,PGSC0003DMG400013022,PGSC0003DMG400025126,PGSC0003DMG400013524,PGSC0003DMG400003429,PGSC0003DMG400013545,PGSC0003DMG400003527,PGSC0003DMG400025613,PGSC0003DMG400013797,PGSC0003DMG400013802,PGSC0003DMG400013803,PGSC0003DMG400013804,PGSC0003DMG400013886,PGSC0003DMG400013928,PGSC0003DMG400003982,PGSC0003DMG402003983,PGSC0003DMG400003999,PGSC0003DMG400004037,PGSC0003DMG400025931,PGSC0003DMG400014213,PGSC0003DMG400026155,PGSC0003DMG400026384,PGSC0003DMG400004409,PGSC0003DMG400026433,PGSC0003DMG400026436,PGSC0003DMG400014678,PGSC0003DMG400004860,PGSC0003DMG400027124,PGSC0003DMG400004930,PGSC0003DMG400027468,PGSC0003DMG400027469,PGSC0003DMG400027771,PGSC0003DMG400015448,PGSC0003DMG400015492,PGSC0003DMG400015835 |
| 0.000565 | GO:0032549 | ribonucleoside binding | PGSC0003DMG400043507,PGSC0003DMG400043830,PGSC0003DMG400044745,PGSC0003DMG400037159,PGSC0003DMG400047346,PGSC0003DMG400040729,PGSC0003DMG400041071,PGSC0003DMG400041311,PGSC0003DMG400041458,PGSC0003DMG400028287,PGSC0003DMG400028339,PGSC0003DMG402028569,PGSC0003DMG400029415,PGSC0003DMG400029520,PGSC0003DMG400029885,PGSC0003DMG400030035,PGSC0003DMG400030058,PGSC0003DMG400030113,PGSC0003DMG400030119,PGSC0003DMG400030144,PGSC0003DMG400030209,PGSC0003DMG400030220,PGSC0003DMG400030405,PGSC0003DMG400030462,PGSC0003DMG400031235,PGSC0003DMG401005729,PGSC0003DMG400031860,PGSC0003DMG400031861,PGSC0003DMG400006000,PGSC0003DMG400006022,PGSC0003DMG400032498,PGSC0003DMG400033334,PGSC0003DMG400006729,PGSC0003DMG400033651,PGSC0003DMG400033667,PGSC0003DMG402016981,PGSC0003DMG400017062,PGSC0003DMG400017106,PGSC0003DMG400017278,PGSC0003DMG400017330,PGSC0003DMG400017358,PGSC0003DMG400007634,PGSC0003DMG400017946,PGSC0003DMG400008149,PGSC0003DMG400018184,PGSC0003DMG402018257,PGSC0003DMG400018428,PGSC0003DMG400018457,PGSC0003DMG400018677,PGSC0003DMG400018752,PGSC0003DMG400018942,PGSC0003DMG400009078,PGSC0003DMG400009114,PGSC0003DMG400009181,PGSC0003DMG400009238,PGSC0003DMG400019721,PGSC0003DMG400019725,PGSC0003DMG400019726,PGSC0003DMG400019804,PGSC0003DMG400009686,PGSC0003DMG400010032,PGSC0003DMG400020564,PGSC0003DMG400010504,PGSC0003DMG400020909,PGSC0003DMG400021131,PGSC0003DMG400011071,PGSC0003DMG400021421,PGSC0003DMG401021514,PGSC0003DMG400022562,PGSC0003DMG400021948,PGSC0003DMG400021993,PGSC0003DMG400000311,PGSC0003DMG400011878,PGSC0003DMG400000957,PGSC0003DMG400001081,PGSC0003DMG400023814,PGSC0003DMG400024055,PGSC0003DMG400024148,PGSC0003DMG400024149,PGSC0003DMG400024260,PGSC0003DMG400001853,PGSC0003DMG400002038,PGSC0003DMG400024661,PGSC0003DMG400002327,PGSC0003DMG400002426,PGSC0003DMG400013022,PGSC0003DMG400025126,PGSC0003DMG400013524,PGSC0003DMG400003429,PGSC0003DMG400013545,PGSC0003DMG400003527,PGSC0003DMG400025613,PGSC0003DMG400013797,PGSC0003DMG400013802,PGSC0003DMG400013803,PGSC0003DMG400013804,PGSC0003DMG400013886,PGSC0003DMG400013928,PGSC0003DMG400003982,PGSC0003DMG402003983,PGSC0003DMG400003999,PGSC0003DMG400004037,PGSC0003DMG400025931,PGSC0003DMG400014213,PGSC0003DMG400026155,PGSC0003DMG400026384,PGSC0003DMG400004409,PGSC0003DMG400026433,PGSC0003DMG400026436,PGSC0003DMG400014678,PGSC0003DMG400004860,PGSC0003DMG400027124,PGSC0003DMG400004930,PGSC0003DMG400027468,PGSC0003DMG400027469,PGSC0003DMG400027771,PGSC0003DMG400015448,PGSC0003DMG400015492,PGSC0003DMG400015835 |
| 0.000486 | GO:0032550 | purine ribonucleoside binding | PGSC0003DMG400043507,PGSC0003DMG400043830,PGSC0003DMG400044745,PGSC0003DMG400037159,PGSC0003DMG400047346,PGSC0003DMG400040729,PGSC0003DMG400041071,PGSC0003DMG400041311,PGSC0003DMG400041458,PGSC0003DMG400028287,PGSC0003DMG400028339,PGSC0003DMG402028569,PGSC0003DMG400029415,PGSC0003DMG400029520,PGSC0003DMG400029885,PGSC0003DMG400030035,PGSC0003DMG400030058,PGSC0003DMG400030113,PGSC0003DMG400030119,PGSC0003DMG400030144,PGSC0003DMG400030209,PGSC0003DMG400030220,PGSC0003DMG400030405,PGSC0003DMG400030462,PGSC0003DMG400031235,PGSC0003DMG401005729,PGSC0003DMG400031860,PGSC0003DMG400031861,PGSC0003DMG400006000,PGSC0003DMG400006022,PGSC0003DMG400032498,PGSC0003DMG400033334,PGSC0003DMG400006729,PGSC0003DMG400033651,PGSC0003DMG400033667,PGSC0003DMG402016981,PGSC0003DMG400017062,PGSC0003DMG400017106,PGSC0003DMG400017278,PGSC0003DMG400017330,PGSC0003DMG400017358,PGSC0003DMG400007634,PGSC0003DMG400017946,PGSC0003DMG400008149,PGSC0003DMG400018184,PGSC0003DMG402018257,PGSC0003DMG400018428,PGSC0003DMG400018457,PGSC0003DMG400018677,PGSC0003DMG400018752,PGSC0003DMG400018942,PGSC0003DMG400009078,PGSC0003DMG400009114,PGSC0003DMG400009181,PGSC0003DMG400009238,PGSC0003DMG400019721,PGSC0003DMG400019725,PGSC0003DMG400019726,PGSC0003DMG400019804,PGSC0003DMG400009686,PGSC0003DMG400010032,PGSC0003DMG400020564,PGSC0003DMG400010504,PGSC0003DMG400020909,PGSC0003DMG400021131,PGSC0003DMG400011071,PGSC0003DMG400021421,PGSC0003DMG401021514,PGSC0003DMG400022562,PGSC0003DMG400021948,PGSC0003DMG400021993,PGSC0003DMG400000311,PGSC0003DMG400011878,PGSC0003DMG400000957,PGSC0003DMG400001081,PGSC0003DMG400023814,PGSC0003DMG400024055,PGSC0003DMG400024148,PGSC0003DMG400024149,PGSC0003DMG400024260,PGSC0003DMG400001853,PGSC0003DMG400002038,PGSC0003DMG400024661,PGSC0003DMG400002327,PGSC0003DMG400002426,PGSC0003DMG400013022,PGSC0003DMG400025126,PGSC0003DMG400013524,PGSC0003DMG400003429,PGSC0003DMG400013545,PGSC0003DMG400003527,PGSC0003DMG400025613,PGSC0003DMG400013797,PGSC0003DMG400013802,PGSC0003DMG400013803,PGSC0003DMG400013804,PGSC0003DMG400013886,PGSC0003DMG400013928,PGSC0003DMG400003982,PGSC0003DMG402003983,PGSC0003DMG400003999,PGSC0003DMG400004037,PGSC0003DMG400025931,PGSC0003DMG400014213,PGSC0003DMG400026155,PGSC0003DMG400026384,PGSC0003DMG400004409,PGSC0003DMG400026433,PGSC0003DMG400026436,PGSC0003DMG400014678,PGSC0003DMG400004860,PGSC0003DMG400027124,PGSC0003DMG400004930,PGSC0003DMG400027468,PGSC0003DMG400027469,PGSC0003DMG400027771,PGSC0003DMG400015448,PGSC0003DMG400015492,PGSC0003DMG400015835 |
| 0.000228 | GO:0001871 | pattern binding | PGSC0003DMG401005729,PGSC0003DMG400006729,PGSC0003DMG400017106,PGSC0003DMG400009115,PGSC0003DMG400009116,PGSC0003DMG400019725,PGSC0003DMG400019726,PGSC0003DMG400002038,PGSC0003DMG400026384 |
| 0.00117 | GO:0030246 | carbohydrate binding | PGSC0003DMG400030144,PGSC0003DMG401005729,PGSC0003DMG400006729,PGSC0003DMG400017106,PGSC0003DMG400018184,PGSC0003DMG400008639,PGSC0003DMG400018677,PGSC0003DMG400009115,PGSC0003DMG400009116,PGSC0003DMG400019725,PGSC0003DMG400019726,PGSC0003DMG400011071,PGSC0003DMG400002038,PGSC0003DMG400026384 |
| 0.000228 | GO:0030247 | polysaccharide binding | PGSC0003DMG401005729,PGSC0003DMG400006729,PGSC0003DMG400017106,PGSC0003DMG400009115,PGSC0003DMG400009116,PGSC0003DMG400019725,PGSC0003DMG400019726,PGSC0003DMG400002038,PGSC0003DMG400026384 |
| 0.0349 | GO:1901265 | nucleoside phosphate binding | PGSC0003DMG400043507,PGSC0003DMG400043830,PGSC0003DMG400044745,PGSC0003DMG400037159,PGSC0003DMG400047346,PGSC0003DMG400040729,PGSC0003DMG400041071,PGSC0003DMG400041311,PGSC0003DMG400041458,PGSC0003DMG400028287,PGSC0003DMG400028339,PGSC0003DMG402028569,PGSC0003DMG400029415,PGSC0003DMG400029520,PGSC0003DMG400029885,PGSC0003DMG400030035,PGSC0003DMG400030058,PGSC0003DMG400030113,PGSC0003DMG400030119,PGSC0003DMG400030144,PGSC0003DMG400030209,PGSC0003DMG400030220,PGSC0003DMG400030405,PGSC0003DMG400030462,PGSC0003DMG400031235,PGSC0003DMG401005729,PGSC0003DMG400031860,PGSC0003DMG400031861,PGSC0003DMG400006000,PGSC0003DMG400006022,PGSC0003DMG400032498,PGSC0003DMG400033126,PGSC0003DMG400033334,PGSC0003DMG400006729,PGSC0003DMG400006764,PGSC0003DMG400033651,PGSC0003DMG400033667,PGSC0003DMG402016981,PGSC0003DMG400017062,PGSC0003DMG400017106,PGSC0003DMG400017278,PGSC0003DMG400017330,PGSC0003DMG400017358,PGSC0003DMG400007634,PGSC0003DMG400017946,PGSC0003DMG400017976,PGSC0003DMG400008149,PGSC0003DMG400018184,PGSC0003DMG402018257,PGSC0003DMG400018428,PGSC0003DMG400018457,PGSC0003DMG400018677,PGSC0003DMG400018752,PGSC0003DMG400018942,PGSC0003DMG400009078,PGSC0003DMG400009114,PGSC0003DMG400009181,PGSC0003DMG400009238,PGSC0003DMG400019721,PGSC0003DMG400019725,PGSC0003DMG400019726,PGSC0003DMG400019804,PGSC0003DMG400009686,PGSC0003DMG400010032,PGSC0003DMG400020564,PGSC0003DMG400010504,PGSC0003DMG400020909,PGSC0003DMG400021131,PGSC0003DMG400011071,PGSC0003DMG400021421,PGSC0003DMG401021514,PGSC0003DMG400022562,PGSC0003DMG400021948,PGSC0003DMG400021993,PGSC0003DMG400000311,PGSC0003DMG400022383,PGSC0003DMG400011878,PGSC0003DMG400000957,PGSC0003DMG400001081,PGSC0003DMG400023814,PGSC0003DMG400024055,PGSC0003DMG400001448,PGSC0003DMG400024148,PGSC0003DMG400024149,PGSC0003DMG400024260,PGSC0003DMG400001853,PGSC0003DMG400001932,PGSC0003DMG400002038,PGSC0003DMG400024661,PGSC0003DMG400002327,PGSC0003DMG400002426,PGSC0003DMG400013022,PGSC0003DMG400025126,PGSC0003DMG400003105,PGSC0003DMG400013524,PGSC0003DMG400003429,PGSC0003DMG400013545,PGSC0003DMG400003461,PGSC0003DMG400003527,PGSC0003DMG401013639,PGSC0003DMG400025613,PGSC0003DMG400013797,PGSC0003DMG400013802,PGSC0003DMG400013803,PGSC0003DMG400013804,PGSC0003DMG400013886,PGSC0003DMG400013928,PGSC0003DMG400003982,PGSC0003DMG402003983,PGSC0003DMG400003999,PGSC0003DMG400004037,PGSC0003DMG400025931,PGSC0003DMG400014213,PGSC0003DMG400026155,PGSC0003DMG400026384,PGSC0003DMG400004409,PGSC0003DMG400026433,PGSC0003DMG400026436,PGSC0003DMG400014678,PGSC0003DMG400004860,PGSC0003DMG400027124,PGSC0003DMG400004930,PGSC0003DMG400027468,PGSC0003DMG400027469,PGSC0003DMG400027771,PGSC0003DMG400015448,PGSC0003DMG400015492,PGSC0003DMG400015835 |
| 0.00147 | GO:0035639 | purine ribonucleoside triphosphate binding | PGSC0003DMG400043507,PGSC0003DMG400043830,PGSC0003DMG400044745,PGSC0003DMG400047346,PGSC0003DMG400040729,PGSC0003DMG400041071,PGSC0003DMG400041311,PGSC0003DMG400028287,PGSC0003DMG402028569,PGSC0003DMG400029520,PGSC0003DMG400029885,PGSC0003DMG400030035,PGSC0003DMG400030058,PGSC0003DMG400030113,PGSC0003DMG400030119,PGSC0003DMG400030144,PGSC0003DMG400030209,PGSC0003DMG400030220,PGSC0003DMG400030405,PGSC0003DMG400030462,PGSC0003DMG400031235,PGSC0003DMG401005729,PGSC0003DMG400031860,PGSC0003DMG400031861,PGSC0003DMG400006000,PGSC0003DMG400006022,PGSC0003DMG400032498,PGSC0003DMG400006729,PGSC0003DMG400033667,PGSC0003DMG400017106,PGSC0003DMG400017278,PGSC0003DMG400017330,PGSC0003DMG400017358,PGSC0003DMG400007634,PGSC0003DMG400017946,PGSC0003DMG400008149,PGSC0003DMG400018184,PGSC0003DMG400018457,PGSC0003DMG400018677,PGSC0003DMG400018752,PGSC0003DMG400018942,PGSC0003DMG400009078,PGSC0003DMG400009114,PGSC0003DMG400009181,PGSC0003DMG400009238,PGSC0003DMG400019721,PGSC0003DMG400019725,PGSC0003DMG400019726,PGSC0003DMG400010032,PGSC0003DMG400020564,PGSC0003DMG400010504,PGSC0003DMG400020909,PGSC0003DMG400021131,PGSC0003DMG400011071,PGSC0003DMG400021421,PGSC0003DMG401021514,PGSC0003DMG400022562,PGSC0003DMG400021993,PGSC0003DMG400000311,PGSC0003DMG400011878,PGSC0003DMG400000957,PGSC0003DMG400023814,PGSC0003DMG400024148,PGSC0003DMG400024149,PGSC0003DMG400024260,PGSC0003DMG400001853,PGSC0003DMG400002038,PGSC0003DMG400024661,PGSC0003DMG400002327,PGSC0003DMG400013022,PGSC0003DMG400025126,PGSC0003DMG400013524,PGSC0003DMG400003429,PGSC0003DMG400013545,PGSC0003DMG400025613,PGSC0003DMG400013797,PGSC0003DMG400013802,PGSC0003DMG400013803,PGSC0003DMG400013804,PGSC0003DMG400013886,PGSC0003DMG400013928,PGSC0003DMG400003982,PGSC0003DMG402003983,PGSC0003DMG400003999,PGSC0003DMG400004037,PGSC0003DMG400025931,PGSC0003DMG400014213,PGSC0003DMG400026155,PGSC0003DMG400026384,PGSC0003DMG400004409,PGSC0003DMG400026433,PGSC0003DMG400026436,PGSC0003DMG400014678,PGSC0003DMG400004860,PGSC0003DMG400027124,PGSC0003DMG400004930,PGSC0003DMG400027468,PGSC0003DMG400027469,PGSC0003DMG400027771,PGSC0003DMG400015448,PGSC0003DMG400015492,PGSC0003DMG400015835 |
| 0.0349 | GO:0000166 | nucleotide binding | PGSC0003DMG400043507,PGSC0003DMG400043830,PGSC0003DMG400044745,PGSC0003DMG400037159,PGSC0003DMG400047346,PGSC0003DMG400040729,PGSC0003DMG400041071,PGSC0003DMG400041311,PGSC0003DMG400041458,PGSC0003DMG400028287,PGSC0003DMG400028339,PGSC0003DMG402028569,PGSC0003DMG400029415,PGSC0003DMG400029520,PGSC0003DMG400029885,PGSC0003DMG400030035,PGSC0003DMG400030058,PGSC0003DMG400030113,PGSC0003DMG400030119,PGSC0003DMG400030144,PGSC0003DMG400030209,PGSC0003DMG400030220,PGSC0003DMG400030405,PGSC0003DMG400030462,PGSC0003DMG400031235,PGSC0003DMG401005729,PGSC0003DMG400031860,PGSC0003DMG400031861,PGSC0003DMG400006000,PGSC0003DMG400006022,PGSC0003DMG400032498,PGSC0003DMG400033126,PGSC0003DMG400033334,PGSC0003DMG400006729,PGSC0003DMG400006764,PGSC0003DMG400033651,PGSC0003DMG400033667,PGSC0003DMG402016981,PGSC0003DMG400017062,PGSC0003DMG400017106,PGSC0003DMG400017278,PGSC0003DMG400017330,PGSC0003DMG400017358,PGSC0003DMG400007634,PGSC0003DMG400017946,PGSC0003DMG400017976,PGSC0003DMG400008149,PGSC0003DMG400018184,PGSC0003DMG402018257,PGSC0003DMG400018428,PGSC0003DMG400018457,PGSC0003DMG400018677,PGSC0003DMG400018752,PGSC0003DMG400018942,PGSC0003DMG400009078,PGSC0003DMG400009114,PGSC0003DMG400009181,PGSC0003DMG400009238,PGSC0003DMG400019721,PGSC0003DMG400019725,PGSC0003DMG400019726,PGSC0003DMG400019804,PGSC0003DMG400009686,PGSC0003DMG400010032,PGSC0003DMG400020564,PGSC0003DMG400010504,PGSC0003DMG400020909,PGSC0003DMG400021131,PGSC0003DMG400011071,PGSC0003DMG400021421,PGSC0003DMG401021514,PGSC0003DMG400022562,PGSC0003DMG400021948,PGSC0003DMG400021993,PGSC0003DMG400000311,PGSC0003DMG400022383,PGSC0003DMG400011878,PGSC0003DMG400000957,PGSC0003DMG400001081,PGSC0003DMG400023814,PGSC0003DMG400024055,PGSC0003DMG400001448,PGSC0003DMG400024148,PGSC0003DMG400024149,PGSC0003DMG400024260,PGSC0003DMG400001853,PGSC0003DMG400001932,PGSC0003DMG400002038,PGSC0003DMG400024661,PGSC0003DMG400002327,PGSC0003DMG400002426,PGSC0003DMG400013022,PGSC0003DMG400025126,PGSC0003DMG400003105,PGSC0003DMG400013524,PGSC0003DMG400003429,PGSC0003DMG400013545,PGSC0003DMG400003461,PGSC0003DMG400003527,PGSC0003DMG401013639,PGSC0003DMG400025613,PGSC0003DMG400013797,PGSC0003DMG400013802,PGSC0003DMG400013803,PGSC0003DMG400013804,PGSC0003DMG400013886,PGSC0003DMG400013928,PGSC0003DMG400003982,PGSC0003DMG402003983,PGSC0003DMG400003999,PGSC0003DMG400004037,PGSC0003DMG400025931,PGSC0003DMG400014213,PGSC0003DMG400026155,PGSC0003DMG400026384,PGSC0003DMG400004409,PGSC0003DMG400026433,PGSC0003DMG400026436,PGSC0003DMG400014678,PGSC0003DMG400004860,PGSC0003DMG400027124,PGSC0003DMG400004930,PGSC0003DMG400027468,PGSC0003DMG400027469,PGSC0003DMG400027771,PGSC0003DMG400015448,PGSC0003DMG400015492,PGSC0003DMG400015835 |
| 0.000909 | GO:0032553 | ribonucleotide binding | PGSC0003DMG400043507,PGSC0003DMG400043830,PGSC0003DMG400044745,PGSC0003DMG400037159,PGSC0003DMG400047346,PGSC0003DMG400040729,PGSC0003DMG400041071,PGSC0003DMG400041311,PGSC0003DMG400041458,PGSC0003DMG400028287,PGSC0003DMG400028339,PGSC0003DMG402028569,PGSC0003DMG400029415,PGSC0003DMG400029520,PGSC0003DMG400029885,PGSC0003DMG400030035,PGSC0003DMG400030058,PGSC0003DMG400030113,PGSC0003DMG400030119,PGSC0003DMG400030144,PGSC0003DMG400030209,PGSC0003DMG400030220,PGSC0003DMG400030405,PGSC0003DMG400030462,PGSC0003DMG400031235,PGSC0003DMG401005729,PGSC0003DMG400031860,PGSC0003DMG400031861,PGSC0003DMG400006000,PGSC0003DMG400006022,PGSC0003DMG400032498,PGSC0003DMG400033334,PGSC0003DMG400006729,PGSC0003DMG400033651,PGSC0003DMG400033667,PGSC0003DMG402016981,PGSC0003DMG400017062,PGSC0003DMG400017106,PGSC0003DMG400017278,PGSC0003DMG400017330,PGSC0003DMG400017358,PGSC0003DMG400007634,PGSC0003DMG400017946,PGSC0003DMG400008149,PGSC0003DMG400018184,PGSC0003DMG402018257,PGSC0003DMG400018428,PGSC0003DMG400018457,PGSC0003DMG400018677,PGSC0003DMG400018752,PGSC0003DMG400018942,PGSC0003DMG400009078,PGSC0003DMG400009114,PGSC0003DMG400009181,PGSC0003DMG400009238,PGSC0003DMG400019721,PGSC0003DMG400019725,PGSC0003DMG400019726,PGSC0003DMG400019804,PGSC0003DMG400009686,PGSC0003DMG400010032,PGSC0003DMG400020564,PGSC0003DMG400010504,PGSC0003DMG400020909,PGSC0003DMG400021131,PGSC0003DMG400011071,PGSC0003DMG400021421,PGSC0003DMG401021514,PGSC0003DMG400022562,PGSC0003DMG400021948,PGSC0003DMG400021993,PGSC0003DMG400000311,PGSC0003DMG400011878,PGSC0003DMG400000957,PGSC0003DMG400001081,PGSC0003DMG400023814,PGSC0003DMG400024055,PGSC0003DMG400024148,PGSC0003DMG400024149,PGSC0003DMG400024260,PGSC0003DMG400001853,PGSC0003DMG400002038,PGSC0003DMG400024661,PGSC0003DMG400002327,PGSC0003DMG400002426,PGSC0003DMG400013022,PGSC0003DMG400025126,PGSC0003DMG400013524,PGSC0003DMG400003429,PGSC0003DMG400013545,PGSC0003DMG400003527,PGSC0003DMG400025613,PGSC0003DMG400013797,PGSC0003DMG400013802,PGSC0003DMG400013803,PGSC0003DMG400013804,PGSC0003DMG400013886,PGSC0003DMG400013928,PGSC0003DMG400003982,PGSC0003DMG402003983,PGSC0003DMG400003999,PGSC0003DMG400004037,PGSC0003DMG400025931,PGSC0003DMG400014213,PGSC0003DMG400026155,PGSC0003DMG400026384,PGSC0003DMG400004409,PGSC0003DMG400026433,PGSC0003DMG400026436,PGSC0003DMG400014678,PGSC0003DMG400004860,PGSC0003DMG400027124,PGSC0003DMG400004930,PGSC0003DMG400027468,PGSC0003DMG400027469,PGSC0003DMG400027771,PGSC0003DMG400015448,PGSC0003DMG400015492,PGSC0003DMG400015835 |
| 0.000574 | GO:0017076 | purine nucleotide binding | PGSC0003DMG400043507,PGSC0003DMG400043830,PGSC0003DMG400044745,PGSC0003DMG400037159,PGSC0003DMG400047346,PGSC0003DMG400040729,PGSC0003DMG400041071,PGSC0003DMG400041311,PGSC0003DMG400041458,PGSC0003DMG400028287,PGSC0003DMG400028339,PGSC0003DMG402028569,PGSC0003DMG400029415,PGSC0003DMG400029520,PGSC0003DMG400029885,PGSC0003DMG400030035,PGSC0003DMG400030058,PGSC0003DMG400030113,PGSC0003DMG400030119,PGSC0003DMG400030144,PGSC0003DMG400030209,PGSC0003DMG400030220,PGSC0003DMG400030405,PGSC0003DMG400030462,PGSC0003DMG400031235,PGSC0003DMG401005729,PGSC0003DMG400031860,PGSC0003DMG400031861,PGSC0003DMG400006000,PGSC0003DMG400006022,PGSC0003DMG400032498,PGSC0003DMG400033334,PGSC0003DMG400006729,PGSC0003DMG400033651,PGSC0003DMG400033667,PGSC0003DMG402016981,PGSC0003DMG400017062,PGSC0003DMG400017106,PGSC0003DMG400017278,PGSC0003DMG400017330,PGSC0003DMG400017358,PGSC0003DMG400007634,PGSC0003DMG400017946,PGSC0003DMG400008149,PGSC0003DMG400018184,PGSC0003DMG402018257,PGSC0003DMG400018428,PGSC0003DMG400018457,PGSC0003DMG400018677,PGSC0003DMG400018752,PGSC0003DMG400018942,PGSC0003DMG400009078,PGSC0003DMG400009114,PGSC0003DMG400009181,PGSC0003DMG400009238,PGSC0003DMG400019721,PGSC0003DMG400019725,PGSC0003DMG400019726,PGSC0003DMG400019804,PGSC0003DMG400009686,PGSC0003DMG400010032,PGSC0003DMG400020564,PGSC0003DMG400010504,PGSC0003DMG400020909,PGSC0003DMG400021131,PGSC0003DMG400011071,PGSC0003DMG400021421,PGSC0003DMG401021514,PGSC0003DMG400022562,PGSC0003DMG400021948,PGSC0003DMG400021993,PGSC0003DMG400000311,PGSC0003DMG400022383,PGSC0003DMG400011878,PGSC0003DMG400000957,PGSC0003DMG400001081,PGSC0003DMG400023814,PGSC0003DMG400024055,PGSC0003DMG400024148,PGSC0003DMG400024149,PGSC0003DMG400024260,PGSC0003DMG400001853,PGSC0003DMG400002038,PGSC0003DMG400024661,PGSC0003DMG400002327,PGSC0003DMG400002426,PGSC0003DMG400013022,PGSC0003DMG400025126,PGSC0003DMG400013524,PGSC0003DMG400003429,PGSC0003DMG400013545,PGSC0003DMG400003527,PGSC0003DMG400025613,PGSC0003DMG400013797,PGSC0003DMG400013802,PGSC0003DMG400013803,PGSC0003DMG400013804,PGSC0003DMG400013886,PGSC0003DMG400013928,PGSC0003DMG400003982,PGSC0003DMG402003983,PGSC0003DMG400003999,PGSC0003DMG400004037,PGSC0003DMG400025931,PGSC0003DMG400014213,PGSC0003DMG400026155,PGSC0003DMG400026384,PGSC0003DMG400004409,PGSC0003DMG400026433,PGSC0003DMG400026436,PGSC0003DMG400014678,PGSC0003DMG400004860,PGSC0003DMG400027124,PGSC0003DMG400004930,PGSC0003DMG400027468,PGSC0003DMG400027469,PGSC0003DMG400027771,PGSC0003DMG400015448,PGSC0003DMG400015492,PGSC0003DMG400015835 |
| 0.000486 | GO:0032555 | purine ribonucleotide binding | PGSC0003DMG400043507,PGSC0003DMG400043830,PGSC0003DMG400044745,PGSC0003DMG400037159,PGSC0003DMG400047346,PGSC0003DMG400040729,PGSC0003DMG400041071,PGSC0003DMG400041311,PGSC0003DMG400041458,PGSC0003DMG400028287,PGSC0003DMG400028339,PGSC0003DMG402028569,PGSC0003DMG400029415,PGSC0003DMG400029520,PGSC0003DMG400029885,PGSC0003DMG400030035,PGSC0003DMG400030058,PGSC0003DMG400030113,PGSC0003DMG400030119,PGSC0003DMG400030144,PGSC0003DMG400030209,PGSC0003DMG400030220,PGSC0003DMG400030405,PGSC0003DMG400030462,PGSC0003DMG400031235,PGSC0003DMG401005729,PGSC0003DMG400031860,PGSC0003DMG400031861,PGSC0003DMG400006000,PGSC0003DMG400006022,PGSC0003DMG400032498,PGSC0003DMG400033334,PGSC0003DMG400006729,PGSC0003DMG400033651,PGSC0003DMG400033667,PGSC0003DMG402016981,PGSC0003DMG400017062,PGSC0003DMG400017106,PGSC0003DMG400017278,PGSC0003DMG400017330,PGSC0003DMG400017358,PGSC0003DMG400007634,PGSC0003DMG400017946,PGSC0003DMG400008149,PGSC0003DMG400018184,PGSC0003DMG402018257,PGSC0003DMG400018428,PGSC0003DMG400018457,PGSC0003DMG400018677,PGSC0003DMG400018752,PGSC0003DMG400018942,PGSC0003DMG400009078,PGSC0003DMG400009114,PGSC0003DMG400009181,PGSC0003DMG400009238,PGSC0003DMG400019721,PGSC0003DMG400019725,PGSC0003DMG400019726,PGSC0003DMG400019804,PGSC0003DMG400009686,PGSC0003DMG400010032,PGSC0003DMG400020564,PGSC0003DMG400010504,PGSC0003DMG400020909,PGSC0003DMG400021131,PGSC0003DMG400011071,PGSC0003DMG400021421,PGSC0003DMG401021514,PGSC0003DMG400022562,PGSC0003DMG400021948,PGSC0003DMG400021993,PGSC0003DMG400000311,PGSC0003DMG400011878,PGSC0003DMG400000957,PGSC0003DMG400001081,PGSC0003DMG400023814,PGSC0003DMG400024055,PGSC0003DMG400024148,PGSC0003DMG400024149,PGSC0003DMG400024260,PGSC0003DMG400001853,PGSC0003DMG400002038,PGSC0003DMG400024661,PGSC0003DMG400002327,PGSC0003DMG400002426,PGSC0003DMG400013022,PGSC0003DMG400025126,PGSC0003DMG400013524,PGSC0003DMG400003429,PGSC0003DMG400013545,PGSC0003DMG400003527,PGSC0003DMG400025613,PGSC0003DMG400013797,PGSC0003DMG400013802,PGSC0003DMG400013803,PGSC0003DMG400013804,PGSC0003DMG400013886,PGSC0003DMG400013928,PGSC0003DMG400003982,PGSC0003DMG402003983,PGSC0003DMG400003999,PGSC0003DMG400004037,PGSC0003DMG400025931,PGSC0003DMG400014213,PGSC0003DMG400026155,PGSC0003DMG400026384,PGSC0003DMG400004409,PGSC0003DMG400026433,PGSC0003DMG400026436,PGSC0003DMG400014678,PGSC0003DMG400004860,PGSC0003DMG400027124,PGSC0003DMG400004930,PGSC0003DMG400027468,PGSC0003DMG400027469,PGSC0003DMG400027771,PGSC0003DMG400015448,PGSC0003DMG400015492,PGSC0003DMG400015835 |
| 1.53E-05 | GO:0030554 | adenyl nucleotide binding | PGSC0003DMG400043507,PGSC0003DMG400043830,PGSC0003DMG400044745,PGSC0003DMG400037159,PGSC0003DMG400047346,PGSC0003DMG400040729,PGSC0003DMG400041071,PGSC0003DMG400041311,PGSC0003DMG400041458,PGSC0003DMG400028287,PGSC0003DMG400028339,PGSC0003DMG402028569,PGSC0003DMG400029415,PGSC0003DMG400029520,PGSC0003DMG400029885,PGSC0003DMG400030035,PGSC0003DMG400030058,PGSC0003DMG400030113,PGSC0003DMG400030119,PGSC0003DMG400030144,PGSC0003DMG400030209,PGSC0003DMG400030220,PGSC0003DMG400030405,PGSC0003DMG400030462,PGSC0003DMG400031235,PGSC0003DMG401005729,PGSC0003DMG400031860,PGSC0003DMG400031861,PGSC0003DMG400006000,PGSC0003DMG400006022,PGSC0003DMG400032498,PGSC0003DMG400033334,PGSC0003DMG400006729,PGSC0003DMG400033651,PGSC0003DMG400033667,PGSC0003DMG402016981,PGSC0003DMG400017062,PGSC0003DMG400017106,PGSC0003DMG400017278,PGSC0003DMG400017330,PGSC0003DMG400017358,PGSC0003DMG400007634,PGSC0003DMG400017946,PGSC0003DMG400008149,PGSC0003DMG400018184,PGSC0003DMG402018257,PGSC0003DMG400018428,PGSC0003DMG400018457,PGSC0003DMG400018677,PGSC0003DMG400018752,PGSC0003DMG400018942,PGSC0003DMG400009078,PGSC0003DMG400009114,PGSC0003DMG400009181,PGSC0003DMG400009238,PGSC0003DMG400019721,PGSC0003DMG400019725,PGSC0003DMG400019726,PGSC0003DMG400019804,PGSC0003DMG400009686,PGSC0003DMG400010032,PGSC0003DMG400020564,PGSC0003DMG400010504,PGSC0003DMG400020909,PGSC0003DMG400021131,PGSC0003DMG400011071,PGSC0003DMG400021421,PGSC0003DMG401021514,PGSC0003DMG400022562,PGSC0003DMG400021948,PGSC0003DMG400021993,PGSC0003DMG400000311,PGSC0003DMG400022383,PGSC0003DMG400011878,PGSC0003DMG400000957,PGSC0003DMG400001081,PGSC0003DMG400023814,PGSC0003DMG400024055,PGSC0003DMG400024148,PGSC0003DMG400024149,PGSC0003DMG400024260,PGSC0003DMG400001853,PGSC0003DMG400002038,PGSC0003DMG400024661,PGSC0003DMG400002327,PGSC0003DMG400002426,PGSC0003DMG400013022,PGSC0003DMG400025126,PGSC0003DMG400013524,PGSC0003DMG400003429,PGSC0003DMG400013545,PGSC0003DMG400003527,PGSC0003DMG400025613,PGSC0003DMG400013797,PGSC0003DMG400013802,PGSC0003DMG400013803,PGSC0003DMG400013804,PGSC0003DMG400013886,PGSC0003DMG400013928,PGSC0003DMG400003982,PGSC0003DMG402003983,PGSC0003DMG400003999,PGSC0003DMG400004037,PGSC0003DMG400014213,PGSC0003DMG400026155,PGSC0003DMG400026384,PGSC0003DMG400004409,PGSC0003DMG400026433,PGSC0003DMG400026436,PGSC0003DMG400014678,PGSC0003DMG400004860,PGSC0003DMG400027124,PGSC0003DMG400004930,PGSC0003DMG400027468,PGSC0003DMG400027469,PGSC0003DMG400027771,PGSC0003DMG400015448,PGSC0003DMG400015492,PGSC0003DMG400015835 |
| 1.21E-05 | GO:0032559 | adenyl ribonucleotide binding | PGSC0003DMG400043507,PGSC0003DMG400043830,PGSC0003DMG400044745,PGSC0003DMG400037159,PGSC0003DMG400047346,PGSC0003DMG400040729,PGSC0003DMG400041071,PGSC0003DMG400041311,PGSC0003DMG400041458,PGSC0003DMG400028287,PGSC0003DMG400028339,PGSC0003DMG402028569,PGSC0003DMG400029415,PGSC0003DMG400029520,PGSC0003DMG400029885,PGSC0003DMG400030035,PGSC0003DMG400030058,PGSC0003DMG400030113,PGSC0003DMG400030119,PGSC0003DMG400030144,PGSC0003DMG400030209,PGSC0003DMG400030220,PGSC0003DMG400030405,PGSC0003DMG400030462,PGSC0003DMG400031235,PGSC0003DMG401005729,PGSC0003DMG400031860,PGSC0003DMG400031861,PGSC0003DMG400006000,PGSC0003DMG400006022,PGSC0003DMG400032498,PGSC0003DMG400033334,PGSC0003DMG400006729,PGSC0003DMG400033651,PGSC0003DMG400033667,PGSC0003DMG402016981,PGSC0003DMG400017062,PGSC0003DMG400017106,PGSC0003DMG400017278,PGSC0003DMG400017330,PGSC0003DMG400017358,PGSC0003DMG400007634,PGSC0003DMG400017946,PGSC0003DMG400008149,PGSC0003DMG400018184,PGSC0003DMG402018257,PGSC0003DMG400018428,PGSC0003DMG400018457,PGSC0003DMG400018677,PGSC0003DMG400018752,PGSC0003DMG400018942,PGSC0003DMG400009078,PGSC0003DMG400009114,PGSC0003DMG400009181,PGSC0003DMG400009238,PGSC0003DMG400019721,PGSC0003DMG400019725,PGSC0003DMG400019726,PGSC0003DMG400019804,PGSC0003DMG400009686,PGSC0003DMG400010032,PGSC0003DMG400020564,PGSC0003DMG400010504,PGSC0003DMG400020909,PGSC0003DMG400021131,PGSC0003DMG400011071,PGSC0003DMG400021421,PGSC0003DMG401021514,PGSC0003DMG400022562,PGSC0003DMG400021948,PGSC0003DMG400021993,PGSC0003DMG400000311,PGSC0003DMG400011878,PGSC0003DMG400000957,PGSC0003DMG400001081,PGSC0003DMG400023814,PGSC0003DMG400024055,PGSC0003DMG400024148,PGSC0003DMG400024149,PGSC0003DMG400024260,PGSC0003DMG400001853,PGSC0003DMG400002038,PGSC0003DMG400024661,PGSC0003DMG400002327,PGSC0003DMG400002426,PGSC0003DMG400013022,PGSC0003DMG400025126,PGSC0003DMG400013524,PGSC0003DMG400003429,PGSC0003DMG400013545,PGSC0003DMG400003527,PGSC0003DMG400025613,PGSC0003DMG400013797,PGSC0003DMG400013802,PGSC0003DMG400013803,PGSC0003DMG400013804,PGSC0003DMG400013886,PGSC0003DMG400013928,PGSC0003DMG400003982,PGSC0003DMG402003983,PGSC0003DMG400003999,PGSC0003DMG400004037,PGSC0003DMG400014213,PGSC0003DMG400026155,PGSC0003DMG400026384,PGSC0003DMG400004409,PGSC0003DMG400026433,PGSC0003DMG400026436,PGSC0003DMG400014678,PGSC0003DMG400004860,PGSC0003DMG400027124,PGSC0003DMG400004930,PGSC0003DMG400027468,PGSC0003DMG400027469,PGSC0003DMG400027771,PGSC0003DMG400015448,PGSC0003DMG400015492,PGSC0003DMG400015835 |
| 3.36E-05 | GO:0005524 | ATP binding | PGSC0003DMG400043507,PGSC0003DMG400043830,PGSC0003DMG400044745,PGSC0003DMG400047346,PGSC0003DMG400040729,PGSC0003DMG400041071,PGSC0003DMG400041311,PGSC0003DMG400028287,PGSC0003DMG402028569,PGSC0003DMG400029520,PGSC0003DMG400029885,PGSC0003DMG400030035,PGSC0003DMG400030058,PGSC0003DMG400030113,PGSC0003DMG400030119,PGSC0003DMG400030144,PGSC0003DMG400030209,PGSC0003DMG400030220,PGSC0003DMG400030405,PGSC0003DMG400030462,PGSC0003DMG400031235,PGSC0003DMG401005729,PGSC0003DMG400031860,PGSC0003DMG400031861,PGSC0003DMG400006000,PGSC0003DMG400006022,PGSC0003DMG400032498,PGSC0003DMG400006729,PGSC0003DMG400033667,PGSC0003DMG400017106,PGSC0003DMG400017278,PGSC0003DMG400017330,PGSC0003DMG400017358,PGSC0003DMG400007634,PGSC0003DMG400017946,PGSC0003DMG400008149,PGSC0003DMG400018184,PGSC0003DMG400018457,PGSC0003DMG400018677,PGSC0003DMG400018752,PGSC0003DMG400018942,PGSC0003DMG400009078,PGSC0003DMG400009114,PGSC0003DMG400009181,PGSC0003DMG400009238,PGSC0003DMG400019721,PGSC0003DMG400019725,PGSC0003DMG400019726,PGSC0003DMG400010032,PGSC0003DMG400020564,PGSC0003DMG400010504,PGSC0003DMG400020909,PGSC0003DMG400021131,PGSC0003DMG400011071,PGSC0003DMG400021421,PGSC0003DMG401021514,PGSC0003DMG400022562,PGSC0003DMG400021993,PGSC0003DMG400000311,PGSC0003DMG400011878,PGSC0003DMG400000957,PGSC0003DMG400023814,PGSC0003DMG400024148,PGSC0003DMG400024149,PGSC0003DMG400024260,PGSC0003DMG400001853,PGSC0003DMG400002038,PGSC0003DMG400024661,PGSC0003DMG400002327,PGSC0003DMG400013022,PGSC0003DMG400025126,PGSC0003DMG400013524,PGSC0003DMG400003429,PGSC0003DMG400013545,PGSC0003DMG400025613,PGSC0003DMG400013797,PGSC0003DMG400013802,PGSC0003DMG400013803,PGSC0003DMG400013804,PGSC0003DMG400013886,PGSC0003DMG400013928,PGSC0003DMG400003982,PGSC0003DMG402003983,PGSC0003DMG400003999,PGSC0003DMG400004037,PGSC0003DMG400014213,PGSC0003DMG400026155,PGSC0003DMG400026384,PGSC0003DMG400004409,PGSC0003DMG400026433,PGSC0003DMG400026436,PGSC0003DMG400014678,PGSC0003DMG400004860,PGSC0003DMG400027124,PGSC0003DMG400004930,PGSC0003DMG400027468,PGSC0003DMG400027469,PGSC0003DMG400027771,PGSC0003DMG400015448,PGSC0003DMG400015492,PGSC0003DMG400015835 |
| 0.00696 | GO:0008792 | arginine decarboxylase activity | PGSC0003DMG400001662,PGSC0003DMG400026671 |
| 0.00722 | GO:0004721 | phosphoprotein phosphatase activity | PGSC0003DMG400030364,PGSC0003DMG400030410,PGSC0003DMG400032820,PGSC0003DMG400007258,PGSC0003DMG400020541,PGSC0003DMG400024772,PGSC0003DMG400024944,PGSC0003DMG400025756,PGSC0003DMG400027174,PGSC0003DMG400005213,PGSC0003DMG400005456,PGSC0003DMG400016290 |
| 0.00175 | GO:0004722 | protein serine/threonine phosphatase activity | PGSC0003DMG400030364,PGSC0003DMG400030410,PGSC0003DMG400032820,PGSC0003DMG400020541,PGSC0003DMG400024772,PGSC0003DMG400024944,PGSC0003DMG400025756,PGSC0003DMG400027174,PGSC0003DMG400005213,PGSC0003DMG400005456 |
| 5.57E-06 | GO:0005509 | calcium ion binding | PGSC0003DMG400028797,PGSC0003DMG400030608,PGSC0003DMG400030892,PGSC0003DMG400005745,PGSC0003DMG400005909,PGSC0003DMG400006729,PGSC0003DMG400033685,PGSC0003DMG400017106,PGSC0003DMG400008149,PGSC0003DMG400008163,PGSC0003DMG402009727,PGSC0003DMG400020261,PGSC0003DMG400010410,PGSC0003DMG400010631,PGSC0003DMG400022562,PGSC0003DMG400022340,PGSC0003DMG400023028,PGSC0003DMG400001333,PGSC0003DMG400025022,PGSC0003DMG400025023,PGSC0003DMG400014168,PGSC0003DMG400026159,PGSC0003DMG400027692,PGSC0003DMG400016011 |
| 0.0276 | GO:0016830 | carbon-carbon lyase activity | PGSC0003DMG400029046,PGSC0003DMG400029050,PGSC0003DMG400022263,PGSC0003DMG400012012,PGSC0003DMG400001662,PGSC0003DMG400012571,PGSC0003DMG400003123,PGSC0003DMG400026671,PGSC0003DMG400014779 |
| 0.0287 | GO:0016832 | aldehyde-lyase activity | PGSC0003DMG400022263,PGSC0003DMG400012012,PGSC0003DMG400003123 |
| 0.0104 | GO:0004332 | fructose-bisphosphate aldolase activity | PGSC0003DMG400022263,PGSC0003DMG400012012,PGSC0003DMG400003123 |
| 6.12E-05 | GO:0016740 | transferase activity | PGSC0003DMG400044745,PGSC0003DMG400045432,PGSC0003DMG400047346,PGSC0003DMG400038970,PGSC0003DMG400040729,PGSC0003DMG400041071,PGSC0003DMG400041311,PGSC0003DMG400028287,PGSC0003DMG400028462,PGSC0003DMG400029520,PGSC0003DMG401029824,PGSC0003DMG401029843,PGSC0003DMG400029885,PGSC0003DMG400030035,PGSC0003DMG400030058,PGSC0003DMG400030113,PGSC0003DMG400030119,PGSC0003DMG400030144,PGSC0003DMG400030209,PGSC0003DMG400030462,PGSC0003DMG400031235,PGSC0003DMG400031454,PGSC0003DMG401005729,PGSC0003DMG400031860,PGSC0003DMG400031861,PGSC0003DMG400006000,PGSC0003DMG400006022,PGSC0003DMG400032272,PGSC0003DMG400032273,PGSC0003DMG400032498,PGSC0003DMG400033126,PGSC0003DMG400006640,PGSC0003DMG400006729,PGSC0003DMG400006814,PGSC0003DMG400033667,PGSC0003DMG400016880,PGSC0003DMG402016888,PGSC0003DMG400017106,PGSC0003DMG400017278,PGSC0003DMG400017299,PGSC0003DMG400017301,PGSC0003DMG400017330,PGSC0003DMG400017358,PGSC0003DMG400007634,PGSC0003DMG400017946,PGSC0003DMG400008149,PGSC0003DMG400018184,PGSC0003DMG401018380,PGSC0003DMG400018457,PGSC0003DMG400018677,PGSC0003DMG400018752,PGSC0003DMG400009078,PGSC0003DMG400009114,PGSC0003DMG400019253,PGSC0003DMG400009181,PGSC0003DMG400009238,PGSC0003DMG400019526,PGSC0003DMG400019721,PGSC0003DMG400019725,PGSC0003DMG400019726,PGSC0003DMG400019883,PGSC0003DMG400009699,PGSC0003DMG400010032,PGSC0003DMG400020564,PGSC0003DMG400010504,PGSC0003DMG400020896,PGSC0003DMG400020909,PGSC0003DMG400021116,PGSC0003DMG400011071,PGSC0003DMG401021514,PGSC0003DMG400021555,PGSC0003DMG400022562,PGSC0003DMG400021993,PGSC0003DMG400022775,PGSC0003DMG400000114,PGSC0003DMG400011624,PGSC0003DMG400011740,PGSC0003DMG400011752,PGSC0003DMG400011872,PGSC0003DMG400000791,PGSC0003DMG400011878,PGSC0003DMG400023731,PGSC0003DMG400023814,PGSC0003DMG400001396,PGSC0003DMG400001444,PGSC0003DMG400024148,PGSC0003DMG400024149,PGSC0003DMG400024260,PGSC0003DMG400024281,PGSC0003DMG400001853,PGSC0003DMG400024491,PGSC0003DMG400002038,PGSC0003DMG400002167,PGSC0003DMG400024661,PGSC0003DMG400024667,PGSC0003DMG400002327,PGSC0003DMG400012635,PGSC0003DMG401003156,PGSC0003DMG400013524,PGSC0003DMG400003429,PGSC0003DMG400013545,PGSC0003DMG400025613,PGSC0003DMG400013797,PGSC0003DMG400013802,PGSC0003DMG400013803,PGSC0003DMG400013804,PGSC0003DMG400013886,PGSC0003DMG400003822,PGSC0003DMG400013928,PGSC0003DMG400025877,PGSC0003DMG400003982,PGSC0003DMG402003983,PGSC0003DMG400025884,PGSC0003DMG400003999,PGSC0003DMG400004037,PGSC0003DMG400014213,PGSC0003DMG400026127,PGSC0003DMG400026155,PGSC0003DMG400026322,PGSC0003DMG400026323,PGSC0003DMG400026384,PGSC0003DMG400004409,PGSC0003DMG400026436,PGSC0003DMG400026593,PGSC0003DMG400014555,PGSC0003DMG400014678,PGSC0003DMG400014770,PGSC0003DMG400014771,PGSC0003DMG400014774,PGSC0003DMG400014776,PGSC0003DMG400014778,PGSC0003DMG400014823,PGSC0003DMG400004860,PGSC0003DMG400027124,PGSC0003DMG400004930,PGSC0003DMG400027338,PGSC0003DMG400027468,PGSC0003DMG400027469,PGSC0003DMG400027771,PGSC0003DMG400015448,PGSC0003DMG400015552,PGSC0003DMG400015835 |
| 1.93E-07 | GO:0016772 | transferase activity, transferring phosphorus-containing groups | PGSC0003DMG400044745,PGSC0003DMG400047346,PGSC0003DMG400040729,PGSC0003DMG400041071,PGSC0003DMG400041311,PGSC0003DMG400029520,PGSC0003DMG400029885,PGSC0003DMG400030035,PGSC0003DMG400030058,PGSC0003DMG400030113,PGSC0003DMG400030119,PGSC0003DMG400030144,PGSC0003DMG400030209,PGSC0003DMG400030462,PGSC0003DMG400031235,PGSC0003DMG401005729,PGSC0003DMG400031860,PGSC0003DMG400031861,PGSC0003DMG400006000,PGSC0003DMG400006022,PGSC0003DMG400032498,PGSC0003DMG400033126,PGSC0003DMG400006729,PGSC0003DMG400033667,PGSC0003DMG400017106,PGSC0003DMG400017278,PGSC0003DMG400017330,PGSC0003DMG400017358,PGSC0003DMG400007634,PGSC0003DMG400017946,PGSC0003DMG400008149,PGSC0003DMG400018184,PGSC0003DMG401018380,PGSC0003DMG400018457,PGSC0003DMG400018677,PGSC0003DMG400018752,PGSC0003DMG400009078,PGSC0003DMG400009114,PGSC0003DMG400009181,PGSC0003DMG400009238,PGSC0003DMG400019721,PGSC0003DMG400019725,PGSC0003DMG400019726,PGSC0003DMG400009699,PGSC0003DMG400010032,PGSC0003DMG400020564,PGSC0003DMG400010504,PGSC0003DMG400020909,PGSC0003DMG400011071,PGSC0003DMG401021514,PGSC0003DMG400022562,PGSC0003DMG400021993,PGSC0003DMG400000114,PGSC0003DMG400011878,PGSC0003DMG400023814,PGSC0003DMG400024148,PGSC0003DMG400024149,PGSC0003DMG400024260,PGSC0003DMG400001853,PGSC0003DMG400002038,PGSC0003DMG400024661,PGSC0003DMG400002327,PGSC0003DMG400013524,PGSC0003DMG400003429,PGSC0003DMG400013545,PGSC0003DMG400025613,PGSC0003DMG400013797,PGSC0003DMG400013802,PGSC0003DMG400013803,PGSC0003DMG400013804,PGSC0003DMG400013886,PGSC0003DMG400013928,PGSC0003DMG400003982,PGSC0003DMG402003983,PGSC0003DMG400003999,PGSC0003DMG400004037,PGSC0003DMG400014213,PGSC0003DMG400026155,PGSC0003DMG400026384,PGSC0003DMG400004409,PGSC0003DMG400026436,PGSC0003DMG400014678,PGSC0003DMG400004860,PGSC0003DMG400027124,PGSC0003DMG400004930,PGSC0003DMG400027468,PGSC0003DMG400027469,PGSC0003DMG400027771,PGSC0003DMG400015448,PGSC0003DMG400015835 |
| 7.8E-11 | GO:0016301 | kinase activity | PGSC0003DMG400044745,PGSC0003DMG400047346,PGSC0003DMG400040729,PGSC0003DMG400041071,PGSC0003DMG400041311,PGSC0003DMG400029520,PGSC0003DMG400029885,PGSC0003DMG400030035,PGSC0003DMG400030058,PGSC0003DMG400030113,PGSC0003DMG400030119,PGSC0003DMG400030144,PGSC0003DMG400030209,PGSC0003DMG400030462,PGSC0003DMG400031235,PGSC0003DMG401005729,PGSC0003DMG400031860,PGSC0003DMG400031861,PGSC0003DMG400006000,PGSC0003DMG400006022,PGSC0003DMG400032498,PGSC0003DMG400033126,PGSC0003DMG400006729,PGSC0003DMG400033667,PGSC0003DMG400017106,PGSC0003DMG400017278,PGSC0003DMG400017330,PGSC0003DMG400017358,PGSC0003DMG400007634,PGSC0003DMG400017946,PGSC0003DMG400008149,PGSC0003DMG400018184,PGSC0003DMG401018380,PGSC0003DMG400018457,PGSC0003DMG400018677,PGSC0003DMG400018752,PGSC0003DMG400009078,PGSC0003DMG400009114,PGSC0003DMG400009181,PGSC0003DMG400009238,PGSC0003DMG400019721,PGSC0003DMG400019725,PGSC0003DMG400019726,PGSC0003DMG400009699,PGSC0003DMG400010032,PGSC0003DMG400020564,PGSC0003DMG400010504,PGSC0003DMG400020909,PGSC0003DMG400011071,PGSC0003DMG401021514,PGSC0003DMG400022562,PGSC0003DMG400021993,PGSC0003DMG400000114,PGSC0003DMG400011878,PGSC0003DMG400023814,PGSC0003DMG400024148,PGSC0003DMG400024149,PGSC0003DMG400024260,PGSC0003DMG400001853,PGSC0003DMG400002038,PGSC0003DMG400024661,PGSC0003DMG400002327,PGSC0003DMG400013524,PGSC0003DMG400003429,PGSC0003DMG400013545,PGSC0003DMG400025613,PGSC0003DMG400013797,PGSC0003DMG400013802,PGSC0003DMG400013803,PGSC0003DMG400013804,PGSC0003DMG400013886,PGSC0003DMG400013928,PGSC0003DMG400003982,PGSC0003DMG402003983,PGSC0003DMG400003999,PGSC0003DMG400004037,PGSC0003DMG400014213,PGSC0003DMG400026155,PGSC0003DMG400026384,PGSC0003DMG400004409,PGSC0003DMG400026436,PGSC0003DMG400014678,PGSC0003DMG400004860,PGSC0003DMG400027124,PGSC0003DMG400004930,PGSC0003DMG400027468,PGSC0003DMG400027469,PGSC0003DMG400027771,PGSC0003DMG400015448,PGSC0003DMG400015835 |
| 6.02E-11 | GO:0016773 | phosphotransferase activity, alcohol group as acceptor | PGSC0003DMG400044745,PGSC0003DMG400047346,PGSC0003DMG400040729,PGSC0003DMG400041071,PGSC0003DMG400041311,PGSC0003DMG400029520,PGSC0003DMG400029885,PGSC0003DMG400030035,PGSC0003DMG400030058,PGSC0003DMG400030113,PGSC0003DMG400030119,PGSC0003DMG400030144,PGSC0003DMG400030209,PGSC0003DMG400030462,PGSC0003DMG400031235,PGSC0003DMG401005729,PGSC0003DMG400031860,PGSC0003DMG400031861,PGSC0003DMG400006000,PGSC0003DMG400006022,PGSC0003DMG400032498,PGSC0003DMG400006729,PGSC0003DMG400033667,PGSC0003DMG400017106,PGSC0003DMG400017278,PGSC0003DMG400017330,PGSC0003DMG400017358,PGSC0003DMG400007634,PGSC0003DMG400017946,PGSC0003DMG400008149,PGSC0003DMG400018184,PGSC0003DMG401018380,PGSC0003DMG400018457,PGSC0003DMG400018677,PGSC0003DMG400018752,PGSC0003DMG400009078,PGSC0003DMG400009114,PGSC0003DMG400009181,PGSC0003DMG400009238,PGSC0003DMG400019721,PGSC0003DMG400019725,PGSC0003DMG400019726,PGSC0003DMG400010032,PGSC0003DMG400020564,PGSC0003DMG400010504,PGSC0003DMG400020909,PGSC0003DMG400011071,PGSC0003DMG401021514,PGSC0003DMG400022562,PGSC0003DMG400021993,PGSC0003DMG400000114,PGSC0003DMG400011878,PGSC0003DMG400023814,PGSC0003DMG400024148,PGSC0003DMG400024149,PGSC0003DMG400024260,PGSC0003DMG400001853,PGSC0003DMG400002038,PGSC0003DMG400024661,PGSC0003DMG400002327,PGSC0003DMG400013524,PGSC0003DMG400003429,PGSC0003DMG400013545,PGSC0003DMG400025613,PGSC0003DMG400013797,PGSC0003DMG400013802,PGSC0003DMG400013803,PGSC0003DMG400013804,PGSC0003DMG400013886,PGSC0003DMG400013928,PGSC0003DMG400003982,PGSC0003DMG402003983,PGSC0003DMG400003999,PGSC0003DMG400004037,PGSC0003DMG400014213,PGSC0003DMG400026155,PGSC0003DMG400026384,PGSC0003DMG400004409,PGSC0003DMG400026436,PGSC0003DMG400014678,PGSC0003DMG400004860,PGSC0003DMG400027124,PGSC0003DMG400004930,PGSC0003DMG400027468,PGSC0003DMG400027469,PGSC0003DMG400027771,PGSC0003DMG400015448,PGSC0003DMG400015835 |
| 1.39E-12 | GO:0004672 | protein kinase activity | PGSC0003DMG400044745,PGSC0003DMG400047346,PGSC0003DMG400040729,PGSC0003DMG400041071,PGSC0003DMG400041311,PGSC0003DMG400029520,PGSC0003DMG400029885,PGSC0003DMG400030035,PGSC0003DMG400030058,PGSC0003DMG400030113,PGSC0003DMG400030119,PGSC0003DMG400030144,PGSC0003DMG400030209,PGSC0003DMG400030462,PGSC0003DMG400031235,PGSC0003DMG401005729,PGSC0003DMG400031860,PGSC0003DMG400031861,PGSC0003DMG400006000,PGSC0003DMG400006022,PGSC0003DMG400032498,PGSC0003DMG400006729,PGSC0003DMG400033667,PGSC0003DMG400017106,PGSC0003DMG400017278,PGSC0003DMG400017330,PGSC0003DMG400017358,PGSC0003DMG400007634,PGSC0003DMG400017946,PGSC0003DMG400008149,PGSC0003DMG400018184,PGSC0003DMG400018457,PGSC0003DMG400018677,PGSC0003DMG400018752,PGSC0003DMG400009078,PGSC0003DMG400009114,PGSC0003DMG400009181,PGSC0003DMG400009238,PGSC0003DMG400019721,PGSC0003DMG400019725,PGSC0003DMG400019726,PGSC0003DMG400010032,PGSC0003DMG400020564,PGSC0003DMG400010504,PGSC0003DMG400020909,PGSC0003DMG400011071,PGSC0003DMG401021514,PGSC0003DMG400022562,PGSC0003DMG400021993,PGSC0003DMG400011878,PGSC0003DMG400023814,PGSC0003DMG400024148,PGSC0003DMG400024149,PGSC0003DMG400024260,PGSC0003DMG400001853,PGSC0003DMG400002038,PGSC0003DMG400024661,PGSC0003DMG400002327,PGSC0003DMG400013524,PGSC0003DMG400003429,PGSC0003DMG400013545,PGSC0003DMG400025613,PGSC0003DMG400013797,PGSC0003DMG400013802,PGSC0003DMG400013803,PGSC0003DMG400013804,PGSC0003DMG400013886,PGSC0003DMG400013928,PGSC0003DMG400003982,PGSC0003DMG402003983,PGSC0003DMG400003999,PGSC0003DMG400004037,PGSC0003DMG400014213,PGSC0003DMG400026155,PGSC0003DMG400026384,PGSC0003DMG400004409,PGSC0003DMG400026436,PGSC0003DMG400014678,PGSC0003DMG400004860,PGSC0003DMG400027124,PGSC0003DMG400004930,PGSC0003DMG400027468,PGSC0003DMG400027469,PGSC0003DMG400027771,PGSC0003DMG400015448,PGSC0003DMG400015835 |
| 5.99E-12 | GO:0004674 | protein serine/threonine kinase activity | PGSC0003DMG400044745,PGSC0003DMG400041071,PGSC0003DMG400041311,PGSC0003DMG400029520,PGSC0003DMG400029885,PGSC0003DMG400030058,PGSC0003DMG400030113,PGSC0003DMG400030119,PGSC0003DMG400030144,PGSC0003DMG400030209,PGSC0003DMG400030462,PGSC0003DMG401005729,PGSC0003DMG400031860,PGSC0003DMG400031861,PGSC0003DMG400006000,PGSC0003DMG400006022,PGSC0003DMG400032498,PGSC0003DMG400006729,PGSC0003DMG400033667,PGSC0003DMG400017106,PGSC0003DMG400017278,PGSC0003DMG400017330,PGSC0003DMG400017358,PGSC0003DMG400007634,PGSC0003DMG400008149,PGSC0003DMG400018184,PGSC0003DMG400018457,PGSC0003DMG400018677,PGSC0003DMG400018752,PGSC0003DMG400009078,PGSC0003DMG400009114,PGSC0003DMG400009181,PGSC0003DMG400019721,PGSC0003DMG400019725,PGSC0003DMG400019726,PGSC0003DMG400010032,PGSC0003DMG400020564,PGSC0003DMG400020909,PGSC0003DMG400011071,PGSC0003DMG400022562,PGSC0003DMG400021993,PGSC0003DMG400011878,PGSC0003DMG400023814,PGSC0003DMG400024149,PGSC0003DMG400024260,PGSC0003DMG400001853,PGSC0003DMG400002038,PGSC0003DMG400024661,PGSC0003DMG400002327,PGSC0003DMG400013524,PGSC0003DMG400013545,PGSC0003DMG400025613,PGSC0003DMG400013797,PGSC0003DMG400013886,PGSC0003DMG400013928,PGSC0003DMG400003982,PGSC0003DMG402003983,PGSC0003DMG400003999,PGSC0003DMG400004037,PGSC0003DMG400014213,PGSC0003DMG400026155,PGSC0003DMG400026384,PGSC0003DMG400004409,PGSC0003DMG400014678,PGSC0003DMG400004860,PGSC0003DMG400027124,PGSC0003DMG400004930,PGSC0003DMG400027771,PGSC0003DMG400015835 |
| 1.05E-08 | GO:0016168 | chlorophyll binding | PGSC0003DMG400042498,PGSC0003DMG400033084,PGSC0003DMG400007375,PGSC0003DMG400007787,PGSC0003DMG400008298,PGSC0003DMG400008300,PGSC0003DMG400008301,PGSC0003DMG400008564,PGSC0003DMG400020505,PGSC0003DMG400023344,PGSC0003DMG400004301,PGSC0003DMG400004458,PGSC0003DMG400016695 |
| 0.00275 | GO:0005516 | calmodulin binding | PGSC0003DMG400005649,PGSC0003DMG400005805,PGSC0003DMG400007947,PGSC0003DMG400021421,PGSC0003DMG400013022,PGSC0003DMG400014589,PGSC0003DMG400005390 |
| 0.0349 | GO:0016628 | oxidoreductase activity, acting on the CH-CH group of donors, NAD or NADP as acceptor | PGSC0003DMG400030683,PGSC0003DMG400005931,PGSC0003DMG400020334,PGSC0003DMG400023957 |
| 0.000208 | GO:0008977 | prephenate dehydrogenase activity | PGSC0003DMG400030683,PGSC0003DMG400020334,PGSC0003DMG400023957 |
| 0.000208 | GO:0004665 | prephenate dehydrogenase (NADP+) activity | PGSC0003DMG400030683,PGSC0003DMG400020334,PGSC0003DMG400023957 |
| 0.00413 | GO:0072509 | divalent inorganic cation transmembrane transporter activity | PGSC0003DMG400043507,PGSC0003DMG400043830,PGSC0003DMG400018942,PGSC0003DMG400021421,PGSC0003DMG400013022,PGSC0003DMG400025244,PGSC0003DMG400004158 |
| 0.000149 | GO:0015085 | calcium ion transmembrane transporter activity | PGSC0003DMG400043507,PGSC0003DMG400043830,PGSC0003DMG400018942,PGSC0003DMG400021421,PGSC0003DMG400013022,PGSC0003DMG400004158 |
| 0.000432 | GO:0015662 | ATPase activity, coupled to transmembrane movement of ions, phosphorylative mechanism | PGSC0003DMG400043507,PGSC0003DMG400043830,PGSC0003DMG400018942,PGSC0003DMG400021421,PGSC0003DMG400013022 |
| 0.000123 | GO:0005388 | calcium-transporting ATPase activity | PGSC0003DMG400043507,PGSC0003DMG400043830,PGSC0003DMG400018942,PGSC0003DMG400021421,PGSC0003DMG400013022 |
| 0.0205 | GO:0016671 | oxidoreductase activity, acting on a sulfur group of donors, disulfide as acceptor | PGSC0003DMG400019872,PGSC0003DMG400003115,PGSC0003DMG400003664,PGSC0003DMG400016477 |
| 0.0401 | GO:0033743 | peptide-methionine (R)-S-oxide reductase activity | PGSC0003DMG400003115,PGSC0003DMG400003664 |

**Supplemental table 11:** List of signaling related DEGs in Waneta and Atlantic after Lso treatment. (mapman analysis)

| **BinCode** | **BinName** | **id** | **description** | **fold change AT** | **fold change Waneta** |
| --- | --- | --- | --- | --- | --- |
| 30.1 | signalling.in sugar and nutrient physiology | pgsc0003dmp400034867|pacid:24398021 | very weakly similar to (83.2) AT3G25070 | Symbols: RIN4 | RIN4 (RPM1 INTERACTING PROTEIN 4); protein binding | chr3:9132458-9133747 FORWARDvery weakly similar to (84.0) loc_os03g63140 12003.m35534 protein nitrate-induced NOI protein, expressed no original description | - | -2.55 |
| 30.1 | signalling.in sugar and nutrient physiology | pgsc0003dmp400036229|pacid:24396956 | weakly similar to ( 103) AT5G52390 | Symbols: | photoassimilate-responsive protein, putative | chr5:21264281-21265173 REVERSEPAR1 no original description | 3.52 | - |
| 30.1 | signalling.in sugar and nutrient physiology | pgsc0003dmp400039805|pacid:24424570 | moderately similar to ( 450) AT2G29100 | Symbols: ATGLR2.9, GLR2.9 | ATGLR2.9; intracellular ligand-gated ion channel | chr2:12501092-12504912 REVERSEmoderately similar to ( 320) GLR31_ORYSA Glutamate receptor 3.1 precursor (Ligand-gated ion channel 3.1) - Oryza sativa (Rice)moderately similar to ( 444) loc_os09g25960 12009.m05748 protein glutamate receptor 2.7 precursor, putativeLig_chan ANF_receptor PBPe LivK no original description | - | -3.32 |
| 30.1 | signalling.in sugar and nutrient physiology | pgsc0003dmp400048580|pacid:24400216 | very weakly similar to (92.0) AT3G25070 | Symbols: RIN4 | RIN4 (RPM1 INTERACTING PROTEIN 4); protein binding | chr3:9132458-9133747 FORWARDvery weakly similar to (89.7) loc_os03g63140 12003.m35534 protein nitrate-induced NOI protein, expressed no original description | - | -2.71 |
| 30.1 | signalling.in sugar and nutrient physiology | pgsc0003dmp400053660|pacid:24382989 | moderately similar to ( 419) AT4G08950 | Symbols: EXO | EXO (EXORDIUM) | chr4:5740378-5741322 FORWARDmoderately similar to ( 368) loc_os02g52040 12002.m10235 protein phi-1-like phosphate-induced protein, putative, expressedPhi_1 no original description | - | -3.58 |
| 30.1 | signalling.in sugar and nutrient physiology | pgsc0003dmp400053661|pacid:24385526 | moderately similar to ( 408) AT4G08950 | Symbols: EXO | EXO (EXORDIUM) | chr4:5740378-5741322 FORWARDmoderately similar to ( 362) loc_os02g52040 12002.m10235 protein phi-1-like phosphate-induced protein, putative, expressedPhi_1 no original description | - | -9.11 |
| 30.1 | signalling.in sugar and nutrient physiology | pgsc0003dmp400053698|pacid:24384851 | moderately similar to ( 404) AT4G08950 | Symbols: EXO | EXO (EXORDIUM) | chr4:5740378-5741322 FORWARDmoderately similar to ( 360) loc_os02g52040 12002.m10235 protein phi-1-like phosphate-induced protein, putative, expressedPhi_1 no original description | - | -11.45 |
| 30.1 | signalling.in sugar and nutrient physiology | pgsc0003dmp400053699|pacid:24386199 | moderately similar to ( 413) AT4G08950 | Symbols: EXO | EXO (EXORDIUM) | chr4:5740378-5741322 FORWARDmoderately similar to ( 370) loc_os02g52040 12002.m10235 protein phi-1-like phosphate-induced protein, putative, expressedPhi_1 no original description | - | -7.79 |
| 30.1 | signalling.in sugar and nutrient physiology | pgsc0003dmp400053700|pacid:24382135 | moderately similar to ( 421) AT4G08950 | Symbols: EXO | EXO (EXORDIUM) | chr4:5740378-5741322 FORWARDmoderately similar to ( 363) loc_os02g52040 12002.m10235 protein phi-1-like phosphate-induced protein, putative, expressedPhi_1 no original description | - | -5.45 |
| 30.2.11 | signalling.receptor kinases.leucine rich repeat XI | pgsc0003dmp400002269|pacid:24408914 | moderately similar to ( 493) AT3G20820 | Symbols: | leucine-rich repeat family protein | chr3:7280930-7282027 FORWARDweakly similar to ( 130) PGIP3_PHAVU Polygalacturonase inhibitor 3 precursor (Polygalacturonase-inhibiting protein) (PGIP-2) (PGIP-3) - Phaseolus vulgaris (Kidney bean) (French bean)moderately similar to ( 404) loc_os08g39550 12008.m07936 protein polygalacturonase inhibitor 2 precursor, putative, expressed no original description | -10.37 | 1.97 |
| 30.2.11 | signalling.receptor kinases.leucine rich repeat XI | pgsc0003dmp400005241|pacid:24380497 | moderately similar to ( 314) AT1G71400 | Symbols: AtRLP12 | AtRLP12 (Receptor Like Protein 12); protein binding | chr1:26909905-26912448 FORWARDmoderately similar to ( 211) RPK1_IPONI Receptor-like protein kinase precursor (EC 2.7.11.1) - Ipomoea nil (Japanese morning glory) (Pharbitis nil)moderately similar to ( 315) loc_os12g10870 12012.m05069 protein verticillium wilt disease resistance protein, putative no original description | 4.71 | -2.77 |
| 30.2.11 | signalling.receptor kinases.leucine rich repeat XI | pgsc0003dmp400006322|pacid:24425809 | moderately similar to ( 404) AT5G66330 | Symbols: | leucine-rich repeat family protein | chr5:26500531-26501787 REVERSEvery weakly similar to (93.2) RPK1_IPONI Receptor-like protein kinase precursor (EC 2.7.11.1) - Ipomoea nil (Japanese morning glory) (Pharbitis nil)weakly similar to ( 198) loc_os02g43250 12002.m09360 protein receptor-like protein kinase precursor, putative, expressed no original description | -4.76 | - |
| 30.2.11 | signalling.receptor kinases.leucine rich repeat XI | pgsc0003dmp400007415|pacid:24385416 | nearly identical (1164) AT1G08590 | Symbols: | CLAVATA1 receptor kinase (CLV1) | chr1:2718859-2721948 FORWARDmoderately similar to ( 255) RPK1_IPONI Receptor-like protein kinase precursor (EC 2.7.11.1) - Ipomoea nil (Japanese morning glory) (Pharbitis nil)nearly identical (1053) loc_os02g02140 12002.m05564 protein receptor protein kinase CLAVATA1 precursor, putative, expressedS_TKc S_TKc Pkinase PTKc TyrKc Pkinase_Tyr PTKc_Ack_like PTKc_EphR PTKc_Csk_like PTKc_Src_like PTKc_Ror PTKc_Jak_rpt2 PTKc_Abl PTKc_Srm_Brk PTKc_EphR_A PTKc_EphR_A2 PTKc_EphR_B SPS1 PTKc_EGFR_like PTKc_Tec_like PTKc_Frk_like PTKc_Trk PTKc_Syk_like PTKc_Lck_Blk PTK_CCK4 PTKc_Lyn PTKc_Chk PTKc_InsR_like PTKc_DDR PTKc_Tec_Rlk PTKc_Musk PTKc_Src PTKc_Fes_like PTKc_Hck PTKc_Yes PTKc_Itk PTKc_Btk_Bmx PTKc_DDR1 PTKc_FGFR PTKc_Fyn_Yrk PTKc_Csk PTKc_HER4 PTKc_Met_Ron PTKc_FGFR3 PTKc_FGFR1 no original description | -6.41 | - |
| 30.2.11 | signalling.receptor kinases.leucine rich repeat XI | pgsc0003dmp400007624|pacid:24379299 | moderately similar to ( 484) AT1G45616 | Symbols: AtRLP6 | AtRLP6 (Receptor Like Protein 6); protein binding | chr1:17183550-17186534 REVERSEweakly similar to ( 180) RPK1_IPONI Receptor-like protein kinase precursor (EC 2.7.11.1) - Ipomoea nil (Japanese morning glory) (Pharbitis nil)moderately similar to ( 410) loc_os01g04070 12001.m07042 protein verticillium wilt disease resistance protein, putative, expressed no original description | - | -8.75 |
| 30.2.11 | signalling.receptor kinases.leucine rich repeat XI | pgsc0003dmp400008858|pacid:24380635 | highly similar to ( 836) AT1G63430 | Symbols: | leucine-rich repeat transmembrane protein kinase, putative | chr1:23522896-23526451 FORWARDweakly similar to ( 142) PSKR_DAUCA Phytosulfokine receptor precursor (EC 2.7.11.1) (Phytosulfokine LRR receptor kinase) - Daucus carota (Carrot)highly similar to ( 665) loc_os04g58700 12004.m10750 protein ATP binding protein, putative, expressedPTKc Pkinase_Tyr TyrKc S_TKc Pkinase no original description | -8.51 | - |
| 30.2.11 | signalling.receptor kinases.leucine rich repeat XI | pgsc0003dmp400009759|pacid:24379851 | moderately similar to ( 310) AT3G05660 | Symbols: AtRLP33 | AtRLP33 (Receptor Like Protein 33); kinase/ protein binding | chr3:1649258-1652001 REVERSEweakly similar to ( 148) PSKR_DAUCA Phytosulfokine receptor precursor (EC 2.7.11.1) (Phytosulfokine LRR receptor kinase) - Daucus carota (Carrot)moderately similar to ( 330) loc_os01g04070 12001.m07042 protein verticillium wilt disease resistance protein, putative, expressed no original description | - | -2.32 |
| 30.2.11 | signalling.receptor kinases.leucine rich repeat XI | pgsc0003dmp400011088|pacid:24400290 | moderately similar to ( 264) AT1G47890 | Symbols: AtRLP7 | AtRLP7 (Receptor Like Protein 7); kinase/ protein binding | chr1:17643976-17647035 FORWARDweakly similar to ( 138) PSKR_DAUCA Phytosulfokine receptor precursor (EC 2.7.11.1) (Phytosulfokine LRR receptor kinase) - Daucus carota (Carrot)moderately similar to ( 332) loc_os01g04070 12001.m07042 protein verticillium wilt disease resistance protein, putative, expressed no original description | - | -5.2 |
| 30.2.11 | signalling.receptor kinases.leucine rich repeat XI | pgsc0003dmp400011110|pacid:24399713 | moderately similar to ( 419) AT1G47890 | Symbols: AtRLP7 | AtRLP7 (Receptor Like Protein 7); kinase/ protein binding | chr1:17643976-17647035 FORWARDweakly similar to ( 198) RPK1_IPONI Receptor-like protein kinase precursor (EC 2.7.11.1) - Ipomoea nil (Japanese morning glory) (Pharbitis nil)highly similar to ( 565) loc_os04g40440 12004.m09026 protein expressed protein no original description | - | -5.81 |
| 30.2.11 | signalling.receptor kinases.leucine rich repeat XI | pgsc0003dmp400012189|pacid:24420308 | moderately similar to ( 415) AT3G20820 | Symbols: | leucine-rich repeat family protein | chr3:7280930-7282027 FORWARDweakly similar to ( 137) PGIP1_PHAVU Polygalacturonase inhibitor 1 precursor (Polygalacturonase-inhibiting protein) (PGIP-1) - Phaseolus vulgaris (Kidney bean) (French bean)moderately similar to ( 382) loc_os08g39550 12008.m07936 protein polygalacturonase inhibitor 2 precursor, putative, expressed no original description | -26.9 | - |
| 30.2.11 | signalling.receptor kinases.leucine rich repeat XI | pgsc0003dmp400013510|pacid:24390287 | nearly identical (1012) AT5G25930 | Symbols: | leucine-rich repeat family protein / protein kinase family protein | chr5:9050880-9053978 FORWARDmoderately similar to ( 405) RPK1_IPONI Receptor-like protein kinase precursor (EC 2.7.11.1) - Ipomoea nil (Japanese morning glory) (Pharbitis nil)highly similar to ( 828) loc_os02g12910 12002.m06538 protein receptor-like protein kinase 5 precursor, putative, expressedS_TKc S_TKc Pkinase_Tyr Pkinase TyrKc PTKc PTKc_EphR PTKc_Jak_rpt2 PTKc_Srm_Brk PTKc_Frk_like SPS1 PTKc_Csk_like PTKc_Trk PTKc_Src_like PTKc_InsR_like PTKc_Abl PTKc_Ror PTKc_Musk PTKc_Chk PTKc_EphR_A2 PTKc_Fes_like PTKc_c-ros PTKc_Src PTKc_Fyn_Yrk PTKc_EGFR_like PTKc_Jak2_Jak3_rpt2 PTKc_EphR_B PTKc_Lck_Blk PTKc_Syk_like PTKc_Yes PTKc_DDR PTKc_EphR_A PTKc_Ack_like PTKc_Tec_like PTKc_Csk PTKc_Met_Ron PTKc_TrkC PTKc_Lyn PTKc_DDR_like PTKc_Itk PTKc_Hck no original description | - | -2.9 |
| 30.2.11 | signalling.receptor kinases.leucine rich repeat XI | pgsc0003dmp400013958|pacid:24379022 | moderately similar to ( 259) AT2G33050 | Symbols: AtRLP26 | AtRLP26 (Receptor Like Protein 26); kinase/ protein binding | chr2:14021870-14024272 FORWARDweakly similar to ( 131) PSKR_DAUCA Phytosulfokine receptor precursor (EC 2.7.11.1) (Phytosulfokine LRR receptor kinase) - Daucus carota (Carrot)moderately similar to ( 254) loc_os01g04070 12001.m07042 protein verticillium wilt disease resistance protein, putative, expressed no original description | - | -3.16 |
| 30.2.11 | signalling.receptor kinases.leucine rich repeat XI | pgsc0003dmp400014331|pacid:24410601 | nearly identical (1106) AT2G41820 | Symbols: | leucine-rich repeat transmembrane protein kinase, putative | chr2:17447170-17449914 FORWARDmoderately similar to ( 344) RPK1_IPONI Receptor-like protein kinase precursor (EC 2.7.11.1) - Ipomoea nil (Japanese morning glory) (Pharbitis nil)highly similar to ( 892) loc_os07g10630 12007.m05518 protein ATP binding protein, putative, expressedPTKc S_TKc Pkinase Pkinase_Tyr S_TKc TyrKc no original description | - | 2.14 |
| 30.2.11 | signalling.receptor kinases.leucine rich repeat XI | pgsc0003dmp400016187|pacid:24405644 | nearly identical (1155) AT1G72300 | Symbols: | leucine-rich repeat transmembrane protein kinase, putative | chr1:27217679-27220966 REVERSEhighly similar to ( 777) PSKR_DAUCA Phytosulfokine receptor precursor (EC 2.7.11.1) (Phytosulfokine LRR receptor kinase) - Daucus carota (Carrot)highly similar to ( 896) loc_os06g47650 12006.m09291 protein phytosulfokine receptor precursor, putative, expressedPkinase_Tyr S_TKc Pkinase S_TKc TyrKc PTKc PTKc_Csk_like PTKc_Trk PTKc_Jak_rpt2 PTKc_Src_like PTKc_EphR SPS1 PTKc_Frk_like PTKc_TrkA PTKc_Itk PTKc_EphR_A2 PTKc_Lck_Blk PTKc_TrkB PTKc_Musk PTKc_RET PTKc_Srm_Brk PTKc_Hck PTKc_Tec_like PTKc_EGFR_like PTKc_Syk_like PTKc_Met_Ron PTKc_Ror PTKc_TrkC PTKc_Lyn PTKc_DDR PTKc_Jak2_Jak3_rpt2 PTKc_Tie2 PTKc_Abl PTKc_Src PTKc_InsR_like PTKc_EphR_B PTKc_FGFR PTKc_Fes_like PTKc_Ack_like PTKc_Axl_like PTKc_Csk PTKc_c-ros PTKc_Tec_Rlk PTKc_Tyk2_rpt2 PTKc_ALK_LTK PTKc_Chk PTKc_EphR_A PTKc_Fyn_Yrk PTKc_Btk_Bmx PTKc_Yes no original description | - | -2.73 |
| 30.2.11 | signalling.receptor kinases.leucine rich repeat XI | pgsc0003dmp400016299|pacid:24408716 | moderately similar to ( 399) AT1G34420 | Symbols: | leucine-rich repeat family protein / protein kinase family protein | chr1:12584587-12587570 FORWARDweakly similar to ( 185) RPK1_IPONI Receptor-like protein kinase precursor (EC 2.7.11.1) - Ipomoea nil (Japanese morning glory) (Pharbitis nil)highly similar to ( 619) loc_os12g08180 12012.m04805 protein receptor-like protein kinase precursor, putative, expressedS_TKc S_TKc Pkinase Pkinase_Tyr no original description | - | -2.62 |
| 30.2.11 | signalling.receptor kinases.leucine rich repeat XI | pgsc0003dmp400018288|pacid:24425831 | highly similar to ( 545) AT3G47570 | Symbols: | leucine-rich repeat transmembrane protein kinase, putative | chr3:17527611-17530748 FORWARDmoderately similar to ( 474) RPK1_IPONI Receptor-like protein kinase precursor (EC 2.7.11.1) - Ipomoea nil (Japanese morning glory) (Pharbitis nil)highly similar to ( 707) loc_os11g46980 12011.m08478 protein receptor-like protein kinase precursor, putativeTyrKc S_TKc Pkinase_Tyr S_TKc Pkinase PTKc PTKc_Srm_Brk PTKc_Csk_like PTKc_Jak_rpt2 PTKc_Fes_like PTKc_Src_like PTKc_Trk PTKc_EphR PTKc_Tec_like PTKc_TrkA PTKc_TrkC PTKc_Fer PTKc_EphR_A2 PTKc_Frk_like PTKc_Itk PTKc_Syk_like SPS1 PTKc_Axl_like PTKc_Ror PTKc_Src PTKc_TrkB PTKc_Lck_Blk PTKc_Btk_Bmx PTKc_Fyn_Yrk PTK_CCK4 PTKc_Fes PTKc_c-ros PTKc_Axl PTKc_InsR_like PTKc_Chk PTKc_Jak2_Jak3_rpt2 PTKc_Tyro3 PTKc_DDR PTKc_Csk PTKc_Abl no original description | 3.37 | - |
| 30.2.11 | signalling.receptor kinases.leucine rich repeat XI | pgsc0003dmp400023342|pacid:24399746 | highly similar to ( 957) AT3G51740 | Symbols: IMK2 | IMK2 (INFLORESCENCE MERISTEM RECEPTOR-LIKE KINASE 2); ATP binding / kinase/ protein kinase/ protein serine/threonine kinase | chr3:19189248-19191842 FORWARDmoderately similar to ( 295) PSKR_DAUCA Phytosulfokine receptor precursor (EC 2.7.11.1) (Phytosulfokine LRR receptor kinase) - Daucus carota (Carrot)highly similar to ( 809) loc_os03g18630 12003.m07284 protein receptor-like kinase RHG1, putative, expressedS_TKc Pkinase S_TKc PTKc TyrKc Pkinase_Tyr PTKc_Jak_rpt2 SPS1 PTKc_Csk_like PTKc_Src_like PTKc_Frk_like PTKc_Met_Ron PTKc_Abl PTKc_Fes_like PTKc_Csk PTKc_Srm_Brk PTKc_EGFR_like PTKc_EphR PTKc_Itk PTKc_Ror PTKc_Chk PTKc_Jak2_Jak3_rpt2 PTKc_TrkB PTKc_Syk_like PTKc_Lck_Blk PTKc_Trk PTKc_Musk PTKc_Tec_like PTKc_Tyro3 PTKc_Src no original description | -9.73 | - |
| 30.2.11 | signalling.receptor kinases.leucine rich repeat XI | pgsc0003dmp400024515|pacid:24380095 | moderately similar to ( 388) AT5G25910 | Symbols: AtRLP52 | AtRLP52 (Receptor Like Protein 52); kinase/ protein binding | chr5:9038860-9041377 FORWARDmoderately similar to ( 270) RPK1_IPONI Receptor-like protein kinase precursor (EC 2.7.11.1) - Ipomoea nil (Japanese morning glory) (Pharbitis nil)moderately similar to ( 444) loc_os10g33040 12010.m06140 protein receptor-like protein kinase precursor, putative, expressed no original description | - | -12.3 |
| 30.2.11 | signalling.receptor kinases.leucine rich repeat XI | pgsc0003dmp400029532|pacid:24388759 | moderately similar to ( 424) AT2G19780 | Symbols: | leucine-rich repeat family protein / extensin family protein | chr2:8522831-8524039 REVERSEweakly similar to ( 114) RPK1_IPONI Receptor-like protein kinase precursor (EC 2.7.11.1) - Ipomoea nil (Japanese morning glory) (Pharbitis nil)moderately similar to ( 365) loc_os05g09640 12005.m05477 protein BRASSINOSTEROID INSENSITIVE 1 precursor, putative, expressed no original description | -6.26 | - |
| 30.2.11 | signalling.receptor kinases.leucine rich repeat XI | pgsc0003dmp400031336|pacid:24389142 | moderately similar to ( 278) AT1G45616 | Symbols: AtRLP6 | AtRLP6 (Receptor Like Protein 6); protein binding | chr1:17183550-17186534 REVERSEweakly similar to ( 132) PSKR_DAUCA Phytosulfokine receptor precursor (EC 2.7.11.1) (Phytosulfokine LRR receptor kinase) - Daucus carota (Carrot)moderately similar to ( 263) loc_os01g04070 12001.m07042 protein verticillium wilt disease resistance protein, putative, expressed no original description | - | -5.17 |
| 30.2.11 | signalling.receptor kinases.leucine rich repeat XI | pgsc0003dmp400032677|pacid:24414508 | highly similar to ( 504) AT3G47570 | Symbols: | leucine-rich repeat transmembrane protein kinase, putative | chr3:17527611-17530748 FORWARDmoderately similar to ( 464) RPK1_IPONI Receptor-like protein kinase precursor (EC 2.7.11.1) - Ipomoea nil (Japanese morning glory) (Pharbitis nil)highly similar to ( 690) loc_os11g46980 12011.m08478 protein receptor-like protein kinase precursor, putativePkinase S_TKc TyrKc S_TKc Pkinase_Tyr PTKc PTKc_Srm_Brk PTKc_Jak_rpt2 PTKc_Csk_like PTKc_Src_like PTKc_Trk PTKc_Frk_like PTKc_EphR PTKc_EphR_A2 PTKc_EphR_A PTKc_Abl PTKc_Syk_like PTKc_Fes_like PTKc_Fyn_Yrk PTKc_Chk PTKc_EphR_B PTKc_Csk PTKc_Src PTKc_Lck_Blk PTKc_InsR_like PTKc_Tec_like PTKc_Jak2_Jak3_rpt2 PTKc_c-ros PTKc_Lyn SPS1 PTKc_Yes PTKc_Fer PTKc_Tyk2_rpt2 PTKc_Itk PTKc_EGFR_like PTKc_Btk_Bmx PTKc_Zap-70 PTKc_Hck PTKc_FGFR PTKc_Ror PTK_CCK4 PTKc_TrkA PTKc_Tie1 PTKc_ALK_LTK PTKc_Axl_like PTKc_Met_Ron PTKc_FAK no original description | - | -2.78 |
| 30.2.11 | signalling.receptor kinases.leucine rich repeat XI | pgsc0003dmp400037295|pacid:24384804 | highly similar to ( 539) AT4G08850 | Symbols: | kinase | chr4:5637467-5640496 REVERSEmoderately similar to ( 209) RPK1_IPONI Receptor-like protein kinase precursor (EC 2.7.11.1) - Ipomoea nil (Japanese morning glory) (Pharbitis nil)moderately similar to ( 472) loc_os02g34790 12002.m08568 protein receptor-like protein kinase 5 precursor, putative, expressedS_TKc Pkinase S_TKc Pkinase_Tyr PTKc TyrKc PTKc_Srm_Brk PTKc_Jak_rpt2 PTKc_EphR PTKc_EphR_A2 SPS1 PTKc_Syk_like no original description | - | -15.33 |
| 30.2.11 | signalling.receptor kinases.leucine rich repeat XI | pgsc0003dmp400037296|pacid:24384803 | highly similar to ( 583) AT4G08850 | Symbols: | kinase | chr4:5637467-5640496 REVERSEmoderately similar to ( 211) RPK1_IPONI Receptor-like protein kinase precursor (EC 2.7.11.1) - Ipomoea nil (Japanese morning glory) (Pharbitis nil)highly similar to ( 511) loc_os02g34790 12002.m08568 protein receptor-like protein kinase 5 precursor, putative, expressedS_TKc S_TKc Pkinase Pkinase_Tyr PTKc TyrKc PTKc_Srm_Brk PTKc_EphR PTKc_Src_like PTKc_EphR_A2 PTKc_Jak_rpt2 PTKc_EphR_B PTKc_Syk_like SPS1 PTKc_EGFR_like PTKc_Fes_like PTKc_Tec_like PTKc_Csk_like PTKc_EphR_A PTKc_Trk PTKc_Frk_like PTKc_Abl PTKc_RET PTKc_Ror PTKc_Fes no original description | - | -9.24 |
| 30.2.11 | signalling.receptor kinases.leucine rich repeat XI | pgsc0003dmp400038574|pacid:24416020 | nearly identical (1285) AT4G26540 | Symbols: | kinase | chr4:13394673-13398028 REVERSEmoderately similar to ( 473) RPK1_IPONI Receptor-like protein kinase precursor (EC 2.7.11.1) - Ipomoea nil (Japanese morning glory) (Pharbitis nil)nearly identical (1152) loc_os08g38560 12008.m07840 protein receptor-like protein kinase precursor, putative, expressedPkinase_Tyr S_TKc Pkinase S_TKc PTKc TyrKc PTKc_Jak_rpt2 PTKc_Srm_Brk PTKc_Src_like PTKc_Frk_like PTKc_Tec_like PTKc_Ack_like PTKc_Csk_like PTKc_EphR PTKc_EphR_A2 SPS1 PTKc_Src PTKc_Itk PTKc_Chk PTKc_Lyn no original description | - | 2.6 |
| 30.2.11 | signalling.receptor kinases.leucine rich repeat XI | pgsc0003dmp400042261|pacid:24379567 | moderately similar to ( 332) AT4G13920 | Symbols: AtRLP50 | AtRLP50 (Receptor Like Protein 50); kinase/ protein binding | chr4:8043861-8046536 FORWARDmoderately similar to ( 228) RPK1_IPONI Receptor-like protein kinase precursor (EC 2.7.11.1) - Ipomoea nil (Japanese morning glory) (Pharbitis nil)moderately similar to ( 325) loc_os12g11370 12012.m05117 protein verticillium wilt disease resistance protein, putative, expressed no original description | - | -4.51 |
| 30.2.11 | signalling.receptor kinases.leucine rich repeat XI | pgsc0003dmp400042691|pacid:24403991 | nearly identical (1225) AT1G74360 | Symbols: | leucine-rich repeat transmembrane protein kinase, putative | chr1:27954299-27957911 FORWARDmoderately similar to ( 222) RPK1_IPONI Receptor-like protein kinase precursor (EC 2.7.11.1) - Ipomoea nil (Japanese morning glory) (Pharbitis nil)highly similar to ( 647) loc_os07g40630 12007.m08303 protein BRASSINOSTEROID INSENSITIVE 1 precursor, putative, expressedS_TKc S_TKc Pkinase TyrKc Pkinase_Tyr PTKc PTKc_Csk_like PTKc_EGFR_like PTKc_Fes_like PTKc_Syk_like PTKc_Jak_rpt2 PTKc_Itk PTKc_EphR SPS1 PTKc_Tec_like PTKc_Frk_like PTKc_Met_Ron PTKc_Src_like PTKc_Srm_Brk PTKc_Btk_Bmx PTKc_Trk PTKc_FGFR PTKc_HER4 PTKc_Abl PTKc_Tec_Rlk PTKc_Fer PTKc_Jak2_Jak3_rpt2 PTKc_InsR_like PTKc_Lck_Blk PTKc_EphR_A PTKc_Ack_like PTKc_HER2 PTKc_EGFR PTKc_c-ros PTKc_Ror PTKc_EphR_B PTKc_EphR_A2 PTKc_Src PTKc_Musk PTKc_Axl_like PTKc_Tie1 PTKc_Csk PTK_HER3 PTKc_Chk PTKc_TrkB PTKc_ALK_LTK PTKc_Tyk2_rpt2 PTKc_Tie2 PTKc_PDGFR COG4886 PTKc_Lyn PTKc_TrkC PTKc_Fes PTKc_Yes PTKc_Tie no original description | - | -3.22 |
| 30.2.11 | signalling.receptor kinases.leucine rich repeat XI | pgsc0003dmp400044870|pacid:24421369 | moderately similar to ( 371) AT2G15320 | Symbols: | leucine-rich repeat family protein | chr2:6666527-6667675 REVERSEvery weakly similar to (84.0) RPK1_IPONI Receptor-like protein kinase precursor (EC 2.7.11.1) - Ipomoea nil (Japanese morning glory) (Pharbitis nil)weakly similar to ( 161) loc_os02g43250 12002.m09360 protein receptor-like protein kinase precursor, putative, expressed no original description | - | 2.5 |
| 30.2.11 | signalling.receptor kinases.leucine rich repeat XI | pgsc0003dmp400047771|pacid:24415419 | moderately similar to ( 226) AT5G48380 | Symbols: | leucine-rich repeat family protein / protein kinase family protein | chr5:19604584-19606532 REVERSEweakly similar to ( 145) PSKR_DAUCA Phytosulfokine receptor precursor (EC 2.7.11.1) (Phytosulfokine LRR receptor kinase) - Daucus carota (Carrot)moderately similar to ( 238) loc_os11g14420 12011.m05578 protein brassinosteroid LRR receptor kinase precursor, putative, expressedPkinase_Tyr PTKc TyrKc no original description | - | -4.69 |
| 30.2.11 | signalling.receptor kinases.leucine rich repeat XI | pgsc0003dmp400047774|pacid:24415721 | weakly similar to ( 136) AT5G48380 | Symbols: | leucine-rich repeat family protein / protein kinase family protein | chr5:19604584-19606532 REVERSEweakly similar to ( 145) loc_os05g34270 12005.m07658 protein BRASSINOSTEROID INSENSITIVE 1-associated receptor kinase 1 precursor, putative, expressed no original description | - | -3.53 |
| 30.2.11 | signalling.receptor kinases.leucine rich repeat XI | pgsc0003dmp400049780|pacid:24422828 | moderately similar to ( 324) AT4G13920 | Symbols: AtRLP50 | AtRLP50 (Receptor Like Protein 50); kinase/ protein binding | chr4:8043861-8046536 FORWARDweakly similar to ( 169) RPK1_IPONI Receptor-like protein kinase precursor (EC 2.7.11.1) - Ipomoea nil (Japanese morning glory) (Pharbitis nil)moderately similar to ( 401) loc_os12g12010 12012.m05176 protein verticillium wilt disease resistance protein precursor, putative no original description | - | -6.98 |
| 30.2.11 | signalling.receptor kinases.leucine rich repeat XI | pgsc0003dmp400052449|pacid:24390176 | moderately similar to ( 427) AT5G25930 | Symbols: | leucine-rich repeat family protein / protein kinase family protein | chr5:9050880-9053978 FORWARDmoderately similar to ( 250) PSKR_DAUCA Phytosulfokine receptor precursor (EC 2.7.11.1) (Phytosulfokine LRR receptor kinase) - Daucus carota (Carrot)moderately similar to ( 383) loc_os06g36270 12006.m091721 protein receptor-like protein kinase 5 precursor, putative, expressedS_TKc S_TKc TyrKc Pkinase_Tyr Pkinase PTKc PTKc_Jak_rpt2 PTKc_Csk_like PTKc_Src_like PTKc_Abl PTKc_EphR_A2 PTKc_Jak2_Jak3_rpt2 PTKc_InsR_like PTKc_EphR PTKc_Frk_like SPS1 PTKc_EphR_B PTKc_Srm_Brk PTKc_Trk PTKc_Src PTKc_EGFR_like PTKc_DDR PTKc_Ror PTKc_EphR_A PTKc_Fyn_Yrk PTKc_Tyk2_rpt2 PTKc_Fes_like PTKc_Hck PTKc_Lck_Blk PTKc_Ack_like PTKc_Csk PTKc_Musk PTKc_TrkB PTKc_Chk PTKc_Jak1_rpt2 PTKc_Lyn PTKc_Yes no original description | - | -3.44 |
| 30.2.11 | signalling.receptor kinases.leucine rich repeat XI | pgsc0003dmp400053338|pacid:24428810 | nearly identical (1502) AT5G65700 | Symbols: BAM1 | BAM1 (BARELY ANY MERISTEM 1); ATP binding / kinase/ protein serine/threonine kinase | chr5:26281826-26284945 FORWARDhighly similar to ( 501) RPK1_IPONI Receptor-like protein kinase precursor (EC 2.7.11.1) - Ipomoea nil (Japanese morning glory) (Pharbitis nil)nearly identical (1379) loc_os03g56270 12003.m35431 protein receptor protein kinase CLAVATA1 precursor, putative, expressedPkinase_Tyr S_TKc TyrKc Pkinase S_TKc PTKc PTKc_Jak_rpt2 PTKc_Src_like PTKc_Srm_Brk PTKc_EphR PTKc_Frk_like PTKc_Csk_like PTKc_Tec_like PTKc_EGFR_like PTKc_Syk_like SPS1 PTKc_Itk PTKc_Tec_Rlk PTKc_Ack_like PTKc_Src PTKc_Btk_Bmx PTKc_Fes_like PTK_HER3 PTKc_Trk PTKc_Met_Ron PTKc_Lyn PTKc_InsR_like PTKc_HER4 PTKc_DDR PTKc_EphR_A2 PTKc_Lck_Blk PTKc_Jak1_rpt2 PTKc_Fyn_Yrk PTKc_Jak2_Jak3_rpt2 PTKc_Abl PTKc_Ror PTKc_Yes PTKc_Tyk2_rpt2 PTKc_TrkA PTKc_FAK PTKc_EphR_A PTKc_EGFR PTK_CCK4 PTKc_Hck PTKc_TrkC PTKc_EphR_B PTKc_HER2 PTKc_Csk PTKc_EphR_A10 PTKc_FGFR PTKc_Axl_like no original description | - | 1.7 |
| 30.2.11 | signalling.receptor kinases.leucine rich repeat XI | pgsc0003dmp400054963|pacid:24428168 | nearly identical (1282) AT1G28440 | Symbols: HSL1 | HSL1 (HAESA-Like 1); ATP binding / kinase/ protein serine/threonine kinase | chr1:9996914-10000171 FORWARDmoderately similar to ( 452) RPK1_IPONI Receptor-like protein kinase precursor (EC 2.7.11.1) - Ipomoea nil (Japanese morning glory) (Pharbitis nil)nearly identical (1056) loc_os01g13800 12001.m07986 protein receptor-like protein kinase 5 precursor, putative, expressedS_TKc Pkinase S_TKc TyrKc PTKc Pkinase_Tyr PTKc_Csk_like PTKc_Frk_like SPS1 PTKc_Src_like PTKc_Jak_rpt2 PTKc_Srm_Brk PTKc_Trk PTKc_Itk PTKc_Ack_like PTKc_Fes_like PTKc_EGFR_like PTKc_Tec_like PTKc_Src PTKc_Fyn_Yrk PTKc_EphR PTKc_Lck_Blk PTKc_Tyk2_rpt2 PTKc_Btk_Bmx PTKc_Lyn PTKc_Jak2_Jak3_rpt2 PTKc_Hck PTKc_Csk PTKc_Yes PTKc_Abl PTKc_Ror PTKc_Syk_like PTKc_Tec_Rlk PTKc_InsR_like PTKc_TrkA PTKc_Chk PTKc_EphR_A2 no original description | - | 2.21 |
| 30.2.11 | signalling.receptor kinases.leucine rich repeat XI | pgsc0003dmp400056119|pacid:24391126 | moderately similar to ( 261) AT3G05660 | Symbols: AtRLP33 | AtRLP33 (Receptor Like Protein 33); kinase/ protein binding | chr3:1649258-1652001 REVERSEweakly similar to ( 127) PSKR_DAUCA Phytosulfokine receptor precursor (EC 2.7.11.1) (Phytosulfokine LRR receptor kinase) - Daucus carota (Carrot)moderately similar to ( 241) loc_os01g04070 12001.m07042 protein verticillium wilt disease resistance protein, putative, expressed no original description | 3.12 | -3.3 |
| 30.2.11 | signalling.receptor kinases.leucine rich repeat XI | pgsc0003dmp400058878|pacid:24382441 | very weakly similar to (95.5) AT3G47580 | Symbols: | leucine-rich repeat transmembrane protein kinase, putative | chr3:17532687-17535810 FORWARDvery weakly similar to (85.9) PSKR_DAUCA Phytosulfokine receptor precursor (EC 2.7.11.1) (Phytosulfokine LRR receptor kinase) - Daucus carota (Carrot)weakly similar to ( 127) loc_os11g36190 12011.m080025 protein receptor-like protein kinase 5 precursor, putative, expressed no original description | 3.48 | - |
| 30.2.16 | signalling.receptor kinases.Catharanthus roseus-like RLK1 | pgsc0003dmp400000405|pacid:24418527 | moderately similar to ( 332) AT4G00300 | Symbols: | fringe-related protein | chr4:126509-130126 FORWARDmoderately similar to ( 300) loc_os03g03290 12003.m05865 protein ATP binding protein, putative, expressed no original description | - | -1.75 |
| 30.2.17 | signalling.receptor kinases.DUF 26 | pgsc0003dmp400000304|pacid:24421887 | moderately similar to ( 449) AT2G45590 | Symbols: | protein kinase family protein | chr2:18786725-18788776 FORWARDweakly similar to ( 110) NORK_PEA Nodulation receptor kinase precursor (EC 2.7.11.1) - Pisum sativum (Garden pea)moderately similar to ( 381) loc_os03g14710 12003.m06909 protein ATP binding protein, putative, expressedS_TKc Pkinase Pkinase_Tyr S_TKc TyrKc PTKc PTKc_Jak_rpt2 no original description | - | 1.76 |
| 30.2.17 | signalling.receptor kinases.DUF 26 | pgsc0003dmp400007093|pacid:24407557 | moderately similar to ( 266) AT5G60900 | Symbols: RLK1 | RLK1 (RECEPTOR-LIKE PROTEIN KINASE 1); ATP binding / carbohydrate binding / kinase/ protein kinase/ protein serine/threonine kinase/ protein tyrosine kinase/ sugar binding | chr5:24498467-24501494 REVERSEweakly similar to ( 167) KPRO_MAIZE Putative receptor protein kinase ZmPK1 precursor (EC 2.7.11.1) - Zea mays (Maize)moderately similar to ( 382) loc_os03g61310 12003.m11014 protein receptor-like protein kinase, putative, expressedTyrKc Pkinase_Tyr S_TKc S_TKc Pkinase PTKc PTKc_Csk_like PTKc_Jak_rpt2 PTKc_Srm_Brk PTKc_Src_like SPS1 PTKc_EGFR_like PTKc_Itk PTKc_EphR PTKc_Fes_like PTKc_Tec_like PTKc_Met_Ron PTKc_Btk_Bmx PTKc_Abl PTKc_Trk PTKc_FGFR PTKc_Jak2_Jak3_rpt2 PTKc_EphR_A2 PTKc_InsR_like PTKc_Syk_like PTKc_Frk_like PTKc_c-ros no original description | - | -5.47 |
| 30.2.17 | signalling.receptor kinases.DUF 26 | pgsc0003dmp400007094|pacid:24407558 | weakly similar to ( 122) AT5G60900 | Symbols: RLK1 | RLK1 (RECEPTOR-LIKE PROTEIN KINASE 1); ATP binding / carbohydrate binding / kinase/ protein kinase/ protein serine/threonine kinase/ protein tyrosine kinase/ sugar binding | chr5:24498467-24501494 REVERSEweakly similar to ( 105) KPRO_MAIZE Putative receptor protein kinase ZmPK1 precursor (EC 2.7.11.1) - Zea mays (Maize)moderately similar to ( 214) loc_os08g13870 12008.m05512 protein ATP binding protein, putative no original description | - | -4.38 |
| 30.2.17 | signalling.receptor kinases.DUF 26 | pgsc0003dmp400007096|pacid:24407414 | moderately similar to ( 345) AT5G60900 | Symbols: RLK1 | RLK1 (RECEPTOR-LIKE PROTEIN KINASE 1); ATP binding / carbohydrate binding / kinase/ protein kinase/ protein serine/threonine kinase/ protein tyrosine kinase/ sugar binding | chr5:24498467-24501494 REVERSEmoderately similar to ( 256) KPRO_MAIZE Putative receptor protein kinase ZmPK1 precursor (EC 2.7.11.1) - Zea mays (Maize)moderately similar to ( 442) loc_os03g61310 12003.m11014 protein receptor-like protein kinase, putative, expressedS_TKc Pkinase S_TKc TyrKc Pkinase_Tyr PTKc PTKc_Csk_like PTKc_Src_like SPS1 PTKc_Itk PTKc_Jak_rpt2 PTKc_Srm_Brk PTKc_EphR PTKc_Frk_like PTKc_Tec_like PTKc_Btk_Bmx PTKc_Trk PTKc_Abl PTKc_Fes_like PTKc_Met_Ron PTKc_EphR_A2 PTKc_EphR_B PTKc_EGFR_like PTKc_Lck_Blk PTKc_EphR_A PTKc_InsR_like PTKc_TrkB PTKc_Ror PTKc_ALK_LTK PTKc_Tec_Rlk PTKc_FGFR PTKc_Jak2_Jak3_rpt2 PTKc_Tie2 PTKc_Chk PTKc_Syk_like PTKc_TrkC PTKc_Fes PTKc_TrkA PTKc_Fyn_Yrk PTK_CCK4 PTKc_c-ros PTKc_PDGFR PTKc_InsR PTKc_Lyn PTKc_DDR_like PTKc_Fer PTKc_Musk PTKc_Ack_like PTKc_Yes PTKc_DDR PTKc_Src PTKc_Tie1 PTKc_FGFR2 PTKc_Axl_like PTKc_Syk PTKc_Csk PTKc_Ror1 no original description | 2.92 | -5.17 |
| 30.2.17 | signalling.receptor kinases.DUF 26 | pgsc0003dmp400007130|pacid:24407530 | moderately similar to ( 423) AT5G60900 | Symbols: RLK1 | RLK1 (RECEPTOR-LIKE PROTEIN KINASE 1); ATP binding / carbohydrate binding / kinase/ protein kinase/ protein serine/threonine kinase/ protein tyrosine kinase/ sugar binding | chr5:24498467-24501494 REVERSEmoderately similar to ( 289) KPRO_MAIZE Putative receptor protein kinase ZmPK1 precursor (EC 2.7.11.1) - Zea mays (Maize)highly similar to ( 535) loc_os08g13870 12008.m05512 protein ATP binding protein, putativeS_TKc Pkinase S_TKc Pkinase_Tyr TyrKc PTKc PTKc_Csk_like PTKc_Src_like SPS1 PTKc_Jak_rpt2 PTKc_Srm_Brk PTKc_Itk PTKc_Frk_like PTKc_EphR PTKc_InsR_like PTKc_Trk PTKc_Tec_like PTKc_Met_Ron PTKc_EGFR_like PTKc_Btk_Bmx PTKc_EphR_A2 PTKc_Abl PTKc_Lck_Blk PTKc_Fes_like PTKc_TrkB PTKc_Jak2_Jak3_rpt2 PTKc_EphR_A PTKc_FGFR PTKc_TrkC PTKc_EphR_B PTKc_ALK_LTK PTKc_InsR PTKc_Chk PTKc_Ror PTKc_TrkA PTKc_PDGFR PTKc_Tec_Rlk PTKc_Ack_like PTKc_Syk_like PTKc_Lyn PTKc_Tie2 PTKc_IGF-1R PTKc_Fyn_Yrk PTKc_Fes PTKc_c-ros PTK_CCK4 PTKc_Musk PTKc_FGFR3 PTKc_Tie1 PTKc_HER4 PTKc_Fer PTKc_Yes PTKc_Src PTKc_DDR PTKc_Tyk2_rpt2 PTKc_Axl PTKc_Hck PTKc_FGFR2 PTKc_DDR_like no original description | - | -3.46 |
| 30.2.17 | signalling.receptor kinases.DUF 26 | pgsc0003dmp400007809|pacid:24411697 | moderately similar to ( 399) AT5G10530 | Symbols: | lectin protein kinase, putative | chr5:3324978-3326933 REVERSEweakly similar to ( 179) PSKR_DAUCA Phytosulfokine receptor precursor (EC 2.7.11.1) (Phytosulfokine LRR receptor kinase) - Daucus carota (Carrot)moderately similar to ( 409) loc_os12g41530 12012.m07934 protein protein kinase, putativePkinase S_TKc S_TKc Pkinase_Tyr TyrKc PTKc PTKc_Jak_rpt2 PTKc_EphR PTKc_Itk SPS1 PTKc_Srm_Brk PTKc_Tec_like PTKc_EphR_B PTKc_EGFR_like PTKc_Src_like PTKc_Csk_like PTKc_Fes_like PTKc_Abl PTKc_FAK PTKc_Frk_like PTKc_Fer PTKc_Trk PTKc_Syk_like PTKc_EphR_A PTKc_Tec_Rlk PTKc_EphR_A2 PTKc_Jak2_Jak3_rpt2 PTKc_InsR_like PTKc_Ror PTKc_Ack_like PTKc_TrkB PTKc_DDR PTKc_Btk_Bmx PTKc_Fes PTKc_Fyn_Yrk PTKc_TrkC PTKc_Musk PTKc_Tyk2_rpt2 PTKc_Lck_Blk PTKc_HER4 PTKc_Src PTKc_TrkA PTKc_Met_Ron PTKc_c-ros PTKc_Jak1_rpt2 PTKc_Axl_like PTKc_ALK_LTK PTK_HER3 PTKc_Lyn PTKc_Zap-70 PTKc_Yes PTKc_Chk PTK_Jak_rpt1 PTKc_FGFR PTKc_Syk PTK_Jak2_Jak3_rpt1 PTKc_DDR1 PTKc_IGF-1R PTKc_Tie2 PTKc_PDGFR no original description | 4.6 | - |
| 30.2.17 | signalling.receptor kinases.DUF 26 | pgsc0003dmp400008766|pacid:24385252 | highly similar to ( 854) AT4G27290 | Symbols: | ATP binding / protein kinase/ protein serine/threonine kinase/ protein tyrosine kinase/ sugar binding | chr4:13666281-13669202 FORWARDmoderately similar to ( 347) SLSG6_BRAOL S-locus-specific glycoprotein S6 precursor (SLSG-6) - Brassica oleracea (Wild cabbage)highly similar to ( 704) loc_os07g36544 12007.m07905 protein serine/threonine-protein kinase receptor precursor, putative, expressedTyrKc Pkinase_Tyr PTKc S_locus_glycop Pkinase S_TKc S_TKc PTKc_Jak_rpt2 PTKc_Src_like PTKc_Srm_Brk PTKc_Csk_like PTKc_Trk PTKc_Syk_like PTKc_EGFR_like PTKc_c-ros PTKc_InsR_like PTKc_Ror B_lectin PTKc_ALK_LTK PTKc_Lck_Blk B_lectin PTKc_Frk_like PTKc_Itk B_lectin PTKc_EphR PTK_CCK4 PTKc_Ack_like PTKc_FGFR PTKc_Tec_like PTKc_Jak2_Jak3_rpt2 PTKc_Axl_like PTKc_Fes_like PTKc_Abl PTKc_Lyn PTKc_Ror2 SPS1 PTKc_IGF-1R PTKc_DDR PTKc_Src PTKc_TrkA PTKc_Fyn_Yrk PTKc_Ror1 PTKc_EphR_A2 PTKc_TrkC PTKc_TrkB PTKc_Hck PTKc_Tec_Rlk PTKc_Yes PTKc_Musk PTKc_Met_Ron PTKc_Btk_Bmx PTKc_RET PTKc_Tyk2_rpt2 PTKc_FGFR4 PTKc_InsR PTKc_HER4 PTKc_EGFR PAN_2 PTKc_Chk PTKc_EphR_A PTKc_DDR_like PTKc_Axl PTKc_Csk PTKc_PDGFR PTKc_EphR_B PTKc_Tyro3 PTKc_FGFR2 PTKc_Fer PTK_Ryk PTK_HER3 PTKc_Zap-70 PTKc_FGFR1 PTKc_DDR1 PTKc_FGFR3 PAN_AP_plant PTKc_DDR2 PTKc_HER2 PTKc_Tie2 PTKc_Syk PTKc_Fes no original description | - | -1.71 |
| 30.2.17 | signalling.receptor kinases.DUF 26 | pgsc0003dmp400010663|pacid:24386422 | moderately similar to ( 344) AT1G70250 | Symbols: | receptor serine/threonine kinase, putative | chr1:26452975-26456088 FORWARDweakly similar to ( 189) KPRO_MAIZE Putative receptor protein kinase ZmPK1 precursor (EC 2.7.11.1) - Zea mays (Maize)moderately similar to ( 366) loc_os04g09770 12004.m06230 protein Ser/Thr protein kinase, putativePkinase_Tyr TyrKc PTKc Pkinase S_TKc S_TKc PTKc_EGFR_like PTKc_Src_like PTKc_Jak_rpt2 PTKc_Csk_like PTKc_Syk_like PTKc_Srm_Brk PTKc_EphR PTKc_Frk_like PTKc_Tec_like PTKc_Lck_Blk PTKc_Itk PTKc_Abl PTKc_Axl_like PTKc_Trk PTKc_FGFR PTKc_Ack_like PTKc_Fyn_Yrk PTKc_HER4 PTKc_Fes_like PTKc_EGFR PTKc_Lyn SPS1 PTKc_Ror PTKc_Yes PTKc_Src PTKc_Chk PTKc_InsR_like PTKc_Tec_Rlk PTKc_Hck PTKc_c-ros PTKc_HER2 PTKc_Btk_Bmx PTKc_ALK_LTK PTKc_Jak2_Jak3_rpt2 PTKc_EphR_A2 PTKc_Axl PTKc_DDR PTKc_Tie2 PTKc_EphR_B PTKc_Tyro3 PTKc_Fer PTKc_PDGFR PTKc_FGFR2 PTKc_Met_Ron PTKc_FGFR3 PTK_HER3 PTKc_EphR_A PTKc_FGFR1 PTKc_Musk PTKc_Csk no original description | - | -3.45 |
| 30.2.17 | signalling.receptor kinases.DUF 26 | pgsc0003dmp400015985|pacid:24404401 | moderately similar to ( 317) AT1G66920 | Symbols: | serine/threonine protein kinase, putative | chr1:24965410-24967432 REVERSEweakly similar to ( 165) KPRO_MAIZE Putative receptor protein kinase ZmPK1 precursor (EC 2.7.11.1) - Zea mays (Maize)moderately similar to ( 383) loc_os01g02810 12001.m06921 protein receptor kinase LRK14, putativePkinase S_TKc S_TKc Pkinase_Tyr PTKc TyrKc PTKc_Src_like SPS1 PTKc_Srm_Brk PTKc_Jak_rpt2 PTKc_Frk_like PTKc_Csk_like PTKc_Tec_like PTKc_EphR PTKc_Abl PTKc_Itk PTKc_Ror PTKc_Trk PTKc_EGFR_like PTKc_Fes_like PTKc_Lck_Blk PTKc_Tec_Rlk PTKc_Btk_Bmx PTKc_Lyn PTKc_Syk_like PTKc_Chk PTK_HER3 PTKc_Fyn_Yrk PTKc_EphR_A2 PTKc_TrkA PTKc_InsR_like PTKc_ALK_LTK PTKc_Jak2_Jak3_rpt2 PTKc_Hck PTKc_FGFR PTKc_Ack_like PTKc_Tie2 PTKc_Src PTKc_Axl_like PTKc_Ror1 PTKc_EphR_B PTKc_Yes PTKc_TrkC PTKc_Tie1 no original description | - | -9.49 |
| 30.2.17 | signalling.receptor kinases.DUF 26 | pgsc0003dmp400016044|pacid:24405493 | moderately similar to ( 336) AT5G38280 | Symbols: PR5K | PR5K; kinase/ transmembrane receptor protein serine/threonine kinase | chr5:15293325-15295838 REVERSEweakly similar to ( 181) KPRO_MAIZE Putative receptor protein kinase ZmPK1 precursor (EC 2.7.11.1) - Zea mays (Maize)moderately similar to ( 367) loc_os01g02400 12001.m06882 protein Ser/Thr receptor-like kinase, putative, expressedPkinase_Tyr PTKc TyrKc Pkinase S_TKc S_TKc PTKc_Itk PTKc_Src_like PTKc_Tec_like PTKc_Csk_like PTKc_Srm_Brk PTKc_EphR PTKc_Jak_rpt2 SPS1 PTKc_Tec_Rlk PTKc_Abl PTKc_Fes_like PTKc_Frk_like PTKc_Btk_Bmx PTKc_Ror PTKc_EphR_B PTKc_FGFR PTKc_EGFR_like PTKc_Chk PTKc_Syk_like PTKc_Trk PTKc_Src PTKc_Lck_Blk PTKc_EphR_A PTKc_EphR_A2 PTKc_DDR_like PTKc_Musk PTKc_PDGFR PTKc_Axl_like PTKc_DDR PTKc_Fer PTKc_Lyn PTKc_InsR_like PTKc_Fyn_Yrk PTKc_Csk PTKc_Axl PTK_Ryk PTKc_EphR_A10 PTKc_DDR1 PTKc_Ror1 PTKc_Hck PTKc_Tie2 PTKc_Yes PTKc_Ack_like PTKc_RET PTKc_c-ros PTKc_Fes PTKc_TrkC PTKc_Aatyk PTKc_Jak2_Jak3_rpt2 PTKc_DDR2 PTKc_TrkB PTKc_FGFR1 PTKc_FGFR2 PTK_HER3 PTKc_FGFR3 PTKc_Tie1 PTKc_ALK_LTK PTKc_TrkA PTKc_FGFR4 no original description | - | -2.02 |
| 30.2.17 | signalling.receptor kinases.DUF 26 | pgsc0003dmp400018584|pacid:24418637 | weakly similar to ( 170) AT1G66920 | Symbols: | serine/threonine protein kinase, putative | chr1:24965410-24967432 REVERSEweakly similar to ( 120) KPRO_MAIZE Putative receptor protein kinase ZmPK1 precursor (EC 2.7.11.1) - Zea mays (Maize)weakly similar to ( 191) loc_os01g02560 12001.m06898 protein Ser/Thr receptor-like kinase, putative, expressedPkinase_Tyr TyrKc PTKc S_TKc Pkinase S_TKc no original description | - | -2.06 |
| 30.2.17 | signalling.receptor kinases.DUF 26 | pgsc0003dmp400019557|pacid:24411653 | highly similar to ( 739) AT3G55550 | Symbols: | lectin protein kinase, putative | chr3:20600019-20602073 REVERSEmoderately similar to ( 201) PSKR_DAUCA Phytosulfokine receptor precursor (EC 2.7.11.1) (Phytosulfokine LRR receptor kinase) - Daucus carota (Carrot)highly similar to ( 668) loc_os12g38610 12012.m07646 protein lectin-like receptor kinase 7, putative, expressedLectin_legB Pkinase PTKc Pkinase_Tyr TyrKc S_TKc S_TKc PTKc_Jak_rpt2 PTKc_Csk_like PTKc_Srm_Brk PTKc_Src_like PTKc_EphR PTKc_Frk_like PTKc_Fes_like PTKc_Tec_like PTKc_InsR_like PTKc_Abl PTKc_EphR_B PTKc_Syk_like PTKc_EGFR_like PTKc_Trk PTKc_Chk PTKc_Fer PTKc_EphR_A2 PTKc_Jak2_Jak3_rpt2 SPS1 PTKc_Itk PTKc_Ror PTKc_Fyn_Yrk PTKc_EphR_A PTKc_Csk PTKc_Src PTKc_Tyk2_rpt2 PTKc_ALK_LTK PTKc_FGFR PTKc_Lck_Blk PTKc_Tie2 PTKc_Tec_Rlk PTKc_Ack_like PTKc_DDR PTKc_Yes PTKc_Tie PTKc_Tie1 PTKc_Musk PTKc_Btk_Bmx PTKc_FAK PTKc_EphR_A10 PTKc_Fes PTKc_Lyn PTKc_Met_Ron PTKc_c-ros PTKc_Aatyk PTKc_RET PTKc_TrkB PTKc_TrkA PTKc_Hck PTKc_TrkC PTKc_PDGFR PTKc_Zap-70 PTK_HER3 PTKc_Axl_like PTK_CCK4 PTKc_DDR_like PTKc_DDR1 PTKc_IGF-1R no original description | - | -5.93 |
| 30.2.17 | signalling.receptor kinases.DUF 26 | pgsc0003dmp400023928|pacid:24389165 | highly similar to ( 847) AT1G70520 | Symbols: | protein kinase family protein | chr1:26584888-26587334 REVERSEmoderately similar to ( 209) NORK_MEDTR Nodulation receptor kinase precursor (EC 2.7.11.1) (Does not make infections protein 2) (Symbiosis receptor-like kinase) (MtSYMRK) - Medicago truncatula (Barrel medic)moderately similar to ( 497) loc_os01g36790 12001.m09975 protein protein kinase, putative, expressedS_TKc Pkinase_Tyr PTKc S_TKc Pkinase TyrKc PTKc_Srm_Brk PTKc_Src_like PTKc_Jak_rpt2 PTKc_Lck_Blk PTKc_Csk_like SPS1 PTKc_EGFR_like PTKc_Frk_like PTKc_Lyn PTKc_Syk_like PTKc_Abl PTKc_EphR PTKc_InsR_like PTKc_EphR_A2 PTKc_Fes_like PTKc_Hck PTKc_Tec_like PTKc_Fyn_Yrk PTKc_Jak2_Jak3_rpt2 PTKc_Ack_like PTKc_Trk PTKc_Itk PTKc_Yes PTKc_HER4 PTKc_Src PTKc_EphR_B PTKc_Ror PTKc_ALK_LTK PTKc_EphR_A PTKc_c-ros PTKc_Chk PTKc_FGFR PTKc_Syk PTKc_DDR PTKc_Met_Ron PTKc_TrkA PTKc_RET PTKc_Jak1_rpt2 PTKc_Btk_Bmx PTKc_Fer PTKc_Zap-70 PTK_HER3 PTKc_Tec_Rlk PTKc_Ror1 PTKc_Tyk2_rpt2 PTKc_FAK PTKc_Axl_like PTKc_IGF-1R PTKc_Csk PTKc_HER2 PTKc_TrkB PTKc_TrkC PTKc_Musk PTKc_Fes PTKc_EGFR PTKc_InsR PTKc_PDGFR PTK_CCK4 PTKc_DDR_like PTKc_Tie1 PTKc_Tie2 PTKc_FGFR4 PTK_Jak_rpt1 PTKc_Axl PTKc_EphR_A10 PTK_Ryk PTKc_DDR2 no original description | - | -2.03 |
| 30.2.17 | signalling.receptor kinases.DUF 26 | pgsc0003dmp400024488|pacid:24428796 | highly similar to ( 793) AT4G21390 | Symbols: B120 | B120; ATP binding / protein kinase/ protein serine/threonine kinase/ sugar binding | chr4:11394458-11397474 REVERSEmoderately similar to ( 231) SLSG3_BRAOL S-locus-specific glycoprotein S13 precursor (SLSG-13) (Fragment) - Brassica oleracea (Wild cabbage)highly similar to ( 666) loc_os07g36570 12007.m07908 protein KI domain interacting kinase 1, putative, expressedTyrKc Pkinase_Tyr S_TKc PTKc S_TKc Pkinase PTKc_Src_like PTKc_Srm_Brk PTKc_Jak_rpt2 PTKc_Ror PTKc_EphR SPS1 PTKc_Lck_Blk PTKc_Csk_like PTKc_EGFR_like PTKc_Frk_like PTKc_ALK_LTK PTKc_Trk PTKc_Fes_like PTKc_InsR_like PTKc_Syk_like PTKc_Musk S_locus_glycop PTKc_Tec_like PTKc_Lyn PTKc_EphR_A PTKc_Itk PTKc_Tyk2_rpt2 PTKc_TrkA PTKc_Jak2_Jak3_rpt2 PTKc_EphR_A2 PTKc_c-ros PTKc_Abl PTKc_Fyn_Yrk PTKc_FGFR PTKc_DDR B_lectin PTKc_TrkB B_lectin PTKc_Src PTKc_EphR_B PTK_CCK4 PTKc_Ror1 PTKc_Axl_like PTKc_Ack_like PTKc_Tec_Rlk PTKc_Chk PTKc_Yes PTKc_Ror2 PTKc_TrkC PTKc_Btk_Bmx PTKc_DDR_like PTKc_Csk PTKc_HER4 PTKc_Hck PTKc_PDGFR PTKc_IGF-1R PTKc_EGFR PTKc_RET PTKc_Fer PTKc_DDR2 PTKc_DDR1 PTKc_InsR PTKc_EphR_A10 PTKc_FGFR2 PTK_HER3 PTKc_Tie2 PTKc_Zap-70 PTKc_Jak1_rpt2 PTKc_Axl PTKc_FGFR3 PTKc_FGFR4 PTKc_Met_Ron PTKc_Tyro3 PTKc_Fes PTK_Ryk B_lectin PTKc_HER2 PTKc_FAK PTKc_FGFR1 PTKc_Tie1 PTKc_Syk PTKc_Tie PAN_AP_plant no original description | - | -4.51 |
| 30.2.17 | signalling.receptor kinases.DUF 26 | pgsc0003dmp400027762|pacid:24378038 | moderately similar to ( 388) AT5G38260 | Symbols: | serine/threonine protein kinase, putative | chr5:15283692-15285837 REVERSEweakly similar to ( 180) KPRO_MAIZE Putative receptor protein kinase ZmPK1 precursor (EC 2.7.11.1) - Zea mays (Maize)moderately similar to ( 403) loc_os01g04460 12001.m07081 protein Ser/Thr protein kinase, putativePkinase_Tyr TyrKc Pkinase PTKc S_TKc S_TKc PTKc_Src_like PTKc_Csk_like PTKc_EGFR_like PTKc_Srm_Brk PTKc_Frk_like PTKc_EphR PTKc_Jak_rpt2 PTKc_Tec_like PTKc_Lck_Blk PTKc_Itk SPS1 PTKc_InsR_like PTKc_Trk PTKc_Abl PTKc_Fes_like PTKc_Axl_like PTKc_Lyn PTKc_Chk PTKc_Syk_like PTKc_Yes PTKc_FGFR PTKc_Ror PTKc_Fyn_Yrk PTKc_Src PTKc_Ack_like PTKc_EphR_B PTKc_Hck PTKc_EphR_A2 PTKc_c-ros PTKc_Tec_Rlk PTKc_HER4 PTKc_Jak2_Jak3_rpt2 PTKc_Btk_Bmx PTKc_HER2 PTKc_EGFR PTKc_Fes PTKc_Csk PTKc_EphR_A10 PTKc_EphR_A PTKc_Fer PTKc_Met_Ron PTK_HER3 PTKc_ALK_LTK PTKc_InsR PTKc_Tie2 PTKc_PDGFR PTKc_Axl PTKc_TrkA PTKc_Tie1 PTKc_Jak1_rpt2 PTKc_Ror1 PTKc_Ror2 PTKc_FAK PTKc_DDR1 PTKc_Tyk2_rpt2 PTKc_FGFR3 PTKc_DDR PTKc_RET PTKc_FGFR1 PTKc_FGFR2 PTKc_TrkC PTKc_FGFR4 PTKc_Tyro3 PTKc_Aatyk PTKc_IGF-1R PTKc_Musk no original description | - | -2.65 |
| 30.2.17 | signalling.receptor kinases.DUF 26 | pgsc0003dmp400030189|pacid:24416508 | moderately similar to ( 331) AT4G32300 | Symbols: SD2-5 | SD2-5 (S-DOMAIN-2 5); carbohydrate binding / kinase/ protein kinase | chr4:15599970-15602435 FORWARDmoderately similar to ( 229) KPRO_MAIZE Putative receptor protein kinase ZmPK1 precursor (EC 2.7.11.1) - Zea mays (Maize)highly similar to ( 502) loc_os04g23760 12004.m07515 protein ATP binding protein, putativePkinase S_TKc TyrKc Pkinase_Tyr S_TKc PTKc PTKc_Jak_rpt2 PTKc_Src_like PTKc_Csk_like PTKc_EphR PTKc_Fes_like SPS1 PTKc_Itk PTKc_Frk_like PTKc_InsR_like PTKc_EphR_A2 PTKc_Tec_like PTKc_Lck_Blk PTKc_Abl PTKc_Srm_Brk PTKc_Jak2_Jak3_rpt2 PTKc_EGFR_like PTKc_Src PTKc_DDR PTKc_EphR_B PTKc_Yes PTKc_Btk_Bmx PTKc_Lyn PTKc_Fes PTKc_Fyn_Yrk PTKc_EphR_A PTKc_Fer PTKc_Trk PTKc_FGFR PTKc_Ror PTKc_Axl_like PTKc_PDGFR PTKc_Chk PTKc_c-ros PTKc_Met_Ron PTKc_Syk PTKc_Tec_Rlk PTKc_Syk_like PTKc_Aatyk PTKc_Csk PTKc_Hck PTKc_ALK_LTK PTKc_Tyk2_rpt2 PTKc_IGF-1R PTKc_InsR PTKc_DDR2 PTKc_Musk PTKc_FAK PTKc_RET PTKc_DDR_like PTKc_Axl PTKc_Ack_like PTKc_EphR_A10 PTKc_Aatyk1_Aatyk3 no original description | - | -3.33 |
| 30.2.17 | signalling.receptor kinases.DUF 26 | pgsc0003dmp400030282|pacid:24414100 | moderately similar to ( 432) AT4G32300 | Symbols: SD2-5 | SD2-5 (S-DOMAIN-2 5); carbohydrate binding / kinase/ protein kinase | chr4:15599970-15602435 FORWARDmoderately similar to ( 220) KPRO_MAIZE Putative receptor protein kinase ZmPK1 precursor (EC 2.7.11.1) - Zea mays (Maize)highly similar to ( 838) loc_os04g23760 12004.m07515 protein ATP binding protein, putativeS_TKc Pkinase S_TKc Pkinase_Tyr TyrKc PTKc PTKc_Csk_like PTKc_Src_like SPS1 PTKc_Frk_like PTKc_Fes_like PTKc_EphR PTKc_Jak_rpt2 PTKc_Srm_Brk PTKc_Lck_Blk PTKc_ALK_LTK PTKc_InsR_like PTKc_EGFR_like PTKc_Fyn_Yrk PTKc_Itk PTKc_Src PTKc_Csk PTKc_Yes PTKc_Lyn PTKc_Fes PTKc_Syk_like PTKc_Tec_like PTKc_Abl PTKc_c-ros PTKc_Trk PTKc_DDR B_lectin PTKc_Ror PTKc_IGF-1R PTKc_Fer PTKc_Jak2_Jak3_rpt2 PTKc_Ack_like PTKc_Musk PTKc_Axl_like PTKc_InsR PTKc_EphR_A2 PTKc_Met_Ron PTKc_Hck PTKc_FGFR PTKc_Axl PTKc_FAK PTKc_Chk PTKc_Btk_Bmx PTKc_Tec_Rlk B_lectin PTKc_Aatyk no original description | - | -4.84 |
| 30.2.17 | signalling.receptor kinases.DUF 26 | pgsc0003dmp400030324|pacid:24416050 | weakly similar to ( 172) AT4G32300 | Symbols: SD2-5 | SD2-5 (S-DOMAIN-2 5); carbohydrate binding / kinase/ protein kinase | chr4:15599970-15602435 FORWARDweakly similar to ( 122) KPRO_MAIZE Putative receptor protein kinase ZmPK1 precursor (EC 2.7.11.1) - Zea mays (Maize)moderately similar to ( 246) loc_os01g10710 12001.m07691 protein receptor-like protein kinase, putativePkinase S_TKc S_TKc TyrKc Pkinase_Tyr PTKc no original description | - | -3.03 |
| 30.2.17 | signalling.receptor kinases.DUF 26 | pgsc0003dmp400032151|pacid:24403387 | moderately similar to ( 340) AT5G60900 | Symbols: RLK1 | RLK1 (RECEPTOR-LIKE PROTEIN KINASE 1); ATP binding / carbohydrate binding / kinase/ protein kinase/ protein serine/threonine kinase/ protein tyrosine kinase/ sugar binding | chr5:24498467-24501494 REVERSEmoderately similar to ( 270) KPRO_MAIZE Putative receptor protein kinase ZmPK1 precursor (EC 2.7.11.1) - Zea mays (Maize)moderately similar to ( 476) loc_os03g61310 12003.m11014 protein receptor-like protein kinase, putative, expressedTyrKc S_TKc Pkinase_Tyr S_TKc Pkinase PTKc PTKc_Csk_like PTKc_Itk PTKc_Tec_like PTKc_Src_like PTKc_EphR PTKc_Srm_Brk PTKc_Fes_like PTKc_Frk_like PTKc_Jak_rpt2 SPS1 PTKc_Btk_Bmx PTKc_InsR_like PTKc_EGFR_like PTKc_Abl PTKc_Lck_Blk PTKc_Syk_like PTKc_Tie2 PTKc_Csk PTKc_Chk PTKc_EphR_B PTKc_Tec_Rlk PTKc_Tie1 PTKc_ALK_LTK PTKc_Met_Ron PTKc_EphR_A10 PTKc_Trk PTKc_FGFR PTKc_EphR_A PTKc_EphR_A2 PTKc_Fyn_Yrk PTKc_Lyn PTKc_Fes no original description | - | -2.42 |
| 30.2.17 | signalling.receptor kinases.DUF 26 | pgsc0003dmp400032555|pacid:24402922 | moderately similar to ( 382) AT5G06740 | Symbols: | lectin protein kinase family protein | chr5:2084094-2086052 FORWARDweakly similar to ( 164) PSKR_DAUCA Phytosulfokine receptor precursor (EC 2.7.11.1) (Phytosulfokine LRR receptor kinase) - Daucus carota (Carrot)highly similar to ( 548) loc_os08g40280 12008.m08008 protein lectin-like protein kinase, putative, expressedLectin_legB Pkinase_Tyr PTKc TyrKc S_TKc S_TKc Pkinase PTKc_Jak_rpt2 PTKc_Ack_like PTKc_EphR PTKc_Src_like PTKc_Syk_like PTKc_Srm_Brk PTKc_InsR_like PTKc_Csk_like PTKc_EGFR_like PTKc_Tec_like PTKc_Trk PTKc_Abl PTKc_Ror PTKc_Met_Ron PTKc_EphR_A2 PTKc_Frk_like PTKc_HER4 PTKc_DDR SPS1 PTKc_Lck_Blk PTKc_Btk_Bmx PTKc_TrkC PTKc_IGF-1R PTKc_Itk PTKc_TrkB PTKc_DDR1 PTKc_FAK PTKc_Fes_like PTKc_InsR PTKc_Chk PTKc_c-ros PTKc_EphR_A PTKc_Src PTKc_ALK_LTK PTKc_Musk PTKc_Lyn PTKc_DDR2 PTKc_Jak2_Jak3_rpt2 PTKc_EphR_B PTKc_Zap-70 PTKc_DDR_like PTKc_Axl_like PTKc_Tyk2_rpt2 PTK_HER3 PTKc_FGFR PTKc_TrkA PTKc_Fyn_Yrk PTKc_Tec_Rlk PTKc_Syk PTKc_Jak1_rpt2 PTKc_Fes PTKc_RET PTKc_FGFR2 PTKc_EphR_A10 PTKc_Tie2 PTKc_Csk PTKc_Yes PTKc_Hck PTKc_Tyro3 PTKc_HER2 PTKc_Ror2 PTKc_Tie1 PTKc_FGFR3 PTK_CCK4 PTKc_EGFR no original description | - | -1.86 |
| 30.2.17 | signalling.receptor kinases.DUF 26 | pgsc0003dmp400034274|pacid:24428562 | moderately similar to ( 307) AT1G66980 | Symbols: | protein kinase family protein / glycerophosphoryl diester phosphodiesterase family protein | chr1:24997491-25001961 REVERSEweakly similar to ( 176) KPRO_MAIZE Putative receptor protein kinase ZmPK1 precursor (EC 2.7.11.1) - Zea mays (Maize)moderately similar to ( 378) loc_os01g02440 12001.m06886 protein Ser/Thr receptor-like kinase, putative, expressedTyrKc Pkinase PTKc Pkinase_Tyr S_TKc S_TKc PTKc_Src_like PTKc_Abl PTKc_EGFR_like PTKc_Jak_rpt2 SPS1 PTKc_Ror PTKc_Srm_Brk PTKc_Csk_like PTKc_Syk_like PTKc_EphR PTKc_Fes_like PTKc_Frk_like PTKc_Itk PTKc_Lck_Blk PTKc_EphR_B PTKc_Btk_Bmx PTKc_Tec_like PTKc_Ack_like PTK_HER3 PTKc_Axl_like PTKc_FGFR PTKc_ALK_LTK PTKc_Trk PTKc_DDR PTKc_Tie2 PTKc_Ror1 PTKc_EphR_A2 PTKc_InsR_like PTKc_Ror2 PTKc_Tie1 PTKc_Lyn PTKc_Tec_Rlk PTKc_DDR1 PTKc_Jak2_Jak3_rpt2 PTKc_Met_Ron PTKc_DDR_like PTKc_Tyro3 PTKc_EphR_A PTKc_EGFR PTKc_Tie PTKc_Axl PTKc_Fyn_Yrk PTKc_DDR2 PTKc_Hck PTKc_c-ros PTKc_Src PTKc_HER4 PTKc_Chk PTKc_Aatyk PTK_CCK4 PTKc_RET PTKc_Yes PTKc_HER2 PTKc_Fer PTKc_FGFR4 PTKc_Fes PTKc_FAK no original description | - | -2.41 |
| 30.2.17 | signalling.receptor kinases.DUF 26 | pgsc0003dmp400034282|pacid:24424848 | moderately similar to ( 243) AT1G66920 | Symbols: | serine/threonine protein kinase, putative | chr1:24965410-24967432 REVERSEweakly similar to ( 174) KPRO_MAIZE Putative receptor protein kinase ZmPK1 precursor (EC 2.7.11.1) - Zea mays (Maize)moderately similar to ( 321) loc_os01g02290 12001.m06871 protein Ser/Thr receptor-like kinase, putative, expressedPkinase TyrKc S_TKc Pkinase_Tyr S_TKc PTKc PTKc_EGFR_like PTKc_Jak_rpt2 PTKc_Src_like PTKc_EphR SPS1 PTKc_Srm_Brk PTKc_Csk_like PTKc_Ror PTKc_EphR_B PTKc_Fes_like PTKc_EphR_A2 PTKc_Trk PTKc_EphR_A no original description | - | -2.91 |
| 30.2.17 | signalling.receptor kinases.DUF 26 | pgsc0003dmp400034283|pacid:24427399 | moderately similar to ( 329) AT1G66910 | Symbols: | protein kinase, putative | chr1:24961634-24963941 REVERSEweakly similar to ( 188) KPRO_MAIZE Putative receptor protein kinase ZmPK1 precursor (EC 2.7.11.1) - Zea mays (Maize)moderately similar to ( 430) loc_os01g02600 12001.m06902 protein Ser/Thr receptor-like kinase, putative, expressedPkinase TyrKc PTKc Pkinase_Tyr S_TKc S_TKc PTKc_Src_like PTKc_EGFR_like SPS1 PTKc_Jak_rpt2 PTKc_Abl PTKc_EphR PTKc_Syk_like PTKc_Csk_like PTKc_Srm_Brk PTKc_Ror PTKc_Fes_like PTKc_Frk_like PTKc_Lck_Blk PTKc_EphR_B PTKc_Itk PTKc_InsR_like PTK_HER3 PTKc_EphR_A2 PTKc_Tec_like PTKc_Ack_like PTKc_Jak2_Jak3_rpt2 PTKc_Chk PTKc_Axl_like PTKc_FGFR PTKc_Tie2 PTKc_EGFR PTKc_Btk_Bmx PTKc_EphR_A PTKc_Tie1 PTKc_Hck PTKc_Lyn PTKc_Trk PTKc_Tec_Rlk PTKc_Fyn_Yrk PTKc_Tie PTKc_HER4 PTKc_DDR PTKc_ALK_LTK PTKc_Ror1 PTKc_Fer PTKc_Ror2 PTKc_Axl PTKc_DDR_like PTKc_Src PTKc_DDR1 PTKc_Fes PTKc_HER2 no original description | - | -2.72 |
| 30.2.17 | signalling.receptor kinases.DUF 26 | pgsc0003dmp400038060|pacid:24397857 | moderately similar to ( 443) AT5G20050 | Symbols: | protein kinase family protein | chr5:6774381-6775739 FORWARDmoderately similar to ( 206) KPRO_MAIZE Putative receptor protein kinase ZmPK1 precursor (EC 2.7.11.1) - Zea mays (Maize)moderately similar to ( 387) loc_os06g05070 12006.m05236 protein receptor-like protein kinase-like protein, putative, expressedPkinase S_TKc S_TKc Pkinase_Tyr TyrKc PTKc PTKc_Csk_like SPS1 PTKc_Chk PTKc_Abl PTKc_Jak_rpt2 PTKc_Tec_like PTKc_Src_like PTKc_Itk PTKc_Frk_like PTKc_Csk PTKc_Fes_like PTKc_EGFR_like PTKc_PDGFR PTKc_Tec_Rlk PTKc_FGFR PTKc_Syk_like PTKc_Srm_Brk PTKc_Musk PTKc_Ror PTKc_Ack_like PTKc_Fer PTKc_Btk_Bmx no original description | - | -2.25 |
| 30.2.17 | signalling.receptor kinases.DUF 26 | pgsc0003dmp400041196|pacid:24424216 | highly similar to ( 780) AT1G11050 | Symbols: | protein kinase family protein | chr1:3681892-3683769 FORWARDmoderately similar to ( 206) NORK_MEDTR Nodulation receptor kinase precursor (EC 2.7.11.1) (Does not make infections protein 2) (Symbiosis receptor-like kinase) (MtSYMRK) - Medicago truncatula (Barrel medic)highly similar to ( 557) loc_os05g34950 12005.m07725 protein protein kinase, putative, expressedS_TKc S_TKc TyrKc Pkinase PTKc Pkinase_Tyr PTKc_Csk_like PTKc_Src_like PTKc_Srm_Brk PTKc_Frk_like PTKc_Trk PTKc_Ror PTKc_Jak_rpt2 SPS1 PTKc_Fes_like PTKc_Itk PTKc_EGFR_like PTKc_c-ros PTKc_Axl_like PTKc_Abl PTKc_Src PTKc_InsR_like PTKc_Chk PTKc_Tec_like PTKc_Tyk2_rpt2 PTKc_Fyn_Yrk PTKc_EphR PTKc_TrkB PTKc_Met_Ron PTKc_Btk_Bmx PTKc_Yes PTKc_Syk_like PTKc_Jak2_Jak3_rpt2 PTKc_Csk PTKc_FGFR PTKc_Lck_Blk PTKc_Lyn PTKc_ALK_LTK PTKc_Fer PTKc_TrkA PTKc_Ack_like PTKc_Hck PTK_CCK4 PTKc_Tec_Rlk PTKc_Musk PTKc_Tie1 PTKc_TrkC PTKc_DDR PTKc_Fes PTKc_Jak1_rpt2 PTKc_Tie2 PTKc_Axl no original description | - | -2.36 |
| 30.2.17 | signalling.receptor kinases.DUF 26 | pgsc0003dmp400041758|pacid:24395098 | weakly similar to ( 182) AT3G57700 | Symbols: | protein kinase, putative | chr3:21384917-21385939 FORWARDweakly similar to ( 107) KPRO_MAIZE Putative receptor protein kinase ZmPK1 precursor (EC 2.7.11.1) - Zea mays (Maize)weakly similar to ( 144) loc_os01g26210 12001.m09075 protein OsWAK6 - OsWAK receptor-like protein kinase, expressedS_TKc S_TKc Pkinase TyrKc PTKc Pkinase_Tyr SPS1 PTKc_Csk_like PTKc_DDR no original description | - | -4.76 |
| 30.2.17 | signalling.receptor kinases.DUF 26 | pgsc0003dmp400041968|pacid:24401794 | moderately similar to ( 343) AT2G19130 | Symbols: | S-locus lectin protein kinase family protein | chr2:8293789-8296275 FORWARDhighly similar to ( 629) KPRO_MAIZE Putative receptor protein kinase ZmPK1 precursor (EC 2.7.11.1) - Zea mays (Maize)highly similar to ( 760) loc_os01g47840 12001.m10977 protein S-locus-like receptor protein kinase, putativePkinase S_TKc S_TKc Pkinase_Tyr TyrKc PTKc SPS1 PTKc_Srm_Brk B_lectin PTKc_EGFR_like PTKc_Src_like PTKc_Jak_rpt2 PTKc_Csk_like PTKc_EphR PTKc_InsR_like B_lectin B_lectin PTKc_Frk_like PTKc_EphR_A2 PTKc_Trk PTKc_Syk_like PTKc_Itk no original description | - | -2.17 |
| 30.2.17 | signalling.receptor kinases.DUF 26 | pgsc0003dmp400044321|pacid:24407441 | weakly similar to ( 188) AT5G60900 | Symbols: RLK1 | RLK1 (RECEPTOR-LIKE PROTEIN KINASE 1); ATP binding / carbohydrate binding / kinase/ protein kinase/ protein serine/threonine kinase/ protein tyrosine kinase/ sugar binding | chr5:24498467-24501494 REVERSEweakly similar to ( 164) KPRO_MAIZE Putative receptor protein kinase ZmPK1 precursor (EC 2.7.11.1) - Zea mays (Maize)moderately similar to ( 204) loc_os04g12600 12004.m06503 protein receptor-like protein kinase, putativeS_TKc S_TKc TyrKc Pkinase Pkinase_Tyr PTKc PTKc_Csk_like PTKc_Jak_rpt2 PTKc_Srm_Brk PTKc_Src_like SPS1 PTKc_EphR PTKc_EGFR_like PTKc_Tec_like PTKc_EphR_A2 PTKc_Fes_like PTKc_Btk_Bmx PTKc_Itk PTKc_Met_Ron PTKc_Trk PTKc_ALK_LTK PTKc_InsR_like no original description | 3.72 | - |
| 30.2.17 | signalling.receptor kinases.DUF 26 | pgsc0003dmp400045383|pacid:24417599 | highly similar to ( 615) AT4G21380 | Symbols: ARK3 | ARK3 (A. THALIANA RECEPTOR KINASE 3); kinase/ transmembrane receptor protein serine/threonine kinase | chr4:11389219-11393090 REVERSEmoderately similar to ( 245) SLSG6_BRAOL S-locus-specific glycoprotein S6 precursor (SLSG-6) - Brassica oleracea (Wild cabbage)highly similar to ( 642) loc_os03g35600 12003.m08696 protein serine/threonine-protein kinase receptor precursor, putative, expressedTyrKc Pkinase_Tyr S_TKc PTKc Pkinase S_TKc PTKc_Src_like PTKc_Srm_Brk PTKc_Jak_rpt2 PTKc_Lck_Blk S_locus_glycop PTKc_Frk_like PTKc_Ror PTKc_EGFR_like PTKc_Fyn_Yrk PTKc_Csk_like PTKc_InsR_like PTKc_Trk PTKc_Syk_like PTKc_Src PTKc_ALK_LTK PTKc_Yes PTKc_Lyn PTKc_Musk SPS1 PTKc_Tec_like PTKc_EphR PTKc_Itk PTKc_Axl_like PTKc_Fes_like PTKc_Jak2_Jak3_rpt2 PTKc_c-ros PTKc_Hck PTKc_Ror2 B_lectin PTKc_Abl PTKc_DDR PTK_CCK4 PTKc_TrkB PTKc_Ror1 PTKc_TrkA PTKc_Met_Ron PTKc_Ack_like PTKc_Btk_Bmx B_lectin PTKc_InsR PTKc_EphR_A2 PTKc_Axl PTKc_Chk PTKc_TrkC PTKc_IGF-1R B_lectin PTK_Ryk PTKc_HER4 PTKc_RET PTKc_Tec_Rlk PTKc_EGFR PTKc_DDR_like PTKc_EphR_B PTKc_HER2 PTKc_Tyk2_rpt2 PTKc_FAK PTKc_EphR_A PTKc_DDR2 PTKc_Csk PTKc_FGFR PTKc_PDGFR no original description | - | -2.13 |
| 30.2.17 | signalling.receptor kinases.DUF 26 | pgsc0003dmp400051419|pacid:24382644 | moderately similar to ( 496) AT1G70740 | Symbols: | protein kinase family protein | chr1:26673847-26675687 REVERSEmoderately similar to ( 223) PSKR_DAUCA Phytosulfokine receptor precursor (EC 2.7.11.1) (Phytosulfokine LRR receptor kinase) - Daucus carota (Carrot)moderately similar to ( 323) loc_os03g35600 12003.m08696 protein serine/threonine-protein kinase receptor precursor, putative, expressedTyrKc Pkinase_Tyr PTKc S_TKc S_TKc Pkinase PTKc_Jak_rpt2 PTKc_Trk PTKc_EphR PTKc_Csk_like PTKc_Src_like PTKc_Srm_Brk PTKc_Syk_like PTKc_Fes_like PTKc_Frk_like PTKc_EGFR_like PTKc_Itk PTKc_Ror SPS1 PTKc_TrkA PTKc_Tec_like PTKc_Jak2_Jak3_rpt2 PTKc_DDR PTKc_TrkC PTKc_Lck_Blk PTKc_EphR_B PTKc_TrkB PTKc_ALK_LTK PTKc_Fyn_Yrk PTKc_EphR_A PTKc_PDGFR PTKc_FGFR PTKc_Abl PTKc_Lyn PTKc_Musk PTKc_Fer PTKc_InsR_like PTKc_Src PTKc_Yes PTK_HER3 PTKc_Fes PTKc_Ror1 PTKc_Chk PTKc_EphR_A2 PTKc_DDR_like PTKc_DDR2 PTK_CCK4 PTKc_Tie2 PTKc_Hck PTKc_Tec_Rlk PTKc_c-ros PTKc_Btk_Bmx PTKc_Ack_like PTK_Ryk PTKc_Ror2 PTKc_FGFR1 PTKc_HER2 PTKc_Tyk2_rpt2 PTKc_FGFR2 PTKc_EphR_A10 PTKc_DDR1 PTKc_FGFR4 PTKc_Axl_like PTKc_RET PTKc_Zap-70 PTKc_Met_Ron PTKc_FGFR3 PTKc_Jak1_rpt2 PTKc_Tie1 PTKc_InsR PTKc_EGFR PTKc_FAK PTKc_Csk PTKc_Syk no original description | - | -2.93 |
| 30.2.17 | signalling.receptor kinases.DUF 26 | pgsc0003dmp400052466|pacid:24392173 | moderately similar to ( 269) AT5G25930 | Symbols: | leucine-rich repeat family protein / protein kinase family protein | chr5:9050880-9053978 FORWARDweakly similar to ( 141) NORK_PEA Nodulation receptor kinase precursor (EC 2.7.11.1) - Pisum sativum (Garden pea)moderately similar to ( 256) loc_os05g44770 12005.m08602 protein receptor-like protein kinase 5 precursor, putative, expressedS_TKc S_TKc Pkinase Pkinase_Tyr PTKc TyrKc no original description | - | -4.95 |
| 30.2.17 | signalling.receptor kinases.DUF 26 | pgsc0003dmp400052589|pacid:24407229 | highly similar to ( 799) AT1G34300 | Symbols: | lectin protein kinase family protein | chr1:12503450-12505939 FORWARDmoderately similar to ( 244) KPRO_MAIZE Putative receptor protein kinase ZmPK1 precursor (EC 2.7.11.1) - Zea mays (Maize)highly similar to ( 846) loc_os03g62180 12003.m101559 protein ATP binding protein, putative, expressedPkinase S_TKc Pkinase_Tyr TyrKc S_TKc PTKc PTKc_Csk_like PTKc_EphR PTKc_Src_like PTKc_Srm_Brk PTKc_Frk_like PTKc_Trk PTKc_InsR_like SPS1 PTKc_Abl PTKc_Jak_rpt2 PTKc_EphR_A2 PTKc_FGFR PTKc_EphR_B PTKc_EphR_A PTKc_Tie2 PTKc_Chk PTKc_Tec_like PTKc_Syk_like PTKc_Fes_like PTKc_EGFR_like PTKc_Ror PTKc_Itk PTKc_TrkA PTKc_TrkB PTKc_Met_Ron PTKc_ALK_LTK PTKc_Musk PTKc_Lck_Blk PTKc_Tie1 PTKc_Axl_like PTKc_Fyn_Yrk PTKc_Src PTKc_Btk_Bmx PTKc_Tie PTKc_Tec_Rlk PTKc_Lyn PTKc_TrkC PTKc_DDR PTKc_Jak2_Jak3_rpt2 PTKc_Yes PTKc_Ror1 PTKc_FGFR4 PTKc_Hck PTKc_Tyk2_rpt2 PTKc_Fer PTKc_RET PTKc_Ack_like PTKc_Fes PTKc_FGFR1 PTKc_PDGFR PTKc_InsR PTK_HER3 PTKc_FGFR3 PTKc_c-ros PTKc_Csk PTK_CCK4 PTKc_Zap-70 PTKc_Syk PTKc_IGF-1R no original description | - | -1.82 |
| 30.2.17 | signalling.receptor kinases.DUF 26 | pgsc0003dmp400055135|pacid:24399675 | moderately similar to ( 419) AT5G60900 | Symbols: RLK1 | RLK1 (RECEPTOR-LIKE PROTEIN KINASE 1); ATP binding / carbohydrate binding / kinase/ protein kinase/ protein serine/threonine kinase/ protein tyrosine kinase/ sugar binding | chr5:24498467-24501494 REVERSEmoderately similar to ( 314) KPRO_MAIZE Putative receptor protein kinase ZmPK1 precursor (EC 2.7.11.1) - Zea mays (Maize)moderately similar to ( 472) loc_os04g12600 12004.m06503 protein receptor-like protein kinase, putativeTyrKc Pkinase_Tyr S_TKc Pkinase S_TKc PTKc PTKc_EphR PTKc_Syk_like PTKc_Tec_like PTKc_Csk_like PTKc_Src_like PTKc_Srm_Brk PTKc_Frk_like PTKc_InsR_like PTKc_Trk PTKc_Itk PTKc_Btk_Bmx PTKc_EphR_A2 PTKc_Jak_rpt2 PTKc_EGFR_like PTKc_Abl SPS1 PTKc_DDR PTKc_ALK_LTK PTKc_Fes_like PTKc_DDR_like PTKc_Ror PTKc_Tie2 PTKc_TrkB PTKc_Syk PTKc_EphR_A PTKc_FGFR PTKc_Tec_Rlk PTKc_Met_Ron PTKc_Zap-70 PTKc_Lck_Blk PTKc_EphR_B PTKc_Csk PTKc_PDGFR PTKc_c-ros PTKc_TrkC PTKc_Aatyk PTK_CCK4 PTKc_TrkA PTKc_Fes PTKc_InsR PTKc_Axl_like PTKc_HER4 PTKc_Chk PTKc_FAK PTKc_Lyn PTKc_Tie PTKc_Tie1 PTK_Ryk PTKc_EphR_A10 PTKc_DDR2 PTKc_RET PTKc_IGF-1R PTKc_Fyn_Yrk PTKc_FGFR4 PTKc_Jak2_Jak3_rpt2 PTKc_DDR1 PTKc_Axl PTKc_Ack_like PTKc_Fer PTKc_Hck PTKc_Ror1 PTKc_Src PTKc_Yes no original description | - | -2.09 |
| 30.2.17 | signalling.receptor kinases.DUF 26 | pgsc0003dmp400055136|pacid:24399466 | moderately similar to ( 446) AT5G60900 | Symbols: RLK1 | RLK1 (RECEPTOR-LIKE PROTEIN KINASE 1); ATP binding / carbohydrate binding / kinase/ protein kinase/ protein serine/threonine kinase/ protein tyrosine kinase/ sugar binding | chr5:24498467-24501494 REVERSEmoderately similar to ( 324) KPRO_MAIZE Putative receptor protein kinase ZmPK1 precursor (EC 2.7.11.1) - Zea mays (Maize)moderately similar to ( 494) loc_os04g12540 12004.m06497 protein receptor-like protein kinase, putative, expressedPkinase_Tyr Pkinase S_TKc S_TKc TyrKc PTKc PTKc_Csk_like PTKc_Frk_like PTKc_Src_like PTKc_ALK_LTK PTKc_Jak_rpt2 PTKc_InsR_like PTKc_EphR PTKc_Tec_like PTKc_Syk_like PTKc_Met_Ron PTKc_Srm_Brk PTKc_Itk SPS1 PTKc_Abl PTKc_Fes_like PTKc_FGFR PTKc_EphR_A2 PTKc_EGFR_like PTKc_c-ros PTKc_EphR_B PTKc_Tie2 PTKc_Trk PTKc_EphR_A PTKc_DDR PTKc_Btk_Bmx PTKc_Axl_like PTKc_Tec_Rlk PTKc_Tyro3 PTKc_IGF-1R PTKc_Tie1 PTKc_Syk PTKc_Chk PTKc_Axl PTKc_FAK PTKc_Lck_Blk PTKc_HER4 PTKc_Ror PTKc_Zap-70 B_lectin B_lectin PTKc_Ack_like PTKc_InsR PTKc_Tie PTKc_Fer PTKc_Fes PTKc_Musk PTKc_FGFR4 PTKc_PDGFR PTKc_TrkB PTKc_FGFR2 PTKc_Lyn PTKc_RET PTKc_Fyn_Yrk PTKc_EGFR PTKc_Csk PTK_CCK4 PTKc_FGFR1 no original description | - | -2.01 |
| 30.2.17 | signalling.receptor kinases.DUF 26 | pgsc0003dmp400063415|pacid:24400029 | highly similar to ( 896) AT2G32800 | Symbols: AP4.3A | AP4.3A; ATP binding / protein kinase/ protein serine/threonine kinase/ protein tyrosine kinase | chr2:13916478-13919033 FORWARDweakly similar to ( 199) PSKR_DAUCA Phytosulfokine receptor precursor (EC 2.7.11.1) (Phytosulfokine LRR receptor kinase) - Daucus carota (Carrot)highly similar to ( 895) loc_os11g25860 12011.m06507 protein ATP binding protein, putative, expressedPkinase S_TKc PTKc S_TKc Pkinase_Tyr TyrKc SPS1 PTKc_Trk PTKc_EGFR_like PTKc_Jak_rpt2 PTKc_Csk_like PTKc_EphR_A2 PTKc_Src_like PTKc_Frk_like PTKc_TrkB PTKc_TrkA PTKc_TrkC PTKc_EphR_B PTKc_Ror PTKc_Srm_Brk PTKc_InsR_like PTKc_FAK PTKc_Ack_like PTKc_EphR PTKc_EphR_A PTKc_Abl PTKc_ALK_LTK PTKc_Musk PTKc_Jak2_Jak3_rpt2 PTKc_Tie1 no original description | - | -4.03 |
| 30.2.24 | signalling.receptor kinases.S-locus glycoprotein like | pgsc0003dmp400008766|pacid:24385252 | highly similar to ( 854) AT4G27290 | Symbols: | ATP binding / protein kinase/ protein serine/threonine kinase/ protein tyrosine kinase/ sugar binding | chr4:13666281-13669202 FORWARDmoderately similar to ( 347) SLSG6_BRAOL S-locus-specific glycoprotein S6 precursor (SLSG-6) - Brassica oleracea (Wild cabbage)highly similar to ( 704) loc_os07g36544 12007.m07905 protein serine/threonine-protein kinase receptor precursor, putative, expressedTyrKc Pkinase_Tyr PTKc S_locus_glycop Pkinase S_TKc S_TKc PTKc_Jak_rpt2 PTKc_Src_like PTKc_Srm_Brk PTKc_Csk_like PTKc_Trk PTKc_Syk_like PTKc_EGFR_like PTKc_c-ros PTKc_InsR_like PTKc_Ror B_lectin PTKc_ALK_LTK PTKc_Lck_Blk B_lectin PTKc_Frk_like PTKc_Itk B_lectin PTKc_EphR PTK_CCK4 PTKc_Ack_like PTKc_FGFR PTKc_Tec_like PTKc_Jak2_Jak3_rpt2 PTKc_Axl_like PTKc_Fes_like PTKc_Abl PTKc_Lyn PTKc_Ror2 SPS1 PTKc_IGF-1R PTKc_DDR PTKc_Src PTKc_TrkA PTKc_Fyn_Yrk PTKc_Ror1 PTKc_EphR_A2 PTKc_TrkC PTKc_TrkB PTKc_Hck PTKc_Tec_Rlk PTKc_Yes PTKc_Musk PTKc_Met_Ron PTKc_Btk_Bmx PTKc_RET PTKc_Tyk2_rpt2 PTKc_FGFR4 PTKc_InsR PTKc_HER4 PTKc_EGFR PAN_2 PTKc_Chk PTKc_EphR_A PTKc_DDR_like PTKc_Axl PTKc_Csk PTKc_PDGFR PTKc_EphR_B PTKc_Tyro3 PTKc_FGFR2 PTKc_Fer PTK_Ryk PTK_HER3 PTKc_Zap-70 PTKc_FGFR1 PTKc_DDR1 PTKc_FGFR3 PAN_AP_plant PTKc_DDR2 PTKc_HER2 PTKc_Tie2 PTKc_Syk PTKc_Fes no original description | - | -1.71 |
| 30.2.24 | signalling.receptor kinases.S-locus glycoprotein like | pgsc0003dmp400031300|pacid:24427019 | moderately similar to ( 484) AT4G27290 | Symbols: | ATP binding / protein kinase/ protein serine/threonine kinase/ protein tyrosine kinase/ sugar binding | chr4:13666281-13669202 FORWARDmoderately similar to ( 285) SLSG6_BRAOL S-locus-specific glycoprotein S6 precursor (SLSG-6) - Brassica oleracea (Wild cabbage)moderately similar to ( 409) loc_os03g35600 12003.m08696 protein serine/threonine-protein kinase receptor precursor, putative, expressedS_locus_glycop B_lectin B_lectin B_lectin PAN_2 no original description | - | -7.61 |
| 30.2.25 | signalling.receptor kinases.wall associated kinase | pgsc0003dmp400029894|pacid:24400360 | moderately similar to ( 216) AT1G21270 | Symbols: WAK2 | WAK2; ATP binding / calcium ion binding / protein kinase/ protein serine/threonine kinase | chr1:7444997-7447345 FORWARDmoderately similar to ( 247) loc_os02g41500 12002.m09186 protein OsWAK13 - OsWAK receptor-like protein kinase, expressed no original description | - | -2.3 |
| 30.2.3 | signalling.receptor kinases.leucine rich repeat III | pgsc0003dmp400004821|pacid:24399595 | highly similar to ( 704) AT2G36570 | Symbols: | leucine-rich repeat transmembrane protein kinase, putative | chr2:15335583-15337725 FORWARDweakly similar to ( 173) PSKR_DAUCA Phytosulfokine receptor precursor (EC 2.7.11.1) (Phytosulfokine LRR receptor kinase) - Daucus carota (Carrot)moderately similar to ( 333) loc_os01g60330 12001.m12161 protein ATP binding protein, putative, expressedPTKc Pkinase_Tyr TyrKc Pkinase S_TKc S_TKc PTKc_Src_like PTKc_Csk_like PTKc_Jak_rpt2 PTKc_Frk_like PTKc_Fes_like PTKc_Srm_Brk PTKc_EphR PTKc_Src PTKc_Lck_Blk PTKc_EGFR_like SPS1 PTKc_Trk PTKc_EphR_B PTKc_Fyn_Yrk PTKc_Abl PTKc_Ror PTKc_Syk_like PTKc_Yes PTKc_EphR_A PTKc_Hck PTKc_Itk PTKc_Lyn PTKc_Fer PTKc_TrkC PTKc_TrkA PTKc_EphR_A2 PTKc_Axl_like PTKc_Jak2_Jak3_rpt2 no original description | -31.03 | - |
| 30.2.3 | signalling.receptor kinases.leucine rich repeat III | pgsc0003dmp400023342|pacid:24399746 | highly similar to ( 957) AT3G51740 | Symbols: IMK2 | IMK2 (INFLORESCENCE MERISTEM RECEPTOR-LIKE KINASE 2); ATP binding / kinase/ protein kinase/ protein serine/threonine kinase | chr3:19189248-19191842 FORWARDmoderately similar to ( 295) PSKR_DAUCA Phytosulfokine receptor precursor (EC 2.7.11.1) (Phytosulfokine LRR receptor kinase) - Daucus carota (Carrot)highly similar to ( 809) loc_os03g18630 12003.m07284 protein receptor-like kinase RHG1, putative, expressedS_TKc Pkinase S_TKc PTKc TyrKc Pkinase_Tyr PTKc_Jak_rpt2 SPS1 PTKc_Csk_like PTKc_Src_like PTKc_Frk_like PTKc_Met_Ron PTKc_Abl PTKc_Fes_like PTKc_Csk PTKc_Srm_Brk PTKc_EGFR_like PTKc_EphR PTKc_Itk PTKc_Ror PTKc_Chk PTKc_Jak2_Jak3_rpt2 PTKc_TrkB PTKc_Syk_like PTKc_Lck_Blk PTKc_Trk PTKc_Musk PTKc_Tec_like PTKc_Tyro3 PTKc_Src no original description | -9.73 | - |
| 30.2.3 | signalling.receptor kinases.leucine rich repeat III | pgsc0003dmp400024950|pacid:24405205 | moderately similar to ( 419) AT3G17840 | Symbols: RLK902 | RLK902; ATP binding / kinase/ protein serine/threonine kinase | chr3:6106092-6108430 FORWARDweakly similar to ( 177) PSKR_DAUCA Phytosulfokine receptor precursor (EC 2.7.11.1) (Phytosulfokine LRR receptor kinase) - Daucus carota (Carrot)moderately similar to ( 432) loc_os03g50450 12003.m10037 protein atypical receptor-like kinase MARK, putative, expressedPkinase S_TKc Pkinase_Tyr S_TKc PTKc TyrKc PTKc_Src_like SPS1 PTKc_EphR PTKc_Jak_rpt2 PTKc_Frk_like PTKc_Srm_Brk PTKc_Csk_like PTKc_EGFR_like PTKc_Trk PTKc_Ror PTKc_Fes_like PTKc_Src PTKc_Jak2_Jak3_rpt2 PTKc_Lyn PTKc_Tec_like PTKc_Itk PTKc_Lck_Blk PTKc_Fyn_Yrk PTKc_Yes PTKc_Hck PTKc_EphR_A2 PTKc_Abl PTKc_EphR_B PTKc_Met_Ron PTKc_Chk PTK_CCK4 PTKc_Syk_like no original description | -5.47 | - |
| 30.2.99 | signalling.receptor kinases.misc | pgsc0003dmp400046240|pacid:24398478 | weakly similar to ( 157) AT5G01740 | Symbols: | FUNCTIONS IN: molecular_function unknown; INVOLVED IN: biological_process unknown; LOCATED IN: cellular_component unknown; EXPRESSED IN: 18 plant structures; EXPRESSED DURING: 9 growth stages; CONTAINS InterPro DOMAIN/s: Wound-induced protein, Wun1 (InterPro:IPR009798); BEST Arabidopsis thaliana protein match is: SAG20 (SENESCENCE ASSOCIATED GENE 20) (TAIR:AT3G10985.1); Has 49 Blast hits to 49 proteins in 12 species: Archae - 0; Bacteria - 4; Metazoa - 0; Fungi - 0; Plants - 45; Viruses - 0; Other Eukaryotes - 0 (source: NCBI BLink). | chr5:280793-281281 FORWARDvery weakly similar to (96.3) loc_os05g27590 12005.m07047 protein wound-induced protein WI12 containing protein, expressedWI12 no original description | - | 3.22 |
| 30.3 | signalling.calcium | pgsc0003dmp400000651|pacid:24378368 | highly similar to ( 538) AT4G33050 | Symbols: EDA39 | EDA39 (embryo sac development arrest 39); calmodulin binding | chr4:15945235-15946736 REVERSEmoderately similar to ( 487) loc_os05g10840 12005.m083645 protein calmodulin binding protein, putative, expressed no original description | - | -3.71 |
| 30.3 | signalling.calcium | pgsc0003dmp400002390|pacid:24424715 | weakly similar to ( 105) AT5G39670 | Symbols: | calcium-binding EF hand family protein | chr5:15883270-15883884 FORWARD no original description | - | -3.24 |
| 30.3 | signalling.calcium | pgsc0003dmp400009535|pacid:24381002 | moderately similar to ( 396) AT1G73805 | Symbols: | calmodulin binding | chr1:27745761-27749178 REVERSEmoderately similar to ( 349) loc_os01g04280 12001.m07063 protein calmodulin binding protein, putative, expressedCalmodulin_bind no original description | - | -5.6 |
| 30.3 | signalling.calcium | pgsc0003dmp400009947|pacid:24406232 | moderately similar to ( 408) AT1G73805 | Symbols: | calmodulin binding | chr1:27745761-27749178 REVERSEmoderately similar to ( 330) loc_os02g08120 12002.m33346 protein calmodulin binding protein, putative, expressedCalmodulin_bind no original description | - | -17.63 |
| 30.3 | signalling.calcium | pgsc0003dmp400010205|pacid:24405811 | weakly similar to ( 156) AT1G18210 | Symbols: | calcium-binding protein, putative | chr1:6268273-6268785 REVERSEvery weakly similar to (95.1) CALM_CHLRE Calmodulin (CaM) - Chlamydomonas reinhardtiiweakly similar to ( 144) loc_os05g13580 12005.m05773 protein calmodulin-related protein 2, touch-induced, putative, expressedFRQ1 no original description | - | -6.25 |
| 30.3 | signalling.calcium | pgsc0003dmp400010464|pacid:24394957 | weakly similar to ( 133) AT1G76650 | Symbols: CML38 | calcium-binding EF hand family protein | chr1:28766909-28767442 REVERSEweakly similar to ( 132) loc_os01g72550 12001.m13290 protein calmodulin-like protein 41, putative no original description | - | -9.81 |
| 30.3 | signalling.calcium | pgsc0003dmp400011026|pacid:24411424 | weakly similar to ( 181) AT4G34150 | Symbols: | C2 domain-containing protein | chr4:16355035-16356955 FORWARDweakly similar to ( 158) loc_os06g43190 12006.m71447 protein calcium-binding protein, putative, expressed no original description | - | -2.95 |
| 30.3 | signalling.calcium | pgsc0003dmp400011434|pacid:24401261 | weakly similar to ( 127) AT4G14750 | Symbols: IQD19 | IQD19 (IQ-domain 19); calmodulin binding | chr4:8470449-8471903 FORWARDweakly similar to ( 114) loc_os10g28420 12010.m05722 protein IQ calmodulin-binding motif family protein no original description | - | 2.84 |
| 30.3 | signalling.calcium | pgsc0003dmp400014336|pacid:24411842 | highly similar to ( 823) AT3G57530 | Symbols: CPK32, ATCPK32, CDPK32 | CPK32 (CALCIUM-DEPENDENT PROTEIN KINASE 32); calcium-dependent protein kinase C/ calmodulin-dependent protein kinase/ kinase/ protein binding | chr3:21296898-21299351 REVERSEhighly similar to ( 508) CDPK_SOYBN Calcium-dependent protein kinase SK5 (EC 2.7.11.1) (CDPK) - Glycine max (Soybean)highly similar to ( 815) loc_os07g38120 12007.m08061 protein calcium-dependent protein kinase, isoform AK1, putative, expressedS_TKc S_TKc Pkinase SPS1 Pkinase_Tyr FRQ1 TyrKc PTKc no original description | - | -2.42 |
| 30.3 | signalling.calcium | pgsc0003dmp400014356|pacid:24410306 | weakly similar to ( 154) AT3G10190 | Symbols: | calmodulin, putative | chr3:3155309-3155938 FORWARDvery weakly similar to (83.2) CALM3_PETHY Calmodulin-related protein - Petunia hybrida (Petunia)weakly similar to ( 109) loc_os01g72100 12001.m13246 protein polcalcin Jun o 2, putative, expressedFRQ1 no original description | - | -2.26 |
| 30.3 | signalling.calcium | pgsc0003dmp400014421|pacid:24390303 | highly similar to ( 561) AT4G33050 | Symbols: EDA39 | EDA39 (embryo sac development arrest 39); calmodulin binding | chr4:15945235-15946736 REVERSEmoderately similar to ( 476) loc_os01g38980 12001.m10178 protein calmodulin binding protein, putative, expressed no original description | 3.39 | -6.03 |
| 30.3 | signalling.calcium | pgsc0003dmp400018400|pacid:24428117 | weakly similar to ( 150) AT1G18210 | Symbols: | calcium-binding protein, putative | chr1:6268273-6268785 REVERSEvery weakly similar to ( 100) CALM_CHLRE Calmodulin (CaM) - Chlamydomonas reinhardtiiweakly similar to ( 149) loc_os05g13580 12005.m05773 protein calmodulin-related protein 2, touch-induced, putative, expressedFRQ1 no original description | - | -4.99 |
| 30.3 | signalling.calcium | pgsc0003dmp400018793|pacid:24385260 | weakly similar to ( 123) AT2G41410 | Symbols: | calmodulin, putative | chr2:17262085-17262735 REVERSEvery weakly similar to (80.9) loc_os05g31620 12005.m07396 protein polcalcin Jun o 2, putative, expressed no original description | - | -1.78 |
| 30.3 | signalling.calcium | pgsc0003dmp400023079|pacid:24426709 | nearly identical (1688) AT4G37640 | Symbols: ACA2 | ACA2 (CALCIUM ATPASE 2); calcium ion transmembrane transporter/ calcium-transporting ATPase/ calmodulin binding | chr4:17683225-17686808 REVERSEnearly identical (1579) ACA1_ORYSA Calcium-transporting ATPase 1, plasma membrane-type (EC 3.6.3.8) (Ca(2+)-ATPase isoform 1) (Plastid envelope ATPase 1) - Oryza sativa (Rice)nearly identical (1579) loc_os12g39660 12012.m07750 protein calcium-transporting ATPase 2, plasma membrane-type, putative, expressedMgtA PRK10517 E1-E2_ATPase KdpB PRK01122 ZntA PRK10671 zntA no original description | - | -7.68 |
| 30.3 | signalling.calcium | pgsc0003dmp400025697|pacid:24387187 | weakly similar to ( 126) AT5G57580 | Symbols: | calmodulin-binding protein | chr5:23314994-23317683 REVERSEweakly similar to ( 126) loc_os04g36660 12004.m08706 protein calmodulin binding protein, putative, expressedCalmodulin_bind no original description | - | -3.77 |
| 30.3 | signalling.calcium | pgsc0003dmp400026832|pacid:24379725 | very weakly similar to (82.0) AT2G15760 | Symbols: | calmodulin-binding protein | chr2:6865807-6866754 REVERSE no original description | - | -3.02 |
| 30.3 | signalling.calcium | pgsc0003dmp400031540|pacid:24402490 | weakly similar to ( 127) AT2G15760 | Symbols: | calmodulin-binding protein | chr2:6865807-6866754 REVERSEDUF1645 no original description | - | -4.69 |
| 30.3 | signalling.calcium | pgsc0003dmp400032979|pacid:24402022 | highly similar to ( 760) AT3G63380 | Symbols: | calcium-transporting ATPase, plasma membrane-type, putative / Ca(2+)-ATPase, putative (ACA12) | chr3:23407112-23410213 REVERSEmoderately similar to ( 465) ACA5_ORYSA Probable calcium-transporting ATPase 5, plasma membrane-type (EC 3.6.3.8) (Ca(2+)-ATPase isoform 5) - Oryza sativa (Rice)highly similar to ( 642) loc_os10g28240 12010.m05706 protein calcium-transporting ATPase 13, plasma membrane-type, putative, expressedMgtA ZntA PRK10517 E1-E2_ATPase no original description | - | -17.35 |
| 30.3 | signalling.calcium | pgsc0003dmp400033763|pacid:24422805 | moderately similar to ( 296) AT5G28830 | Symbols: | calcium-binding EF hand family protein | chr5:10856801-10858394 FORWARD no original description | - | -2.42 |
| 30.3 | signalling.calcium | pgsc0003dmp400035212|pacid:24425934 | weakly similar to ( 129) AT1G76650 | Symbols: CML38 | calcium-binding EF hand family protein | chr1:28766909-28767442 REVERSEweakly similar to ( 130) loc_os01g72550 12001.m13290 protein calmodulin-like protein 41, putative no original description | - | -7.35 |
| 30.3 | signalling.calcium | pgsc0003dmp400037126|pacid:24425584 | nearly identical (1652) AT4G37640 | Symbols: ACA2 | ACA2 (CALCIUM ATPASE 2); calcium ion transmembrane transporter/ calcium-transporting ATPase/ calmodulin binding | chr4:17683225-17686808 REVERSEnearly identical (1561) ACA1_ORYSA Calcium-transporting ATPase 1, plasma membrane-type (EC 3.6.3.8) (Ca(2+)-ATPase isoform 1) (Plastid envelope ATPase 1) - Oryza sativa (Rice)nearly identical (1561) loc_os12g39660 12012.m07750 protein calcium-transporting ATPase 2, plasma membrane-type, putative, expressedMgtA PRK10517 E1-E2_ATPase KdpB PRK01122 ZntA zntA no original description | - | -2.31 |
| 30.3 | signalling.calcium | pgsc0003dmp400038709|pacid:24427118 | weakly similar to ( 112) AT4G27280 | Symbols: | calcium-binding EF hand family protein | chr4:13663770-13664162 REVERSEweakly similar to ( 115) loc_os06g46950 12006.m09221 protein EF-hand Ca2+-binding protein CCD1, putative, expressed no original description | - | -3.89 |
| 30.3 | signalling.calcium | pgsc0003dmp400039912|pacid:24382710 | weakly similar to ( 104) AT1G21550 | Symbols: | calcium-binding protein, putative | chr1:7553317-7553784 REVERSEvery weakly similar to (83.6) loc_os04g41540 12004.m09132 protein calmodulin, putative, expressed no original description | - | -2.92 |
| 30.3 | signalling.calcium | pgsc0003dmp400043424|pacid:24412717 | weakly similar to ( 136) AT4G27280 | Symbols: | calcium-binding EF hand family protein | chr4:13663770-13664162 REVERSEweakly similar to ( 107) loc_os06g46950 12006.m09221 protein EF-hand Ca2+-binding protein CCD1, putative, expressed no original description | 3.94 | -4.67 |
| 30.3 | signalling.calcium | pgsc0003dmp400043425|pacid:24410566 | weakly similar to ( 130) AT4G27280 | Symbols: | calcium-binding EF hand family protein | chr4:13663770-13664162 REVERSEweakly similar to ( 103) loc_os06g46950 12006.m09221 protein EF-hand Ca2+-binding protein CCD1, putative, expressed no original description | 4.11 | -6.95 |
| 30.3 | signalling.calcium | pgsc0003dmp400044873|pacid:24418288 | moderately similar to ( 206) AT4G34150 | Symbols: | C2 domain-containing protein | chr4:16355035-16356955 FORWARDweakly similar to ( 187) loc_os06g43190 12006.m71447 protein calcium-binding protein, putative, expressed no original description | - | -8.29 |
| 30.3 | signalling.calcium | pgsc0003dmp400045391|pacid:24414455 | weakly similar to ( 124) AT4G27280 | Symbols: | calcium-binding EF hand family protein | chr4:13663770-13664162 REVERSEweakly similar to ( 117) loc_os01g57470 12001.m11898 protein caltractin, putative, expressed no original description | - | -4.22 |
| 30.3 | signalling.calcium | pgsc0003dmp400048164|pacid:24412194 | weakly similar to ( 191) AT4G20780 | Symbols: | calcium-binding protein, putative | chr4:11133309-11133884 REVERSEmoderately similar to ( 313) CAST_SOLTU Calcium-binding protein CAST - Solanum tuberosum (Potato)weakly similar to ( 152) loc_os03g21380 12003.m35206 protein calcium-binding protein CAST, putative, expressed no original description | - | -3.59 |
| 30.3 | signalling.calcium | pgsc0003dmp400049012|pacid:24411067 | very weakly similar to ( 100) AT3G56880 | Symbols: | VQ motif-containing protein | chr3:21060044-21060781 FORWARD no original description | - | -10.54 |
| 30.3 | signalling.calcium | pgsc0003dmp400050162|pacid:24394827 | weakly similar to ( 159) AT1G18210 | Symbols: | calcium-binding protein, putative | chr1:6268273-6268785 REVERSEvery weakly similar to (95.1) CALM1_SOLTU Calmodulin-1 (CaM-1) - Solanum tuberosum (Potato)weakly similar to ( 133) loc_os05g13580 12005.m05773 protein calmodulin-related protein 2, touch-induced, putative, expressedFRQ1 no original description | - | -6.89 |
| 30.3 | signalling.calcium | pgsc0003dmp400053306|pacid:24426038 | weakly similar to ( 151) AT5G37770 | Symbols: TCH2, CML24 | TCH2 (TOUCH 2); calcium ion binding | chr5:14999075-14999560 REVERSEweakly similar to ( 106) CALM_CHLRE Calmodulin (CaM) - Chlamydomonas reinhardtiiweakly similar to ( 154) loc_os05g13580 12005.m05773 protein calmodulin-related protein 2, touch-induced, putative, expressedFRQ1 no original description | - | -9.72 |
| 30.3 | signalling.calcium | pgsc0003dmp400053806|pacid:24414821 | weakly similar to ( 118) AT2G43290 | Symbols: MSS3 | MSS3 (multicopy suppressors of snf4 deficiency in yeast 3); calcium ion binding | chr2:17991308-17991955 REVERSEweakly similar to ( 101) loc_os12g12730 12012.m05246 protein EF hand family protein, expressed no original description | - | -4.96 |
| 30.3 | signalling.calcium | pgsc0003dmp400055692|pacid:24414237 | highly similar to ( 535) AT4G33050 | Symbols: EDA39 | EDA39 (embryo sac development arrest 39); calmodulin binding | chr4:15945235-15946736 REVERSEmoderately similar to ( 448) loc_os01g38980 12001.m10178 protein calmodulin binding protein, putative, expressed no original description | 8.69 | -3.29 |
| 30.3 | signalling.calcium | pgsc0003dmp400056198|pacid:24404859 | moderately similar to ( 233) AT2G27030 | Symbols: CAM5 | CAM5 (CALMODULIN 5); calcium ion binding | chr2:11532719-11533060 FORWARDmoderately similar to ( 232) CALM3_PETHY Calmodulin-related protein - Petunia hybrida (Petunia)moderately similar to ( 226) loc_os07g48780 12007.m29366 protein calmodulin, putative, expressedFRQ1 no original description | - | -2.05 |
| 30.3 | signalling.calcium | pgsc0003dmp400065611|pacid:24400679 | highly similar to ( 742) AT3G22910 | Symbols: | calcium-transporting ATPase, plasma membrane-type, putative / Ca(2+)-ATPase, putative (ACA13) | chr3:8116335-8119388 REVERSEmoderately similar to ( 473) ACA5_ORYSA Probable calcium-transporting ATPase 5, plasma membrane-type (EC 3.6.3.8) (Ca(2+)-ATPase isoform 5) - Oryza sativa (Rice)highly similar to ( 663) loc_os10g28240 12010.m05706 protein calcium-transporting ATPase 13, plasma membrane-type, putative, expressedMgtA E1-E2_ATPase PRK10517 ZntA KdpB PRK01122 zntA no original description | - | -45.19 |
| 30.3 | signalling.calcium | pgsc0003dmp400065934|pacid:24400080 | highly similar to ( 657) AT3G63380 | Symbols: | calcium-transporting ATPase, plasma membrane-type, putative / Ca(2+)-ATPase, putative (ACA12) | chr3:23407112-23410213 REVERSEmoderately similar to ( 409) ACA5_ORYSA Probable calcium-transporting ATPase 5, plasma membrane-type (EC 3.6.3.8) (Ca(2+)-ATPase isoform 5) - Oryza sativa (Rice)highly similar to ( 583) loc_os10g28240 12010.m05706 protein calcium-transporting ATPase 13, plasma membrane-type, putative, expressedMgtA PRK10517 E1-E2_ATPase ZntA no original description | - | -14.88 |
| 30.3 | signalling.calcium | pgsc0003dmp400069377|pacid:24398813 | highly similar to ( 530) AT3G22910 | Symbols: | calcium-transporting ATPase, plasma membrane-type, putative / Ca(2+)-ATPase, putative (ACA13) | chr3:8116335-8119388 REVERSEmoderately similar to ( 369) ACA5_ORYSA Probable calcium-transporting ATPase 5, plasma membrane-type (EC 3.6.3.8) (Ca(2+)-ATPase isoform 5) - Oryza sativa (Rice)moderately similar to ( 467) loc_os10g28240 12010.m05706 protein calcium-transporting ATPase 13, plasma membrane-type, putative, expressedMgtA PRK10517 ZntA no original description | - | -8.6 |
| 30.5 | signalling.G-proteins | pgsc0003dmp400000271|pacid:24420756 | moderately similar to ( 300) AT1G01200 | Symbols: ATRABA3, ATRAB-A3 | ATRABA3 (ARABIDOPSIS RAB GTPASE HOMOLOG A3); GTP binding | chr1:86715-88145 REVERSEmoderately similar to ( 280) RGP1_ORYSA Ras-related protein RGP1 (GTP-binding regulatory protein RGP1) - Oryza sativa (Rice)moderately similar to ( 280) loc_os09g10940 12009.m04357 protein ras-related protein RGP1, putative, expressedRab11_like RAB Ras Rab Rab4 Rab5_related Rab2 Rab14 Rab8_Rab10_Rab13_like Rab1_Ypt1 Rab19 Rab39 Rab18 Rab6 Rab26 Rab21 Rab30 Rab35 RabA_like Rab3 Rab15 Rab32_Rab38 Rab12 Rab7 Rab27A Rab33B_Rab33A Ras_like_GTPase Ras Rab40 Rab36_Rab34 Rab24 COG1100 Rab23_lke RJL RAS Rab28 Rab9 Ran RalA_RalB RheB Ras2 RabL2 Rho RabL4 M_R_Ras_like H_N_K_Ras_like Rap_like RSR1 RERG_RasL11_like RHO Rho4_like Rab20 Spg1 Rit_Rin_Ric RAN Rap1 Rap2 ARHI_like no original description | -4.74 | - |
| 30.5 | signalling.G-proteins | pgsc0003dmp400004431|pacid:24404212 | weakly similar to ( 171) AT1G55190 | Symbols: PRA1.F2, PRA7 | PRA7 | chr1:20588450-20589019 FORWARDweakly similar to ( 115) loc_os05g11120 12005.m05581 protein prenylated rab acceptor family protein, putative, expressedPRA1 no original description | - | -2.05 |
| 30.5 | signalling.G-proteins | pgsc0003dmp400044994|pacid:24424156 | highly similar to ( 775) AT2G23460 | Symbols: XLG1, ATXLG1 | XLG1 (EXTRA-LARGE G-PROTEIN 1); guanyl nucleotide binding / signal transducer | chr2:9995699-9998945 FORWARDweakly similar to ( 161) GPA2_SOYBN Guanine nucleotide-binding protein alpha-2 subunit (GP-alpha-2) - Glycine max (Soybean)highly similar to ( 685) loc_os12g40190 12012.m07803 protein XLG, putative, expressedG-alpha G_alpha G-alpha no original description | - | -2.81 |
| 30.5 | signalling.G-proteins | pgsc0003dmp400044995|pacid:24424158 | moderately similar to ( 360) AT2G23460 | Symbols: XLG1, ATXLG1 | XLG1 (EXTRA-LARGE G-PROTEIN 1); guanyl nucleotide binding / signal transducer | chr2:9995699-9998945 FORWARDweakly similar to ( 131) GPA1_LOTJA Guanine nucleotide-binding protein alpha-1 subunit (GP-alpha-1) - Lotus japonicusmoderately similar to ( 342) loc_os12g40190 12012.m07803 protein XLG, putative, expressedG-alpha G_alpha G-alpha no original description | - | -3.09 |
| 30.5 | signalling.G-proteins | pgsc0003dmp400052768|pacid:24395860 | weakly similar to ( 107) AT1G07620 | Symbols: | FUNCTIONS IN: molecular_function unknown; INVOLVED IN: biological_process unknown; LOCATED IN: cellular_component unknown; CONTAINS InterPro DOMAIN/s: GTP-binding protein Obg/CgtA (InterPro:IPR014100); BEST Arabidopsis thaliana protein match is: unknown protein (TAIR:AT5G02390.1); Has 447 Blast hits to 405 proteins in 72 species: Archae - 0; Bacteria - 6; Metazoa - 43; Fungi - 29; Plants - 122; Viruses - 3; Other Eukaryotes - 244 (source: NCBI BLink). | chr1:2344556-2346374 REVERSE no original description | - | -2.1 |
| 30.8 | signalling.misc | pgsc0003dmp400042880|pacid:24419627 | weakly similar to ( 119) AT4G15800 | Symbols: RALFL33 | RALFL33 (ralf-like 33); signal transducer | chr4:8984915-8985265 FORWARDvery weakly similar to (95.5) loc_os12g35670 12012.m07360 protein RALF precursor, putative, expressedRALF no original description | - | -3.44 |

**Supplemental table 12:** List of hormones related DEGs in Waneta and Atlantic after Lso treatment. (mapman analysis)

| **BinCode** | **BinName** | **id** | **description** | **Fold change AT** | **Fold Change Waneta** | **Hormone** |
| --- | --- | --- | --- | --- | --- | --- |
| 17.8.1 | hormone metabolism.salicylic acid.synthesis-degradation | pgsc0003dmp400052665|pacid:24422451 | moderately similar to ( 213) AT1G68040 | Symbols: | S-adenosyl-L-methionine:carboxyl methyltransferase family protein | chr1:25502876-25505224 FORWARDweakly similar to ( 144) BAMT_ANTMA Benzoate carboxyl methyltransferase (EC 2.1.1.-) (S-adenosyl-L-methionine:benzoic acid carboxyl methyltransferase) - Antirrhinum majus (Garden snapdragon)weakly similar to ( 137) loc_os01g50610 12001.m150693 protein jasmonate O-methyltransferase, putative, expressedMethyltransf_7 no original description | -6.4 | - | Salicylic Acid |
|  |  |  |  |  |  |  |
| 17.1.1.1.10 | hormone metabolism.abscisic acid.synthesis-degradation.synthesis.9-cis-epoxycarotenoid dioxygenase | pgsc0003dmp400007668|pacid:24392007 | weakly similar to ( 187) AT3G63520 | Symbols: CCD1, ATCCD1, ATNCED1, NCED1 | CCD1 (CAROTENOID CLEAVAGE DIOXYGENASE 1); 9-cis-epoxycarotenoid dioxygenase | chr3:23452940-23455896 FORWARDhighly similar to ( 506) loc_os08g28240 12008.m06830 protein crocetin dialdehyde, putative, expressedCOG3670 RPE65 no original description | 3.35 |  | Absicissic Acid |
| 17.1.1.1.10 | hormone metabolism.abscisic acid.synthesis-degradation.synthesis.9-cis-epoxycarotenoid dioxygenase | pgsc0003dmp400048044|pacid:24417504 | highly similar to ( 834) AT3G14440 | Symbols: NCED3, ATNCED3, STO1, SIS7 | NCED3 (NINE-CIS-EPOXYCAROTENOID DIOXYGENASE 3); 9-cis-epoxycarotenoid dioxygenase | chr3:4831678-4833477 REVERSEhighly similar to ( 756) loc_os03g44380 12003.m09477 protein 9-cis-epoxycarotenoid dioxygenase 2, putative, expressedRPE65 COG3670 no original description | - | -7.36 | Absicissic Acid |
| 17.1.3 | hormone metabolism.abscisic acid.induced-regulated-responsive-activated | pgsc0003dmp400008459|pacid:24391220 | moderately similar to ( 315) AT5G13200 | Symbols: | GRAM domain-containing protein / ABA-responsive protein-related | chr5:4207081-4208079 FORWARDmoderately similar to ( 260) loc_os12g29400 12012.m06754 protein ABA-responsive protein, putative, expressed no original description | - | -9.13 | Absicissic Acid |
| 17.1.3 | hormone metabolism.abscisic acid.induced-regulated-responsive-activated | pgsc0003dmp400008460|pacid:24391221 | moderately similar to ( 232) AT5G13200 | Symbols: | GRAM domain-containing protein / ABA-responsive protein-related | chr5:4207081-4208079 FORWARDweakly similar to ( 191) loc_os12g29400 12012.m06754 protein ABA-responsive protein, putative, expressed no original description | - | -13.75 | Absicissic Acid |
| 17.1.3 | hormone metabolism.abscisic acid.induced-regulated-responsive-activated | pgsc0003dmp400018009|pacid:24403865 | weakly similar to ( 164) AT5G08350 | Symbols: | GRAM domain-containing protein / ABA-responsive protein-related | chr5:2686417-2687175 REVERSEweakly similar to ( 175) loc_os02g42440 12002.m09279 protein FIP1, putative, expressed no original description | - | -6.08 | Absicissic Acid |
|  |  |  |  |  |  |  |
| 17.2.3 | hormone metabolism.auxin.induced-regulated-responsive-activated | pgsc0003dmp400000279|pacid:24423880 | weakly similar to ( 112) AT3G61900 | Symbols: | auxin-responsive family protein | chr3:22925813-22926379 FORWARDAuxin_inducible no original description | - | 2.09 | Auxin |
| 17.2.3 | hormone metabolism.auxin.induced-regulated-responsive-activated | pgsc0003dmp400002928|pacid:24422600 | weakly similar to ( 164) AT4G34760 | Symbols: | auxin-responsive family protein | chr4:16582471-16582794 REVERSEvery weakly similar to (86.3) AX15A_SOYBN Auxin-induced protein 15A - Glycine max (Soybean)weakly similar to ( 107) loc_os02g24700 12002.m07617 protein OsSAUR8 - Auxin-responsive SAUR gene family member, expressedAuxin_inducible no original description | - | 3.43 | Auxin |
| 17.2.3 | hormone metabolism.auxin.induced-regulated-responsive-activated | pgsc0003dmp400002990|pacid:24421610 | weakly similar to ( 146) AT2G16580 | Symbols: | auxin-responsive protein, putative | chr2:7186602-7186928 REVERSEvery weakly similar to (85.5) ARG7_PHAAU Indole-3-acetic acid-induced protein ARG7 - Phaseolus aureus (Mung bean) (Vigna radiata)very weakly similar to (99.8) loc_os02g24700 12002.m07617 protein OsSAUR8 - Auxin-responsive SAUR gene family member, expressedAuxin_inducible no original description | - | 2.49 | Auxin |
| 17.2.3 | hormone metabolism.auxin.induced-regulated-responsive-activated | pgsc0003dmp400002991|pacid:24422117 | weakly similar to ( 170) AT1G75580 | Symbols: | auxin-responsive protein, putative | chr1:28377530-28377856 FORWARDvery weakly similar to (88.2) AX15A_SOYBN Auxin-induced protein 15A - Glycine max (Soybean)weakly similar to ( 110) loc_os02g24700 12002.m07617 protein OsSAUR8 - Auxin-responsive SAUR gene family member, expressedAuxin_inducible no original description | - | 3.73 | Auxin |
| 17.2.3 | hormone metabolism.auxin.induced-regulated-responsive-activated | pgsc0003dmp400005820|pacid:24404106 | very weakly similar to ( 100) AT4G00880 | Symbols: | auxin-responsive family protein | chr4:366692-367060 REVERSEAuxin_inducible no original description | - | -14.63 | Auxin |
| 17.2.3 | hormone metabolism.auxin.induced-regulated-responsive-activated | pgsc0003dmp400018589|pacid:24411673 | highly similar to ( 677) AT2G44500 | Symbols: | unknown protein | chr2:18374447-18376435 FORWARDmoderately similar to ( 266) loc_os04g47520 12004.m35417 protein auxin-independent growth promoter, putative, expressedDUF246 no original description | - | -17.25 | Auxin |
| 17.2.3 | hormone metabolism.auxin.induced-regulated-responsive-activated | pgsc0003dmp400022705|pacid:24403461 | moderately similar to ( 333) AT4G27450 | Symbols: | unknown protein | chr4:13727665-13728683 REVERSEweakly similar to ( 123) TSJT1_TOBAC Stem-specific protein TSJT1 - Nicotiana tabacum (Common tobacco)moderately similar to ( 223) loc_os03g53270 12003.m35408 protein stem-specific protein TSJT1, putative, expressedWali7 Gn_AT_II no original description | -27.4 | -8.8 | Auxin |
| 17.2.3 | hormone metabolism.auxin.induced-regulated-responsive-activated | pgsc0003dmp400036081|pacid:24389934 | moderately similar to ( 395) AT5G47530 | Symbols: | auxin-responsive protein, putative | chr5:19281471-19282870 FORWARDmoderately similar to ( 328) loc_os03g09900 12003.m06444 protein membrane protein, putative, expressedDUF568 B561 no original description | - | -5.97 | Auxin |
| 17.2.3 | hormone metabolism.auxin.induced-regulated-responsive-activated | pgsc0003dmp400042616|pacid:24406063 | highly similar to ( 759) AT5G15740 | Symbols: | unknown protein | chr5:5134788-5136956 REVERSEhighly similar to ( 787) loc_os06g17390 12006.m06451 protein auxin-independent growth promoter, putative, expressedDUF246 no original description | -4.17 | - | Auxin |
| 17.2.3 | hormone metabolism.auxin.induced-regulated-responsive-activated | pgsc0003dmp400045440|pacid:24416500 | highly similar to ( 928) AT5G54510 | Symbols: GH3.6, DFL1 | DFL1 (DWARF IN LIGHT 1); indole-3-acetic acid amido synthetase | chr5:22131321-22133564 REVERSEhighly similar to ( 826) GH31_ORYSA Probable indole-3-acetic acid-amido synthetase GH3.1 (EC 6.3.2.-) (Auxin-responsive GH3-like protein 1) (OsGH3-1) - Oryza sativa (Rice)highly similar to ( 826) loc_os01g57610 12001.m11911 protein indole-3-acetic acid-amido synthetase GH3.1, putative, expressedGH3 no original description | - | 6.75 | Auxin |
| 17.2.3 | hormone metabolism.auxin.induced-regulated-responsive-activated | pgsc0003dmp400046065|pacid:24395350 | highly similar to ( 523) AT1G51630 | Symbols: | unknown protein | chr1:19142141-19144082 REVERSEmoderately similar to ( 437) loc_os09g32320 12009.m06320 protein expressed proteinDUF246 no original description | -4.57 | - | Auxin |
| 17.2.3 | hormone metabolism.auxin.induced-regulated-responsive-activated | pgsc0003dmp400048995|pacid:24410640 | no original description | - | 5.76 | Auxin |
| 17.2.3 | hormone metabolism.auxin.induced-regulated-responsive-activated | pgsc0003dmp400052632|pacid:24394996 | weakly similar to ( 116) AT2G46690 | Symbols: | auxin-responsive family protein | chr2:19180904-19181269 FORWARDAuxin_inducible no original description | - | -2.41 | Auxin |
|  |  |  |  |  |  |  |
| 17.3.1.2.2 | hormone metabolism.brassinosteroid.synthesis-degradation.sterols.SMT2 | pgsc0003dmp400032477|pacid:24421019 | highly similar to ( 624) AT1G20330 | Symbols: SMT2, CVP1, FRL1 | SMT2 (STEROL METHYLTRANSFERASE 2); S-adenosylmethionine-dependent methyltransferase | chr1:7038968-7040053 REVERSEhighly similar to ( 549) SMT2_ORYSA 24-methylenesterol C-methyltransferase 2 (EC 2.1.1.143) (24-sterol C-methyltransferase 2) (Sterol-C-methyltransferase 2) - Oryza sativa (Rice)highly similar to ( 549) loc_os03g04340 12003.m05964 protein 24-methylenesterol C-methyltransferase 2, putative, expressedSterol_MT_C Methyltransf_11 UbiE ubiE PRK08317 CMAS no original description | - | 1.67 | Brassinosteroids |
| 17.3.1.2.4 | hormone metabolism.brassinosteroid.synthesis-degradation.sterols.FACKEL | pgsc0003dmp400004888|pacid:24398207 | highly similar to ( 513) AT3G52940 | Symbols: FK, HYD2, ELL1 | FK (FACKEL); delta14-sterol reductase | chr3:19631030-19633112 REVERSEmoderately similar to ( 493) loc_os09g39220 12009.m06861 protein delta(14)-sterol reductase, putative, expressedERG4_ERG24 no original description | -4.68 | - | Brassinosteroids |
| 17.3.1.2.6 | hormone metabolism.brassinosteroid.synthesis-degradation.sterols.DWF7 | pgsc0003dmp400045832|pacid:24429130 | moderately similar to ( 411) AT3G02580 | Symbols: STE1, DWF7, BUL1 | STE1 (STEROL 1); C-5 sterol desaturase | chr3:547048-548615 FORWARDmoderately similar to ( 495) SC5D_TOBAC Delta(7)-sterol-C5(6)-desaturase (EC 1.3.3.-) (Delta-7-C-5 sterol desaturase) (Delta(7)-sterol-C5-desaturase) - Nicotiana tabacum (Common tobacco)moderately similar to ( 391) loc_os01g04260 12001.m07061 protein delta-7-sterol-C5, putative, expressed no original description | -17.28 | -2.36 | Brassinosteroids |
| 17.3.1.2.7 | hormone metabolism.brassinosteroid.synthesis-degradation.sterols.DWF5 | pgsc0003dmp400010516|pacid:24396577 | highly similar to ( 682) AT1G50430 | Symbols: DWF5, PA, LE, ST7R, 7RED | DWF5 (DWARF 5); sterol delta7 reductase | chr1:18682175-18685555 REVERSEhighly similar to ( 647) loc_os02g26650 12002.m34011 protein 7-dehydrocholesterol reductase, putative, expressedERG4_ERG24 no original description | -8.31 | -1.76 | Brassinosteroids |
| 17.3.2.2 | hormone metabolism.brassinosteroid.signal transduction.BZR | pgsc0003dmp400007972|pacid:24424773 | moderately similar to ( 226) AT1G19350 | Symbols: BES1, BZR2 | BES1 (BRI1-EMS-SUPPRESSOR 1); protein binding / transcription factor/ transcription regulator | chr1:6688841-6690165 FORWARDmoderately similar to ( 215) loc_os07g39220 12007.m08164 protein BES1/BZR1 homolog protein 1, putative, expressedDUF822 no original description | - | -1.88 | Brassinosteroids |
| 17.3.2.2 | hormone metabolism.brassinosteroid.signal transduction.BZR | pgsc0003dmp400027023|pacid:24379861 | moderately similar to ( 267) AT1G75080 | Symbols: BZR1 | BZR1 (BRASSINAZOLE-RESISTANT 1); DNA binding / transcription regulator/ transcription repressor | chr1:28185709-28187063 FORWARDweakly similar to ( 184) loc_os07g39220 12007.m08164 protein BES1/BZR1 homolog protein 1, putative, expressedDUF822 no original description | - | -1.73 | Brassinosteroids |
| 17.3.2.2 | hormone metabolism.brassinosteroid.signal transduction.BZR | pgsc0003dmp400049547|pacid:24428730 | moderately similar to ( 274) AT1G78700 | Symbols: | brassinosteroid signalling positive regulator-related | chr1:29599854-29601539 FORWARDmoderately similar to ( 229) loc_os06g35900 12006.m08127 protein brassinazole-resistant 1 protein, putative, expressedDUF822 no original description | - | -2.11 | Brassinosteroids |
|  |  |  |  |  |  |  |
| 17.5.1.1 | hormone metabolism.ethylene.synthesis-degradation.1-aminocyclopropane-1-carboxylate synthase | pgsc0003dmp400010333|pacid:24392946 | highly similar to ( 660) AT4G11280 | Symbols: ACS6 | ACS6 (1-AMINOCYCLOPROPANE-1-CARBOXYLIC ACID (ACC) SYNTHASE 6); 1-aminocyclopropane-1-carboxylate synthase | chr4:6864168-6865922 FORWARDhighly similar to ( 688) 1A1C_SOYBN 1-aminocyclopropane-1-carboxylate synthase (EC 4.4.1.14) (ACC synthase) (S-adenosyl-L-methionine methylthioadenosine-lyase) - Glycine max (Soybean)highly similar to ( 597) loc_os04g48850 12004.m09820 protein 1-aminocyclopropane-1-carboxylate synthase, putative, expressedAminotran_1_2 COG0436 PRK06056 PRK06108 PRK07683 ARO8 PRK06836 PRK05794 PRK07309 PRK08069 PRK08068 PRK07310 PRK08443 PRK09276 PRK08960 PRK08912 PRK07367 PRK07682 HisC PRK07681 PRK05942 PRK07568 PRK07778 PRK08363 PRK08175 PRK05764 PRK07337 PRK06575 PRK06507 PRK08362 PRK06220 PRK07212 PRK08361 PRK07550 PRK07777 MalY PRK06107 PRK07366 no original description | - | -3.8 | Ethylene |
| 17.5.1 | hormone metabolism.ethylene.synthesis-degradation | pgsc0003dmp400015865|pacid:24420861 | moderately similar to ( 342) AT1G02400 | Symbols: ATGA2OX4, ATGA2OX6, DTA1, GA2OX6 | GA2OX6 (GIBBERELLIN 2-OXIDASE 6); gibberellin 2-beta-dioxygenase | chr1:486964-489391 FORWARDmoderately similar to ( 395) G2OX2_PEA Gibberellin 2-beta-dioxygenase 2 (EC 1.14.11.13) (Gibberellin 2-beta-hydroxylase 2) (Gibberellin 2-oxidase 2) (GA 2-oxidase 2) - Pisum sativum (Garden pea)moderately similar to ( 261) loc_os05g06670 12005.m05194 protein gibberellin 2-oxidase, putative, expressedPcbC 2OG-FeII_Oxy no original description | - | -15.79 | Ethylene |
| 17.5.2 | hormone metabolism.ethylene.signal transduction | pgsc0003dmp400018962|pacid:24426934 | weakly similar to ( 108) AT3G23240 | Symbols: ERF1, ATERF1 | ERF1 (ETHYLENE RESPONSE FACTOR 1); DNA binding / transcription activator/ transcription factor | chr3:8295705-8296361 FORWARDvery weakly similar to (95.5) ERF2_TOBAC Ethylene-responsive transcription factor 2 (Ethylene-responsive element-binding factor 2) (EREBP-2) (NtERF2) - Nicotiana tabacum (Common tobacco)weakly similar to ( 102) loc_os01g54890 12001.m11650 protein ethylene-responsive transcription factor 2, putative, expressedAP2 AP2 no original description | 3.06 | -2.57 | Ethylene |
| 17.5.2 | hormone metabolism.ethylene.signal transduction | pgsc0003dmp400019030|pacid:24379854 | weakly similar to ( 179) AT4G17500 | Symbols: ATERF-1 | ATERF-1 (ETHYLENE RESPONSIVE ELEMENT BINDING FACTOR 1); DNA binding / transcription activator/ transcription factor | chr4:9759405-9760211 FORWARDmoderately similar to ( 294) ERF2_NICSY Ethylene-responsive transcription factor 2 (Ethylene-responsive element-binding factor 2) (EREBP-2) (NsERF2) - Nicotiana sylvestris (Wood tobacco)weakly similar to ( 142) loc_os02g43790 12002.m09413 protein ethylene responsive protein, putative, expressedAP2 AP2 no original description | -2.07 |  | Ethylene |
| 17.5.2 | hormone metabolism.ethylene.signal transduction | pgsc0003dmp400019035|pacid:24379598 | weakly similar to ( 128) AT5G51190 | Symbols: | AP2 domain-containing transcription factor, putative | chr5:20800708-20801373 REVERSEmoderately similar to ( 245) ERF5_TOBAC Ethylene-responsive transcription factor 5 (Ethylene-responsive element-binding factor 5 homolog) (EREBP-4) (NtERF4) - Nicotiana tabacum (Common tobacco)very weakly similar to (97.8) loc_os10g41330 12010.m06897 protein ethylene-responsive transcription factor 5, putative, expressedAP2 AP2 no original description | 3.29 | -11.46 | Ethylene |
| 17.5.1 | hormone metabolism.ethylene.synthesis-degradation | pgsc0003dmp400020834|pacid:24417285 | weakly similar to ( 189) AT5G24530 | Symbols: DMR6 | DMR6 (DOWNY MILDEW RESISTANT 6); oxidoreductase/ oxidoreductase, acting on paired donors, with incorporation or reduction of molecular oxygen, 2-oxoglutarate as one donor, and incorporation of one atom each of oxygen into both donors | chr5:8378964-8383154 FORWARDweakly similar to ( 145) FL3H_MALDO Naringenin,2-oxoglutarate 3-dioxygenase (EC 1.14.11.9) (Flavonone-3-hydroxylase) (F3H) (FHT) - Malus domestica (Apple) (Malus sylvestris)weakly similar to ( 184) loc_os07g07410 12007.m05200 protein hyoscyamine 6-dioxygenase, putative, expressedPcbC 2OG-FeII_Oxy no original description | -62.27 | -8.24 | Ethylene |
| 17.5.2 | hormone metabolism.ethylene.signal transduction | pgsc0003dmp400024296|pacid:24377921 | weakly similar to ( 105) AT5G44210 | Symbols: ERF9, ATERF9, ATERF-9 | ERF9 (ERF DOMAIN PROTEIN 9); DNA binding / transcription factor/ transcription repressor | chr5:17806742-17807344 FORWARDweakly similar to ( 128) ERF4_TOBAC Ethylene-responsive transcription factor 4 (Ethylene-responsive element-binding factor 4 homolog) (EREBP-3) (NtERF3) - Nicotiana tabacum (Common tobacco)very weakly similar to (87.8) loc_os01g58420 12001.m11985 protein ethylene-responsive transcription factor 4, putative, expressedAP2 AP2 no original description | - | -10.07 | Ethylene |
| 17.5.2 | hormone metabolism.ethylene.signal transduction | pgsc0003dmp400025392|pacid:24410945 | weakly similar to ( 125) AT1G50640 | Symbols: ERF3, ATERF3 | ERF3 (ETHYLENE RESPONSIVE ELEMENT BINDING FACTOR 3); DNA binding / protein binding / transcription factor/ transcription repressor | chr1:18757602-18758279 REVERSEweakly similar to ( 169) ERF3_TOBAC Ethylene-responsive transcription factor 3 (Ethylene-responsive element-binding factor 3 homolog) (EREBP-5) (NtERF5) - Nicotiana tabacum (Common tobacco)AP2 AP2 no original description | - | -4.36 | Ethylene |
| 17.5.2 | hormone metabolism.ethylene.signal transduction | pgsc0003dmp400028007|pacid:24386784 | weakly similar to ( 110) AT5G47230 | Symbols: ERF5, ATERF-5, ATERF5 | ERF5 (ETHYLENE RESPONSIVE ELEMENT BINDING FACTOR 5); DNA binding / transcription activator/ transcription factor | chr5:19180072-19180974 FORWARDweakly similar to ( 187) ERF5_NICSY Ethylene-responsive transcription factor 5 (Ethylene-responsive element-binding factor 5 homolog) (EREBP-4) (NsERF4) - Nicotiana sylvestris (Wood tobacco)very weakly similar to (89.7) loc_os10g41330 12010.m06897 protein ethylene-responsive transcription factor 5, putative, expressedAP2 AP2 no original description | - | -7.66 | Ethylene |
| 17.5.2 | hormone metabolism.ethylene.signal transduction | pgsc0003dmp400028008|pacid:24389428 | weakly similar to ( 149) AT5G07580 | Symbols: | DNA binding / transcription factor | chr5:2399525-2400349 FORWARDweakly similar to ( 119) ERF5_TOBAC Ethylene-responsive transcription factor 5 (Ethylene-responsive element-binding factor 5 homolog) (EREBP-4) (NtERF4) - Nicotiana tabacum (Common tobacco)weakly similar to ( 116) loc_os02g43820 12002.m09416 protein AP2 domain containing protein, expressedAP2 AP2 no original description | - | -1.91 | Ethylene |
| 17.5.2 | hormone metabolism.ethylene.signal transduction | pgsc0003dmp400028010|pacid:24388261 | weakly similar to ( 173) AT4G17500 | Symbols: ATERF-1 | ATERF-1 (ETHYLENE RESPONSIVE ELEMENT BINDING FACTOR 1); DNA binding / transcription activator/ transcription factor | chr4:9759405-9760211 FORWARDmoderately similar to ( 290) ERF1_TOBAC Ethylene-responsive transcription factor 1 (Ethylene-responsive element-binding factor 1) (EREBP-1) (NtERF1) - Nicotiana tabacum (Common tobacco)weakly similar to ( 125) loc_os02g43790 12002.m09413 protein ethylene responsive protein, putative, expressedAP2 AP2 no original description | - | -3.52 | Ethylene |
| 17.5.1 | hormone metabolism.ethylene.synthesis-degradation | pgsc0003dmp400030018|pacid:24398438 | weakly similar to ( 105) AT1G06620 | Symbols: | 2-oxoglutarate-dependent dioxygenase, putative | chr1:2025618-2027094 FORWARDvery weakly similar to (93.6) DV4H_CATRO Desacetoxyvindoline 4-hydroxylase (EC 1.14.11.20) - Catharanthus roseus (Rosy periwinkle) (Madagascar periwinkle)very weakly similar to (92.4) loc_os03g48430 12003.m09851 protein 1-aminocyclopropane-1-carboxylate oxidase, putative, expressed no original description | - | -3.31 | Ethylene |
| 17.5.1 | hormone metabolism.ethylene.synthesis-degradation | pgsc0003dmp400036556|pacid:24428177 | moderately similar to ( 390) AT1G30040 | Symbols: ATGA2OX2 | ATGA2OX2 (GIBBERELLIN 2-OXIDASE); gibberellin 2-beta-dioxygenase | chr1:10537769-10538781 FORWARDmoderately similar to ( 410) G2OX1_PEA Gibberellin 2-beta-dioxygenase 1 (EC 1.14.11.13) (Gibberellin 2-beta-hydroxylase 1) (Gibberellin 2-oxidase 1) (GA 2-oxidase 1) (Protein SLENDER) - Pisum sativum (Garden pea)moderately similar to ( 352) loc_os01g55240 12001.m11683 protein gibberellin 2-beta-dioxygenase, putative, expressedPcbC 2OG-FeII_Oxy no original description | - | 2.91 | Ethylene |
| 17.5.2 | hormone metabolism.ethylene.signal transduction | pgsc0003dmp400039575|pacid:24411485 | very weakly similar to (99.8) AT3G15210 | Symbols: ATERF-4, ERF4, RAP2.5, ATERF4 | ERF4 (ETHYLENE RESPONSIVE ELEMENT BINDING FACTOR 4); DNA binding / protein binding / transcription factor/ transcription repressor | chr3:5121472-5122140 FORWARDvery weakly similar to ( 100) ERF4_NICSY Ethylene-responsive transcription factor 4 (Ethylene-responsive element-binding factor 4 homolog) (EREBP-3) (NsERF3) - Nicotiana sylvestris (Wood tobacco)very weakly similar to (88.2) loc_os05g41780 12005.m08352 protein ethylene-responsive transcription factor 4, putative, expressedAP2 AP2 no original description | - | -3.39 | Ethylene |
| 17.5.2 | hormone metabolism.ethylene.signal transduction | pgsc0003dmp400045528|pacid:24393301 | weakly similar to ( 131) AT5G47230 | Symbols: ERF5, ATERF-5, ATERF5 | ERF5 (ETHYLENE RESPONSIVE ELEMENT BINDING FACTOR 5); DNA binding / transcription activator/ transcription factor | chr5:19180072-19180974 FORWARDweakly similar to ( 119) ERF5_TOBAC Ethylene-responsive transcription factor 5 (Ethylene-responsive element-binding factor 5 homolog) (EREBP-4) (NtERF4) - Nicotiana tabacum (Common tobacco)weakly similar to ( 103) loc_os02g43820 12002.m09416 protein AP2 domain containing protein, expressedAP2 AP2 no original description | - | -6.98 | Ethylene |
| 17.5.2 | hormone metabolism.ethylene.signal transduction | pgsc0003dmp400045576|pacid:24391562 | weakly similar to ( 148) AT5G47220 | Symbols: ATERF2, ATERF-2, ERF2 | ERF2 (ETHYLENE RESPONSIVE ELEMENT BINDING FACTOR 2); DNA binding / transcription activator/ transcription factor | chr5:19172023-19172754 REVERSEweakly similar to ( 126) ERF2_TOBAC Ethylene-responsive transcription factor 2 (Ethylene-responsive element-binding factor 2) (EREBP-2) (NtERF2) - Nicotiana tabacum (Common tobacco)weakly similar to ( 103) loc_os04g46220 12004.m09573 protein ethylene-responsive transcription factor 2, putative, expressedAP2 AP2 no original description | - | -5.19 | Ethylene |
| 17.5.1 | hormone metabolism.ethylene.synthesis-degradation | pgsc0003dmp400047526|pacid:24410129 | moderately similar to ( 431) AT2G36690 | Symbols: | oxidoreductase, 2OG-Fe(II) oxygenase family protein | chr2:15379930-15381987 FORWARDweakly similar to ( 176) FL3H_DIACA Naringenin,2-oxoglutarate 3-dioxygenase (EC 1.14.11.9) (Flavonone-3-hydroxylase) (F3H) (FHT) - Dianthus caryophyllus (Carnation) (Clove pink)moderately similar to ( 354) loc_os08g44590 12008.m08432 protein gibberellin 20 oxidase 2, putative, expressedPcbC 2OG-FeII_Oxy no original description | - | 2.32 | Ethylene |
| 17.5.1 | hormone metabolism.ethylene.synthesis-degradation | pgsc0003dmp400050565|pacid:24409420 | moderately similar to ( 229) AT3G13610 | Symbols: | oxidoreductase, 2OG-Fe(II) oxygenase family protein | chr3:4449448-4450616 FORWARDvery weakly similar to (84.7) LDOX_MALDO Leucoanthocyanidin dioxygenase (EC 1.14.11.19) (LDOX) (Leucocyanidin oxygenase) (Leucoanthocyanidin hydroxylase) (Anthocyanidin synthase) - Malus domestica (Apple) (Malus sylvestris)weakly similar to ( 104) loc_os01g61610 12001.m43254 protein gibberellin 20 oxidase 2, putative, expressed no original description | - | -2.99 | Ethylene |
| 17.5.2 | hormone metabolism.ethylene.signal transduction | pgsc0003dmp400058670|pacid:24378384 | weakly similar to ( 137) AT5G51190 | Symbols: | AP2 domain-containing transcription factor, putative | chr5:20800708-20801373 REVERSEmoderately similar to ( 239) ERF5_TOBAC Ethylene-responsive transcription factor 5 (Ethylene-responsive element-binding factor 5 homolog) (EREBP-4) (NtERF4) - Nicotiana tabacum (Common tobacco)weakly similar to ( 107) loc_os10g41330 12010.m06897 protein ethylene-responsive transcription factor 5, putative, expressedAP2 AP2 no original description | 4.09 | -12.27 | Ethylene |
| 17.5.2 | hormone metabolism.ethylene.signal transduction | pgsc0003dmp400062150|pacid:24378835 | weakly similar to ( 146) AT5G51190 | Symbols: | AP2 domain-containing transcription factor, putative | chr5:20800708-20801373 REVERSEmoderately similar to ( 243) ERF5_TOBAC Ethylene-responsive transcription factor 5 (Ethylene-responsive element-binding factor 5 homolog) (EREBP-4) (NtERF4) - Nicotiana tabacum (Common tobacco)very weakly similar to ( 100) loc_os10g41330 12010.m06897 protein ethylene-responsive transcription factor 5, putative, expressedAP2 AP2 no original description | 3.84 | -9.08 | Ethylene |
|  |  |  |  |  |  |  |
| 17.7.1.2 | hormone metabolism.jasmonate.synthesis-degradation.lipoxygenase | pgsc0003dmp400039701|pacid:24404158 | nearly identical (1311) AT1G17420 | Symbols: LOX3 | LOX3; electron carrier/ iron ion binding / lipoxygenase/ metal ion binding / oxidoreductase, acting on single donors with incorporation of molecular oxygen, incorporation of two atoms of oxygen | chr1:5977512-5981384 FORWARDnearly identical (1151) LOX6_ORYSA Probable lipoxygenase 6 (EC 1.13.11.12) - Oryza sativa (Rice)nearly identical (1151) loc_os03g08220 12003.m06331 protein lipoxygenase 6, putative, expressedLipoxygenase PLAT_LH2 LH2 no original description | - | -7.62 | Jasmonic Acid |
| 17.7.1.2 | hormone metabolism.jasmonate.synthesis-degradation.lipoxygenase | pgsc0003dmp400019188|pacid:24392793 | nearly identical (1249) AT1G55020 | Symbols: LOX1 | LOX1; lipoxygenase | chr1:20525798-20530143 FORWARDnearly identical (1426) LOX1_SOLTU Lipoxygenase 1 (EC 1.13.11.12) - Solanum tuberosum (Potato)nearly identical (1091) loc_os03g49380 12003.m35371 protein lipoxygenase 4, putative, expressedLipoxygenase PLAT_LH2 LH2 PLAT no original description | - | -3.4 | Jasmonic Acid |

**Supplemental table 13:** List of Transcription factors DEGs in Waneta and Atlantic after Lso treatment. (mapman analysis)

| **BinCode** | **BinName** | **id** | **description** | **Fold Change AT** | **fold change WN** |  |
| --- | --- | --- | --- | --- | --- | --- |
| 27.3.25 | RNA.regulation of transcription.MYB domain transcription factor family | pgsc0003dmp400002398|pacid:24427940 | moderately similar to ( 298) AT3G01140 | Symbols: MYB106, AtMYB106 | MYB106 (myb domain protein 106); DNA binding / transcription factor | chr3:46619-48143 REVERSEmoderately similar to ( 204) MYB38_MAIZE Myb-related protein Zm38 - Zea mays (Maize)moderately similar to ( 284) loc_os08g33660 12008.m07356 protein MYB1, putative, expressed no original description | - | 2.79 | MYB |
| 27.3.25 | RNA.regulation of transcription.MYB domain transcription factor family | pgsc0003dmp400010956|pacid:24418204 | moderately similar to ( 306) AT4G38620 | Symbols: ATMYB4, MYB4 | MYB4; DNA binding / transcription factor | chr4:18053866-18054876 FORWARDmoderately similar to ( 292) MYB1_HORVU Myb-related protein Hv1 - Hordeum vulgare (Barley)moderately similar to ( 306) loc_os09g36730 12009.m06622 protein myb-related protein Hv1, putative, expressed no original description | -3.82 | - | MYB |
| 27.3.25 | RNA.regulation of transcription.MYB domain transcription factor family | pgsc0003dmp400014055|pacid:24382781 | weakly similar to ( 176) AT3G50060 | Symbols: MYB77 | MYB77; DNA binding / transcription factor | chr3:18558146-18559051 REVERSEweakly similar to ( 110) MYBA1_ORYSA Myb-related protein MYBAS1 - Oryza sativa (Rice)weakly similar to ( 178) loc_os09g01960 12009.m03570 protein sucrose responsive element binding protein, putative, expressedREB1 no original description | - | -14.82 | MYB |
| 27.3.25 | RNA.regulation of transcription.MYB domain transcription factor family | pgsc0003dmp400014627|pacid:24405106 | weakly similar to ( 151) AT3G23250 | Symbols: MYB15, ATY19, ATMYB15 | MYB15 (MYB DOMAIN PROTEIN 15); DNA binding / transcription factor | chr3:8309742-8310624 FORWARDweakly similar to ( 148) MYB4_ORYSA Myb-related protein Myb4 (OsMyb4) (Transcription factor RLTR1) - Oryza sativa (Rice)weakly similar to ( 154) loc_os07g37210 12007.m07971 protein MYB transcription factor, putative, expressed no original description | - | -3.6 | MYB |
| 27.3.25 | RNA.regulation of transcription.MYB domain transcription factor family | pgsc0003dmp400021429|pacid:24393250 | moderately similar to ( 201) AT3G13540 | Symbols: ATMYB5 | ATMYB5 (MYB DOMAIN PROTEIN 5); DNA binding / transcription factor | chr3:4420239-4421443 FORWARDmoderately similar to ( 203) MYB1_HORVU Myb-related protein Hv1 - Hordeum vulgare (Barley)moderately similar to ( 204) loc_os07g37210 12007.m07971 protein MYB transcription factor, putative, expressed no original description | -13.62 | - | MYB |
| 27.3.25 | RNA.regulation of transcription.MYB domain transcription factor family | pgsc0003dmp400023726|pacid:24404079 | moderately similar to ( 238) AT3G23250 | Symbols: MYB15, ATY19, ATMYB15 | MYB15 (MYB DOMAIN PROTEIN 15); DNA binding / transcription factor | chr3:8309742-8310624 FORWARDmoderately similar to ( 226) MYB4_ORYSA Myb-related protein Myb4 (OsMyb4) (Transcription factor RLTR1) - Oryza sativa (Rice)moderately similar to ( 226) loc_os04g43680 12004.m09327 protein myb-related protein Myb4, putative, expressed no original description | - | -2.91 | MYB |
| 27.3.25 | RNA.regulation of transcription.MYB domain transcription factor family | pgsc0003dmp400027453|pacid:24410172 | moderately similar to ( 266) AT5G16600 | Symbols: MYB43, AtMYB43 | MYB43 (myb domain protein 43); DNA binding / transcription factor | chr5:5438291-5440214 FORWARDmoderately similar to ( 245) ODO1_PETHY ODORANT1 protein (MYB-like protein ODO1) - Petunia hybrida (Petunia)moderately similar to ( 266) loc_os09g23620 12009.m05519 protein odorant 1 protein, putative, expressed no original description | - | -15.01 | MYB |
| 27.3.25 | RNA.regulation of transcription.MYB domain transcription factor family | pgsc0003dmp400027651|pacid:24413450 | moderately similar to ( 257) AT1G09540 | Symbols: MYB61, ATMYB61 | MYB61 (MYB DOMAIN PROTEIN 61); DNA binding / transcription factor | chr1:3086333-3087689 FORWARDmoderately similar to ( 219) MYB3_HORVU Myb-related protein Hv33 - Hordeum vulgare (Barley)moderately similar to ( 271) loc_os05g04820 12005.m05013 protein MYB2, putative, expressed no original description | - | 10.51 | MYB |
| 27.3.25 | RNA.regulation of transcription.MYB domain transcription factor family | pgsc0003dmp400030100|pacid:24399030 | moderately similar to ( 251) AT3G23250 | Symbols: MYB15, ATY19, ATMYB15 | MYB15 (MYB DOMAIN PROTEIN 15); DNA binding / transcription factor | chr3:8309742-8310624 FORWARDmoderately similar to ( 245) MYB4_ORYSA Myb-related protein Myb4 (OsMyb4) (Transcription factor RLTR1) - Oryza sativa (Rice)moderately similar to ( 245) loc_os04g43680 12004.m09327 protein myb-related protein Myb4, putative, expressed no original description | - | -2.16 | MYB |
| 27.3.25 | RNA.regulation of transcription.MYB domain transcription factor family | pgsc0003dmp400031557|pacid:24405742 | moderately similar to ( 225) AT4G01680 | Symbols: MYB55 | MYB55 (myb domain protein 55); DNA binding / transcription factor | chr4:716401-717415 REVERSEmoderately similar to ( 211) MYB3_HORVU Myb-related protein Hv33 - Hordeum vulgare (Barley)moderately similar to ( 229) loc_os05g04820 12005.m05013 protein MYB2, putative, expressed no original description | - | 2.67 | MYB |
| 27.3.26 | RNA.regulation of transcription.MYB-related transcription factor family | pgsc0003dmp400009906|pacid:24405982 | moderately similar to ( 216) AT1G49010 | Symbols: | myb family transcription factor | chr1:18132714-18133778 FORWARDweakly similar to ( 194) loc_os04g58020 12004.m10684 protein DNA binding protein, putative, expressed no original description | - | 2.02 | MYB |
| 27.3.26 | RNA.regulation of transcription.MYB-related transcription factor family | pgsc0003dmp400001422|pacid:24421729 | weakly similar to ( 103) AT1G75250 | Symbols: ATRL6 | ATRL6 (ARABIDOPSIS RAD-LIKE 6); transcription factor | chr1:28244463-28245453 REVERSEvery weakly similar to (94.0) loc_os05g50340 12005.m09103 protein RADIALIS, putative, expressed no original description | - | -2.17 | MYB |
| 27.3.26 | RNA.regulation of transcription.MYB-related transcription factor family | pgsc0003dmp400056302|pacid:24411419 | weakly similar to ( 102) AT4G39250 | Symbols: ATRL1 | ATRL1 (ARABIDOPSIS RAD-LIKE 1); DNA binding / transcription factor | chr4:18271457-18271857 REVERSEvery weakly similar to (86.3) loc_os05g50340 12005.m09103 protein RADIALIS, putative, expressed no original description | - | -3.61 | MYB |
|  |  |  |  |  |  |  |
| 27.3.35 | RNA.regulation of transcription.bZIP transcription factor family | pgsc0003dmp400002986|pacid:24420431 | moderately similar to ( 328) AT4G38900 | Symbols: | bZIP protein | chr4:18139564-18141520 REVERSEweakly similar to ( 125) RF2A_ORYSA Transcription factor RF2a - Oryza sativa (Rice)moderately similar to ( 233) loc_os03g03550 12003.m05888 protein DNA binding protein, putative, expressed no original description | - | -3.57 | Bzip |
| 27.3.35 | RNA.regulation of transcription.bZIP transcription factor family | pgsc0003dmp400053892|pacid:24418620 | very weakly similar to (92.8) AT1G75390 | Symbols: AtbZIP44 | AtbZIP44 (Arabidopsis thaliana basic leucine-zipper 44); DNA binding / protein heterodimerization/ transcription factor | chr1:28292224-28292665 FORWARDvery weakly similar to (89.0) loc_os02g03960 12002.m05745 protein ocs element-binding factor 1, putative, expressed no original description | - | 2.1 | Bzip |
| 27.3.35 | RNA.regulation of transcription.bZIP transcription factor family | pgsc0003dmp400006602|pacid:24384537 | weakly similar to ( 124) AT1G75390 | Symbols: AtbZIP44 | AtbZIP44 (Arabidopsis thaliana basic leucine-zipper 44); DNA binding / protein heterodimerization/ transcription factor | chr1:28292224-28292665 FORWARDvery weakly similar to (93.2) loc_os02g03960 12002.m05745 protein ocs element-binding factor 1, putative, expressed no original description | - | 1.92 | Bzip |
|  |  |  |  |  |  |  |
| 27.3.8 | RNA.regulation of transcription.C2C2(Zn) DOF zinc finger family | pgsc0003dmp400051790|pacid:24428647 | weakly similar to ( 116) AT1G28310 | Symbols: | Dof-type zinc finger domain-containing protein | chr1:9912534-9913685 REVERSEweakly similar to ( 110) PBF_MAIZE Dof zinc finger protein PBF (Prolamin box-binding factor) - Zea mays (Maize)weakly similar to ( 127) loc_os07g32510 12007.m07514 protein expressed proteinzf-Dof no original description | - | 1.7 | DOF |
| 27.3.8 | RNA.regulation of transcription.C2C2(Zn) DOF zinc finger family | pgsc0003dmp400007249|pacid:24394545 | weakly similar to ( 115) AT5G60850 | Symbols: OBP4 | OBP4; DNA binding / transcription factor | chr5:24480578-24481501 FORWARDweakly similar to ( 103) MNB1A_MAIZE Dof zinc finger protein MNB1A - Zea mays (Maize)weakly similar to ( 117) loc_os02g49440 12002.m09978 protein expressed proteinzf-Dof no original description | - | 1.66 | DOF |
| 27.3.8 | RNA.regulation of transcription.C2C2(Zn) DOF zinc finger family | pgsc0003dmp400004435|pacid:24403489 | weakly similar to ( 122) AT1G51700 | Symbols: ADOF1 | ADOF1; DNA binding / transcription factor | chr1:19174266-19174850 FORWARDvery weakly similar to (99.4) PBF_MAIZE Dof zinc finger protein PBF (Prolamin box-binding factor) - Zea mays (Maize)weakly similar to ( 106) loc_os07g32510 12007.m07514 protein expressed proteinzf-Dof no original description | - | -5.81 | DOF |
|  |  |  |  |  |  |  |
| 27.3.3 | RNA.regulation of transcription.AP2/EREBP, APETALA2/Ethylene-responsive element binding protein family | pgsc0003dmp400001547|pacid:24387871 | moderately similar to ( 381) AT1G68840 | Symbols: RAV2, RAP2.8, TEM2 | RAV2 (REGULATOR OF THE ATPASE OF THE VACUOLAR MEMBRANE); DNA binding / transcription factor/ transcription repressor | chr1:25880442-25881500 FORWARDmoderately similar to ( 337) loc_os01g49830 12001.m11168 protein DNA-binding protein RAV1, putative, expressedB3 AP2 AP2 no original description | - | -5.08 | ERF |
| 27.3.3 | RNA.regulation of transcription.AP2/EREBP, APETALA2/Ethylene-responsive element binding protein family | pgsc0003dmp400004686|pacid:24403002 | weakly similar to ( 155) AT5G11590 | Symbols: TINY2 | TINY2 (TINY2); DNA binding / transcription factor | chr5:3727789-3728499 REVERSEweakly similar to ( 127) loc_os10g41130 12010.m06878 protein DREB-like protein, putative, expressedAP2 AP2 no original description | - | 4.42 | ERF |
| 27.3.3 | RNA.regulation of transcription.AP2/EREBP, APETALA2/Ethylene-responsive element binding protein family | pgsc0003dmp400005224|pacid:24378045 | weakly similar to ( 150) AT1G19210 | Symbols: | AP2 domain-containing transcription factor, putative | chr1:6626973-6627530 REVERSEweakly similar to ( 117) loc_os02g54050 12002.m10434 protein transcriptional factor TINY, putative, expressedAP2 AP2 no original description | - | -20.03 | ERF |
| 27.3.3 | RNA.regulation of transcription.AP2/EREBP, APETALA2/Ethylene-responsive element binding protein family | pgsc0003dmp400008715|pacid:24398085 | weakly similar to ( 194) AT1G53910 | Symbols: RAP2.12 | RAP2.12; DNA binding / transcription factor | chr1:20135242-20136581 FORWARDweakly similar to ( 184) ERF1_ORYSA Ethylene-responsive transcription factor 1 (Ethylene-responsive element-binding factor 1) (EREBP-1) (OsEREBP1) - Oryza sativa (Rice)moderately similar to ( 207) loc_os06g09390 12006.m091636 protein ethylene-responsive element binding protein 1, putative, expressedAP2 AP2 no original description | - | -1.8 | ERF |
[truncated: 15,660 more chars]
